# Synthesis of Pyrazolesulfoximines Using α-Diazosulfoximines with Alkynes

**DOI:** 10.1021/acs.orglett.3c04274

**Published:** 2024-02-02

**Authors:** Zhenhao Zhong, Tsz-Kan Ma, Andrew J. P. White, James A. Bull

**Affiliations:** Department of Chemistry, Imperial College London, Molecular Sciences Research Hub, White City Campus, Wood Lane, London W12 0BZ, U.K.

## Abstract

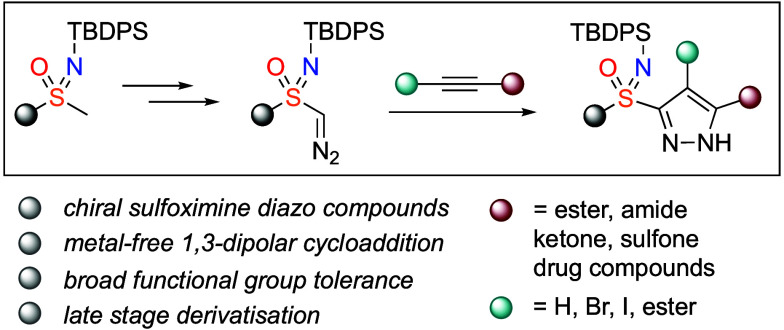

Sulfoximines and pyrazoles are both important motifs in medicinal compounds. Here we report the synthesis and reactivity of sulfoximine diazo compounds as new reagents for the incorporation of sulfoximines. The use of *N*-silyl sulfoximines enabled formation of monosubstituted diazo compounds. Their application is demonstrated in a [3 + 2] cycloaddition with alkynes to form pyrazole sulfoximines in a new combination of these important chemotypes. Further derivatization of the pyrazole sulfoximines is demonstrated, including silyl deprotection to form unprotected pyrazolesulfoximines.

Sulfoximines and pyrazoles are both important motifs in drug discovery. Several sulfoximine-containing compounds have entered clinical trials in recent years, notably as anticancer agents, including Atuveciclib, Roniciclib (Bayer), and Ceralasertib (AstraZeneca, Figure 1a).^1^ These high profile examples and others have established the sulfoximine group as an important and attractive motif in medicinal chemistry.^2,3^ Sulfoximines are now investigated more routinely along with common S(VI) analogues, sulfones, and sulfonamides.^4^ Sulfoximines have been found to offer potentially improved physical and metabolic properties.^5^ Furthermore, as stable chiral motifs they can exploit the additional vector from the sulfoximine nitrogen to probe 3D chemical space.^3,6^ Pyrazoles and their derivatives are much more established as highly important biologically and medicinally relevant motifs.^7^ The numerous pyrazole-derived medicines include Celecoxib, Lonazolac, and Rimonabant, which display diverse biological activities (Figure 1b).^8^ Pyrazoles are commonly prepared by the condensation of hydrazines with 1,3-dicarbonyl compounds or the cycloaddition of diazo compounds as 1,3-dipoles with alkynes.^9^

**Figure 1:**
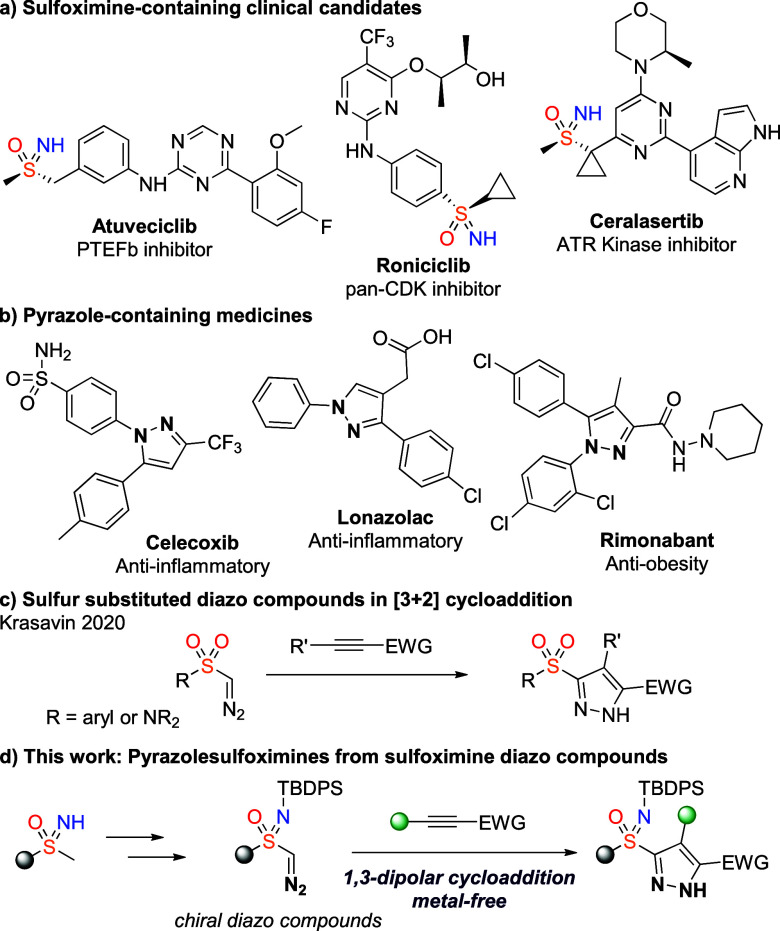
(a) Chiral sulfoximine-containing clinical candidates. (b) Pyrazole-containing drugs. (c) Synthesis of α-pyrazole sulfones and sulfonamides from sulfonyl diazo compounds. (d) Sulfoximine diazo compounds and [3 + 2] dipolar cycloaddition. TBDPS = *tert*-Butyldiphenylsilyl.

With the emergence of sulfoximines in drug discovery, their synthesis has seen notable advances.^3^ Developed strategies include N-transfer to sulfoxides,^10^ N,O transfer to sulfides,^11^ and several approaches to C–S bond formation, involving nucleophilic^12^ and electrophilic sulfur reagents.^13^ Despite this, there remain relatively few sulfoximine containing reagents whereby a preformed sulfoximine motif can be installed in a strategic step. In 2005, Bolm demonstrated the cross-coupling of bromophenyl sulfoximine in Suzuki and Stille cross-coupling and Buchwald–Hartwig amination reactions.^14^ More recently, Lopchuk developed an S_N_Ar protocol with methylsulfoximines to form β-heteroaryl sulfoximines.^15^

We envisaged that the generation of sulfoximine diazo compounds could provide valuable building blocks to allow the introduction of a preformed sulfoximine by exploiting the chemistry of diazo compounds. Isosteric sulfonyl diazo compounds have been recently investigated in various transformations.^16^ This includes reports from Krasavin on the generation of pyrazole-sulfones and -sulfonamides using diazosulfones and diazosulfonamides in the presence of electron-deficient alkynes. On the other hand, sulfoximine containing diazo compounds are unknown. Furthermore, these present an unusual example of α-chiral diazo compounds, of which there are few examples.^17^ Notably, acyclic α-sulfoxide diazo compounds are intrinsically unstable, though cyclic derivatives have been isolated.^18^ Here we report the first synthesis of sulfoximine diazo compounds including an enantioenriched derivative. The nature of the N group is important for their synthesis and stability. A facile [3 + 2] cycloaddition of the diazo compounds with alkynes enables the preparation of sulfoximine pyrazoles and together provides a strategy to combine these important chemotypes.

Studies began using readily available methyltolylsulfoximine, bearing *tert*-butyloxycarbonyl (Boc) or *tert*-butyldiphenylsilyl (TBDPS) protecting groups on the sulfoximine nitrogen. Moreover, the variation in the N-protecting group acts to vary the α-acidity of the sulfoximine compound and so was likely to influence both the steps in the synthesis and the stability of the diazo compounds. N-Boc and N-TBDPS derivatives **1a** and **2a** were carried through an acetylation and diazo transfer sequence (Scheme 1). Deprotonation of the methylsulfoximines with lithium amide bases and quenching with ethyl acetate gave β-ketosulfoximines **3a** and **4a**.^19^ Installation of the diazo group by a Regitz diazo transfer^20^ required different conditions dependent on the N-substituent. For Boc-**5a** nonafluorobutanesulfonyl azide (NfN_3_) and TMEDA as base was required in MeCN;^21^ using *p-*ABSA or TsN_3_ with various amine bases led to decomposition and formation of Boc-sulfinamide. Moreover, the diazo compound slowly decomposed under the reaction conditions, but a good yield was obtained by controlling the reaction time. Silyl derivative **4a** gave sulfoximine diazo-ketone **6a** in good yield using *p-*ABSA and Et_3_N and did not show decomposition over time.^22^ All attempts to form the monosubstituted N-Boc diazo compound were unsuccessful, and this compound could not be obtained, either affording no reaction or decomposition. On the other hand, the TBDPS derivative **8a** was obtained in high yield by methanolysis with catalytic K_2_CO_3_ in MeOH.^23^

**Scheme 1:**
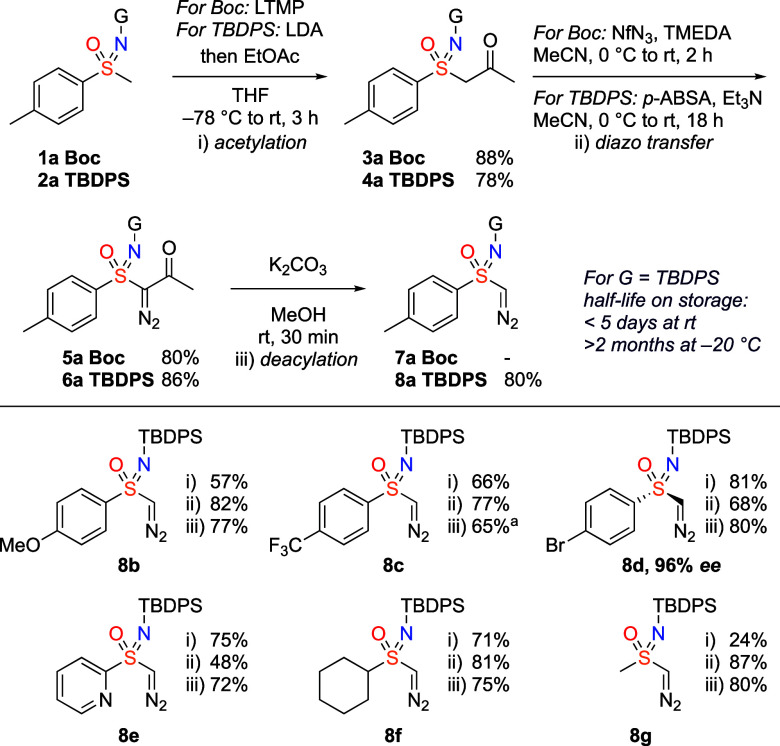
Synthesis of Mono-Substituted Sulfoximine Diazo Compounds 57% at 3 mmol reaction scale. Deacylation step (iii) was performed at 1.0 mmol scale. TBDPS = *tert*-Butyldiphenylsilyl.

With this sequence established, a collection of N-silyl monosubstituted sulfoximine diazo compounds was prepared through acetylation, diazo transfer, and deacetylation. Arylsulfoximine diazo compounds **8a**–**8d** were achieved in good yields with electron-withdrawing and electron-donating substituents. Enantioenriched sulfoximine diazo compound **8d** was generated with retained enantiomeric ratio from the methyl sulfoximine.^24^ Pyridyl (**8e**), cyclohexyl (**8f**), and methyl (**8g**) substituted sulfoximine diazo compounds were similarly successful, demonstrating heteroarene and alkyl substituents commonly of value in medicinal chemistry. It is notable that the diazo compounds were stable to chromatography.

With the collection of diazo compounds available, their reactivity in dipolar cycloaddition reactions with alkynes was assessed to form pyrazolesulfoximines. The pyrazolesulfoximine motif is little known but does appear in patents for possible medicinal (Novartis,^25^ Agios Pharmaceuticals)^26^ and agrochemical applications (PI industries).^27^ Optimization of the [3 + 2] cycloaddition was conducted using sulfoximine diazo **8a** and methylalkynoate. Performing the reaction in toluene was considerably more effective than other solvents tested (MeCN, pentane, THF, Et_2_O, and CH_2_Cl_2_, see the Supporting Information for further details). Using an excess of the alkyne (5 equiv) at room temperature gave an 80% isolated yield of pyrazolesulfoximine **9a** (Scheme 2). Good yields were also obtained using 2 and 1.2 equiv of alkyne (76% and 67% yield, respectively, by ^1^H NMR against an internal standard). Under these conditions, the scope of the reaction to prepare pyrazolesulfoximines was investigated by varying the diazo and alkyne components (Scheme 2, 0.2 mmol scale). Good to excellent yields were achieved on changing the diazo reagent with both electron-donating and electron-withdrawing aryl substituents (**9b**, **9c**). A larger scale protocol for preparation of **9c** (1.3 mmol scale) was conducted using 2 equiv of methyl propiolate, which maintained the high yield. Enantioenriched pyrazolesulfoximine **9d** was prepared with complete retention of the stereochemical information on the highly enantioenriched reagent. Pyridyl- and alkyl-sulfoximine diazo compounds were also successful, providing the corresponding pyrazolesulfoximines with good to excellent yields (**9e**–**9g**).

**Scheme 2:**
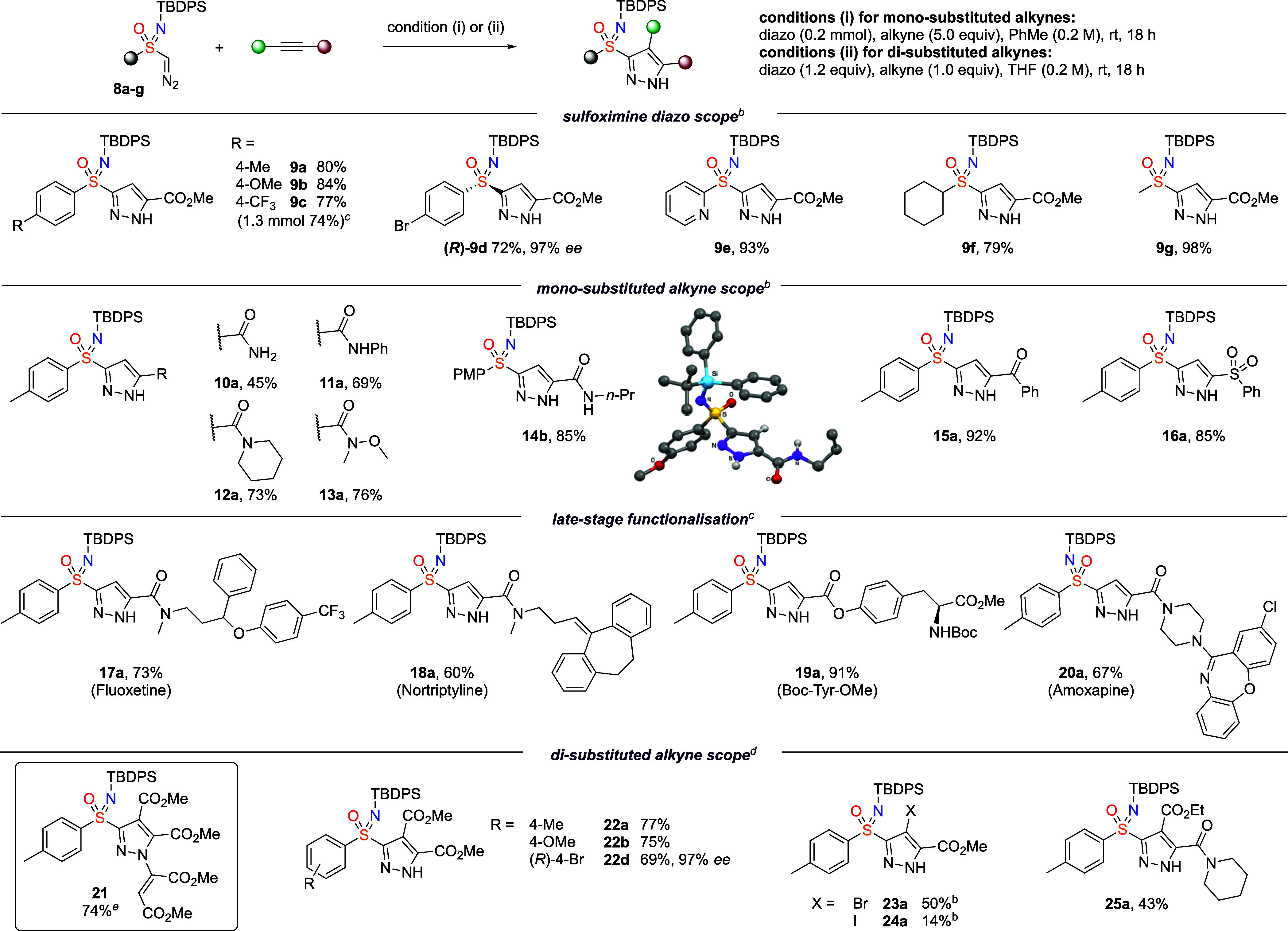
Substrate Scope of Pyrazolesulfoximines Yields of isolated products. Using conditions (i). Using conditions (i) with 2 equiv of alkyne. Using conditions (ii). Using conditions (i) in MeCN as solvent. TBDPS = *tert*-Butyldiphenylsilyl, PMP = *para*-methoxyphenyl.

Various alkynes were successfully applied in the reaction, bearing different electron-withdrawing groups. Primary, secondary, and tertiary amides as well as Weinreb amides were tolerant with moderate to good yields (**10a**–**13a**). Amide **14b** derived from the *para*-methoxyphenyl (PMP) sulfoximine diazo compound was further characterized by X-ray crystallography. Other alkyne derivatives bearing ketone and sulfone functionalities afforded the pyrazoles in good to excellent yields (**15a**, **16a**).

The reaction required at least 1 electron-withdrawing group and was unsuccessful with phenylacetylene. The cycloaddition process was demonstrated in the presence of biologically relevant compounds derived from drug molecules and amino acid derivatives bearing alkyne esters and amides (**17a**–**20a**).

When using dimethyl acetylene dicarboxylate, applying the excess alkyne conditions gave over-reaction, where the pyrazole product underwent conjugate addition to excess alkyne (**21**, Scheme 2). Switching to the alkyne as the limiting reagent with 1.2 equiv of sulfoximine diazo compound and using THF as solvent prevented the formation of **21** and gave **22a** in 77% isolated yield (see the Supporting Information for further discussion). Under these conditions, good yields were witnessed when reacting with disubstituted alkynes (**22a**, **22b**), with retained *ee* using enantioenriched substrate (**22d**). Halo-alkynes introduced bromo- or iodogroups at the pyrazole C3 position (**23a**, **24a**). The use of an unsymmetrical alkyne disubstituted with ester and amide groups gave a single regioisomer assigned as **25a**.^28^ Other disubstituted alkynes, such as methyl phenyl propiolate and ethyl but-2-ynoate, were unreactive, indicating an unfavorable steric interaction, as also seen with iodide **24a**.

The pyrazolesulfoximines were further derivatized to exploit the pendant functionalities (Scheme 3). The ester group could be readily reduced to afford the corresponding alcohol **26**, or hydrolyzed to carboxylic acid **27**, in high yields.

**Scheme 3:**
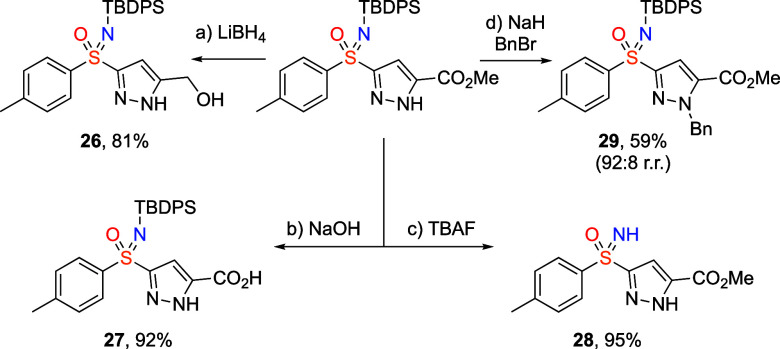
Derivatizations of Pyrazolesulfoximines Conditions: (a) LiBH_4_ (5.0 equiv), THF, 65 °C, 15 min. (b) 1 M aq. NaOH (1.0 mL), MeOH/THF (1:1, 1 mL), rt, 16 h. (c) TBAF (1.5 equiv), THF, 70 °C, 24 h. (d) NaH (1.2 equiv), BnBr (1.2 equiv), THF, 0–25 °C, 96 h. TBDPS = *tert*-Butyldiphenylsilyl.

The silyl group was readily removed using TBAF to give NH sulfoximine **28** in excellent yield. The benzylation of the pyrazole ring was achieved with high regioselectivity (**29**).

In conclusion, the first sulfoximine-containing diazo compounds were synthesized, including an enantioenriched derivative. The synthetic sequence was facile with the N-TBDPS group to provide suitable stability of the diazo compounds. Pyrazolesulfoximines were prepared by a cycloaddition reaction between the sulfoximine diazo compounds and alkynes bearing one or two electron-withdrawing groups. The application of amine-containing drugs and amino acid derivatives showed the potential to afford complex pyrazole sulfoximine analogues. The products were further derivatized, demonstrating the potential to be a useful method to access new chemical space and to provide new vectors for use in drug design. Studies into further applications of the sulfoximine diazo compounds are underway in our laboratories.

## Data Availability

The data underlying this study are available in the published article and its Supporting Information. All characterization data for synthesized compounds can be found at https://data.hpc.imperial.ac.uk/resolve/?doi=13396.

## Supporting Information Available

Experimental procedures, characterization data, and copies of ^1^H and ^13^C NMR spectra; summaries of reaction optimization for diazo transfer, deacetylation, and cycloaddition reactions; X-ray crystallography data; and HPLC traces for enantioenriched compounds (PDF)
