# Supplementary material for: Synthesis of Pyrazolesulfoximines Using α-Diazosulfoximines with Alkynes
Source: Org Lett. 2024 Feb 2;26(6):1178–83. doi: 10.1021/acs.orglett.3c04274 (PMC10877601; doi:10.1021/acs.orglett.3c04274)
Supplement: Supplementary file 1 — ol3c04274_si_001.pdf [file ol3c04274_si_001.pdf]

SUPPORTING INFORMATION

Synthesis of Pyrazolesulfoximines using α-Diazosulfoximines with Alkynes

Zhenhao Zhong,[a] Tsz-Kan Ma,[a] Andrew J. P. White,[a] and James A. Bull*,[a]

[a] Department of Chemistry, Imperial College London, Molecular Sciences Research Hub, White City Campus, Wood Lane, London W12 0BZ, UK.

E-mail: j.bull@imperial.ac.uk

General Experimental Considerations	S2
Structure of Additional Compounds in SI	S3
Synthesis of Pyrazolesulfoximines	S4
Optimisation of Diazo Transfer to β-Ketone Sulfoximine	S4
Optimisation of De-acylation of Sulfoximine Diazo Compound	S5
Optimisation of Cycloaddition with mono-Substituted Alkyne	S6
Optimisation of Cycloaddition with di-Substituted Alkyne	S7
Unsuccessful Alkynes in Cycloaddition Scope	S8
Regioisomeric Assignment of Compound **25a**	S9
Regioisomeric Assignment of Compound **29**	S9
X-Ray Crystallography Details of Compound **14b**	S10
Experimental Details and Characterisation Data	S13
General Procedure A for Sulfide Preparation (**S1b, S1c, S1e, S1f**)	S13
General Procedure B for Sulfoximine Preparation (**S1a**–**c**, **S1e**–**g**)	S14
General Procedure C for TBDPS Protection (**2a**–**g**)	S17
General Procedure D for Acetylation (**4a**–**g**)	S21
General Procedure E for Diazo Transfer (**6a**–**g**)	S25
Synthesis of Boc-protected Sulfoximine Diazo Compounds (**3a** and **5a**)	S28
General Procedure F for De-acetylation (**8a**–**g**)	S29
Preparation of Alkyne Starting Materials (**S11**–**25**)	S33
General Procedure G for Cycloaddition with mono-Substituted Alkynes (**9**–**21**)	S40
General Procedure H for Cycloaddition with di-Substituted Alkynes (**22**–**25**)	S50
Derivatisation of Pyrazolesulfoximines (**26**–**29**)	S54
^{1}H and ^{13}C Spectra of Selected Compounds	S57
HPLC Traces of Enantioenriched Substrates and Products	S124
References	S130

General Experimental Considerations

All reactions were run under an inert atmosphere (argon) with flame-dried glassware using standard techniques unless otherwise stated. Anhydrous solvents were obtained by filtration through drying columns (THF, toluene, EtOH, MeOH, DMF, MeCN, CH_2Cl_2). *n*-Butylithium solution (1.6 M in hexane) was purchased from Sigma-Aldrich and titrated with 2-hydroxybenzaldehyde phenylhydrazone before use. Anhydrous EtOAc was purchased from thermo scientific and used as provided. $PhI(OAc)_2$, TBDPSCl, *p*-ABSA and methyl propiolate were purchased from Fluorochem and used as provided. Dimethylacetylenedicarboxylate was purchased from Sigma-Aldrich and used as provided. All other commercial reagents were used as supplied or purified by standard techniques where necessary. Flash column chromatography was performed using 230-400 mesh silica with the indicated solvent system according to standard techniques. Analytical thin-layer chromatography (TLC) was performed on precoated, glass-backed silica gel plates. Visualisation of the developed chromatogram was performed by UV absorbance (254 nm), aqueous potassium permanganate, *p*-anisaldehyde, phosphomolybdic acid or vanillin stains. Infrared spectra (ν_{max}, FTIR-ATR) were recorded in reciprocal centimeters (cm^{-1}). Nuclear magnetic resonance (NMR) spectra were recorded on 400 MHz spectrometers. Chemical shifts for ^{1}H NMR spectra are recorded in parts per million from tetramethylsilane with the solvent resonance as the internal standard (chloroform: δ = 7.27 ppm, DMSO: δ = 2.50 ppm, MeOH: δ = 3.31 ppm). Data is reported as follows: chemical shift [multiplicity (s = singlet, d = doublet, t = triplet, q = quartet, pent = pentet, m = multiplet and b = broad), coupling constant in Hz, integration, assignment]. ^{13}C NMR spectra were recorded with complete proton decoupling. Chemical shifts are reported in parts per million from tetramethylsilane with the solvent resonance as the internal standard (chloroform: δ = 77.0 ppm, DMSO: δ = 39.5 ppm, MeOH: δ = 49.0 ppm). *J* values are reported in Hz. Assignments of $^{1}H/^{13}C$ spectra were made by the analysis of δ/J values, and HSQC experiments as appropriate. ^{19}F NMR spectra were recorded without complete proton decoupling unless otherwise stated. ^{19}F NMR spectra are indirectly referenced to $CFCl_3$ automatically via direct measurement of the absolute frequency of the deuterium lock signal by the spectrometer hardware. Melting points are uncorrected.

The high-resolution mass spectrometry (HRMS) analyses were performed using electrospray ion source (ESI) or pneumatically assisted atmospheric pressure chemical ionization (APCI) using an atmospheric solids analysis probe (ASAP). ESI was performed using a Waters LCT Premier equipped with an ESI source operated in positive or negative ion mode. The software used was MassLynx 4.1. This software does not account for the electron and all the calibrations/references are calculated accordingly, i.e. $[M+H]^+$ is detected and the mass is calibrated to output [M+H]. APCI was performed using an Orbitrap XL or Xevo G2S using an ASAP to insert samples into the APCI source. The sample was introduced at ambient temperature and the temperature increased until the sample vaporised.

The enantioenriched and racemic methyl *p*-bromophenyl sulfoximine were used synthesized according to the literature procedure.[1]

Notes:

For the diazo compounds and the α-pyrazole sulfoximine compounds prepared, the resonance for the fully substituted C=N=N carbon in the ^{13}C NMR spectrum is often not seen due to quadrupole coupling to ^{14}N. The ^{13}C resonance is therefore only reported when observed.

Structure of Additional Compounds in SI

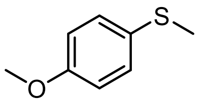

S1b

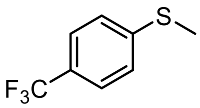

S1c

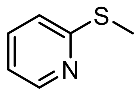

S1e

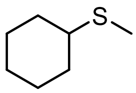

S1f

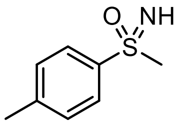

S1a

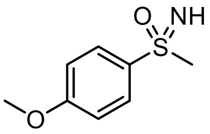

S1b

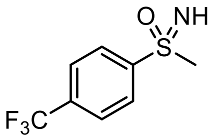

S1c

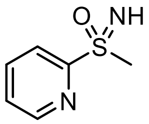

S1e

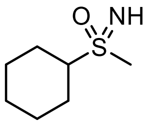

S1f

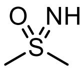

S1g

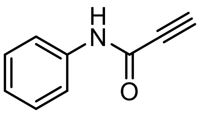

S11a

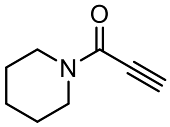

S12a

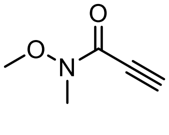

S13a

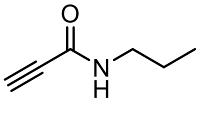

S14b

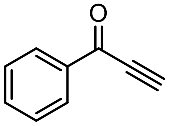

S15a

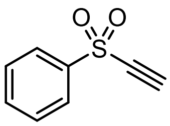

S16a

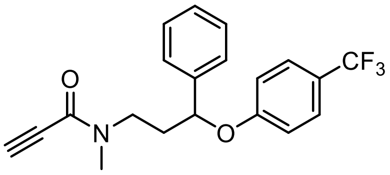

S17a

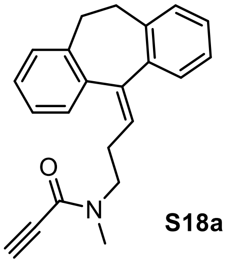

S18a

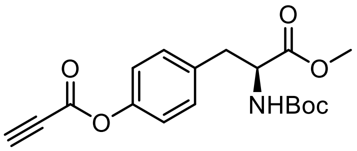

S19a

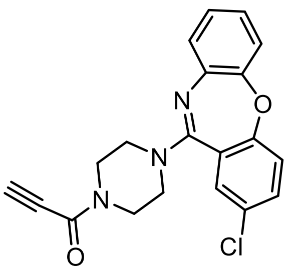

S20a

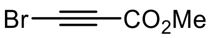

S23a

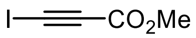

S24a

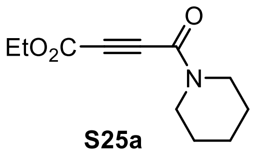

S25a

Synthesis of Pyrazolesulfoximines

Selected Optimisation of Diazo Transfer to β-Ketone Sulfoximine

The optimisation of diazo transfer to TBDPS-protected β-ketone sulfoximine was shown in Table S1. Using *p*-ABSA as diazo transfer agent in the presence of DBU afforded the diazo product with a low yield, presumably because of the decomposition of the diazo compound in the presence of strong base. Switching to Et_3N showed an increase in yield to 77%, and no decomposition of diazo product was observed. Using the reaction conditions developed for Boc-protected substrate also afforded the product with a good yield. Given the commercial availability of the diazo transfer agent, the generation of the TBDPS-protected diazo compound was conducted with *p*-ABSA.

Table S1. Optimisation of the diazo transfer to β-ketone sulfoximine

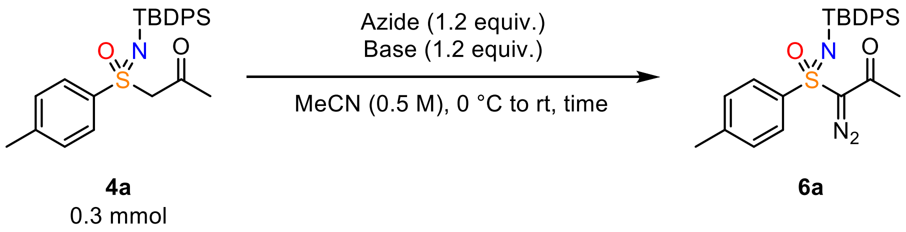

Entry	Azide	Base	Time	Yield[a]
1	*p*-ABSA	DBU	10 min	28%[b]
**2**	***p*-ABSA**	**Et_3N**	**18 h**	**77%**
3	NfN_3	TMEDA	18 h	72%

[a] Yield of diazo determined by the analysis of ^1H NMR using 1,3,5-trimethoxybenzene as an internal standard. [b] No recovery of starting material.

Selected Optimisation of De-Acetylation of Sulfoxmine Diazo Compound

The optimisation of the de-acetylation with TBDPS protecting group was shown in Table S2. Using Lewis acid Al_2O_3 in CH_2Cl_2 afforded the mono-substituted product with a 23% yield, where no recovery of the starting material was witnessed. Switching to methanolysis conditions was successful, showing the mono-substituted product could be achieved in high yield with catalytic amount of K_2CO_3.

Table S2. Optimisation of the de-acetylation to afford mono-substituted sulfoximine diazo compound

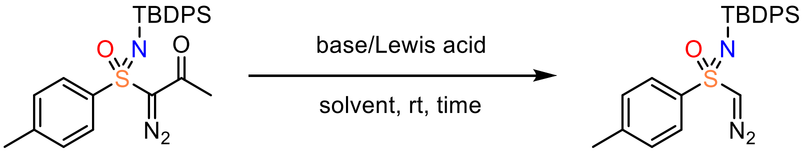

6a 0.3 mmol **8a**

Entry	base/Lewis acid (equiv)	solvent	time (h)	Yield[a]
1	Al_2O_3 (70 equiv)	CH_2Cl_2 (0.1 M)	8	23%[b]
2	Na_3PO_4 (0.5)	MeOH (1.0 M)	1	75%
**3**	**K_2CO_3 (0.1)**	**MeOH (0.06 M)**	**0.5**	**80%**

[a] Yield of diazo determined by the analysis of ^{1}H NMR using 1,3,5-trimethoxybenzene as the internal standard. [b] no recovery of starting material.

Optimisation of Cycloaddition with Mono-Substituted Alkyne

The cycloaddition with mono-substituted alkyne was optimised between *p*-tolyl sulfoximine diazo compound and methyl propiolate. PhMe was found the optimal reaction solvent to generate 5-substituted pyrazole (80% isolated yield). Reducing the alkyne equivalents was tolerated but with slightly slower reaction rates and lower yields.

Table S3. Optimization for the formation of 5-substituted pyrazole sulfoximine

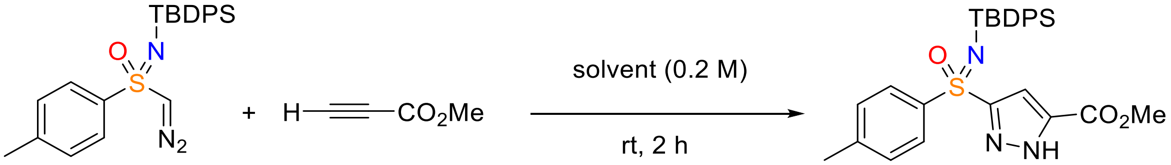

entry	solvent	alkyne equiv	yield of **9a** (%)[a]
1	pentane	5.0	trace
2	MeCN	5.0	34
3	THF	5.0	52
4	Et_2O	5.0	57
5	CH_2Cl_2	5.0	66
**6**	**PhMe**	**5.0**	**79(80)**[b]
7	PhMe	2.0	76
8	PhMe	1.2	67

[a] Yield calculated by analysis of the ^{1}H NMR spectrum of the crude mixture of the reaction using 1,3,5-trimethoxybenzene as internal standard. [b] Isolated yields in parenthesis.

Optimisation of Cycloaddition with Di-Substituted Alkyne

The cycloaddition with a di-substituted alkyne was optimised between *p*-tolyl sulfoximine diazo compound and dimethyl acetylene dicarboxylate. Using the alkyne in excess led to further conjugate addition between the pyrazolesulfoximine and excess alkyne, so a slight excess of sulfoximine diazo compound was applied to avoid over-reaction. THF was found the optimal reaction solvent to generate 4,5-disubstituted pyrazole (77% isolated yield) with low yielding on side product **21**, which is also beneficial to chromatographic separation.

Table S4. Optimization for the formation of 4,5-disubstituted pyrazole sulfoximine

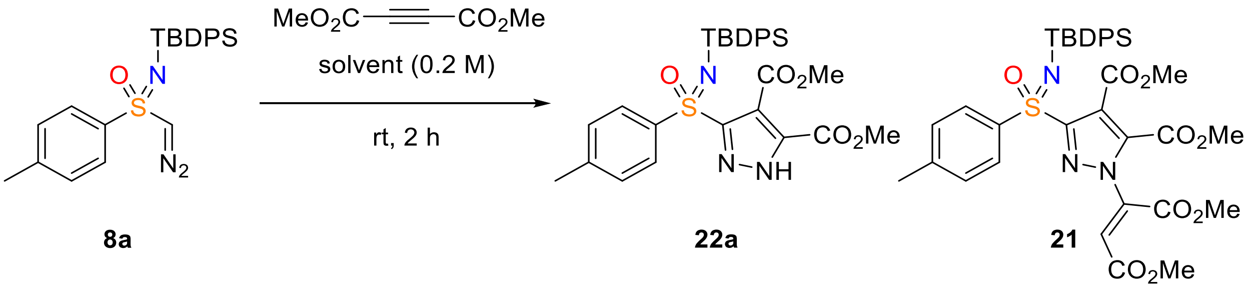

entry[a]	solvent	diazo:alkyne	yield of **22a** (%)[b]	yield of **21** (%)[b]
1	MeCN	1:5	trace	(74)
2	MeCN	1.2:1	78	15
3	PhMe	1.2:1	46	10
4	CH_2Cl_2	1.2:1	58	5
5	pentane	1.2:1	78	2
**6**	**THF**	**1.2:1**	**89 (77)[c]**	**2**

[a] Reactions performed on a 0.2 mmol scale [b] Yield calculated by analysis of the ^{1}H NMR spectrum of the crude mixture of the reaction using 1,3,5-trimethoxybenzene as internal standard. [c] Isolated yields in parenthesis.

Unsuccessful Alkynes in Cycloaddition Scope

Figure S1 showed the unsuccessful alkyne examples attempted in cycloaddition scope. The decomposition of sulfoximine diazo compound was observed in the presence of an alkyne containing acidic protons. No reaction was witnessed between sulfoximine diazo compound and methyl phenyl propiolate and phenylacetylene (e.g. phenylacetylene), indicating that one electron-withdrawing activating group and low steric demand were required. With unreactive alkynes only slow thermal decomposition of sulfoximine diazo compound was witnessed.

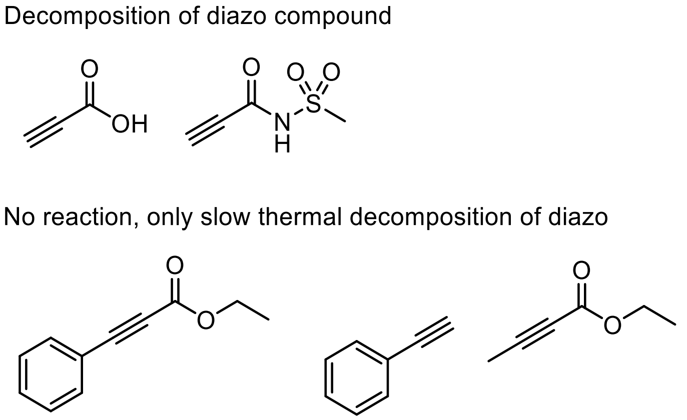

Figure S1. Unsuccessful alkyne examples in cycloaddition scope.

Regioisomeric Assignment of Compound 25a

The regioisomer for compound **25a** was not definitively determined through using NOE and other NMR experiments. NOE experiments were inconclusive as there was no correlation between the TBDPS group and the either ester or amide to indicate proximity.

The assignment as regioisomer A was therefore made on the basis of the expected steric and electronic features. The smaller ester substituent would be expected to adopt the C3 position, with the amide away from the bulky sulfoximine in the cycloaddition. This is consistent with the expected leading interactions in the 1,3-dipolar cycloaddition.

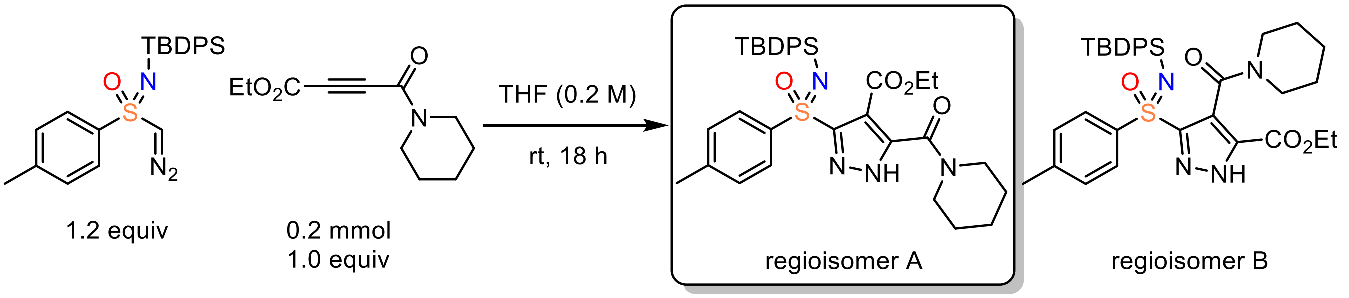

Scheme S1. Regioisomeric assignment of Compounds 25a

Regioisomeric Assignment of Compound 29

The regioisomer for compound **29** was not definitively determined and was assigned as regioisomer A on the basis of the steric demands. The benzyl group was assumed to be adjacent to the ester, rather than the bulky, tetrahedral sulfoximine group. There was no NOE correlation between the Bn group and the *p*-tolyl or TBDPS groups.

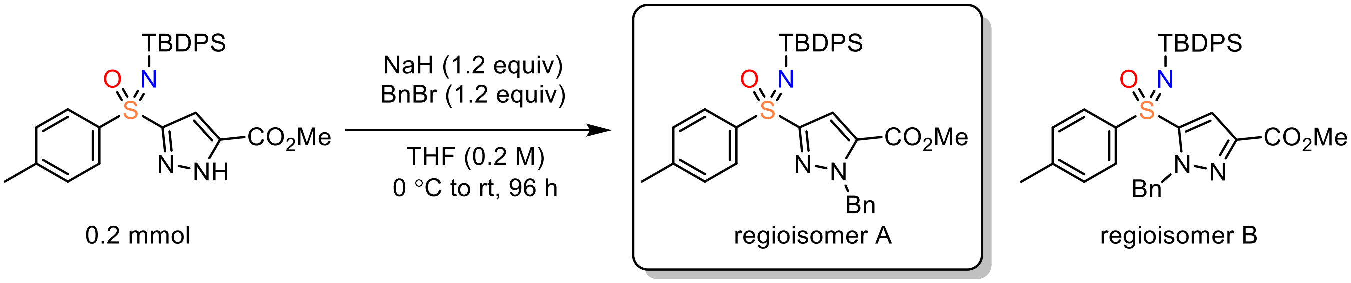

Scheme S2. Regioisomeric assignment of Compounds 29

X-Ray Crystallography Details of Compound 14b

Crystals suitable for X-ray analysis were grown by vapor diffusion from THF/cyclohexane at 23 °C.

Crystal data for **14b**: $C_{30}H_{36}N_4O_3SSi$, M = 560.78, triclinic, P-1 (no. 2), a = 11.9158(3), b = 24.0227(8), c = 24.7882(8) Å, α = 61.727(3), β = 81.419(3), γ = 76.758(3)°, V = 6076.1(4) Å^3, Z = 8 [4 independent molecules], D_c = 1.226 g cm^{-3}, μ(Cu-Kα) = 1.615 mm^{-1}, T = 173 K, colourless blocky needles, Agilent Xcalibur PX Ultra A diffractometer; 23176 independent measured reflections (R_{int} = 0.0382), F^2 refinement,[X1,X2,X3] R_1(obs) = 0.0449, wR_2(all) = 0.1286, 17203 independent observed absorption-corrected reflections [$|F_o| > 4\sigma(|F_o|)$, completeness to θ_{full}(67.7°) = 98.3%], 1498 parameters. CCDC 2305547.

The structure of **14b** was found to contain four crystallographically independent molecules (**14b-A**, **14b-B**, **14b-C** and **14b-D**) in the asymmetric unit. The C7 to C11 *n*-propylamide group of molecules **14b-C** and **14b-D** was found to be disordered, and in each case two orientations were identified, of *ca*. 72:28 and 78:22% occupancy respectively. The geometries of each pair of orientations were optimised, the thermal parameters of adjacent atoms were restrained to be similar, and only the non-hydrogen atoms of the major occupancy orientations were refined anisotropically (those of the minor occupancy orientations were refined isotropically). The N4–H hydrogen atoms of all four independent molecules, and the N8–H hydrogen atoms of molecules **14b-A** and **14b-B**, were located from Δ*F* maps and refined freely subject to an N–H distance constraint of 0.90 Å. The N8–H hydrogen atoms of the partial occupancy orientations of the disordered *n*-propylamide groups in molecules **14b-C** and **14b-D** could not be reliably located and so they were added in idealised positions with N–H distances of 0.90 Å.

References

[X1] O.V. Dolomanov, L.J. Bourhis, R.J. Gildea, J.A.K. Howard, H. Puschmann, *J. Appl. Cryst.*, 2009, **42**, 339-341.
[X2] SHELXTL v5.1, Bruker AXS, Madison, WI, 1998.
[X3] SHELX-2013, G.M. Sheldrick, *Acta Cryst.*, 2015, **C71**, 3-8.

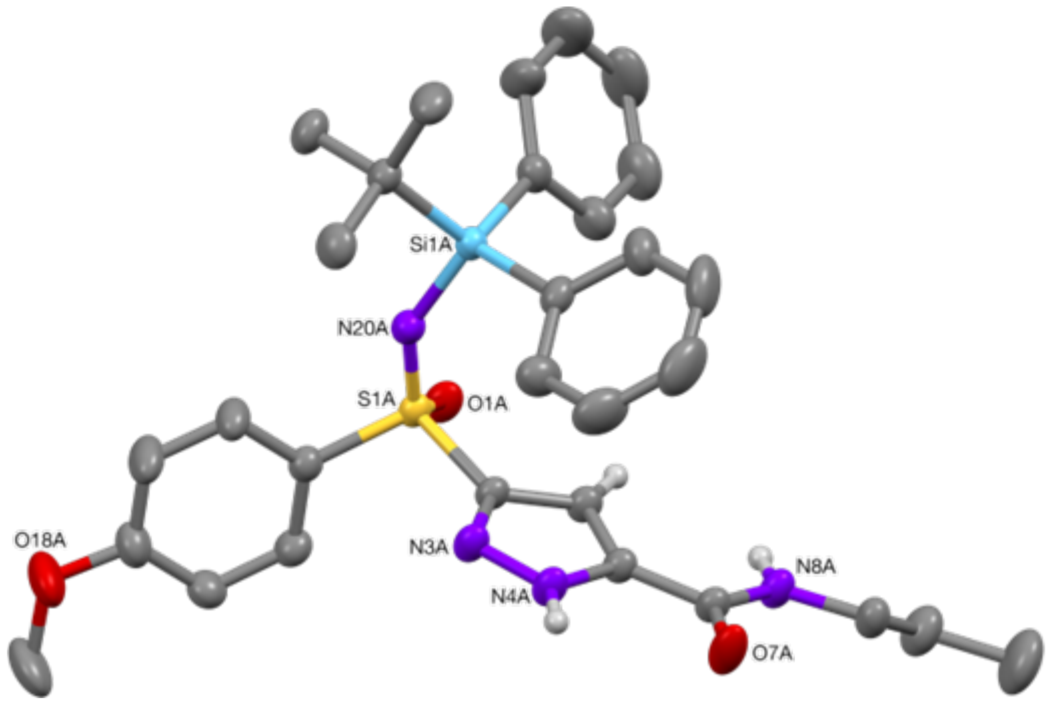

Fig. S1 The structure of **14b-A**, one of the four independent molecules present in the crystal of **14b** (50% probability ellipsoids).

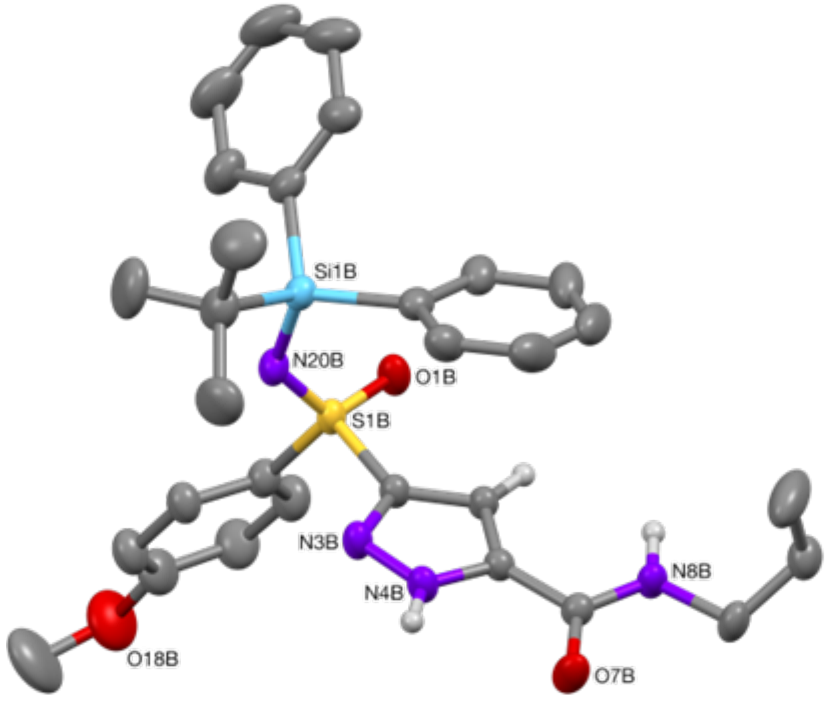

Fig. S2 The structure of **14b-B**, one of the four independent molecules present in the crystal of **14b** (50% probability ellipsoids).

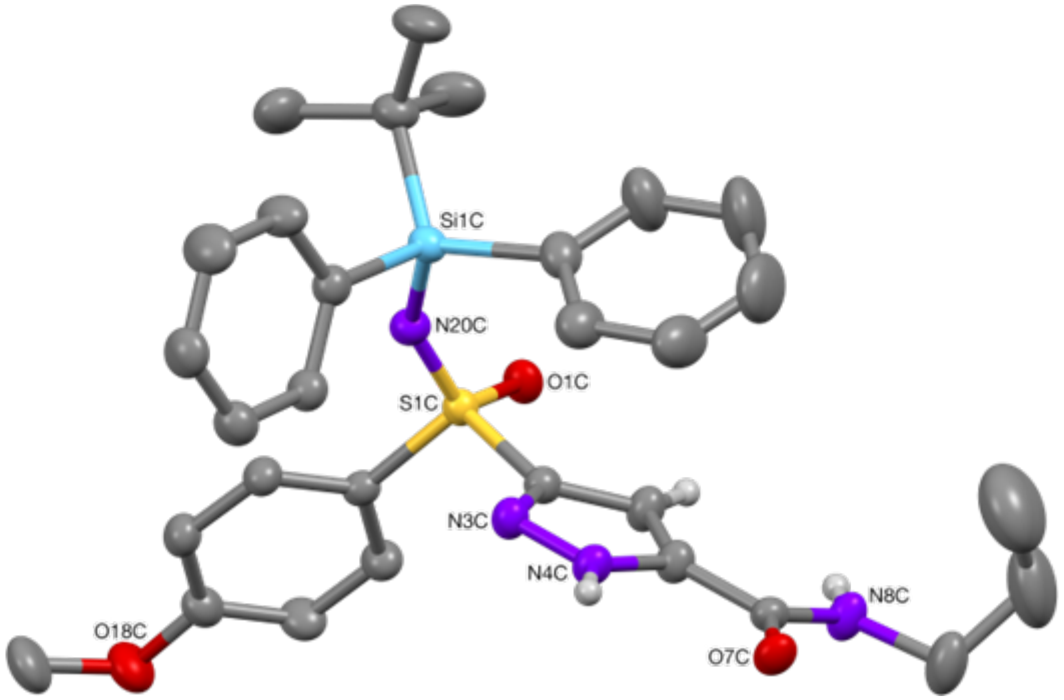

Fig. S3 The structure of **14b-C**, one of the four independent molecules present in the crystal of **14b** (50% probability ellipsoids).

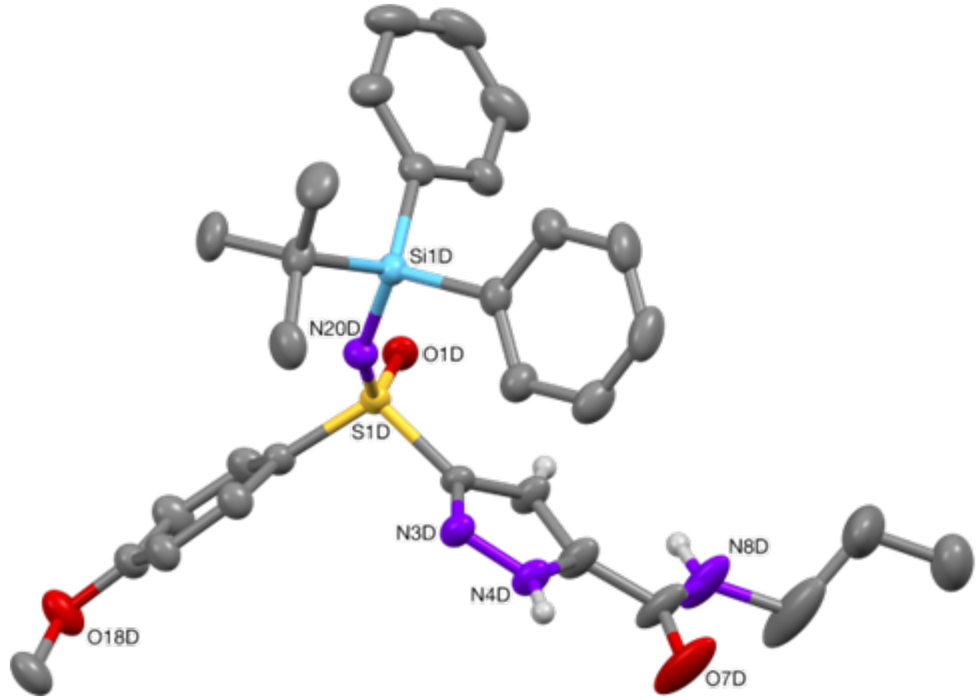

Fig. S4 The structure of **14b-D**, one of the four independent molecules present in the crystal of **14b** (50% probability ellipsoids).

Experimental Details and Characterisation Data

General Procedure A for Sulfide Preparation

Sodium hydroxide (1.5 equiv) was added to the corresponding thiol (1.0 equiv) in anhydrous EtOH (ca. 1.2 mL/mmol with reference to thiol) at rt and stirred for 30 min. The mixture was cooled down to 0 °C and iodomethane (1.5 equiv) was added dropwise. Then the resulting mixture was warmed to rt for 3 h followed by concentration under reduced pressure. H_2O (50 mL) was added, and the mixture was extracted with CH_2Cl_2 (3 × 50 mL). The combined organic layer was dried over Na_2SO_4 and concentrated under reduced pressure to afford the corresponding sulfide which was used without further purification.[2]

(4-Methoxyphenyl)(methyl)sulfane (S1b)

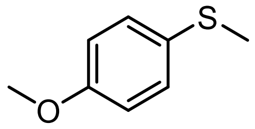

Prepared using general procedure A with 4-methoxybenzenethiol (8.6 mL, 70 mmol, 1.0 equiv), iodomethane (6.5 mL, 105 mmol, 1.5 equiv) and NaOH (4.2 g, 105 mmol, 1.5 equiv) in EtOH (85 mL). **S1b** was afforded as a colourless oil (10.6 g, 98%). IR (film)/cm^{-1} 2996, 2940, 2917, 2832, 1592, 1490, 1436, 1281, 1238, 1175, 1028, 818, 621; ^{1}H NMR (400 MHz, $CDCl_3$) δ 7.28 (d, *J* = 8.8 Hz, 2H, 2 × Ar–H), 6.86 (d, *J* = 8.8 Hz, 2H, 2 × Ar–H), 3.80 (s, 3H, OCH_3), 2.46 (s, 3H, SCH_3); ^{13}C NMR (101 MHz, $CDCl_3$) δ 158.2 (Ar–C_q), 130.2 (2 × Ar–C), 128.7 (Ar–C_q), 114.6 (2 × Ar–C), 55.3 (OCH_3), 18.1 (SCH_3). Analytical data (NMR and IR) in agreement with those reported in the literature.[3]

Methyl(4-(trifluoromethyl)phenyl)sulfane (S1c)

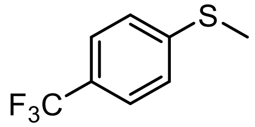

Prepared using general procedure A with 4-trifluoromethylbenzenethiol (6.8 mL, 50 mmol, 1.0 equiv), iodomethane (4.7 mL, 75 mmol, 1.5 equiv) and NaOH (3.0 g, 75 mmol, 1.5 equiv) in EtOH (60 mL). **S1c** was afforded as a pale-orange oil (8.9 g, 93%). IR (film)/cm^{-1} 2979, 2924, 2101, 1603, 1402, 1321, 1156, 1087, 1158, 1007, 966, 814, 587, 492; ^{1}H NMR (400 MHz, $CDCl_3$) δ 7.53 (d, *J* = 8.3 Hz, 2H, 2 × Ar–H), 7.31 (d, *J* = 8.2 Hz, 2H, 2 × Ar–H), 2.52 (s, 3H, SCH_3); ^{19}F NMR (377 MHz, $CDCl_3$) δ –62.3; ^{13}C NMR (101 MHz, $CDCl_3$) δ 143.8 (Ar–C_q), 126.8 (q, *J* = 33.0 Hz, *C*CF_3), 125.6 (2 × Ar–C), 125.5 (2 × Ar–C), 124.2 (q, *J* = 271.4, CF_3), 122.9 (Ar–C_q), 15.0 (SCH_3). Analytical data (NMR and IR) in agreement with those reported in the literature.[4]

2-(Methylthio)pyridine (S1e)

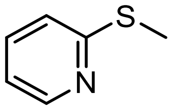

Prepared using general procedure A with 2-mercaptopyridine (7.8 g, 70 mmol, 1.0 equiv), iodomethane (6.5 mL, 105 mmol, 1.5 equiv) and NaOH (4.2 g, 105 mmol, 1.5 equiv) in EtOH (85 mL). **S1e** was afforded as a colourless oil (7.4 g, 84%). IR (film)/cm^{-1} 3065, 3043, 2992, 2923, 1577, 1452, 1412, 1123, 754, 732, 618; ^{1}H NMR (400 MHz, $CDCl_3$) δ 8.45–8.43 (m, 1H, Ar–H), 7.50–7.45 (m, 1H, Ar–H), 7.19–7.16 (m, 1H, Ar–H), 6.98–6.95 (m, 1H, Ar–H), 2.56 (s, 3H, SCH_3); ^{13}C NMR (101 MHz, $CDCl_3$) δ 159.9 (Ar–C_q), 149.4 (Ar–C), 135.7 (Ar–C), 121.4 (Ar–C), 119.0 (Ar–C), 13.2 (SCH_3). Analytical data (NMR and IR) in agreement with those reported in the literature.[5]

Cyclohexyl(methyl)sulfane (S1f)

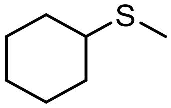

Prepared using general procedure A with cyclohexanethiol (8.6 mL, 70 mmol, 1.0 equiv), iodomethane (6.5 mL, 105 mmol, 1.5 equiv) and NaOH (4.2 g, 105 mmol, 1.5 equiv) in EtOH (85 mL). **S1f** was afforded as a colourless oil (4.0 g, 44%). IR (film)/cm^{-1} 2923, 2851, 1445, 1265, 1203, 1003, 885, 744, 717; ^{1}H NMR (400 MHz, $CDCl_3$) δ 2.57–2.52 (m, 1H, SCH), 2.09 (s, 3H, SCH_3), 2.01–1.96 (m, 2H, CH_2), 1.80–1.75 (m, 2H, CH_2), 1.65–1.60 (m, 1H, C*H*H), 1.36–1.22 (m, 5H, CH*H*(C*H*$_2$)$_2$); ^{13}C NMR (101 MHz, $CDCl_3$) δ 44.9 (SCH), 33.1 (SCH(*C*H$_2$)$_2$), 26.1 (SCH(CH$_2$*C*H$_2$)$_2$), 25.9(CH_2), 13.2 (SCH_3). Analytical data (NMR and IR) in agreement with those reported in the literature.[6]

General Procedure B for Sulfoximine Preparation

Prepared using a method developed by Bull and Luisi.[7] $PhI(OAc)_2$ (2.1 equiv) was added portion-wise to a stirred solution of sulfide (1.0 equiv) and NH_4Oac (2.0 equiv) in MeOH (2 mL/mmol with reference to sulfide) at rt under air. The resulting mixture was stirred at rt for 3 h. Subsequently, the mixture was concentrated under reduced pressure, quenched with saturated aqueous $NaHCO_3$ solution (150 mL) and extracted with CH_2Cl_2 (3 × 50 mL). The combined organic layer was dried over Na_2SO_4 and concentrated under reduced pressure. Purification by flash column chromatography (SiO_2) afforded the corresponding sulfoximine.

Imino(methyl)(*p*-tolyl)-λ^{6}-sulfanone (S2a)

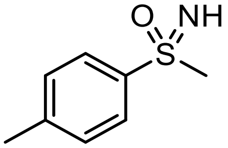

Prepared using general procedure B with methyl *p*-tolyl sulfide (6.9 g, 50 mmol, 1.0 equiv), $PhI(Oac)_2$ (33.8 g, 105 mmol, 2.1 equiv) and NH_4Oac (7.7 g, 100 mmol, 2.0 equiv) in MeOH (100 mL). Purification by flash column

chromatography (SiO_2, 5% EtOH in EtOAc) afforded sulfoximine **S2a** as an off-white solid (6.9 g, 76%). R_f = 0.18 (EtOAc); R_f 0.18 (EtOAc). Mp: 69-70 °C. IR (film)/cm^{-1} 3279, 2920, 1593, 1404, 1218, 1092, 1000, 814, 745, 543. ^{1}H NMR (400 MHz, $CDCl_3$) δ 7.88 (d, *J* = 8.3 Hz, 2H, 2 × Ar–H), 7.35–7.33 (m, 2H, 2 × Ar–H), 3.09 (s, 3H, SCH_3), 2.65 (br, 1H, NH), 2.44 (s, 3H, Ar–CH_3). ^{13}C NMR (101 MHz, $CDCl_3$) δ 143.9 (Ar–C_q), 140.5 (Ar–C_q), 129.8 (2 × Ar–C), 127.7 (2 × Ar–C), 46.3 (SCH_3), 21.5 (Ar–CH_3). Analytical data (NMR and IR) in agreement with those reported in the literature.[8]

Imino(4-methoxyphenyl)(methyl)-λ^6-sulfanone (S2b)

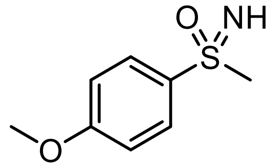

Prepared using general procedure B with sulfide **S1b** (7.7 g, 50 mmol, 1.0 equiv), PhI(Oac)$_2$ (33.8 g, 105 mmol, 2.1 equiv) and NH_4Oac (7.7 g, 100 mmol, 2.0 equiv) in MeOH (100 mL). Purification by flash column chromatography (SiO_2, 5% EtOH in EtOAc) afforded sulfoximine **S2b** as a colourless oil (8.8 g, 95%). R_f = 0.11 (EtOAc); IR (film)/cm^{-1} 3266, 2839, 2112, 1592, 1494, 1309, 1253, 1212, 1094, 1016, 832, 731; ^{1}H NMR (400 MHz, $CDCl_3$) δ 7.92 (d, *J* = 8.9 Hz, 2H, 2 × Ar-H), 6.99 (d, *J* = 8.7 Hz, 2H, 2 × Ar-H), 3.86 (s, 3H, OCH_3), 3.09 (s, 3H, SCH_3); ^{13}C NMR (101 MHz, $CDCl_3$) δ 163.3 (Ar–C_q), 129.9 (Ar–C_q), 129.8 (2 × Ar–C), 114.3 (2 × Ar–C), 55.6 (OCH_3), 46.4 (SCH_3); Analytical data (NMR and IR) in agreement with those reported in the literature.[10]

1-(Methylsulfinyl)-4-(trifluoromethyl)benzene (S2c')[9]

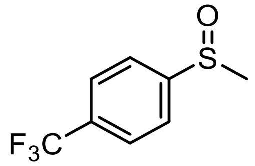

H_2O_2 (30% in H_2O, 1.9 mL, 40 mmol, 1.0 equiv) was added dropwise to a stirred solution of **S1c** in AcOH (8 mL) at 0 °C. The resulting mixture was slowly warmed to rt for 18 h. The reaction mixture was diluted with CH_2Cl_2 (50 mL), quenched with $NaHCO_3$ (50 mL) and extracted with CH_2Cl_2 (2 × 50 mL). The combined organic layer was dried over Na_2SO_4 and concentrated under reduced pressure to afford the corresponding sulfoxide **xx** as a white solid (7.9 g, 95%) which was used without further purification.

Imino(methyl)(4-(trifluoromethyl)phenyl)-λ^6-sulfanone (S2c)

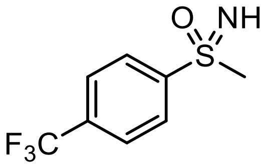

Prepared using the method developed by Bolm.[10] PhI(Oac)$_2$ (14.5 g, 45 mmol, 1.5 equiv) was added to a stirred solution of sulfoxide **S2c'** (6.25 g, 30 mmol, 1.0 equiv) and Rh$_2$(Oac)$_4$ (530 mg, 1.2 mmol, 4 mol%) and CF$_3$CONH$_2$ (6.8 g, 60 mmol, 2.0 equiv) and MgO (4.8 g, 120 mmol, 4.0 equiv) in CH$_2$Cl$_2$ (300 mL). The resulting mixture was stirred at rt for 18 h. The mixture was filtered through celite and concentrated under reduced pressure. Purification by flash column chromatography (SiO$_2$, 25% EtOAc in hexane) afforded *N*-trifluoroacetyl sulfoximine as a colourless oil (8.0 g, 84%). K$_2$CO$_3$ (17.3 g, 125 mmol, 5.0 equiv) was added to a stirred solution of *N*-trifluoroacetyl sulfoximine (8.0 g, 25 mmol, 1.0 equiv) in MeOH (250 mL) and the resulting mixture was stirred at rt for 2 h. The mixture was concentrated under reduced pressure followed by the addition of saturated aqueous NaHCO$_3$ (150 mL) and extraction with CH$_2$Cl$_2$ (3 × 50 mL). The combined organic layer was dried over Na$_2$SO$_4$ and concentrated under reduced pressure to afford sulfoximine **S2c** (5.4 g, 97%) as a colourless oil without further purification. IR (film)/cm^{-1} 3271, 3010, 3082, 2919, 2104, 1399, 1318, 1221, 1125, 1094, 1058, 944, 835, 695, 593. ^{1}H NMR (400 MHz, CDCl$_3$) δ 8.16 (d, *J* = 8.1 Hz, 2H, 2 × Ar-H), 7.84 (d, *J* = 8.2 Hz, 2H, 2 × Ar-H), 3.14 (s, 3H, SCH$_3$), 2.79 (br, 1H, NH). ^{13}C NMR (101 MHz, CDCl$_3$) δ 147.2 (Ar–C$_q$), 134.9 (q, *J* = 33.0 Hz,*C*CF$_3$), 128.3 (Ar–C$_q$), 128.1 (Ar–C), 125.9 (q, *J* = 273.2 Hz, CF$_3$), 124.6 (Ar–C), 121.9 (Ar–C), 46.0 (SCH$_3$). ^{19}F (377 MHz, CDCl$_3$) δ –63.1 (CF$_3$). Analytical data (NMR and IR) in agreement with those reported in the literature.[9]

Imino(methyl)(16yridine-2-yl)-λ^6-sulfanone (S2e)

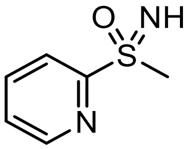

Prepared using general procedure B with sulfide **S1e** (6.9 g, 50 mmol, 1.0 equiv), PhI(Oac)$_2$ (33.8 g, 105 mmol, 2.1 equiv) and NH$_4$Oac (7.7 g, 100 mmol, 2.0 equiv) in MeOH (100 mL). Purification by flash column chromatography (SiO$_2$, 5% EtOH in EtOAc) afforded sulfoximine **S2e** as a colourless oil (3.8 g, 49%). R_f = 0.09 (EtOAc); IR (film)/cm^{-1} 3261, 2925, 1712, 1634, 1575, 1453, 1214, 986, 751, 613, 502; ^{1}H NMR (400 MHz, CDCl$_3$) δ 8.74 (ddd, *J* = 4.8, 1.7, 0.9 Hz, 1H, Ar–H), 8.13 (dt, *J* = 7.8, 1.1 Hz, 1H, Ar–H), 7.95 (td, *J* = 7.7, 1.7 Hz, 1H, Ar–H), 7.52 (ddd, *J* = 7.6, 4.7, 1.1 Hz, 1H, Ar–H), 3.27 (s, 3H, SCH$_3$), 3.17 (br s, 1H, NH); ^{13}C NMR (101 MHz, CDCl$_3$) δ 160.4 (Ar–C$_q$), 150.0 (Ar–C), 138.2 (Ar–C), 126.7 (Ar–C), 121.1 (Ar–C), 42.23 (SCH$_3$). Analytical data (NMR and IR) in agreement with those reported in the literature.[5]

Cyclohexyl(imino)(methyl)-λ^6-sulfanone (S2f)

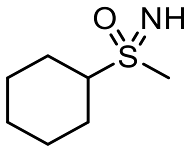

Prepared using general procedure B with sulfide **S1f** (3.9 g, 30 mmol, 1.0 equiv), $PhI(Oac)_2$ (20.3 g, 63 mmol, 2.1 equiv) and NH_4Oac (4.6 g, 60 mmol, 2.0 equiv) in MeOH (60 mL). Purification by flash column chromatography (SiO_2, 5% EtOH in EtOAc) afforded sulfoximine **S2f** as a colourless oil (3.7 g, 76%). R_f = 0.08 (EtOAc); IR (film)/cm^{-1} 3267, 2927, 2854, 2125, 1450, 1191, 1016, 986, 890, 721; ^{1}H NMR (400 MHz, $CDCl_3$) δ 2.90–2.82 (m, 1H, SCH), 2.87 (s, 3H, SCH_3), 2.75 (br s, 1H, NH), 2.24–2.17 (m, 2H, CH_2), 1.96–1.90 (m, 2H, CH_2), 1.75–1.70 (m, 1H, CH*H*), 1.52-1.41 (m, 2H, CH_2), 1.38–1.13 (m, 3H, C*H*$_2$C*H*H); ^{13}C NMR (101 MHz, $CDCl_3$) δ 64.2 (SCH), 39.3 (SCH_3), 25.8 (CH_2), 25.8 (CH_2), 25.2 (2 × CH_2), 25.0 (CH_2). Analytical data (NMR and IR) in agreement with those reported in the literature.[11]

Iminodimethyl-λ^6-sulfanone (S2g)

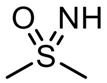

Prepared using a modification of general procedure B with a different work-up procedure with dimethyl sulfide (2.2 mL, 30 mmol, 1.0 equiv), $PhI(Oac)_2$ (20.3 g, 63 mmol, 2.1 equiv) and NH_4Oac (4.7 g, 60 mmol, 2.0 equiv) in MeOH (60 mL). The reaction mixture was concentrated under reduced pressure and diluted with CH_2Cl_2 (50 mL). Solid $NaHCO_3$ (15 g) was added to the reaction mixture and stirred for 15 min, followed by the addition of Na_2SO_4 (15 g) and stirred for 15 min. The solid is removed by filtration and the filtrate was concentrated under reduced pressure. Purification by flash column chromatography (SiO_2, 50% acetone in EtOAc) afforded sulfoximine **S2g** as a pale-yellow oil (2.24 g, 80%). R_f = 0.1 (40% acetone in EtOAc); IR (film)/cm^{-1} 3262, 3013, 2927, 1653, 1421, 1330, 1196, 1032, 976, 919, 756. ^{1}H NMR (400 MHz, $CDCl_3$) δ 3.03 (s, 6H, $S(CH_3)_2$), 2.80 (s, 1H, NH); ^{13}C NMR (101 MHz, $CDCl_3$) δ 45.1 ($S(CH_3)_2$); Analytical data (NMR and IR) in agreement with those reported in the literature.[12]

General Procedure C for TBDPS Protection

TBDPSCl (1.05 equiv) was added to a stirred solution of sulfoximine (1.0 equiv) and imidazole (2.0 equiv) in anhydrous DMF (ca. 0.6 mL/mmol with reference to sulfoximine) at rt. The resulting mixture was stirred at 60 °C for 72 h. Subsequently, the mixture was quenched with H_2O (100 mL) and extracted with Et_2O (3 × 50 mL). The combined organic layer was washed with H_2O (50 mL) and brine (50 mL), dried over Na_2SO_4 and concentrated under reduced pressure. Purification by flash column chromatography (SiO_2) afforded the corresponding TBDPS-protected sulfoximine.[13]

((*tert*-Butyldiphenylsilyl)imino)(methyl)(*p*-tolyl)-λ^6-sulfanone (2a)

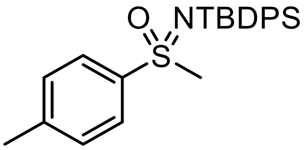

Prepared using general procedure C with sulfoximine **S2a** (5.08 g, 30 mmol, 1.0 equiv), TBDPSCl (8.2 mL, 31.5 mmol, 1.05 equiv) and imidazole (4.08 g, 60 mmol, 2.0 equiv) in anhydrous DMF (18 mL). Purification by flash column chromatography (SiO_2, 5% Et_2O in hexane) afforded TBDPS-protected sulfoximine **2a** as a white solid (9.8 g, 80%). Mp: 96–99 °C; R_f = 0.12 (5% EtOAc in hexane); IR (film)/cm^{-1} 3064, 2957, 2924, 2849, 1426, 1319, 1285, 1148, 1103, 961, 816, 766, 699, 668, 594. ^{1}H NMR (400 MHz, $CDCl_3$) δ 7.84–7.82 (m, 2H, 2 × Ar–H), 7.80–7.78 (m, 2H, 2 × Ar–H), 7.75–7.72 (m, 2H, 2 × Ar–H), 7.39–7.30 (m, 6H, 6 × Ar–H), 7.26–7.24 (m, 2H, 2 × Ar–H), 2.85 (s, 3H, SCH_3), 2.42 (s, 3H, Ar–CH_3), 1.11 (s, 9H, $C(CH_3)_3$). ^{13}C NMR (101 MHz, $CDCl_3$) δ 142.8 (Ar–C_q), 141.8 (Ar–C_q), 136.4 (Ar–C_q), 136.3 (Ar–C_q), 135.6 (2 × Ar–C), 135.6 (2 × Ar–C), 129.4 (2 × Ar–C), 129.0 (Ar–C), 128.9 (Ar–C), 127.4 (2 × Ar–C), 127.4 (2 × Ar–C), 127.0 (2 × Ar–C), 49.1 (SCH_3), 27.1 ($C(\mathit{C}H_3)_3$), 21.4 (Ar–CH_3), 19.4 ($\mathit{C}(CH_3)_3$); HRMS (ESI-TOF) *m/z*: Calcd. for $C_{24}H_{30}NOSSi$ $[M+H]^+$: 408.1817; Found: 408.1807. Analytical data (NMR and IR) in agreement with those reported in the literature.[13]

((*tert*-Butyldiphenylsilyl)imino)(4-methoxyphenyl)(methyl)-λ^6-sulfanone (2b)

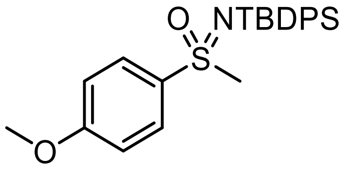

Prepared using general procedure C with sulfoximine **S2b** (5.56 g, 30 mmol, 1.0 equiv), TBDPSCl (8.2 mL, 31.5 mmol, 1.05 equiv) and imidazole (4.08 g, 60 mmol, 2.0 equiv) in anhydrous DMF (18 mL). Purification by flash column chromatography (SiO_2, 10% EtOAc in hexane) afforded TBDPS-protected sulfoximine **2b** as a white solid (10.5 g, 83%). Mp: 97–99 °C; R_f = 0.09 (5% EtOAc in hexane); IR (film)/cm^{-1} 3041, 2953, 2923, 2852, 1592, 1492, 1424, 1280, 1256, 1145, 1096, 1025, 949, 784, 699; ^{1}H NMR (400 MHz, $CDCl_3$) δ 7.88–7.86 (m, 2H, 2 × Ar–H), 7.80–7.77 (m, 2H, 2 × Ar–H), 7.74–7.71 (m, 2H, 2 × Ar–H), 7.38–7.30 (m, 6H, 6 × Ar–H), 6.93–6.91 (m, 2H, 2 × Ar–H), 3.86 (s, 3H, OCH_3), 2.84 (s, 3H, SCH_3) 1.10 (s, 9H, $C(CH_3)_3$); ^{13}C NMR (101 MHz, $CDCl_3$) δ 162.5 (Ar–C_q), 136.5 (2 × Ar–C), 136.4 (Ar–C_q), 135.6 (2 × Ar–C), 135.6 (2 × Ar–C), 129.1 (2 × Ar–C), 129.0 (Ar–C_q), 128.9 (Ar–C_q), 127.4 (2 × Ar–C), 127.4 (2 × Ar–C), 113.9 (2 × Ar–C), 55.6 (OCH_3), 49.4 (SCH_3), 27.1 ($C(\mathit{C}H_3)_3$), 19.4 ($\mathit{C}(CH_3)_3$); HRMS (ESI-TOF) *m/z*: Calcd. for $C_{24}H_{30}NO_2SiS$ $[M+H]^+$: 424.1767, found: 424.1764. Analytical data (NMR and IR) in agreement with those reported in the literature.[13]

((*tert*-Butyldiphenylsilyl)imino)(methyl)(4-(trifluoromethyl)phenyl)-λ^6-sulfanone (2c)

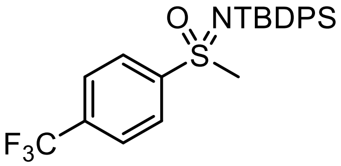

Prepared using general procedure C with sulfoximine **S2c** (4.5 g, 20 mmol, 1.0 equiv), TBDPSCl (5.5 mL, 21 mmol, 1.05 equiv) and imidazole (2.64 g, 40 mmol, 2.0 equiv) in anhydrous DMF (12 mL). Purification by flash column chromatography (SiO_2, 10% Et_2O in hexane) afforded TBDPS-protected sulfoximine **2c** as a white solid (8.4 g, 90%). Mp: 82–85 °C R_f = 0.2 (10% Et_2O in hexane); IR (film)/cm^{-1} 3075, 2959, 2933, 2888, 2853, 1469, 1427, 1401, 1315, 1283, 1156, 1129, 1060, 1013, 962, 843, 773, 698, 601, 501; ^{1}H NMR (400 MHz, $CDCl_3$) δ 8.01 (d, *J* = 8.1 Hz, 2H, 2 × Ar–H), 7.76–7.74 (m, 2H, 2 × Ar–H), 7.69–7.67 (m, 4H, 4 × Ar–H), 7.42–7.28 (m, 6H, 6 × Ar–H), 2.94 (s, 3H, SCH_3), 1.13 (s, 9H, $C(CH_3)_3$); ^{13}C NMR (101 MHz, $CDCl_3$) δ 147.6 (Ar–C_q), 135.7 (Ar–C_q), 135.7 (Ar–C_q), 135.5 (2 × Ar–C), 135.5 (2 × Ar–C), 133.9 (q, *J* = 32.9 Hz, Ar–C_q), 129.2 (Ar–C), 129.1 (Ar–C), 127.5 (2 × Ar–C), 127.4 (2 × Ar–C), 126.0 (Ar–C), 126.0 (Ar–C), 126.0 (Ar–C), 125.9 (Ar–C), 123.3 (q, *J* = 273.7 Hz, CF_3), 48.8 (SCH_3), 27.1 ($C(\mathit{C}H_3)_3$), 19.3 ($C(\mathit{C}H_3)_3$); ^{19}F (377 MHz, $CDCl_3$) δ −63.0 (CF_3). HRMS (ESI-TOF) *m/z*: Calcd. for $C_{24}H_{27}F_3NOSi$ $[M+H]^+$: 462.1529, found: 462.1535.

(S)-(4-Bromophenyl)((*tert*-butyldiphenylsilyl)imino)(methyl)-λ^6-sulfanone ((*S*)-2d)

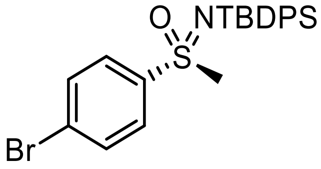

Prepared using general procedure C with (*S*)-methyl *p*-bromophenyl sulfoximine (7.02 g, 30 mmol, 1.0 equiv),[1] TBDPSCl (8.2 mL, 31.5 mmol, 1.05 equiv) and imidazole (4.08 g, 60 mmol, 2.0 equiv) in anhydrous DMF (18 mL). Purification by flash column chromatography (SiO_2, 10% EtOAc in hexane) afforded TBDPS-protected sulfoximine **(*S*)-2d** as a white solid (12.3 g, 86%, 97% *ee*). Mp: 100–102 °C; R_f = 0.10 (5% EtOAc in hexane); IR (film)/cm^{-1} 3068, 2997, 2928, 2912, 2851, 2087, 1605, 1425, 1402, 1320, 1139, 1058, 954, 823, 697, 594; ^{1}H NMR (400 MHz, $CDCl_3$) δ 7.79–7.75 (m, 4H, 4 × Ar–H), 7.72–7.68 (m, 2H, 2 × Ar–H), 7.58–7.55 (m, 2H, 2 × Ar–H), 7.42–7.30 (m, 6H, 6 × Ar–H), 2.89 (s, 3H, SCH_3), 1.12 (s, 9H, $C(CH_3)_3$); ^{13}C NMR (101 MHz, $CDCl_3$) δ 143.4 (Ar–C_q), 136.0 (Ar–C), 135.9 (Ar–C), 135.5 (2 × Ar–C), 135.5 (2 × Ar–C), 132.0 (2 × Ar–C), 129.1 (Ar–C_q), 129.1 (Ar–C_q), 128.6 (2 × Ar–C), 127.5 (2 × Ar–C), 127.4 (2 × Ar–C), 127.2 (Ar–C_q), 49.0 (SCH_3), 27.1 ($C(\mathit{C}H_3)_3$), 19.3 ($\mathit{C}(CH_3)_3$). HRMS (ESI-TOF) *m/z*: Calcd. for $C_{23}H_{27}NOS^{79}BrSi$ $[M+H]^+$: 472.0766, found: 472.0766. $[\alpha]^{21}_D$ = –1 (c 1.0, $CHCl_3$).

Identical reaction conditions were applied to racemic sample methyl *p*-bromophenyl sulfoximine on a 30 mmol scale to afford racemic TBDPS-protected sulfoximine **(*rac*)-2d** as a white solid (12.2 g, 86%). Analytical data (NMR and IR) in agreement with enantioenriched sample.

((*tert*-Butyldiphenylsilyl)imino)(methyl)(pyridin-2-yl)-λ^6-sulfanone (2e)

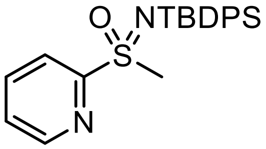

Prepared using general procedure C with sulfoximine **S2e** (3.12 g, 20 mmol, 1.0 equiv), TBDPSCl (5.5 mL, 21 mmol, 1.05 equiv) and imidazole (2.64 g, 40 mmol, 2.0 equiv) in anhydrous DMF (12 mL). Purification by flash column chromatography (SiO_2, 10% EtOAc in hexane) afforded TBDPS-protected sulfoximine **2e** as a colourless oil (6.5 g, 83%). R_f = 0.13 (10% EtOAc in hexane); IR (film)/cm^{-1} 3066, 3044, 2927, 2851, 1578, 1424, 1293, 1159, 1107, 971, 822, 731, 695, 604, 492; ^{1}H NMR (400 MHz, $CDCl_3$) δ 8.59–8.57 (m, 1H, Ar–H), 7.91–7.88 (m, 1H, Ar–H), 7.75–7.67 (m, 5H, 5 × Ar–H), 7.38–7.27 (m, 7H, 7 × Ar–H), 3.12 (s, 3H, SCH_3), 1.09 (s, 9H, $C(CH_3)_3$); ^{13}C NMR (101 MHz, $CDCl_3$) δ 161.3 (Ar–C_q), 149.1 (Ar–C), 137.7 (Ar–C), 136.0 (Ar–C_q), 135.9 (Ar–C_q), 135.6 (2 × Ar–C), 135.5 (2 × Ar–C), 129.0 (2 × Ar–C), 128.9 (2 × Ar–C), 127.4 (2 × Ar–C), 125.9 (Ar–C), 119.9 (Ar–C), 44.2 (SCH_3), 27.1 ($C(\mathit{C}H_3)_3$), 19.3 ($\mathit{C}(CH_3)_3$); HRMS (ESI-TOF) *m/z*: Calcd. for $C_{22}H_{27}N_2OsiS$ $[M+H]^+$: 395.1613, found: 395.1604.

((*tert*-Butyldiphenylsilyl)imino)(cyclohexyl)(methyl)-λ^6-sulfanone (2f)

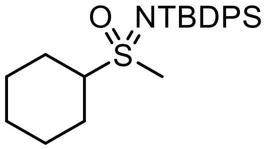

Prepared using general procedure C with sulfoximine **S2f** (3.13 g, 19.4 mmol, 1.0 equiv), TBDPSCl (5.3 mL, 20.4 mmol, 1.05 equiv) and imidazole (2.64 g, 38.8 mmol, 2.0 equiv) in anhydrous DMF (12 mL). Purification by flash column chromatography (SiO_2, 10% EtOAc in hexane) afforded TBDPS-protected sulfoximine **2f** as a colourless oil (6.5 g, 83%). R_f = 0.09 (5% EtOAc in hexane); IR (film)/cm^{-1} 3070, 2930, 2852, 1449, 1286, 1254, 1147, 1104, 955, 740, 698; ^{1}H NMR (400 MHz, $CDCl_3$) δ 7.77–7.72 (m, 4H, 4 × Ar–H), 7.41–7.33 (m, 6H, 6 × Ar–H), 2.68 (tt, *J* = 12.2, 3.5 Hz, 1H, SCH), 2.52 (s, 3H, SCH_3), 2.22 (t, *J* = 13.2 Hz, 2H, 2 × C*H*H), 1.90 (d, *J* = 12.1 Hz, 2H, 2 × CH*H*), 1.71 (d, *J* = 11.5 Hz, 1H, C*H*H), 1.47 (pd, *J* = 12.4, 3.7 Hz, 2H, 2 × C*H*H), 1.28–1.13 (m, 3H, 3 × CH*H*), 1.08 (s, 9H, $C(CH_3)_3$); ^{13}C NMR (101 MHz, $CDCl_3$) δ 136.9 (Ar–C_q), 136.6 (Ar–C_q), 135.7 (2 × Ar–C), 135.6 (2 × Ar–C), 129.0 (2 × Ar–C), 127.4 (4 × Ar–C), 65.6 (SCH), 40.8 (SCH_3), 27.2 ($C(\mathit{C}H_3)_3$), 26.6 (CH_2), 25.5 (CH_2), 25.4 (CH_2), 25.3 (CH_2), 25.3 (CH_2), 19.3 ($\mathit{C}(CH_3)_3$) HRMS (ESI-TOF) *m/z*: Calcd. for $C_{23}H_{34}NOSiS$ $[M+H]^+$: 400.2130, found: 400.2135.

((*tert*-Butyldiphenylsilyl)imino)dimethyl-λ^6-sulfanone (2g)

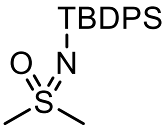

Prepared using general procedure C with sulfoximine **S2g** (2.17 g, 23.3 mmol, 1.0 equiv), TBDPSCl (6.4 mL, 24.5 mmol, 1.05 equiv) and imidazole (3.17 g, 46.6 mmol, 2.0 equiv) in anhydrous DMF (13 mL). Purification by flash column chromatography (SiO_2, 20% EtOAc in hexane) afforded TBDPS-protected sulfoximine **2g** as a colourless oil

(4.34 g, 55%). R_f = 0.3 (30% EtOAc in hexane); IR (film)/cm^{-1} 3067, 3046, 2956, 2929, 2853, 1588, 1425, 1332, 1291, 1157, 1104, 1008, 930, 738, 701; ^{1}H NMR (400 MHz, $CDCl_3$) δ 7.77–7.75 (m, 4H, 4 × Ar–H), 7.43–7.35 (m, 6H, 6 × Ar–H), 2.84 (s, 6H, $S(CH_3)_2$), 1.09 (s, 9H, $C(CH_3)_3$); ^{13}C NMR (101 MHz, $CDCl_3$) δ 136.3 (2 × Ar–C_q), 135.5 (4 × Ar–C), 129.1 (2 × Ar–C), 127.5 (4 × Ar–C), 47.0 ($S(CH_3)_2$), 27.0 ($C(CH_3)_3$), 19.1 ($C(CH_3)_3$); HRMS (ESI-TOF) *m/z*: Calcd. for $C_{18}H_{26}NOSiS$ $[M+H]^+$: 332.1504; Found: 332.1509. Analytical data (NMR and IR) in agreement with those reported in the literature.[14]

General Procedure D for Acetylation

n-BuLi (1.6 M in hexane, 2.1 equiv) was added dropwise to a stirred solution of diisopropylamine (2.1 equiv) in anhydrous THF (1 mL/mmol with reference to TBDPS-protected sulfoximine) at –78 °C. The resulting mixture was stirred at –78 °C for 10 min followed by the dropwise addition of TBDPS-protected sulfoximine (1.0 equiv) in anhydrous THF (1.5 mL/mmol). After stirring at –78 °C for 30 min, EtOAc (1.05 equiv) was added dropwise to the mixture at –78 °C and the resulting mixture was warmed to rt for 3 h. The reaction mixture was quenched with saturated aqueous NH_4Cl solution (50 mL) and extracted with EtOAc (3 × 50 mL). The combined organic layer was washed with brine (50 mL), dried over Na_2SO_4 and concentrated under reduced pressure. Purification by flash column chromatography afforded β-keto sulfoximine.

((*tert*-Butyldiphenylsilyl)imino)(2-oxopropyl)(*p*-tolyl)-λ^6-sulfanone (4a)

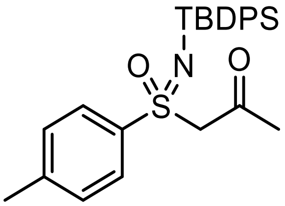

Prepared using general procedure D with TBDPS-protected sulfoximine **2a** (8.2 g, 20 mmol, 1.0 equiv), *n*-BuLi (1.6 M in hexane, 26.3 mL, 42 mmol, 2.1 equiv) and diisopropylamine (5.9 mL, 42 mmol, 2.1 equiv) in anhydrous THF (50 mL). Purification by flash column chromatography (SiO_2, 10% EtOAc in pentane) afforded β-keto sulfoximine **4a** as a colourless oil (7.0 g, 78%). R_f 0.25 (10% EtOAc in pentane). IR (film)/cm^{-1} 3063, 3042, 2925, 2851, 1710 (C=O), 1593, 1317, 1291, 1232, 1160, 1105, 815, 739, 700, 602, 530. ^{1}H NMR (400 MHz, $CDCl_3$) δ 7.78–7.71 (m, 6H, 6 × Ar–H), 7.40–7.31 (m, 6H, 6 × Ar–H), 7.25–7.23 (m, 2H, 2 × Ar–H), 3.94 (d, *J* = 12.5 Hz, 1H, SC*H*H), 3.86 (d, *J* = 12.5 Hz, 1H, SCH*H*), 2.41 (s, 3H, Ar–CH_3), 2.16 (s, 3H, H_3CC=O), 1.11 (s, 9H, $C(CH_3)_3$). ^{13}C NMR (101 MHz, $CDCl_3$) δ 197.6 (C=O), 143.6 (Ar–C_q), 139.4 (Ar–C_q), 135.7 (2 × Ar–C_q), 135.6 (2 × Ar–C), 135.6 (2 × Ar–C), 129.5 (2 × Ar–C), 129.2 (Ar–C), 129.1 (Ar–C), 127.9 (2 × Ar–C), 127.5 (2 × Ar–C), 127.5 (2 × Ar–C), 71.9 (SCH_2), 31.4 (H_3CC=O), 27.1 ($C(CH_3)_3$), 21.5 (Ar-CH_3), 19.4 ($C(CH_3)_3$). HRMS (ESI-TOF) *m/z*: Calcd. for $C_{26}H_{32}NO_2SSi$ $[M+H]^+$: 450.1923; Found: 450.1931.

((*tert*-Butyldiphenylsilyl)imino)(4-methoxyphenyl)(2-oxopropyl)-λ^6-sulfanone (4b)

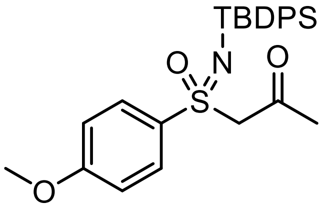

Prepared using general procedure D with TBDPS-protected sulfoximine **2b** (9.3 g, 20 mmol, 1.0 equiv), *n*-BuLi (1.6 M in hexane, 26.3 mL, 42 mmol, 2.1 equiv) and diisopropylamine (5.9 mL, 42 mmol, 2.1 equiv) in anhydrous THF (50 mL). Purification by flash column chromatography (SiO_2, 20% EtOAc in hexane) afforded β-keto sulfoximine **4b** as a colourless oil (5.3 g, 57%). R_f 0.14 (20% EtOAc in pentane). IR (film)/cm^{-1} 3069, 2956, 2930, 2854, 1712 (C=O), 1592, 1494, 1310, 1257, 1152, 1105, 1024, 829, 803, 699, 601. ^{1}H NMR (400 MHz, $CDCl_3$) δ□7.78–7.72 (m, 6H, 6 × Ar–H), 7.42–7.31 (m, 6H, 6 × Ar–H), 6.90 (d, *J* = 8.9 Hz, 2H, 2 × Ar–H), 3.94 (d, *J* = 12.4 Hz, 1H, SC*H*H), 3.86 (d, *J* = 12.4 Hz, 1H, SCH*H*), 3.85 (s, 3H, OCH_3), 2.17 (s, 3H, $COCH_3$), 1.11 (s, 9H, $C(CH_3)_3$). ^{13}C NMR (101 MHz, $CDCl_3$) δ 197.7 (C=O), 163.0 (Ar–C_q), 135.7 (Ar–C_q), 135.7 (Ar–C_q), 135.6 (2 × Ar–C), 135.6 (2 × Ar–C), 134.1 (Ar–C_q), 130.0 (2 × Ar–C), 129.2 (Ar–C), 129.1 (Ar–C), 127.5 (2 × Ar–C), 127.4 (2 × Ar–C), 113.9 (2 × Ar–C), 72.0 (SCH_2), 55.5 (OCH_3), 31.4 (CO*C*H_3), 27.1 (C(*C*H_3)$_3$), 19.4 (*C*(CH_3)$_3$). HRMS (ESI-TOF) *m/z*: Calcd. for $C_{26}H_{32}NO_3SSi$ $[M+H]^+$: 466.1867, found: 466.1866.

((*tert*-Butyldiphenylsilyl)imino)(2-oxopropyl)(4-(trifluoromethyl)phenyl)-λ^6-sulfanone (4c)

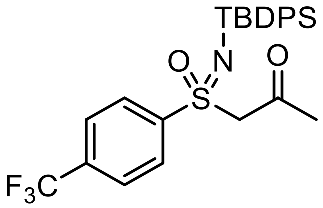

Prepared using general procedure D with TBDPS-protected sulfoximine **2c** (7.71 g, 16.7 mmol, 1.0 equiv), *n*-BuLi (1.6 M in hexane,21.9 mL, 35.1 mmol, 2.1 equiv) and diisopropylamine (4.9 mL, 35.1 mmol, 2.1 equiv) in anhydrous THF (42 mL). Purification by flash column chromatography (5% EtOAc in hexane) afforded β-keto sulfoximine **4c** as a colourless oil (5.56 g, 66%). R_f 0.11 (5% EtOAc in hexane); IR (film)/cm^{-1} 3070, 2956, 2891, 2856, , 1716 (C=O), 1470, 1426, 1397, 1318, 1167, 1131, 1106, 1012, 843, 701, 607, 527; ^{1}H NMR (400 MHz, $CDCl_3$) δ□7.93 (d, *J* = 8.2 Hz, 2H, 2 × Ar–H), 7.74 (dt, *J* = 6.5, 1.6 Hz, 2H, 2 × Ar–H), 7.70–7.66 (m, 4H, 4 × Ar–H), 7.44–7.30 (m, 6H, 6 × Ar–H), 4.04 (d, *J* = 12.9 Hz, 1H, SC*H*H), 3.97 (d, *J* = 12.9 Hz, 1H, SCH*H*), 2.24 (s, 3H, $COCH_3$), 1.14 (s, 9H, $C(CH_3)_3$). ^{13}C NMR (101 MHz, $CDCl_3$) δ□196.9 (C=O), 145.4 (Ar–C_q), 135.5 (2 × Ar–C), 135.5 (2 × Ar–C), 135.1 (Ar–C_q), 135.0 (Ar–C_q), 134.2 (q, *J* = 32.9 Hz, Ar–C_q), 129.4 (Ar–C), 129.3 (Ar–C), 128.4 (2 × Ar–C), 127.6 (2 × Ar–C), 127.5 (2 × Ar–C), 125.9 (q, *J* = 3.7 Hz, 2 × Ar–C), 123.2 (q, *J* = 273.7 Hz, CF_3), 71.0 (SCH_2), 31.6 (CO*C*H_3), 27.0 (C(*C*H_3)$_3$), 19.3 (*C*(CH_3)$_3$); ^{19}F (377 MHz, $CDCl_3$) δ□–63.1 (CF_3). HRMS (ESI-TOF) *m/z*: Calcd. for $C_{26}H_{29}F_3NO_2SSi$ $[M+H]^+$: 504.1635, found: 504.1640.

(*S*)-(4-Bromophenyl)((*tert*-butyldiphenylsilyl)imino)(2-oxopropyl)-λ⁶-sulfanone ((*S*)-4d)

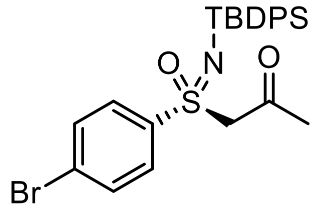

Prepared using general procedure D with TBDPS-protected sulfoximine **(*S*)-2d** (9.5 g, 20 mmol, 1.0 equiv), *n*-BuLi (1.6 M in hexane, 26.3 mL, 42 mmol, 2.1 equiv) and diisopropylamine (5.9 mL, 42 mmol, 2.1 equiv) in anhydrous THF (50 mL). Purification by flash column chromatography (SiO_2, 10% EtOAc in hexane) afforded β-keto sulfoximine **(*S*)-4d** as a colourless oil (8.3 g, 81%, 96% *ee*). R_f 0.11 (10% EtOAc in pentane). IR (film)/cm^{-1} 3068, 2955, 2929, 2854, 1713 (C=O), 1570, 1425, 1308, 1156, 1105, 1065, 1005, 820, 737, 698, 603. ^1H NMR (400 MHz, $CDCl_3$) δ 7.73–7.71 (m, 2H, 2 × Ar–H), 7.69–7.64 (m, 4H, 4 × Ar–H), 7.56–7.53 (m, 2H, 2 × Ar–H), 7.41–7.30 (m, 6H, 6 × Ar–H), 3.96 (d, *J* = 12.7 Hz, 1H, SC*H*H), 3.89 (d, *J* = 12.7 Hz, 1H, SCH*H*), 2.19 (s, 3H, $COCH_3$), 1.10 (s, 9H, $C(CH_3)_3$). ^{13}C NMR (101 MHz, $CDCl_3$) δ 197.3 (C=O), 141.2 (Ar–C_q), 135.6 (2 × Ar–C), 135.5 (2 × Ar–C), 135.3 (Ar–C_q), 135.3 (Ar–C_q), 132.0 (2 × Ar–C), 129.4 (2 × Ar–C), 129.4 (Ar–C), 129.3 (Ar–C), 128.0 (Ar–C_q), 127.6 (2 × Ar–C), 127.5 (2 × Ar–C), 71.4 (SCH_2), 31.6 ($COCH_3$), 27.0 ($C(CH_3)_3$), 19.4 ($C(CH_3)_3$). HRMS (ESI-TOF) *m/z*: Calcd. for $C_{25}H_{29}{}^{79}BrNO_2SSi$ $[M+H]^+$: 514.0866, found: 514.0870. $[\alpha]^{22}_D$ = +33 (c 1.0, $CHCl_3$).

Identical reaction conditions were applied to racemic sample **(*rac*)-2d** on a 20 mmol scale to afford racemic β-keto sulfoximine **(*rac*)-4d** as a colourless oil (8.5 g, 83%). Analytical data (NMR and IR) in agreement with enantioenriched sample.

((*tert*-Butyldiphenylsilyl)imino)(2-oxopropyl)(pyridin-2-yl)-λ⁶-sulfanone (4e)

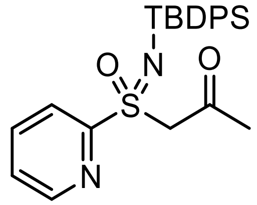

Prepared using general procedure D with TBDPS-protected sulfoximine **2e** (5.92 g, 15 mmol, 1.0 equiv), *n*-BuLi (1.6 M in hexane, 19.7 mL, 31.5 mmol, 2.1 equiv) and diisopropylamine (4.4 mL, 31.5 mmol, 2.1 equiv) in anhydrous THF (37.5 mL). Purification by flash column chromatography (SiO_2, 20% EtOAc in hexane) afforded β-keto sulfoximine **4e** as a colourless oil (4.9 g, 75%). R_f 0.16 (20% EtOAc in hexane); IR (film)/cm^{-1} 3068, 3047, 2955, 2929, 2889, 2854, 1714 (C=O), 1577, 1424, 1307, 1233, 1173, 1107, 992, 820, 771, 741, 700, 604, 495; ^1H NMR (400 MHz, $CDCl_3$) δ 8.59–8.57 (m, 1H, Ar–H), 7.82 (dt, *J* = 7.9, 1.0 Hz, 1H, Ar–H), 7.74–7.68 (m, 5H, 5 × Ar–H), 7.40–7.28 (m, 7H, 7 × Ar–H), 4.40 (d, *J* = 13.5 Hz, 1H, SC*H*H), 4.26 (d, *J* = 13.5 Hz, 1H, SCH*H*), 2.20 (s, 3H, $COCH_3$), 1.09 (s, 9H, $C(CH_3)_3$). ^{13}C NMR (101 MHz, $CDCl_3$) δ 197.4 (C=O), 159.6 (Ar–C_q), 149.3 (Ar–C), 137.7 (Ar–C), 135.6 (2 × Ar–C), 135.6 (2 × Ar–C), 135.4 (Ar–C_q), 135.4 (Ar–C_q), 129.1 (Ar–C), 129.1 (Ar–C), 127.4 (4 × Ar–C), 126.2 (Ar–C), 121.0 (Ar–C), 66.8 (SCH_2), 31.4 ($COCH_3$), 27.0 ($C(CH_3)_3$), 19.4 ($C(CH_3)_3$); HRMS (ESI-TOF) *m/z*: Calcd. for $C_{24}H_{29}N_2O_2SSi$ $[M+H]^+$: 437.1714, found: 437.1712.

((*tert*-Butyldiphenylsilyl)imino)(cyclohexyl)(2-oxopropyl)-λ^6-sulfanone (4f)

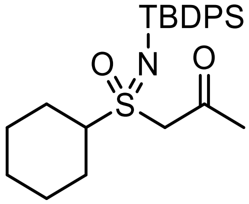

Prepared using general procedure D with TBDPS-protected sulfoximine **2f** (5.60 g, 14 mmol, 1.0 equiv), *n*-BuLi (1.6 M in hexane, 18.4 mL, 29.4 mmol, 2.1 equiv) and diisopropylamine (4.1 mL, 29.4 mmol, 2.1 equiv) in anhydrous THF (35 mL). Purification by flash column chromatography (SiO_2, 10% EtOAc in hexane) afforded β-keto sulfoximine **4f** as a colourless oil (4.4 g, 71%). R_f 0.13 (10% EtOAc in hexane); IR (film)/cm^{-1} 3068, 3047, 2930, 2854, 1710 (C=O), 1469, 1425, 1297, 1260, 1150, 1104, 820, 699, 601; ^{1}H NMR (400 MHz, $CDCl_3$) δ 7.76 (td, *J* = 7.5, 1.9 Hz, 4H, 4 × Ar–H), 7.42–7.35 (m, 6H, 6 × Ar–H), 3.65 (d, *J* = 12.9 Hz, 1H, SC*H*H), 3.51 (d, *J* = 12.9 Hz, 1H, SCH*H*), 3.01 (tt, *J* = 12.0, 3.4 Hz, 1H, SCH), 2.22–2.15 (m, 2H, CH_2), 2.08 (s, 3H, $COCH_3$), 1.93–1.87 (m, 2H, CH_2), 1.71 (dd, *J* = 9.7, 6.2 Hz, 1H, C*H*H), 1.59–1.52 (m, 2H, CH_2), 1.32–1.19 (m, 3H, CH*H* and CH_2), 1.09 (s, 9H, $C(CH_3)_3$); ^{13}C NMR (101 MHz, $CDCl_3$) δ 199.4 (C=O), 136.0 (Ar–C_q), 136.0 (Ar–C_q), 135.7 (2 × Ar–C), 135.6 (2 × Ar–C), 129.3 (Ar–C), 129.2 (Ar–C), 127.5 (2 × Ar–C), 127.5 (2 × Ar–C), 64.0 (SCH_2), 63.4 (SCH), 31.9 (CO*C*H_3), 27.1 (C(*C*H_3)$_3$), 26.8 (CH_2), 25.2 (CH_2), 25.2 (CH_2), 25.1 (CH_2), 24.2 (CH_2), 19.4 (*C*(CH_3)$_3$); HRMS (ESI-TOF) *m/z*: Calcd. for $C_{26}H_{36}NO_2SSi$ $[M+H]^+$: 442.2231, found: 442.2231.

((*tert*-Butyldiphenylsilyl)imino)(methyl)(2-oxopropyl)-λ^6-sulfanone (4g)

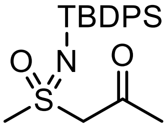

Prepared using general procedure D with TBDPS-protected sulfoximine **2g** (4.34 g, 13 mmol, 1.0 equiv), *n*-BuLi (1.5 M in hexane, 18.2 mL, 27.3 mmol, 2.1 equiv) and diisopropylamine (3.85 mL, 27.3 mmol, 2.1 equiv) in anhydrous THF (19.5 mL). Purification by flash column chromatography (SiO_2, 15% EtOAc in hexane) afforded pure β-keto sulfoximine **4g** as a colourless oil (1.16 g, 24%) and a co-eluted mixture of TBDPS-sulfoximine **2g** and β-keto sulfoximine **4g** (2.35 g, containing 32% β-keto sulfoximine **4g**, 15%, and TBDPS-sulfoximine **2g**, 68%). R_f 0.5 (30% EtOAc in hexane). IR (film)/cm^{-1} 3069, 3047, 2957, 2931, 2856, 1716 (C=O), 1470, 1426, 1301, 1107, 953, 703; ^{1}H NMR (400 MHz, $CDCl_3$) δ 7.74–7.71 (m, 4H, 4 × Ar–H), 7.44–7.36 (m, 6H, 6 × Ar–H), 3.83 (d, *J* = 13.3 Hz, 1H, SC*H*H), 3.79 (d, *J* = 13.3 Hz, 1H, SCH*H*), 2.88 (s, 3H, SCH_3), 2.24 (s, 3H, $COCH_3$), 1.08 (s, 9H, $C(CH_3)_3$); ^{13}C NMR (101 MHz, $CDCl_3$) δ 198.8 (C=O), 135.7 (2 × Ar–C_q), 135.5 (2 × Ar–C), 129.4 (Ar–C), 129.4 (Ar–C), 127.6 (2 × Ar–C), 69.0 (SCH_2), 45.4 (SCH_3), 32.1 (CO*C*H_3), 27.0 (C(*C*H_3)$_3$), 19.2 (*C*(CH_3)$_3$); HRMS (ESI-TOF) *m/z*: Calcd. for $C_{20}H_{28}NO_2SSi$ $[M+H]^+$: 374.1610; Found: 374.1619.

General Procedure E for Diazo Transfer

Et_3N (1.2 equiv) was added dropwise to a stirring solution of β-keto sulfoximine (1.0 equiv) and *p*-ABSA (1.2 equiv) in MeCN (2 mL/mmol with reference to β-keto sulfoximine) at 0 °C. The resulting mixture was warmed to rt. After stirring for 16 h, the reaction mixture was quenched with saturated aqueous NH_4Cl solution (50 mL) and extracted with EtOAc (3 × 50 mL). The combined organic layer was dried over Na_2SO_4 and concentrated under reduced pressure. Purification by flash column chromatography afforded the corresponding α-diazo β-keto sulfoximine.

((*tert*-Butyldiphenylsilyl)imino)(1-diazo-2-oxopropyl)(*p*-tolyl)-λ^6-sulfanone (6a)

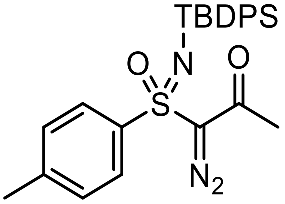

Prepared using general procedure E with β-keto sulfoximine **4a** (4.50 g, 10 mmol, 1.0 equiv), *p*-ABSA (2.88 g, 12 mmol, 1.2 equiv), Et_3N (1.67 mL, 12 mmol, 1.2 equiv) in MeCN (20 mL). Purification by flash column chromatography (SiO_2, 5% EtOAc in pentane) afforded α-diazo β-keto sulfoximine **6a** as a pale-yellow solid (3.91 g, 86%). R_f 0.16 (5% EtOAc in pentane). IR (film)/cm^{-1} 3066, 3045, 2927, 2854, 2086 (C=N=N out-of-phase), 1663 (C=O), 1593, 1467, 1425, 1319 (C=N=N in-phase), 1293, 1278, 1152, 1106, 1014, 815, 700, 663, 545, 496. ^{1}H NMR (400 MHz, $CDCl_3$) δ 7.88–7.86 (m, 2H, 2 × Ar–H), 7.76–7.73 (2 × Ar–H), 7.69–7.67 (2 × Ar–H), 7.41–7.31 (m, 6H, 6 × Ar-H), 7.28–7.26 (m, 2H, 2 × Ar–H), 2.42 (s, 3H, Ar–CH_3), 1.89 (s, 3H, CH_3), 1.13 (s, 9H, $C(CH_3)_3$). ^{13}C NMR (101 MHz, $CDCl_3$) δ 186.3 (C=O) 143.6 (Ar–C_q), 142.7 (Ar–C_q), 135.6 (2 × Ar–C), 135.6 (2 × Ar–C), 134.9 (Ar–C_q), 134.7 (Ar–C_q), 129.5 (2 × Ar–C), 129.3 (2 × Ar–C), 127.5 (2 × Ar–C), 127.5 (2 × Ar–C), 127.2 (2 × Ar–C), 27.1 ($C(\mathit{C}H_3)_3$), 26.6 ($CO\mathit{C}H_3$), 21.5 (Ar–CH_3), 19.4 ($\mathit{C}(CH_3)_3$). HRMS (ESI-TOF) *m/z*: Calcd. for $C_{26}H_{30}N_3O_2SSi$ $[M+H]^+$: 476.1828; Found: 476.1841.

((*tert*-Butyldiphenylsilyl)imino)(1-diazo-2-oxopropyl)(4-methoxyphenyl)-λ^6-sulfanone (6b)

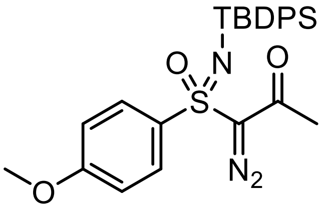

Prepared using general procedure E with β-keto sulfoximine **4b** (4.66 g, 10 mmol, 1.0 equiv), *p*-ABSA (2.88 g, 12 mmol, 1.2 equiv), Et_3N (1.67 mL, 12 mmol, 1.2 equiv) in MeCN (20 mL). Purification by flash column chromatography (SiO_2, 30% CH_2Cl_2 in hexane) afforded α-diazo β-keto sulfoximine **6b** as a pale yellow solid (3.82 g, 82%). R_f 0.37 (30% CH_2Cl_2 in hexane); IR (film)/cm^{-1} 3068, 3015, 2929, 2893, 2856, 2086 (C=N=N out-of-phase), 1658 (C=O), 1588, 1493, 1426, 1357, 1312 (C=N=N in-phase), 1255, 1149, 1107, 1020, 834, 733, 701, 608; ^{1}H NMR (400 MHz, $CDCl_3$) δ 7.94 (d, *J* = 8.9 Hz, 2H, 2 × Ar–H), 7.77–7.75 (m, 2H, 2 × Ar–H), 7.71–7.68 (m, 2H, 2 × Ar–H),

7.44–7.32 (m, 6H, 6 × Ar–H), 6.95 (d, *J* = 8.9 Hz, 2H, 2 × Ar–H), 3.87 (s, 3H, OCH_3), 1.88 (s, 3H, $COCH_3$), 1.15 (s, 9H, $C(CH_3)_3$). ^{13}C NMR (101 MHz, $CDCl_3$) δ 186.2 (C=O), 162.9 (Ar–C_q), 137.3 (Ar–C_q), 135.6 (2 × Ar–C), 135.6 (2 × Ar–C), 134.9 (Ar–C_q), 134.7 (Ar–C_q), 129.5 (2 × Ar–C), 129.3 (2 × Ar–C), 127.5 (2 × Ar–C), 127.4 (2 × Ar–C), 113.9 (2 × Ar–C), 55.6 (OCH_3), 27.1 (C($C$$H_3$)$_3$), 26.6 (CO$C$$H_3$), 19.4 ($C$($CH_3$)$_3$); HRMS (ESI-TOF) *m/z*: Calcd. for $C_{26}H_{30}N_3O_3SSi$ $[M+H]^+$: 492.1772, found: 492.1765.

((*tert*-Butyldiphenylsilyl)imino)(1-diazo-2-oxopropyl)(4-(trifluoromethyl)phenyl)-λ^6-sulfanone (6c)

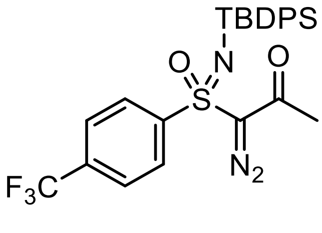

Prepared using general procedure E with β-keto sulfoximine **4c** (5.44 g, 10.8 mmol, 1.0 equiv), *p*-ABSA (3.12 g, 13.0 mmol, 1.2 equiv), Et_3N (1.8 mL, 13.0 mmol, 1.2 equiv) in MeCN (21.6 mL). Purification by flash column chromatography (SiO_2, 5% EtOAc in hexane) afforded α-diazo β-keto sulfoximine **6c** as a pale-yellow solid (4.39 g, 77%). R_f 0.5 (10% EtOAc in hexane); IR (film)/cm^{-1} 3072, 3050, 2958, 2925, 2886, 2854, 2092 (C=N=N out-of-phase), 1666 (C=O), 1319 (C=N=N in-phase), 1279, 1158, 1133, 1060, 1012, 840, 817, 704, 592, 534; ^1H NMR (400 MHz, $CDCl_3$) δ 8.11 (d, *J* = 8.1 Hz, 2H, 2 × Ar–H), 7.74 (td, *J* = 5.7, 3.0 Hz, 4H, 4 × Ar–H), 7.68–7.66 (m, 2H, 2 × Ar–H), 7.45–7.33 (m, 6H, 6 × Ar–H), 1.88 (s, 3H, $COCH_3$), 1.16 (s, 9H, $C(CH_3)_3$); ^{13}C NMR (101 MHz, $CDCl_3$) δ 185.4 (C=O) 148.3 (Ar–C_q), 135.6 (2 × Ar–C), 135.6 (2 × Ar–C), 134.8 (Ar–C_q), 134.5 (Ar–C), 134.2 (Ar–C), 134.2 (q, *J* = 32.3 Hz, $C$$CF_3$), 129.5 (Ar–C), 127.8 (Ar–C), 127.7 (Ar–C_q), 127.6 (2 × Ar–C), 127.5 (2 × Ar–C), 126.1 (Ar–C), 126.0 (Ar–C), 123.2 (q, *J* = 273.7 Hz, CF_3), 27.0 (C($C$$H_3$)$_3$), 26.6 (CO$C$$H_3$), 19.4 ($C$($CH_3$)$_3$); ^{19}F (377 MHz, $CDCl_3$) δ −63.0 (CF_3); HRMS (ESI-TOF) *m/z*: Calcd. for $C_{26}H_{27}F_3N_3O_2SSi$ $[M+H]^+$: 530.1540, found: 530.1545.

(*R*)-(4-Bromophenyl)((*tert*-butyldiphenylsilyl)imino)(1-diazo-2-oxopropyl)-λ^6-sulfanone ((*R*)-6d)

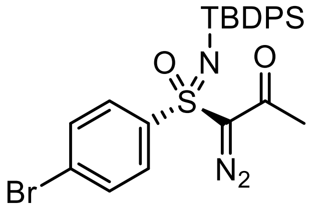

Prepared using general procedure E with β-keto sulfoximine **(*S*)-4d** (7.03 g, 13 mmol, 1.0 equiv), *p*-ABSA (3.75 g, 15.6 mmol, 1.2 equiv), Et_3N (2.17 mL, 15.6 mmol, 1.2 equiv) in MeCN (26 mL). Purification by flash column chromatography (SiO_2, 10% EtOAc in hexane) afforded α-diazo β-keto sulfoximine **(*R*)-6d** as a pale-yellow solid (4.8 g, 68%, 99% *ee*). R_f 0.3 (10% EtOAc in hexane); IR (film)/cm^{-1} 3069, 2954, 2929, 2890, 2855, 2089 (C=N=N out-of-phase), 1663 (C=O), 1569, 1425, 1316 (C=N=N in-phase), 1278, 1257, 1105, 1065, 1005, 819, 698, 599, 539; ^1H NMR (400 MHz, $CDCl_3$) δ 7.84 (d, *J* = 8.6 Hz, 2H, 2 × Ar–H), 7.74–7.71 (m, 2H, 2 × Ar–H), 7.67–7.65 (m, 2H, 2 × Ar–H), 7.61–7.59 (m, 2H, 2 × Ar–H), 7.44–7.32 (m, 6H, 6 × Ar–H), 1.87 (s, 3H, $COCH_3$), 1.14 (s, 9H, $C(CH_3)_3$). ^{13}C NMR (101 MHz, $CDCl_3$) δ 185.6 (C=O), 144.2 (Ar–C_q), 135.6 (2 × Ar–C), 135.6 (2 × Ar–C), 134.6 (Ar–C_q), 134.4 (Ar–C_q),

132.1 (2 × Ar–C), 129.5 (2 × Ar–C), 128.8 (2 × Ar–C), 127.8 (Ar–C_q), 127.6 (2 × Ar–C), 127.5 (2 × Ar–C), 27.1 (C(*C*H_3)$_3$), 26.6 (CO*C*H_3), 19.4 (*C*(CH_3)$_3$). HRMS (ESI-TOF) *m/z*: Calcd. for $C_{25}H_{27}N_3O_2SSi^{79}Br$ $[M+H]^+$: 540.0771, found: 540.0770. $[\alpha]^{22}_D = -33$ (c 1.0, $CHCl_3$).

Identical reaction conditions were applied to racemic sample **(*rac*)-4d** on a 12 mmol scale to afford racemic α-diazo β-keto sulfoximine **(*rac*)-6d** as a pale-yellow solid (5.3 g, 82%). Analytical data (NMR and IR) in agreement with enantioenriched sample.

((*tert*-Butyldiphenylsilyl)imino)(1-diazo-2-oxopropyl)(pyridin-2-yl)-λ^6-sulfanone (6e)

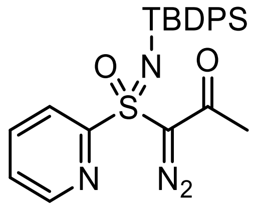

Prepared using general procedure E with β-keto sulfoximine **4e** (4.10 g, 9.4 mmol, 1.0 equiv), *p*-ABSA (2.71 g, 11.3 mmol, 1.2 equiv), Et_3N (1.57 mL, 11.3 mmol, 1.2 equiv) in MeCN (18.8 mL). Purification by flash column chromatography (SiO_2, 25% EtOAc in hexane) afforded α-diazo β-keto sulfoximine **6e** as a pale-yellow solid (1.97 g, 48%). R_f 0.23 (25% EtOAc in hexane); IR (film)/cm^{-1} 3069, 3048, 2955, 2930, 2890, 2856, 2099 (C=N=N out-of-phase), 1656 (C=O), 1424, 1319 (C=N=N in-phase), 1256, 1171, 1108, 820, 736, 701, 602; ^{1}H NMR (400 MHz, $CDCl_3$) δ 8.58 (ddd, *J* = 4.7, 1.7, 0.9 Hz, 1H, Ar–H), 7.94 (d, *J* = 7.9 Hz, 1H, Ar–H), 7.77 (td, *J* = 7.7, 1.8 Hz, 1H, Ar–H), 7.72–7.70 (m, 2H, 2 × Ar–H), 7.65 (dt, *J* = 6.7, 1.5 Hz, 2H, 2 × Ar–H), 7.40–7.29 (m, 7H, 7 × Ar–H), 2.06 (s, 3H, $COCH_3$), 1.13 (s, 9H, C(CH_3)$_3$). ^{13}C NMR (101 MHz, $CDCl_3$) δ 187.0 (C=O), 161.2 (Ar–C_q), 149.7 (Ar–C), 137.8 (Ar–C), 135.6 (2 × Ar–C), 135.5 (2 × Ar–C), 134.6 (Ar–C_q), 134.6 (Ar–C_q), 129.3 (2 × Ar–C), 127.5 (2 × Ar–C), 127.4 (2 × Ar–C), 126.4 (Ar–C), 121.1 (Ar–C), 27.0 (C(*C*H_3)$_3$), 26.9 (CO*C*H_3), 19.4 (*C*(CH_3)$_3$); HRMS (ESI-TOF) *m/z*: Calcd. for $C_{24}H_{27}N_4O_2SSi$ $[M+H]^+$: 463.1618, found: 463.1627.

((*tert*-Butyldiphenylsilyl)imino)(cyclohexyl)(1-diazo-2-oxopropyl)-λ^6-sulfanone (6f)

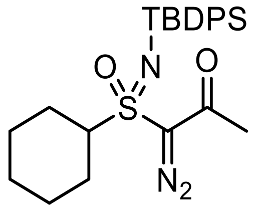

Prepared using general procedure E with β-keto sulfoximine **4f** (4.41 g, 10 mmol, 1.0 equiv), *p*-ABSA (2.88 g, 12 mmol, 1.2 equiv), Et_3N (1.67 mL, 12 mmol, 1.2 equiv) in MeCN (20 mL). Purification by flash column chromatography (SiO_2, 10% EtOAc in hexane) afforded α-diazo β-keto sulfoximine **6f** as a pale-yellow solid (3.8 g, 81%). R_f 0.18 (10% EtOAc in hexane); IR (film)/cm^{-1} 3068, 3046, 2930, 2853, 2089 (C=N=N out-of-phase), 1660 (C=O), 1469, 1450, 1425, 1356, 1308 (C=N=N in-phase), 1286, 1257, 1148, 1104, 999, 819, 699, 599, 485; ^{1}H NMR (400 MHz, $CDCl_3$) δ 7.76–7.74 (m, 2H, 2 × Ar–H), 7.69–7.67 (m, 2H, 2 × Ar–H), 7.42–7.32 (m, 6H, 6 × Ar–H), 3.25

(tt, *J* = 12.3, 3.5 Hz, 1H, SCH), 2.38–2.34 (m, 1H, SCHC*H*H), 2.09–2.04 (m, 1H, SCHC*H*H), 1.99–1.87 (m, 2H, 2 × SCHCH*H*), 1.95 (s, 3H, $COCH_3$), 1.75–1.70 (m, 1H, CH), 1.66–1.45 (m, 2H, 2 × CH), 1.36-1.16 (m, 3H, 3 × CH), 1.10 (s, 9H, $C(CH_3)_3$); ^{13}C NMR (101 MHz, $CDCl_3$) δ□186.7 (C=O), 135.7 (2 × Ar–C), 135.6 (2 × Ar–C), 135.2 (Ar–C_q), 134.8 (Ar–C_q), 129.3 (2 × Ar–C), 127.5 (2 × Ar–C), 127.4 (2 × Ar–C), 81.6 (C=N=N), 68.4 (SCH), 27.1 ($C(CH_3)_3$), 26.7($COCH_3$), 26.2 ($SCH(CH_2)_2CH_2$), 25.3 and 25.3 ($SCH(CH_2)_2$), 25.2 and 25.1 (2 × $SCHCH_2CH_2$) 19.3 ($C(CH_3)_3$). HRMS (ESI-TOF) *m/z*: Calcd. for $C_{25}H_{34}N_3O_2SSi$ $[M+H]^+$: 468.2136, found: 468.2137.

((*tert*-Butyldiphenylsilyl)imino)(1-diazo-2-oxopropyl)(methyl)-λ⁶-sulfanone (6g)

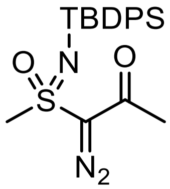

Prepared using general procedure E with β-keto sulfoximine **4g** (1.16 g, 3.1 mmol, 1.0 equiv), *p*-ABSA (890 mg, 3.7 mmol, 1.2 equiv), Et_3N (515 □L, 3.7 mmol, 1.2 equiv) in MeCN (6.2 mL). Purification by flash column chromatography (SiO_2, 20% EtOAc in hexane) afforded α-diazo β-keto sulfoximine **6g** as a pale-yellow oil (1.08 g, 87%). R_f 0.2 (20% EtOAc in hexane); IR (film)/cm^{-1} 3070, 2957, 2931, 2857, 2097 (C=N=N out-of-phase), 1662 (C=O), 1469, 1329 (C=N=N in-phase), 1285, 1159, 1108, 960, 704; ^1H NMR (400 MHz, $CDCl_3$) δ 7.76–7.72 (m, 2H, 2 × Ar–H), 7.69–7.66 (m, 2H, 2 × Ar–H), 7.45–7.33 (m, 6H, 6 × Ar–H), 3.25 (s, 3H, SCH_3), 1.97 (s, 3H, $COCH_3$), 1.09 (s, 9H, $(C(CH_3)_3)$; ^{13}C NMR (101 MHz, $CDCl_3$) δ□186.0 (C=O), 135.6 (2 × Ar–C), 135.5 (2 × Ar–C), 135.0 (Ar–C_q), 134.6 (Ar–C_q), 129.4 (2 × Ar–C), 127.5 (4 × Ar–C), 49.8 (SCH_3), 27.0 ($C(CH_3)_3$), 26.6 ($COCH_3$), 19.2 ($C(CH_3)_3$); HRMS (ESI-TOF) *m/z*: Calcd. for $C_{20}H_{26}N_3O_2SSi$ $[M+H]^+$: 400.1515, found: 400.1507.

Synthesis of Boc-protected Sulfoximine Diazo Compounds

***tert*-Butyl (oxo(2-oxopropyl)(*p*-tolyl)-λ⁶-sulfaneylidene)carbamate (3a)**

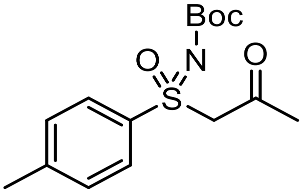

n-Butyllithium (1.54 M in hexane, 6.8 mL, 10.5 mmol, 2.1 equiv) was added dropwise to a stirred solution of tetramethylpiperidine (1.95 mL, 11.5 mmol, 2.3 equiv) in anhydrous THF (5.0 mL) at –78 °C. The mixture was stirred for 10 min followed by the dropwise addition of a solution of Boc-sulfoxmine **1a** (1.35 g, 5.0 mmol, 1.0 equiv) in anhydrous THF (8.0 mL). After stirring for 30 min at –78 °C, ethyl acetate (537 mL, 5.5 mmol, 1.1 equiv) was added dropwise and the mixture was warmed to rt for 3 h. The reaction mixture was quenched with saturated aqueous ammonium chloride (5 mL) and extracted with CH_2Cl_2 (3 × 25 mL). The combined organic layer was dried over Na_2SO_4 and the solvent was removed under reduced pressure. Purification by flash column chromatography (SiO_2, 30% EtOAc in pentane) afforded ketone **3a** as a colourless oil (1.37 g, 88%). R_f 0.24 (30% EtOAc in pentane).

IR (film)/cm^{-1} 2974, 2915, 1725 (C=O), 1660 (C=O), 1592, 1361, 1268, 1248, 1222, 1146, 1116, 1080, 1012, 858, 814, 788, 649, 512. ^{1}H NMR (400 MHz, $CDCl_3$) δ 7.83 (d, *J* = 8.4 Hz, 2H, 2 × Ar–H), 7.38 (d, *J* = 8.4 Hz, 2H, 2 × Ar–H), 4.67 (d, *J* = 13.6 Hz, 1H, SC*H*H), 4.39 (d, *J* = 13.6 Hz, 1H, SCH*H*), 2.46 (s, 3H, Ar–CH_3), 2.38 (s, 3H, C=OCH_3), 1.44 (s, 9H, $C(CH_3)_3$). ^{13}C NMR (101 MHz, $CDCl_3$) δ 195.7 (C=O), 157.9 (NCO_2), 145.5 (Ar–C_q), 133.3 (Ar–C_q), 130.1 (2 × Ar–C), 128.3 (2 × Ar–C), 81.0 (*C*$(CH_3)_3$), 65.7 (SCH_2), 31.6 (*C*H_3CO), 28.0 (C(*C*H_3)$_3$), 21.7 (Ar–CH_3). HRMS (ESI-TOF) *m/z*: Calcd. for $C_{15}H_{22}NO_4S$ $[M+H]^+$: 312.1270; Found: 312.1270.

tert-Butyl ((1-diazo-2-oxopropyl)(oxo)(*p*-tolyl)-λ^6-sulfaneylidene)carbamate (5a)

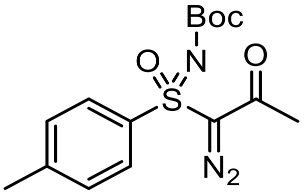

TMEDA (791 mL, 5.28 mmol, 1.2 equiv) was added dropwise to a stirred solution of β-keto sulfoximine **3a** (1.37 g, 4.4 mmol, 1.0 equiv) and NfN_3 (1.0 mL, 5.28 mmol, 1.2 equiv) in anhydrous MeCN (8.8 mL) at 0 °C. The reaction mixture was slowly warmed to rt and stirred for 30 min. The reaction mixture was quenched with saturated aqueous $NaHCO_3$ (5 mL) and extracted with CH_2Cl_2 (3 × 25 mL). The combined organic layer was dried over Na_2SO_4 and the solvent was removed under reduced pressure. Purification by flash column chromatography (SiO_2, 15% EtOAc in pentane) afforded diazo **5a** as a pale-yellow oil (1.19 mg, 80%). R_f 0.17 (15% EtOAc in pentane). IR (film)/cm^{-1} 2974, 2927, 2103 (C=N=N out-of-phase), 1706 (C=O), 1665 (C=O), 1592, 1392 (C=N=N in-phase), 1364, 1265, 1237, 1193, 1139, 1083, 1034, 896, 858, 812, 786, 564, 495. ^{1}H NMR (400 MHz, $CDCl_3$) δ ^{1}H NMR (400 MHz, $CDCl_3$) δ 8.00–7.98 (m, 2H, 2 × Ar–H), 7.39–7.37 (m, 2H, 2 × Ar–H), 2.46 (s, 3H, Ar–CH_3), 2.21 (s, 3H, $COCH_3$), 1.46 (s, 9H, $C(CH_3)_3$). ^{13}C NMR (101 MHz, $CDCl_3$) δ 185.2 (C=O), 156.4 (COO), 145.6 (Ar–C_q), 135.9 (Ar–C_q), 130.0 (2 × Ar–C), 128.2 (2 × Ar–C), 81.6 (*C*$(CH_3)_3$), 28.0 (C(*C*H_3)$_3$), 26.9 (H_3*C*C=O), 21.7 (Ar–CH_3). HRMS (ESI-TOF) *m/z*: Calcd. for $C_{15}H_{20}N_3O_4S$ $[M+H]^+$: 338.1175; Found: 338.1169.

General Procedure F for De-acetylation

K_2CO_3 (10 mol%) was added to a stirring solution of α-diazo β-keto sulfoximine (1.0 equiv) in anhydrous MeOH (15 mL/mmol with reference to α-diazo β-keto sulfoximine) at rt. The reaction was monitored by TLC. After the full consumption of starting material, brine (20 mL) was added to the reaction mixture followed by the extraction with CH_2Cl_2 (3 × 10 mL). The combined organic layer was washed with brine (2 × 10 mL), dried over Na_2SO_4 and concentrated under reduced pressure to afford the corresponding α-diazo sulfoximine which could be used without further purification. The prepared α-diazo sulfoximines were stored at –20 °C as a slow decomposition was observed at rt.

((*tert*-Butyldiphenylsilyl)imino)(diazomethyl)(*p*-tolyl)-λ⁶-sulfanone (8a)

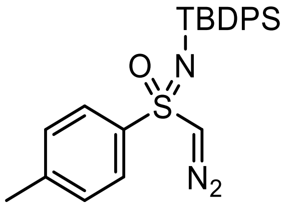

Prepared using general procedure F with α-diazo β-keto sulfoximine **6a** (476 mg, 1.0 mmol, 1.0 equiv) and K_2CO_3 (13.8 mg, 0.1 mmol, 10 mol%) in MeOH (15 mL). α-Diazo sulfoximine **8a** was achieved as a yellow oil (346 mg, 80%) which was used without further purification. R_f 0.30 (5% EtOAc in hexane); IR (film)/cm^{-1} 3064, 3043, 2851, 2083 (C=N=N out-of-phase), 1591, 1466, 1319 (C=N=N in-phase), 1293, 1162, 1105, 1009, 814, 699, 496; ^{1}H NMR (400 MHz, $CDCl_3$) δ 7.82–7.74 (m, 6H, 6 × Ar–H), 7.42–7.33 (m, 6H, 6 × Ar–H), 7.26 (d, *J* = 8.0 Hz, 2H, 2 × Ar–H), 4.91 (s, 1H, CHN_2), 2.42 (s, 3H, Ar–CH_3), 1.13 (s, 9H, $C(CH_3)_3$); ^{13}C NMR (101 MHz, $CDCl_3$) δ 145.3 (Ar–C_q), 142.6 (Ar–C_q), 135.7 (2 × Ar–C), 135.6 (2 × Ar–C), 135.5 (2 × Ar–C_q), 129.5 (2 × Ar–C), 129.2 (Ar–C), 129.1 (Ar–C), 127.4 (4 × Ar–C), 126.0 (2 × Ar–C), 60.5 (CHN_2), 27.0 ($C(\mathit{C}H_3)_3$), 21.4 (Ar–CH_3), 19.4 ($\mathit{C}(CH_3)_3$); HRMS (ESI-TOF) *m/z*: Calcd. for $C_{24}H_{28}N_3OSSi$ $[M+H]^+$: 434.1722, found: 434.1740.

((*tert*-Butyldiphenylsilyl)imino)(diazomethyl)(4-methoxyphenyl)-λ⁶-sulfanone (8b)

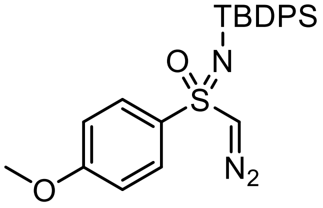

Prepared using general procedure F with α-diazo β-keto sulfoximine **6b** (494 mg, 1.0 mmol, 1.0 equiv) and K_2CO_3 (13.8 mg, 0.1 mmol, 10 mol%) in MeOH (15 mL). α-Diazo sulfoximine **8b** was achieved as a yellow oil (346 mg, 77%) which was used without further purification. R_f 0.10 (5% EtOAc in pentane); IR (film)/cm^{-1} 3070, 2956, 2930, 2891, 2855, 2085 (C=N=N out-of-phase), 1592, 1493, 1311 (C=N=N in-phase), 1254, 1158, 1139, 1026, 907, 730, 533; ^{1}H NMR (400 MHz, $CDCl_3$) δ 7.86–7.83 (m, 2H, 2 × Ar–H), 7.78–7.72 (m, 4H, 4 × Ar–H), 7.41–7.32 (m, 6H, 6 × Ar–H), 6.93–6.90 (m, 2H, 2 × Ar–H), 4.90 (s, 1H, CHN_2), 3.86 (s, 3H, OCH_3), 1.12 (s, 9H, $C(CH_3)_3$). ^{13}C NMR (101 MHz, $CDCl_3$) δ 162.3 (Ar–C_q), 140.2 (Ar–C_q), 135.7 (Ar–C_q), 135.6 (Ar–C_q), 135.5 (4 × Ar–C), 129.2 (Ar–C), 129.1 (Ar–C), 128.1 (2 × Ar–C), 127.4 (4 × Ar–C), 113.9 (2 × Ar–C), 60.7 (CHN_2), 55.6 (OCH_3), 27.1 ($C(\mathit{C}H_3)_3$), 19.4 ($\mathit{C}(CH_3)_3$). HRMS (ESI-TOF) *m/z*: Calcd. for $C_{24}H_{28}N_3O_2SiS$ $[M+H]^+$: 450.1672, found: 450.1667.

((*tert*-Butyldiphenylsilyl)imino)(diazomethyl)(4-(trifluoromethyl)phenyl)-λ^6-sulfanone (8c)

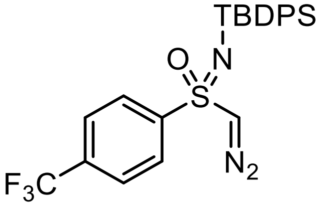

Prepared using general procedure F with α-diazo β-keto sulfoximine **6c** (530 mg, 1.0 mmol, 1.0 equiv) and K_2CO_3 (13.8 mg, 0.1 mmol, 10 mol%) in MeOH (15 mL). Further purification by flash column chromatography (SiO_2, 5% EtOAc in pentane) afforded α-diazo sulfoximine **8c** as a pale-yellow solid (316 mg, 65%). R_f 0.32 (5% EtOAc in pentane); IR (film)/cm^{-1} 3071, 2931, 2890, 2956, 2857, 2092 (C=N=N out-of-phase), 1468, 1426, 1318 (C=N=N in-phase), 1166, 1131, 1059, 1012, 732, 698, 606; ^{1}H NMR (500 MHz, $CDCl_3$) δ 7.99–7.96 (m, 2H, 2 × Ar–H), 7.74–7.72 (m, 2H, 2 × Ar–H), 7.71–7.68 (m, 4H, 4 × Ar–H), 7.42–7.38 (m, 2H, 2 × Ar–H), 7.37–7.32 (m, 4H, 4 × Ar–H), 4.98 (s, 1H, CHN_2), 1.13 (s, 9H, $C(CH_3)_3$); ^{13}C NMR (125 MHz, $CDCl_3$) δ 150.9 (C=O), 135.5 (2 × Ar–C), 135.5 (2 × Ar–C), 135.1 (Ar–C_q), 135.0 (Ar–C_q), 133.5 (q, *J* = 33.0 Hz, *C*CF_3), 129.4 (Ar–C), 129.4 (Ar–C), 127.6 (2 × Ar–C), 127.5 (2 × Ar–C), 126.4 (2 × Ar–C), 126.2 (Ar–C), 126.1 (Ar–C), 123.3 (q, *J* = 273.0 Hz, CF_3), 60.2 (CHN_2), 27.0 (C(*C*H_3)$_3$), 19.4 (*C*(CH_3)$_3$); HRMS (ESI-TOF) *m/z*: Calcd. for $C_{24}H_{25}N_3OSiSF_3$ [M+H]$^+$: 488.1440, found: 488.1424.

Large scale reaction: K_2CO_3 (41 mg, 0.3 mmol, 10 mol%) was added to a solution of α-diazo β-keto sulfoximine **6c** (1.59 g, 3.0 mmol, 1 euiqv.) in MeOH (45 mL) and the resulting mixture was stirred for 30 min at room temperature. Brine (60 mL) was added and the phases were separated. The aqueous layer was extracted with CH_2Cl_2 (4 x 60 mL) and the combined organic layers were dried over Na_2SO_4, filtered and concentrated to give the crude product as a yellow oil. Purification by flash column chromatography (SiO_2, 5% EtOAc in pentane) afforded α-diazo sulfoximine **8c** as a pale-yellow solid (837 mg, 1.72 mmol, 57%).

(*R*)-(4-Bromophenyl)((*tert*-butyldiphenylsilyl)imino)(diazomethyl)-λ^6-sulfanone ((*R*)-8d)

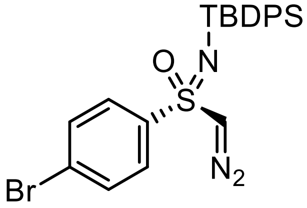

Prepared using general procedure F with α-diazo β-keto sulfoximine **((*R*)-6d)** (540 mg, 1.0 mmol, 1.0 equiv) and K_2CO_3 (13.8 mg, 0.1 mmol, 10 mol%) in MeOH (15 mL). Further purification by flash column chromatography (SiO_2, 3% EtOAc in pentane) afforded α-diazo sulfoximine **((*R*)-8d)** as a pale-yellow solid (398 mg, 80%, 96% *ee*). R_f 0.42 (10% EtOAc in pentane); IR (film)/cm^{-1} 3069, 2955, 2929, 2854, 2088 (C=N=N out-of-phase), 1570, 1468, 1425, 1316 (C=N=N in-phase), 1163, 1106, 1065, 818, 739, 699, 603; ^{1}H NMR (400 MHz, $CDCl_3$) δ 7.79–7.73 (m, 6H, 6 × Ar–H), 7.59–7.57 (m, 2H, 2 × Ar–H), 7.44–7.35 (m, 6H, 6× Ar–H), 4.97 (s, 1H, CHN_2), 1.16 (s, 9H, $C(CH_3)_3$); ^{13}C NMR (101 MHz, $CDCl_3$) δ 146.9 (Ar–C_q), 135.5 (2 × Ar–C), 135.4 (2 × Ar–C), 135.2 (Ar–C_q), 135.1 (Ar–C_q), 132.1 (4 × Ar–C), 129.3 (Ar–C), 129.3 (Ar–C), 127.5 (4 × Ar–C), 126.7 (Ar–C_q), 60.3 (CHN_2), 27.0 (C(*C*H_3)$_3$), 19.4 (*C*(CH_3)$_3$); HRMS (ESI-TOF) *m/z*: Calcd. for $C_{23}H_{25}N_3OsiS^{79}Br$ [M+H]$^+$: 498.0671, found: 498.0680. $[\alpha]^{22}_D$ = –34 (c 1.0, $CHCl_3$).

Identical reaction conditions were applied to racemic sample **((*rac*)-6d)** on a 1.0 mmol scale to afford racemic α-diazo sulfoximine **((*rac*)-8d)** as a pale-yellow solid (412.5 mg, 83%). Analytical data (NMR and IR) in agreement with enantioenriched sample.

((*tert*-Butyldiphenylsilyl)imino)(diazomethyl)(pyridin-2-yl)-λ^6-sulfanone (8e)

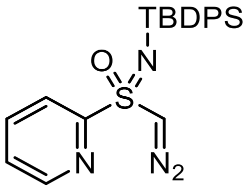

Prepared using general procedure F with α-diazo β-keto sulfoximine **6e** (463 mg, 1.0 mmol, 1.0 equiv) and K_2CO_3 (13.8 mg, 0.1 mmol, 10 mol%) in MeOH (15 mL). α-Diazo sulfoximine **8e** was achieved as a yellow oil (302 mg, 72%) which was used without further purification. R_f 0.32 (25% EtOAc in pentane; IR (film)/cm^{-1} 3069, 3048, 2955, 2929, 2854, 2093 (C=N=N out-of-phase), 1573, 1450, 1423, 1318 (C=N=N in-phase), 1173, 1107, 992, 818, 735, 698, 602; ^{1}H NMR (400 MHz, $CDCl_3$) δ 8.61 (d, *J* = 4.6 Hz, 1H, Ar–H), 7.88 (d, *J* = 7.9 Hz, 1H, Ar–H), 7.78–7.71 (m, 5H, 5 × Ar–H), 7.39–7.31 (m, 7H, 7 × Ar–H), 5.08 (s, 1H, CHN_2), 1.12 (s, 9H, $C(CH_3)_3$); ^{13}C NMR (101 MHz, $CDCl_3$) δ 163.3 (Ar–C_q), 149.6 (Ar–C), 137.7 (Ar–C), 135.5 (2 × Ar–C), 135.6 (2 × Ar–C), 135.4 (Ar–C_q), 135.2 (Ar–C_q), 129.1 (2 × Ar–C), 127.4 (4 × Ar–C), 125.9 (Ar–C), 119.5 (Ar–C), 57.5 (CHN_2), 27.0 ($C(\mathit{C}H_3)_3$), 19.4 ($\mathit{C}(CH_3)_3$); HRMS (ESI-TOF) *m/z*: Calcd. for $C_{22}H_{23}N_3O_4Si$ $[M+H]^+$: 421.1458, found: 421.1462.

((*tert*-Butyldiphenylsilyl)imino)(cyclohexyl)(diazomethyl)-λ^6-sulfanone (8f)

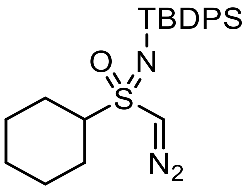

Prepared using general procedure F with α-diazo β-keto sulfoximine **6f** (468 mg, 1.0 mmol, 1.0 equiv) and K_2CO_3 (13.8 mg, 0.1 mmol, 10 mol%) in MeOH (15 mL). Further purification by flash column chromatography (SiO_2, 3% EtOAc in pentane) afforded α-diazo sulfoximine **8f** as a pale-yellow solid (319 mg, 75%). R_f 0.6 (10% EtOAc in pentane); IR (film)/cm^{-1} 3069, 2930, 2855, 2088 (C=N=N out-of-phase), 1469, 1450, 1307 (C=N=N in-phase), 1260, 1141, 1104, 1000, 908, 820, 730, 698, 604; ^{1}H NMR (500 MHz, $CDCl_3$) δ 7.77–7.73 (m, 4H, 4 × Ar–H), 7.41–7.35 (m, 6H, 6 × Ar–H), 4.58 (s, 1H, CHN_2), 2.88 (tt, *J* = 12.1, 3.4 Hz, 1H, SCH), 2.31–2.23 (m, 2H, 2 × C*H*H), 1.93–1.89 (m, 2H, 2 × CH*H*), 1.71 (dddt, *J* = 11.6, 5.3, 3.4, 1.8 Hz, 1H, C*H*H), 1.53–1.43 (m, 2H, 2 × C*H*H), 1.32-1.12 (m, 3H, 3 × CH*H*), 1.10 (s, 9H, $C(CH_3)_3$); ^{13}C NMR (125 MHz, $CDCl_3$) δ 136.0 (Ar–C_q), 135.8 (Ar–C_q), 135.6 (2 × Ar–C), 135.5 (2 × Ar–C), 129.1 (Ar–C), 129.1 (Ar–C), 127.4 (4 × Ar–C), 70.3 (CHN_2), 53.0 (SCH), 27.1 ($C(\mathit{C}H_3)_3$), 26.7 (CH_2), 26.2 (CH_2), 25.3 (CH_2), 25.3 (CH_2), 19.4 ($\mathit{C}(CH_3)_3$); HRMS (ESI-TOF) *m/z*: Calcd. for $C_{23}H_{34}N_4OSiS$ $[M+NH_3]^+$: 442.2223, found: 442.2220.

((*tert*-Butyldiphenylsilyl)imino)(diazomethyl)(methyl)-λ^6-sulfanone (8g)

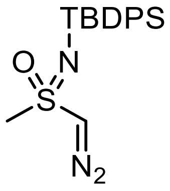

Prepared using general procedure F with α-diazo β-keto sulfoximine **6g** (397 mg, 1.0 mmol, 1.0 equiv) and K_2CO_3 (13.8 mg, 0.1 mmol, 10 mol%) in MeOH (15 mL). α-Diazo sulfoximine **8g** was achieved as a yellow oil (287 mg, 80%) which was used without further purification. R_f 0.35 (20% EtOAc in hexane); IR (film)/cm^{-1} 3070, 2930, 2855, 2092 (C=N=N out-of-phase), 1469, 1328 (C=N=N in-phase), 1305, 1165, 958, 701; ^{1}H NMR (400 MHz, $CDCl_3$) δ 7.76–7.72 (m, 4H, 4 × Ar–H), 7.43–7.35 (m, 6H, 6 × Ar–H), 4.94 (s, 1H, CHN_2), 3.10 (s, 3H, SCH_3), 1.09 (s, 9H, $(C(CH_3)_3)$; ^{13}C NMR (101 MHz, $CDCl_3$) δ 135.5 (2 × Ar–C_q), 135.5 (4 × Ar–C), 129.3 (2 × Ar–C), 127.5 (4 × Ar–C), 58.7 (CHN_2), 52.0 (SCH_3), 26.9 ($C(\mathit{C}H_3)_3$), 19.2 ($\mathit{C}(CH_3)_3$); HRMS (ESI-TOF) *m/z*: Calcd. for $C_{24}H_{28}N_3OSSi$ $[M+H]^+$: 434.1722, found: 434.1740.

Preparation of Alkyne Starting Materials

***N*-Phenylpropiolamide (S11a)**

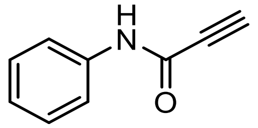

n-BuLi (1.5 M in hexane, 4.3 mL, 6.5 mmol, 1.3 equiv) was added dropwise to a stirred solution of propiolic acid (369 μL, 6.0 mmol, 1.2 equiv) in anhydrous THF (15 mL) at –78 °C. The resulting mixture was slowly warmed to rt for 45 min followed by dropwise addition of trimethylacetyl chloride (740 μL, 6.0 mmol, 1.2 equiv) at 0 °C. The mixture was slowly warmed to rt for 2 h followed by dropwise addition of aniline (456 μL, 5.0 mmol, 5.0 equiv) in anhydrous THF (10 mL). The resulting mixture was stirred overnight at rt followed by quenching with saturated aqueous NH_4Cl solution (10 mL) and extracted with EtOAc (3 × 5 mL). The combined organic layer was washed with brine (10 mL), dried over Na_2SO_4 and concentrated under reduced pressure. Purification by flash column chromatography (SiO_2, 15% EtOAc in pentane) afforded alkynyl amide **S11a** as a pale-orange solid (0.37 g, 51%). R_f 0.5 (40% EtOAc in pentane); IR (film)/cm^{-1} 3266, 3125, 3019, 2107, 1638 (C=O), 1592, 1525, 1318, 1261, 1171, 1077, 760, 724, 691; ^{1}H NMR (400 MHz, $CDCl_3$) δ 7.79 (s, 1H, NH), 7.54 (d, *J* = 7.9 Hz, 2H, 2 × Ar–H), 7.35 (t, *J* = 7.8 Hz, 2H, 2 × Ar–H), 7.16 (t, *J* = 7.4 Hz, 1H, Ar–H), 2.93 (s, 1H, CCH); ^{13}C NMR (101 MHz, $CDCl_3$) δ 149.7 (C=O), 136.9 (Ar–C_q), 129.1 (2 × Ar–C), 125.2 (Ar–C), 120.0 (2 × Ar–C), 77.6 (*C*CH), 74.1 (C*C*H). Analytical data (NMR and IR) in agreement with those reported in the literature.[15]

1-(Piperidin-1-yl)prop-2-yn-1-one (S12a)[16]

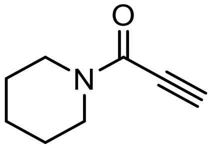

N,*N*'-Dicyclohexylcarbodiimide (4.55 g, 22 mmol, 1.1 equiv) was added to a stirred solution of propiolic acid (1.24 mL, 20 mmol, 1.0 equiv) and piperidine (2.17 mL, 22 mmol, 1.1 equiv) in anhydrous CH_2Cl_2 (20 mL, 1.0 M) at 0 °C. The resulting mixture was slowly warmed to rt for 18 h. The reaction mixture was concentrated under reduced pressure, diluted with EtOAc (20 mL) and filtered. H_2O (20 mL) was added to the filtrate, separated, and the aqueous layer was further extracted with EtOAc (2 × 10 mL). The combined organic layer was dried over Na_2SO_4, filtered and concentrated under reduced pressure. Purification by flash column chromatography (SiO_2, 25% EtOAc in hexane) afforded the alkynyl amide **S12a** as a pale-red liquid (1.35 g, 49%). R_f 0.21 (25% EtOAc in hexane); IR (film)/cm^{-1} 3204, 2937, 2857, 2099, 1613 (C=O), 1432, 1259, 1228, 1140, 1013, 954, 896, 853, 734, 600; ^{1}H NMR (400 MHz, $CDCl_3$) δ□3.68 (t, *J* = 5.4 Hz, 2H, NCH_2), 3.55–3.52 (m, 2H, NCH_2), 3.11 (s, 1H, CCH), 1.64–1.51 (m, 6H, 3 × CH_2). ^{13}C NMR (101 MHz, $CDCl_3$) δ□151.6 (C=O), 78.9 (*C*CH), 75.6 (C*C*H), 48.0 (NCH_2), 42.2 (NCH_2), 26.2 (CH_2), 25.2 (CH_2), 24.3 (CH_2). Analytical data (NMR and IR) in agreement with those reported in the literature.[17]

***N*-Methoxy- *N*-methylpropiolamide (S13a)[18]**

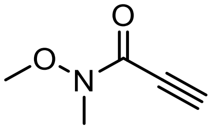

Diisopropylethylamine (1.9 mL, 11 mmol, 1.1 equiv) was added to a stirred solution of propiolic acid (625 □L, 10 mmol, 1.0 equiv) and *N,O*-dimethylhydroxyamine hydrochloride (1.07 g, 11 mmol, 1.1 equiv) in anhydrous CH_2Cl_2 (20 mL, 0.5 M) at 0 °C, followed by the addition of *N*,*N*'-dicyclohexylcarbodiimide (2.27 g, 22 mmol, 1.1 equiv) at 0 °C. The resulting mixture was slowly warmed to rt for 18 h. The reaction mixture was concentrated under reduced pressure, diluted with EtOAc (20 mL) and filtered. H_2O (20 mL) was added to the filtrate, separated, and the aqueous layer was further extracted with EtOAc (2 × 10 mL). The combined organic layer was dried over Na_2SO_4, filtered and concentrated under reduced pressure. Purification by flash column chromatography (SiO_2, 30% EtOAc in pentane) afforded *N*-methoxy-*N*-methylpropiolamide **S13a** as a pale-red liquid (495 mg, 44%). R_f 0.29 (30% EtOAc in pentane); IR (film)/cm^{-1} 3225, 2977, 2938, 2106, 1632 (C=O), 1414, 1382, 1186, 1109, 996, 924, 723, 579; ^{1}H NMR (400 MHz, $CDCl_3$) δ□3.74 (s, 3H, OCH_3), 3.18 (s, 3H, NCH_3), 3.14 (s, 1H, CCH); ^{13}C NMR (101 MHz, $CDCl_3$) δ□ 153.0 (C=O), 78.8 (*C*CH), 74.8 (C*C*H), 62.1 (OCH_3), 32.1 (NCH_3). Analytical data (NMR and IR) in agreement with those reported in the literature.[18]

N-Propylpropiolamide (S14b)[19]

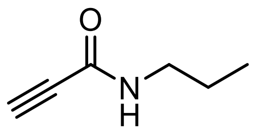

Methyl propiolate (890 μL, 10 mmol, 1.0 equiv) was added dropwise to a stirred solution of propylamine (863 μL, 10.5 mmol, 1.05 equiv) in H_2O (3 mL) at 0 °C. The resulting mixture was stirred at 0 °C for 2 h followed by the addition of acetic acid (0.2 mL) and stirring for 10 min at 0 °C. The reaction mixture was diluted with brine (5 mL) and extracted with EtOAc (3 × 5 mL). The combined organic layer was washed with saturated $NaHCO_3$ solution (5 mL), dried over Na_2SO_4 and concentrated under reduced pressure. Purification by flash column chromatography (SiO_2, 50% EtOAc in hexane) afforded *N*-propylpropiolamide **S14b** as a pale-yellow oil (0.72 g, 65%). R_f 0.33 (50% EtOAc in hexane); IR (film)/cm^{-1} 3246, 3062, 2934, 2876, 2106, 1624 (C=O), 1533, 1268, 1149, 942, 656, 587; ^1H NMR (400 MHz, $CDCl_3$) δ 6.21 (br s, 1H, NH), 3.25 (qd, *J* = 8.2, 7.1, 2.2 Hz, 1H), 2.78 (s, 1H, CCH), 1.55 (pd, *J* = 7.4, 3.8 Hz, 1H), 0.92 (td, *J* = 7.5, 2.1 Hz, 2H); ^{13}C NMR (101 MHz, $CDCl_3$) δ 152.2 (C=O), 79.2 (*C*CH), 72.9 (C*C*H), 41.5 (NCH_2), 22.4 (NCH_2CH_2), 11.2 (CH_3). Analytical data (NMR and IR) in agreement with those reported in the literature.[20]

1-Phenylprop-2-yn-1-one (S15a)[21]

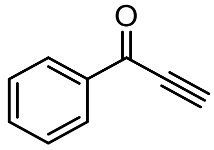

A solution of CrO_3 (2.88 g, 28.8 mmol, 1.44 equiv) and H_2SO_4 (2.48 mL) in H_2O (10.8 mL in total) was added to a stirred solution of 1-phenyl-2-propyn-1-ol (2.43 mL, 20 mmol, 1.0 equiv) in acetone (100 mL, 0.2 M) at 0 °C. The resulting mixture was stirred at 0 °C for 5 min and then warmed to rt for 30 min. The reaction mixture was concentrated under reduced pressure, diluted with H_2O (20 mL) and extracted with CH_2Cl_2 (3 × 20 mL). The combined organic layer was dried over Na_2SO_4 and concentrated under reduced pressure. Purification by flash column chromatography (SiO_2, 3% EtOAc in hexane) afforded phenyl propargyl ketone **S15a** as a white solid (1.43 g, 55%). R_f 0.37 (5% EtOAc in hexane); IR (film)/cm^{-1} 3228, 3065, 2920, 2090, 1638 (C=O), 1594, 1576, 1450, 1258, 1002, 937, 738, 693; ^1H NMR (400 MHz, $CDCl_3$) δ 8.19–8.17 (m, 2H, 2 × Ar–H), 7.68–7.63 (m, 1H, Ar–H), 7.52 (t, *J* = 7.8 Hz, 2H, 2 × Ar–H), 3.45 (s, 1H, CCH); ^{13}C NMR (101 MHz, $CDCl_3$) δ 177.4 (C=O), 136.2 (Ar–C_q), 134.5 (Ar–C), 129.7 (2 × Ar–C), 128.7 (2 × Ar–C), 80.7 (C*C*H), 80.3 (*C*CH). Analytical data (NMR and IR) in agreement with those reported in the literature.[22]

(Ethynylsulfonyl)benzene (S16a)[23]

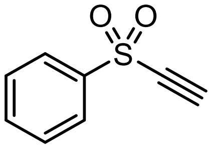

Aluminium chloride (2.67 g, 20 mmol, 1.0 equiv) was added to a stirred solution of benzenesulfonyl chloride (3.06 mL, 24 mmol, 1.2 equiv) and trimethylsilylacetylene (2.85 mL, 20 mmol, 1.0 equiv) in anhydrous CH_2Cl_2 (40 mL) at 0 °C. The resulting mixture was slowly warmed to rt for 72 h. The reaction mixture was quenched with H_2O (20 mL), separated, and extracted with CH_2Cl_2 (3 × 10 mL). The combined organic layer was dried over Na_2SO_4 and concentrated under reduced pressure. Purification by flash column chromatography (SiO_2, 30% EtOAc in pentane) afforded alkynyl phenyl sulfone **S16a** as a brown oil (0.63 g, 19%). R_f 0.36 (30% EtOAc in pentane); IR (film)/cm^{-1} 3243, 3066, 2066, 1583, 1447, 1161, 1087, 736, 682, 573; ^{1}H NMR (400 MHz, $CDCl_3$) δ 8.03–7.99 (m, 2H, 2 × Ar–H), 7.74–7.70 (m, 1H, Ar–H), 7.60 (td, *J* = 7.9, 2.3 Hz, 2H, 2 × Ar–H), 3.59–3.51 (m, 1H, CCH); ^{13}C NMR (101 MHz, $CDCl_3$) δ 140.5 (Ar–C_q), 134.7 (Ar–C), 129.4 (2 × Ar–C), 127.5 (2 × Ar–C), 81.9 (*C*CH), 79.9 (C*C*H); Analytical data (NMR and IR) in agreement with those reported in the literature.[24]

***N*-Methyl-*N*-(3-phenyl-3-(4-(trifluoromethyl)phenoxy)propyl)propiolamide (S17a)**

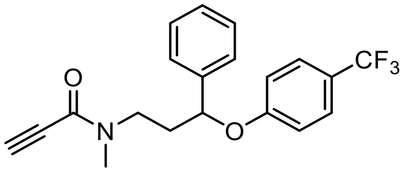

N,*N*'-Dicyclohexylcarbodiimide (619 mg, 3.0 mmol, 2.0 equiv) was added to a stirred solution of propiolic acid (185 μL, 3.0 mmol, 2.0 equiv) in anhydrous CH_2Cl_2 (1.5 mL) at 0 °C. The resulting mixture was stirred at 0 °C for 1 h. Diisopropylethylamine (520 μL, 3.0 mmol, 2.0 equiv) was added to a stirred solution of fluoxetine hydrochloride (519 mg, 2.0 mmol, 2.0 equiv) in anhydrous CH_2Cl_2 (1.5 mL) at 0 °C. After stirring at 0 °C for 30 min, fluoxetine in CH_2Cl_2 solution was transferred to the solution with propiolic acid and slowly warmed to rt overnight. The reaction mixture was concentrated, diluted with EtOAc (10 mL) and filtered. H_2O (10 mL) was added and the aqueous layer was extracted with EtOAc (3 × 5 mL). The combined organic layer was dried over Na_2SO_4 and concentrated under reduced pressure. Purification by flash column chromatography (SiO_2, 20% to 30% EtOAc in pentane) afforded propargylic amide **S17a** as a pale-orange gum (259 mg, 48%). R_f 0.4 (40% EtOAc in pentane); IR (film)/cm^{-1} 3282, 2931, 2857, 2103, 1631 (C=O), 1515, 1451, 1323, 1245, 1107, 1065, 1006, 835, 700, 638, 591; ^{1}H NMR (400 MHz, $CDCl_3$, observed as a mixture of 2 rotamers) δ 7.43 (d, *J* = 8.4 Hz, 2H, 2 × Ar–H), 7.38–7.26 (m, 5H, 5 × Ar–H), 6.91–6.87 (m, 2H, 2 × Ar–H), 5.23–5.19 (m, 1H, OCH), 3.99–3.68 (m, 1H, NC*H*H), 3.63 (t, *J* = 7.2 Hz, 1H, NCH*H*), 3.21 and 2.98 (s, 3H, NCH_3), 3.12 and 2.84 (s, 1H, CCH), 2.32–2.09 (m, 2H, OCHC*H*$_2$); ^{13}C NMR (101 MHz, $CDCl_3$, observed as a mixture of 2 rotamers) δ 160.1 and 160.0 (Ar–C_q), 153.4 and 153.4 (C=O), 140.3 and 140.0 (Ar–C_q), 129.0 and 128.8 (2 × Ar–C), 128.2 and 128.0 (Ar–C), 126.8 and 126.7 (2 × Ar–C), 125.7 and 125.6 (2 × Ar–C), 115.7 and 115.6 (2 × Ar–C), 79.0 and 78.6 (C*C*H), 78.1 and 77.2 (OCH), 75.8 and 75.5 (*C*CH), 47.7 and 44.0 (NCH_2), 37.2 and 35.8 (NCH_2*C*H_2), 36.7 and 32.4 (NCH_3); ^{19}F (377 MHz, $CDCl_3$, observed as a mixture of 2 rotamers) δ –61.6 and –61.6 (CF_3); HRMS (ESI-TOF) *m/z*: Calcd. for $C_{20}H_{19}NO_2F_3$ $[M+H]^+$: 362.1368, found: 362.1371.

N-(3-(10,11-Dihydro-*5H*-dibenzo[*a*,*d*][7]annulen-5-ylidene)propyl)-*N*-methylpropiolamide (S18a)

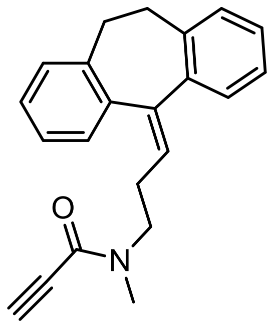

n-BuLi (1.5 M in hexane, 0.73 mL, 1.1 mmol, 1.1 equiv) was added dropwise to a stirred solution of nortriptyline hydrochloride (0.3 g, 1.0 mmol, 1.0 equiv) in anhydrous THF (3 mL) at –78 °C. The resulting mixture was slowly warmed to rt for 1 h. *n*-BuLi (1.5 M in hexane, 0.87 mL, 1.3 mmol, 1.3 equiv) was added dropwise to a stirred solution of propiolic acid (74 □L, 1.2 mmol, 1.2 equiv) in anhydrous THF (3 mL) at –78 °C. The resulting mixture was slowly warmed to rt for 45 min followed by dropwise addition of trimethylacetyl chloride (150 □L, 1.2 mmol, 1.2 equiv) at 0 °C. The resulting mixture was slowly warmed to rt for 2 h before transferring to the solution of nortriptyline and stirred overnight at rt. The reaction mixture was quenched with saturated aqueous NH_4Cl solution (5 mL) and extracted with EtOAc (3 × 5 mL). The combined organic layer was dried over Na_2SO_4 and concentrated under reduced pressure. Purification by flash column chromatography (SiO_2, 30% EtOAc in pentane) afforded propargylic amide **S18a** as a pale-yellow gum (234 mg, 74%). R_f 0.44 (40% EtOAc in pentane); IR (film)/cm^{-1} 3268, 3060, 3016, 2920, 2102, 1626 (C=O), 1483, 1429, 1400, 1195, 1124, 909, 756, 729, 595; ^{1}H NMR (400 MHz, $CDCl_3$, observed as a mixture of 2 rotamers) δ□7.31–7.26 (m, 1H, Ar–H), 7.23–7.10 (m, 6H, 6 × Ar–H), 7.08–7.03 (m, 2H, 2 × Ar–H), 5.84 (t, *J* = 7.6 Hz, 1H, C=CH), 3.75–3.30 (m, 4H, 2 × CH_2), 3.11–2.78 (m, 6H, CCH and CH_2 and NCH_3), 2.51–2.37 (m, 2H, CH_2); ^{13}C NMR (101 MHz, $CDCl_3$, observed as a mixture of 2 rotamers) δ□153.2 (C=O), 145.9 and 145.0 (Ar–*C*CH), 140.8 and 140.6 (Ar–C_q), 139.6 and 139.5 (Ar–C_q), 139.4 and 139.3 (Ar–C_q), 137.0 and 136.9 (Ar–C_q), 130.1 and 130.0 (Ar–C), 128.5 and 128.4 (Ar–C), 128.1 and 128.0 (Ar–C), 128.1 and 127.8 (Ar–C), 127.7 and 127.6 (Ar–C), 127.3 and 127.2 (Ar–C), 127.0 and 126.2 (C*C*H), 126.0 and 126.0 (Ar–C), 125.8 and 125.8 (Ar–C), 78.8 and 78.6 (C*C*H), 76.0 and 75.8 (*C*CH), 53.4 and 50.7 (NCH_2), 46.1 and 36.1–31.8 (NCH_3 and 2 × CH_2), 28.2 and 27.0 (CH_2); HRMS (ESI-TOF) *m/z*: Calcd. for $C_{22}H_{22}NO$ $[M+H]^+$: 316.1700, found: 316.1701.

(*S*)-4-(2-((*tert*-Butoxycarbonyl)amino)-3-methoxy-3-oxopropyl)phenyl propiolate (S19a)

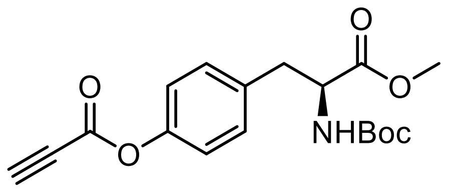

Prepared on 1 mmol scale using procedure reported by Cervi.[25] **S19a** was afforded as a colourless oil (302 mg, 87%). R_f 0.17 (15% EtOAc in pentane); IR (film)/cm^{-1} 2978, 2122, 1728 (C=O), 1703 (C=O), 1502, 1440, 1365, 1182, 1159, 1055, 1017, 912, 732; ^{1}H NMR (400 MHz, $CDCl_3$) δ□7.17 (d, *J* = 7.7 Hz, 2H, 2 × Ar–H), 7.08 (dt, *J* = 8.9, 2.2 Hz, 2H, 2 × Ar–H), 5.03–4.79 (m, 1H, NH), 4.61–4.39 (m, 1H, NCH), 3.71 (s, 3H, CO_2CH_3), 3.16–3.01 (m, 3H, Ar–CH_2 and CCH), 1.41 (s, 9H, $C(CH_3)_3$); ^{13}C NMR (101 MHz, $CDCl_3$) δ□172.1 (C=O), 155.0 (C=O), 150.8 (Ar–C_q), 148.8 (C=O), 134.6

(Ar–C_q), 130.4 (2 × Ar–C), 121.2 (2 × Ar–C), 80.0 (*C*(CH_3)$_3$), 76.8 (C*C*H), 74.2 (*C*CH), 54.3 (NCH), 52.3 (CO_2CH_3), 37.7 (Ar–CH_2), 28.2 (C(*C*H_3)$_3$). Analytical data (NMR and IR) in agreement with those reported in the literature.[25]

1-(4-(2-Chlorodibenzo[*b*,*f*][1,4]oxazepin-11-yl)piperazin-1-yl)prop-2-yn-1-one (S20a)

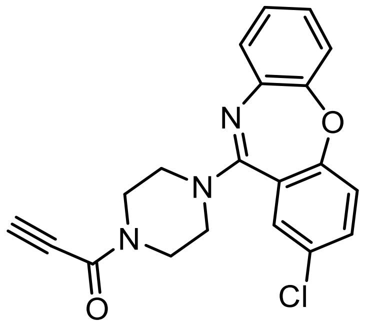

N,*N*'-Dicyclohexylcarbodiimide (194 mg, 0.94 mmol, 1.2 equiv) was added to a stirred solution of propiolic acid (58 □L, 0.94 mmol, 1.2 equiv) and amoxapine (244 mg, 0.78 mmol, 1.0 equiv) in anhydrous CH_2Cl_2 (1.6 mL, 0.5 M) at 0 °C. The resulting mixture was slowly warmed to rt for 18 h. The reaction mixture was concentrated under reduced pressure, diluted with EtOAc (20 mL) and filtered. H_2O (20 mL) was added to the filtrate, separated, and the aqueous layer was further extracted with EtOAc (2 × 10 mL). The combined organic layer was dried over Na_2SO_4, filtered and concentrated under reduced pressure. Purification by flash column chromatography (SiO_2, 25% EtOAc in hexane) afforded alkynyl amide **S20a** as a yellow solid (281 mg, 98%). Mp: 204–206 °C; R_f 0.5 (40% EtOAc in hexane); IR (film)/cm^{-1} 3185, 2984, 2930, 2097, 1631 (C=O), 1586, 1400, 1232, 1187, 1019, 829, 669; ^{1}H NMR (400 MHz, $CDCl_3$) δ□7.42 (dd, *J* = 8.6, 2.6 Hz, 1H, Ar–H), 7.32 (d, *J* = 2.6 Hz, 1H, Ar–H), 7.21 (d, *J* = 8.7 Hz, 1H, Ar–H), 7.17–7.09 (m, 3H, 3 × Ar–H), 7.05–7.01 (m, 1H, Ar–H), 3.92 (s, 2H, CH_2), 3.77 (s, 2H, CH_2), 3.61 (s, 2H, CH_2), 3.53 (s, 2H, CH_2), 3.18 (s, 1H, CCH); ^{13}C NMR (101 MHz, $CDCl_3$) δ□159.3 (Ar–C_q), 158.5 (C=N), 151.9 (C=O), 151.7 (Ar–C_q), 139.6 (Ar–C_q), 132.9 (Ar–H), 130.5 (Ar–C_q), 128.7 (Ar–H), 127.1 (Ar–H), 125.8 (Ar–H), 125.1 (Ar–H), 124.6 (Ar–C_q), 122.9 (Ar–H), 120.2 (Ar–H), 79.7 (C*C*H), 75.1 (*C*CH), 47.6 (CH_2), 47.1 (CH_2), 46.5 (CH_2), 41.2 (CH_2); HRMS (ESI-TOF) *m/z*: Calcd. for $C_{20}H_{17}N_3O_2Cl$ $[M+H]^+$: 366.1009, found: 366.1012.

Methyl 3-bromopropiolate (S23a)[26]

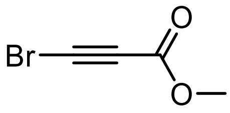

$AgNO_3$ (255 mg, 1.5 mmol, 0.1 equiv) was added to a stirred solution of methyl propiolate (1.33 mL, 15 mmol, 1.0 equiv) and *N*-bromosuccinimide (3.07 g, 1.15 equiv, 17.25 mmol) in acetone (50 mL) at rt. The resulting mixture was stirred at rt for 18 h. The reaction mixture was concentrated under reduced pressure, diluted with pentane (100 mL) and filtered through a short pad of Celite followed by a short pad of silica gel. The short pad of silica gel was washed with pentane (50 mL) and the combined organic layer was concentrated under reduced pressure to afford methyl 3-bromopropiolate **S23a** as a pale-yellow liquid (2.10 g, 86%) which was used directly without further purification. R_f 0.1 (pentane); IR (film)/cm^{-1} 2955, 2200, 1706, 1434, 1234, 1011, 887, 745, 720, 547; ^{1}H

NMR (400 MHz, $CDCl_3$) δ 3.76 (s, 3H, CO_2CH_3); ^{13}C NMR (101 MHz, $CDCl_3$) δ 152.8 (C=O), 72.4 (*C*CBr), 52.9 (CO_2*C*H_3), 52.8 (C*C*Br). Analytical data (NMR and IR) in agreement with those reported in the literature.[26]

Methyl 3-iodopropiolate (S24a)

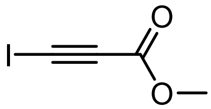

$AgNO_3$ (255 mg, 1.5 mmol, 0.1 equiv) was added to a stirred solution of methyl propiolate (1.33 mL, 15 mmol, 1.0 equiv) and *N*-iodosuccinimide (3.88 g, 1.15 equiv, 17.25 mmol) in acetone (50 mL) at rt. The resulting mixture was stirred at rt for 18 h. The reaction mixture was concentrated under reduced pressure, diluted with pentane (100 mL) and filtered through a short pad of Celite followed by a short pad of silica gel. The short pad of silica gel was washed with pentane (50 mL) and the combined organic layer was concentrated under reduced pressure to afford methyl 3-iodopropiolate **S24a** as a white solid (1.89 g, 60%) which was used directly without further purification. R_f 0.1 (pentane); IR (film)/cm^{-1} 2951, 2169, 1673, 1429, 1260, 1001, 876, 743, 548; ^{1}H NMR (400 MHz, $CDCl_3$) δ 3.79 (s, 3H, CO_2CH_3); ^{13}C NMR (101 MHz, $CDCl_3$) δ 152.7 (C=O), 86.7 (*C*CI), 53.0 (CO_2*C*H_3), 13.7 (C*C*I); HRMS (ESI-TOF) *m/z*: Calcd. for $C_4H_4IO_2$ $[M+H]^+$: 210.9250, found: 210.9252.

Ethyl 4-oxo-4-(piperidin-1-yl)but-2-ynoate (S25a)

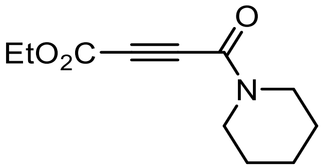

n-BuLi (1.5 M in hexane, 3.7 mL, 5.5 mmol, 1.1 equiv) was added dropwise to a stirred solution of 1-(piperidin-1-yl)prop-2-yn-1-one (0.69 g, 5.0 mmol, 1.0 equiv) in anhydrous THF (12.5 mL) at –78 °C. The resulting mixture was stirred at –78 °C for 1 h followed by dropwise addition of ethyl chloroformate (520 μL, 5.5 mmol, 1.1 equiv). The resulting mixture was slowly warmed to rt for 3 h followed by quenching with saturated aqueous NH_4Cl solution (10 mL) and extracted with EtOAc (3 × 5 mL). The combined organic layer was washed with brine (10 mL), dried over Na_2SO_4 and concentrated under reduced pressure. Purification by flash column chromatography (SiO_2, 15% EtOAc in pentane) afforded amide-ester alkyne **S25a** as a colourless oil (0.34 g, 34%). R_f 0.44 (30% EtOAc in pentane); IR (film)/cm^{-1} 2941, 2860, 1714 (C=O), 1630 (C=O), 1434, 1368, 1235, 1052, 1011, 910, 725, 627; ^{1}H NMR (400 MHz, $CDCl_3$) δ 4.25 (q, *J* = 7.1 Hz, 2H, C*H*$_2$CH$_3$), 3.63 (t, *J* = 5.4 Hz, 2H, NCH_2), 3.54 (t, *J* = 5.4 Hz, 2H, NCH_2), 1.66–1.52 (m, 6H, 3 × CH_2), 1.29 (t, *J* = 7.1 Hz, 3H, CH_2C*H*$_3$); ^{13}C NMR (101 MHz, $CDCl_3$) δ 152.3 (C=O), 150.2 (C=O), 79.8 (*C*CCO$_2$Et), 75.9 (C*C*CO$_2$Et), 62.6 (*C*H$_2$CH$_3$), 48.0 (NCH_2), 42.4 (NCH_2), 26.3 (CH_2), 25.1 (CH_2), 24.2 (CH_2), 13.8 (CH_3); HRMS (ESI-TOF) *m/z*: Calcd. for $C_{11}H_{16}NO_3$ $[M+H]^+$: 210.1130, found: 210.1128.

General Procedure G for Cycloaddition between α-diazo sulfoximines and mono-substituted alkynes

Alkyne (1.0 mmol, 5.0 equiv) was added to a stirred solution of a-diazo sulfoximine (0.2 mmol, 1.0 equiv) in anhydrous toluene (1.0 mL, 0.2 M) at rt. The resulting mixture was stirred at rt until TLC analysis showed the complete consumption of the diazo compound. The reaction mixture was concentrated under reduced pressure followed by purification by flash column chromatography (SiO_2) to afford the corresponding pyrazolesulfoximine.

Methyl 3-(*N*-(*tert*-butyldiphenylsilyl)-4-methylphenylsulfonimidoyl)-1*H*-pyrazole-5-carboxylate (9a)

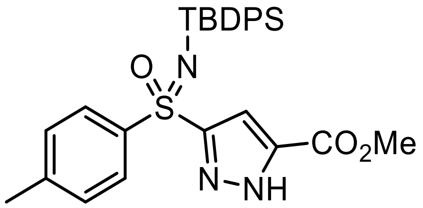

Prepared using general procedure G with α-diazo sulfoximine **8a** (86.7 mg, 0.2 mmol, 1.0 equiv) and methyl propiolate (89 □L, 1.0 mmol, 5.0 equiv). Purification by flash column chromatography (SiO_2, 20% Et_2O and 30% CH_2Cl_2 in hexane) afforded α-pyrazole sulfoximine **9a** as a colourless foam (82.9 mg, 80%). R_f 0.23 (20% Et_2O and 30% CH_2Cl_2 in hexane); IR (film)/cm^{-1} 3213, 3071, 2930, 2855, 1730 (C=O), 1431, 1349, 1297, 1156, 813, 701; ^{1}H NMR (400 MHz, $CDCl_3$) δ□11.23 (s, 1H, NH), 7.92–7.90 (m, 2H, 2 × Ar–H), 7.72–7.69 (m, 4H, 4 × Ar–H), 7.32–7.21 (m, 8H, 8 × Ar–H), 6.87 (s, 1H, Py–H), 3.87 (s, 3H, CO_2CH_3), 2.37 (s, 3H, Ar–CH_3), 1.11 (s, 9H, $C(CH_3)_3$); ^{13}C NMR (101 MHz, $CDCl_3$) δ□159.6 (C=O), 143.3 (Ar–C_q), 141.0 (Ar–C_q), 135.6 (2 × Ar–C_q), 135.5 (2 × Ar–C), 135.5 (2 × Ar–C), 129.5 (2 × Ar–C), 128.9 (2 × Ar–C), 127.7 (2 × Ar–C), 127.3 (4 × Ar–C), 109.0 (Py–CH), 52.5 (CO_2CH_3), 27.1 ($C(CH_3)_3$), 21.5 (Ar–CH_3), 19.4 ($C(CH_3)_3$); HRMS (ESI-TOF) *m/z*: Calcd. for $C_{28}H_{32}N_3O_3SSi$ $[M+H]^+$: 518.1934, found: 518.1941.

Methyl 3-(N-(*tert*-butyldiphenylsilyl)-4-methoxyphenylsulfonimidoyl)-1*H*-pyrazole-5-carboxylate (9b)

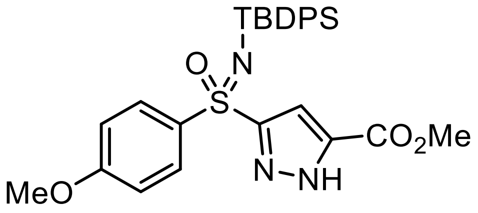

Prepared using general procedure G with α-diazo sulfoximine **8b** (90.1 mg, 0.2 mmol, 1.0 equiv) and methyl propiolate (89 □L, 1.0 mmol, 5.0 equiv). Purification by flash column chromatography (SiO_2, 20% to 35% EtOAc in pentane) afforded α-pyrazole sulfoximine **9b** as a colourless foam (90.0 mg, 84%). R_f 0.18 (35% EtOAc in pentane); IR (film)/cm^{-1} 3070, 2952, 2932, 2890, 2854, 1729 (C=O), 1591, 1494, 1435, 1348, 1309, 1257, 1151, 1105, 1013, 830, 802, 701, 603; ^{1}H NMR (400 MHz, $CDCl_3$) δ□11.58 (s, 1H, NH), 7.97 (d, *J* = 8.5 Hz, 2H, 2 × Ar–H), 7.73–7.70 (m, 4H, 4 × Ar–H), 7.34–7.24 (m, 6H, 6 × Ar–H), 6.89 (d, *J* = 8.5 Hz, 2H, 2 × Ar–H), 6.88 (s, 1H, Py–H), 3.87 (s, 3H, CO_2CH_3), 3.82 (s, 3H, OCH_3), 1.13 (s, 9H, $C(CH_3)_3$); ^{13}C NMR (101 MHz, $CDCl_3$) δ□162.8 (C=O), 159.7 (Ar–C_q), 135.6 (2 × Ar–C_q), 135.5 (2 × Ar–C), 135.5 (2 × Ar–C), 130.1 (Ar–C_q), 129.9 (2 × Ar–C), 128.9 (2 × Ar–C), 127.3 (4 × Ar–C),

114.0 (2 × Ar–C), 108.9 (Py–CH), 55.5 (OCH_3), 52.4 (CO_2CH_3), 27.1 ($C(CH_3)_3$), 19.4 ($C(CH_3)_3$); HRMS (ESI-TOF) *m/z*: Calcd. for $C_{28}H_{32}N_3O_4SSi$ $[M+H]^+$: 534.1883, found: 534.1882.

Methyl 3-(*N*-(*tert*-butyldiphenylsilyl)-4-(trifluoromethyl)phenylsulfonimidoyl)-1*H*-pyrazole-5-carboxylate (9c)

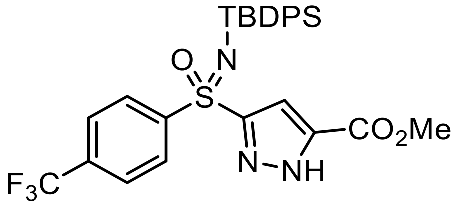

Prepared using general procedure G with α-diazo sulfoximine **8c** (97.4 mg, 0.2 mmol, 1.0 equiv) and methyl propiolate (89 □L, 1.0 mmol, 5.0 equiv). Purification by flash column chromatography (SiO_2, 10% to 25% EtOAc in pentane) afforded α-pyrazole sulfoximine **9c** as a colourless foam (88.0 mg, 77%). R_f 0.45 (35% EtOAc in pentane); IR (film)/cm^{-1} 3071, 2954, 2932, 2890, 2856, 1730 (C=O), 1551, 1429, 1318, 1217, 1162, 1138, 1012, 908, 842, 730, 700, 612; ^{1}H NMR (400 MHz, $CDCl_3$) δ□11.55 (s, 1H, NH), 8.08 (d, *J* = 8.1 Hz, 2H, 2 × Ar–H), 7.71–7.63 (m, 4H, 4 × Ar–H), 7.62 (d, *J* = 8.2 Hz, 2H, 2 × Ar–H), 7.35–7.23 (m, 6H, 6 × Ar–H), 7.01 (s, 1H, Py–H), 3.90 (s, 3H, CO_2CH_3), 1.13 (s, 9H, $C(CH_3)_3$); ^{13}C NMR (101 MHz, $CDCl_3$) δ□159.3 (C=O), 147.3 ($Ar–C_q$), 135.5 (2 × Ar–C), 135.5 (2 × Ar–C), 135.1 (2 × $Ar–C_q$), 133.9 (q, *J* = 33.0 Hz, CCF_3), 129.1 (2 × Ar–C), 128.1 (2 × Ar–C), 127.4 (4 × Ar–C), 125.9 (Ar–C), 125.9 (Ar–C), 123.2 (q, *J* = 273.2 Hz, CF_3), 109.6 (Py–CH), 52.6 (CO_2CH_3), 27.0 ($C(CH_3)_3$), 19.3 ($C(CH_3)_3$); ^{19}F (377 MHz, $CDCl_3$) δ□-63.1; HRMS (ESI-TOF) *m/z*: Calcd. for $C_{28}H_{29}N_3O_3SiSF_3$ $[M+H]^+$: 572.1651, found: 572.1652.

Large scale reaction: Methyl propiolate (0.23 mL, 2.6 mmol, 2 equiv.) was added to a solution of α-diazo sulfoximine **8c** (634 mg, 1.3 mmol, 1 equiv.) in PhMe (6.5 mL) and the resulting reaction mixture was stirred at room temperature for 16 h. The solvent was removed under reduced pressure and the crude reaction mixture was purified by column chromatography (SiO_2, 10 to 50% EtOAc in pentane) to afford α-pyrazole sulfoximine **9c** as a colourless foam (549 mg, 0.96 mmol, 74%).

Methyl (*R*)-3-(4-bromo-*N*-(*tert*-butyldiphenylsilyl)phenylsulfonimidoyl)-1*H*-pyrazole-5-carboxylate ((*R*)-9d)

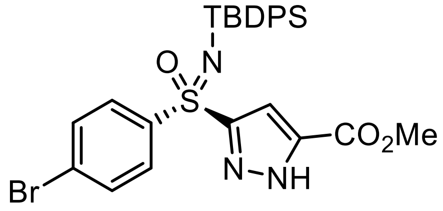

Prepared using general procedure G with α-diazo sulfoximine **(*R*)-8d** (99.8 mg, 0.2 mmol, 1.0 equiv) and methyl propiolate (89 □L, 1.0 mmol, 5.0 equiv). Purification by flash column chromatography (SiO_2, 10% to 25% EtOAc in pentane) afforded α-pyrazole sulfoximine **(*R*)-9d** as a colourless foam (83.4 mg, 72%, 97% *ee*). R_f 0.4 (35% EtOAc in pentane); IR (film)/cm^{-1} 3070, 2953, 2930, 2889, 2854, 1728 (C=O), 1570, 1468, 1345, 1309, 1154, 1105, 1008, 907, 820, 727, 698, 606; ^{1}H NMR (400 MHz, $CDCl_3$) δ□11.35 (s, 1H, NH), 7.84 (d, *J* = 8.5 Hz, 2H, 2 × Ar–H), 7.70–7.66 (m, 4H, 4 × Ar–H), 7.51 (d, *J* = 8.3 Hz, 2H, 2 × Ar–H), 7.35–7.24 (m, 6H, 6 × Ar–H), 6.94 (s, 1H, Py–H), 3.89 (s, 3H, CO_2CH_3), 1.11 (s, 9H, $C(CH_3)_3$); ^{13}C NMR (101 MHz, $CDCl_3$) δ□159.3 (C=O), 143.0 ($Ar–C_q$), 135.5 (2 × Ar–C), 135.5 (2 × Ar–C), 135.3 (2 × $Ar–C_q$), 132.0 (2 × Ar–C), 129.3 (2 × Ar–C), 129.1 (2 × Ar–C), 127.6 ($Ar–C_q$), 127.4 (4 × Ar–C),

109.2 (Py–CH), 52.6 (CO_2CH_3), 27.1 (C(CH_3)$_3$), 19.4 (*C*(CH_3)$_3$); HRMS (ESI-TOF) *m/z*: Calcd. for $C_{27}H_{29}N_3O_3SiSBr$ $[M+H]^+$: 582.0882, found: 582.0890. $[\alpha]^{22}_D$ =□–3 (c 1.0, $CHCl_3$).

Methyl 3-(*N*-(*tert*-butyldiphenylsilyl)pyridine-2-sulfonimidoyl)-1*H*-pyrazole-5-carboxylate (9e)

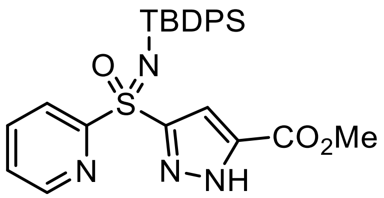

Prepared using general procedure G with α-diazo sulfoximine **8e** (83.9 mg, 0.2 mmol, 1.0 equiv) and methyl propiolate (89 □L, 1.0 mmol, 5.0 equiv). Purification by flash column chromatography (SiO_2, 20% to 40% EtOAc in pentane) afforded α-pyrazole sulfoximine **9e** as an off-white solid (94.1 mg, 93%). R_f 0.26 (50% EtOAc in pentane); Mp: 135–138 °C; IR (film)/cm^{-1} 3069, 2953, 2931, 2890, 2855, 1729 (C=O), 1577, 1424, 1345, 1217, 1168, 1108, 1011, 908, 731, 701, 604; ^{1}H NMR (400 MHz, $CDCl_3$) δ□12.21 (s, 1H, NH), 8.53 (dd, *J* = 4.8, 1.6 Hz, 1H, Ar–H), 8.12 (d, *J* = 7.9 Hz, 1H, Ar–H), 7.75 (td, *J* = 7.9, 1.7 Hz, 1H, Ar–H), 7.71–7.68 (m, 4H, 4 × Ar–H), 7.33–7.23 (m, 7H, 7 × Ar–H), 7.14 (s, 1H, Py–H), 3.88 (s, 3H, CO_2CH_3), 1.11 (s, 9H, C(CH_3)$_3$); ^{13}C NMR (101 MHz, $CDCl_3$) δ□160.5 (C=O), 160.1 (Ar–C_q), 149.7 (Ar–C), 137.8 (Ar–C), 135.5 (4 × Ar–C), 135.3 (Ar–C_q), 135.2 (Ar–C_q), 129.0 (2 × Ar–C), 127.3 (4 × Ar–C), 126.2 (Ar–C), 121.6 (Ar–C), 111.0 (Py–CH), 52.4 (CO_2CH_3), 27.0 (C(*C*H_3)$_3$), 19.3 (*C*(CH_3)$_3$); HRMS (ESI-TOF) *m/z*: Calcd. for $C_{26}H_{29}N_4O_3SiS$ $[M+H]^+$: 505.1730, found: 505.1728.

Methyl 3-(*N*-(*tert*-butyldiphenylsilyl)cyclohexanesulfonimidoyl)-1*H*-pyrazole-5-carboxylate (9f)

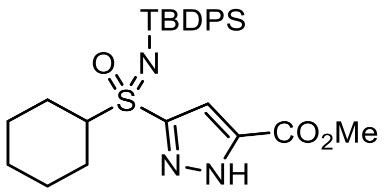

Prepared using general procedure G with α-diazo sulfoximine **8f** (83.9 mg, 0.2 mmol, 1.0 equiv) and methyl propiolate (89 □L, 1.0 mmol, 5.0 equiv). Purification by flash column chromatography (SiO_2, 10% to 20% EtOAc in pentane) afforded α-pyrazole sulfoximine **9f** as a colourless foam (80.4 mg, 79%). R_f 0.4 (35% EtOAc in pentane); IR (film)/cm^{-1} 3070, 2931, 2855, 1730 (C=O), 1549, 1447, 1346, 1301, 1214, 1177, 1105, 1012, 908, 728, 700, 608; ^{1}H NMR (400 MHz, $CDCl_3$) δ□11.43 (s, 1H, NH), 7.67 (ddd, *J* = 17.2, 7.7, 1.7 Hz, 4H, 4 × Ar–H), 7.34–7.22 (m, 6H, 6 × Ar–H), 6.82 (s, 1H, Py–H), 3.90 (s, 3H, CO_2CH_3), 3.00 (td, *J* = 10.4, 9.0, 5.9 Hz, 1H, SCH), 2.20 (d, *J* = 12.7 Hz, 1H, SCHC*H*H), 2.10–2.06 (m, 1H, SCHCH*H*), 1.87–1.80 (m, 2H, SCHC*H*$_2$), 1.65 (d, *J* = 12.3 Hz, 1H, CH), 1.45–1.35 (m, 2H, 2 × CH), 1.27–1.13 (m, 3H, 3 × CH), 1.08 (s, 9H, C(CH_3)$_3$); ^{13}C NMR (101 MHz, $CDCl_3$) δ□160.1 (C=O), 135.9 (Ar–C_q), 135.7 (Ar–C_q), 135.5 (4 × Ar–C), 128.9 (Ar–C), 128.8 (Ar–C), 127.2 (2 × Ar–C), 127.2 (2 × Ar–C), 110.8 (Py–C), 66.4 (SCH), 52.4 (CO_2CH_3), 27.1 (C(*C*H_3)$_3$), 25.7 (CH_2), 25.6 (CH_2), 25.2 (CH_2), 25.1 (CH_2), 19.3 (*C*(CH_3)$_3$); HRMS (ESI-TOF) *m/z*: Calcd. for $C_{27}H_{36}N_3O_3SiS$ $[M+H]^+$: 510.2247, found: 510.2230.

Methyl 3-(*N*-(*tert*-butyldiphenylsilyl)-*S*-methylsulfonimidoyl)-1*H*-pyrazole-5-carboxylate (9g)

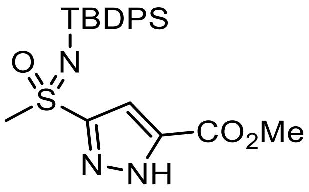

Prepared using general procedure G with α-diazo sulfoximine **8g** (71.5 mg, 0.2 mmol, 1.0 equiv) and methyl propiolate (89 □L, 1.0 mmol, 5.0 equiv). Purification by flash column chromatography (SiO_2, 20% EtOAc in hexane) afforded α-pyrazole sulfoximine **9g** as a colourless foam (86.5 mg, 98%). R_f 0.25 (30% EtOAc in hexane); IR (film)/cm^{-1} 3070, 2954, 2931, 2855, 1731 (C=O), 1348, 1300, 1151, 1013, 945, 702; ^{1}H NMR (400 MHz, $CDCl_3$) δ□ 11.88 (s, 1H, NH), 7.74–7.68 (m, 4H, 4 × Ar–H), 7.38–7.27 (m, 6H, 6 × Ar–H), 7.00 (s, 1H, Py–H), 3.93 (s, 3H, CO_2CH_3), 3.11 (s, 3H SCH_3), 1.09 (s, 9H, $C(CH_3)_3$); ^{13}C NMR (101 MHz, $CDCl_3$) δ□159.5 (C=O), 135.6 (2 × Ar–C_q), 135.5 (2 × Ar–C), 135.5 (2 × Ar–C), 129.1 (Ar–C), 129.0 (Ar–C), 127.4 (2 × Ar–C), 127.4 (2 × Ar–C), 108.6 (Py–C), 52.6 ($CO_2\textit{C}H_3$), 47.9 (SCH_3), 27.0 ($C(\textit{C}H_3)_3$), 19.2 ($\textit{C}(CH_3)_3$); HRMS (ESI-TOF) *m/z*: Calcd. for $C_{22}H_{28}N_3O_3SSi$ $[M+H]^+$: 442.1621, found: 442.1604.

3-(*N*-(*tert*-Butyldiphenylsilyl)-4-methylphenylsulfonimidoyl)-1*H*-pyrazole-5-carboxamide (10a)

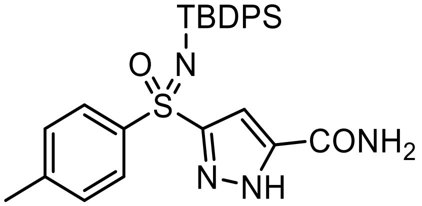

Prepared using general procedure G with α-diazo sulfoximine **8a** (86.7 mg, 0.2 mmol, 1.0 equiv) and propiolamide (69.1 mg, 1.0 mmol, 5.0 equiv). Purification by flash column chromatography (SiO_2, 5% to 20% MeCN in CH_2Cl_2) afforded α-pyrazole sulfoximine **10a** as a white solid (45.4 mg, 45%). R_f 0.18 (20% MeCN in CH_2Cl_2); Mp: 222–225 °C; IR (film)/cm^{-1} 3461, 3068, 2929, 2854, 1662 (C=O), 1425, 1318, 1150, 1105, 730, 700, 604, 496; ^{1}H NMR (400 MHz, $CDCl_3$) δ□12.54 (d, *J* = 126.5 Hz, 1H), 7.89 (d, *J* = 7.9 Hz, 2H, 2 × Ar–H), 7.66 (t, *J* = 8.4 Hz, 4H, 4 × Ar–H), 7.30–7.20 (m, 8H, 8 × Ar–H), 6.89 (s, 1H, Py–H), 6.76–5.97 (m, 2H, $CONH_2$), 2.36 (s, 3H, Ar–CH_3), 1.11 (s, 9H, $C(CH_3)_3$); ^{13}C NMR (101 MHz, $CDCl_3$) δ 154.1 (C=O), 143.6 (Ar–C_q), 141.0 (Ar–C_q), 135.5 (2 × Ar–C), 135.5 (2 × Ar–C), 135.3 (2 × Ar–C_q), 129.6 (2 × Ar–C), 129.1 (2 × Ar–C), 127.5 (2 × Ar–C), 127.3 (4 × Ar–C), 107.2 (Py–CH), 27.1 ($C(\textit{C}H_3)_3$), 21.4 (Ar–CH_3), 19.3 ($\textit{C}(CH_3)_3$); HRMS (ESI-TOF) *m/z*: Calcd. for $C_{27}H_{31}N_4O_2SiS$ $[M+H]^+$: 503.1937, found: 503.1915.□

3-(*N*-(*tert*-Butyldiphenylsilyl)-4-methylphenylsulfonimidoyl)-*N*-phenyl-1*H*-pyrazole-5-carboxamide (11a)

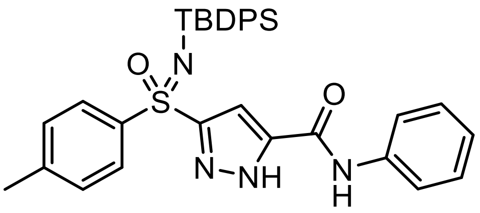

Prepared using general procedure G with α-diazo sulfoximine **8a** (86.7 mg, 0.2 mmol, 1.0 equiv) and *N*-phenylpropiolamide **S11a** (145 mg, 1.0 mmol, 5.0 equiv). Purification by flash column chromatography (SiO_2, 100% CH_2Cl_2 then 40% EtOAc in pentane) afforded α-pyrazole sulfoximine **11a** as an off-white solid (80.1 mg, 69%). R_f 0.09 (CH_2Cl_2); Mp: 142–145 °C; IR (film)/cm^{-1} 3135, 3066, 2953, 2929, 2890, 2854, 1656 (C=O), 1599, 1550, 1534, 1496, 1427, 1317, 1293, 1145, 1106, 1010, 973, 816, 698, 605, 498; ^{1}H NMR (400 MHz, $CDCl_3$) δ 12.03 (s, 1H, NH), 8.61 (s, 1H, NH), 7.95–7.93 (m, 2H, 2 × Ar–H), 7.72–7.68 (m, 4H, 4 × Ar–H), 7.64–7.61 (m, 2H, 2 × Ar–H), 7.35–7.30 (m, 4H, 4 × Ar–H), 7.27–7.23 (m, 6H, 6 × Ar–H), 7.18–7.14 (m, 2H, 2 × Ar–H), 7.05 (s, 1H, Py–H), 2.41 (s, 3H, Ar–CH_3), 1.16 (s, 9H, $C(CH_3)_3$); ^{13}C NMR (101 MHz, $CDCl_3$) δ 157.9 (C=O), 143.8 (Ar–C_q), 140.7 (Ar–C_q), 137.2 (Ar–C_q), 135.5 (2 × Ar–C), 135.5 (2 × Ar–C), 135.1 (Ar–C_q), 135.0 (Ar–C_q), 129.7 (2 × Ar–C), 129.1 (2 × Ar–C), 129.0 (2 × Ar–C), 127.5 (2 × Ar–C), 127.4 (2 × Ar–C), 127.4 (2 × Ar–C), 124.6 (Ar–C), 120.1 (2 × Ar–C), 107.1 (Py–CH), 27.1 ($C(\mathit{C}H_3)_3$), 21.5 (Ar–CH_3), 19.3 ($\mathit{C}(CH_3)_3$); HRMS (ESI-TOF) *m/z*: Calcd. for $C_{33}H_{35}N_4O_2SiS$ $[M+H]^+$: 579.2250, found: 579.2247.

((*tert*-Butyldiphenylsilyl)imino)(5-(piperidine-1-carbonyl)-1*H*-pyrazol-3-yl)(*p*-tolyl)-λ^6-sulfanone (12a)

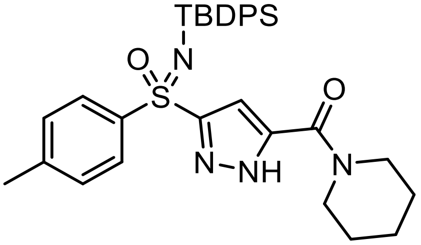

Prepared using general procedure G with α-diazo sulfoximine **8a** (86.7 mg, 0.2 mmol, 1.0 equiv) and α,β-alkynyl tertiary amide **S12a** (137 mg, 1.0 mmol, 5.0 equiv). Purification by flash column chromatography (SiO_2, 20% to 40% EtOAc in hexane) afforded α-pyrazole sulfoximine **12a** as a colourless foam (83.3 mg, 73%). R_f 0.13 (40% EtOAc in hexane); IR (film)/cm^{-1} 3067, 2929, 2853, 2116, 1603 (C=O), 1446, 1322, 1289, 1159, 1104, 985, 817, 731, 700, 649; ^{1}H NMR (400 MHz, $CDCl_3$) δ 12.13 (s, 1H, NH), 7.94 (d, *J* = 8.1 Hz, 2H, 2 × Ar–H), 7.73 (dt, *J* = 7.9, 1.7 Hz, 4H, 4 × Ar–H), 7.34–7.21 (m, 8H, 8 × Ar–H), 6.55 (s, 1H, Py–H), 3.63–3.61 (m, 2H, NCH_2), 3.42 (s, 2H, NCH_2), 2.37 (s, 3H, Ar–CH_3), 1.69–1.61 (m, 2H, CH_2), 1.59 (s, 2H, CH_2), 1.49–1.45 (m, 2H, CH_2), 1.11 (s, 9H, $C(CH_3)_3$); ^{13}C NMR (126 MHz, $CDCl_3$) δ 158.7 (C=O), 143.0 (Ar–C_q), 141.3 (Ar–C_q), 135.8 (Ar–C_q), 135.8 (Ar–C_q), 135.6 (4 × Ar–C), 129.4 (2 × Ar–C), 128.8 (2 × Ar–C), 127.7 (2 × Ar–C), 127.2 (2 × Ar–C), 127.2 (2 × Ar–C), 106.6 (Py–CH), 47.8 (NCH_2), 43.9 (NCH_2), 27.1 ($C(\mathit{C}H_3)_3$), 26.3 (CH_2), 25.5 (CH_2), 24.3 (CH_2), 21.4 (q, *J* = 3.4 Hz, Ar–CH_3), 19.4 ($\mathit{C}(CH_3)_3$); HRMS (ESI-TOF) *m/z*: Calcd. for $C_{32}H_{39}N_4O_2SSi$ $[M+H]^+$: 571.2563, found: 571.2567.

3-(*N*-(*tert*-Butyldiphenylsilyl)-4-methylphenylsulfonimidoyl)-*N*-methoxy-*N*-methyl-1*H*-pyrazole-5-carboxamide (13a)

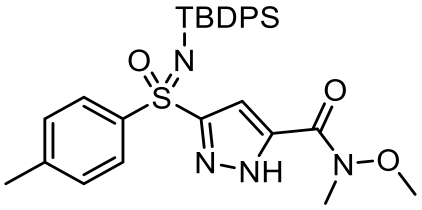

Prepared using general procedure G with α-diazo sulfoximine **8a** (86.7 mg, 0.2 mmol, 1.0 equiv) and *N*-methoxy-*N*-methylpropiolamide **S13a** (113 mg, 1.0 mmol, 5.0 equiv). Purification by flash column chromatography (SiO_2, 15% to 30% EtOAc in pentane) afforded α-pyrazole sulfoximine **13a** as a colourless foam (82.9 mg, 76%). R_f 0.15 (30% EtOAc in pentane); IR (film)/cm^{-1} 3068, 2956, 2931, 2890, 2855, 1621 (C=O), 1543, 1425, 1321, 1294, 1158, 1106, 972, 908, 817, 729, 699, 603; ^{1}H NMR (400 MHz, $CDCl_3$) δ 12.42 (s, 1H, NH), 8.00 (d, *J* = 7.9 Hz, 2H, 2 × Ar–H), 7.80–7.77 (m, 4H, 4 × Ar–H), 7.35–7.21 (m, 8H, 8 × Ar–H), 7.02 (s, 1H, Py–H), 3.53 (s, 3H, OCH_3), 3.32 (s, 3H, NCH_3), 2.36 (s, 3H, Ar–CH_3), 1.15 (s, 9H, $C(CH_3)_3$); ^{13}C NMR (101 MHz, $CDCl_3$) δ 158.2 (Ar–C_q), 157.7 (C=O), 142.8 (Ar–C_q), 141.4 (Ar–C_q), 135.9 (Ar–C_q), 135.8 (Ar–C_q), 135.5 (2 × Ar–C), 135.5 (2 × Ar–C), 134.4 (Ar–C_q), 129.3 (2 × Ar–C), 128.7 (2 × Ar–C), 127.7 (2 × Ar–C), 127.1 (2 × Ar–C), 127.1 (2 × Ar–C), 108.9 (Py–CH), 61.3 (OCH_3), 32.6 (NCH_3), 27.0 ($C(\mathit{C}H_3)_3$), 21.3 (Ar–CH_3), 19.4 ($\mathit{C}(CH_3)_3$); HRMS (ESI-TOF) *m/z*: Calcd. for $C_{29}H_{35}N_4O_3SSi$ $[M+H]^+$: 547.2199, found: 547.2204.

3-(*N*-(*tert*-Butyldiphenylsilyl)-4-methoxyphenylsulfonimidoyl)-*N*-propyl-1*H*-pyrazole-5-carboxamide (14b)

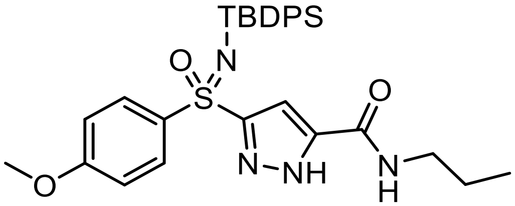

Prepared using general procedure G with α-diazo sulfoximine **8b** (90.1 mg, 0.2 mmol, 1.0 equiv) and methyl propiolate **S14b** (111 mg, 1.0 mmol, 5.0 equiv). Purification by flash column chromatography (SiO_2, 40% EtOAc in pentane) afforded α-pyrazole sulfoximine **14b** as a white solid (95.8 mg, 85%). R_f 0.18 (40% EtOAc in pentane); Mp: 177–180 °C; IR (film)/cm^{-1} 3328, 3135, 3070, 2961, 2933, 2857, 1646 (C=O), 1592, 1495, 1310, 1259, 1150, 1108, 830, 703, 606, 501; ^{1}H NMR (400 MHz, $CDCl_3$) δ 11.42 (d, *J* = 222.4 Hz, 1H, NH), 7.92 (d, *J* = 8.6 Hz, 2H, 2 × Ar–H), 7.70–7.65 (m, 4H, 4 × Ar–H), 7.34–7.24 (m, 6H, 6 × Ar–H), 6.90–6.87 (m, 2H, 2 × Ar–H), 6.71 (s, 1H, Py–H), 3.83 (s, 3H, OCH_3), 3.30 (q, *J* = 6.8 Hz, 2H, NCH_2), 1.56 (qd, *J* = 7.4, 2.3 Hz, 2H, CH_2), 1.11 (s, 9H, $C(CH_3)_3$), 0.93 (td, *J* = 7.6, 2.5 Hz, 3H, CH_3); ^{13}C NMR (101 MHz, $CDCl_3$) δ 163.0 (Ar–C_q), 135.6 (4 × Ar–C), 135.5 (2 × Ar–C_q), 129.7 (2 × Ar–C), 129.1 (2 × Ar–C), 127.4 (4 × Ar–C and Ar–C_q), 114.2 (2 × Ar–C), 55.6 (OCH_3), 41.0 (NCH_2), 27.1 ($C(\mathit{C}H_3)_3$), 22.8 (CH_2), 19.4 ($\mathit{C}(CH_3)_3$), 11.3 (CH_3). HRMS (ESI-TOF) *m/z*: Calcd. for $C_{30}H_{37}N_4O_3SSi$ $[M+H]^+$: 561.2356, found: 561.2357.

Note: The ^{13}C NMR signal for the amide carbonyl (CO-NH) is not observed.

(5-Benzoyl-1*H*-pyrazol-3-yl)((*tert*-butyldiphenylsilyl)imino)(*p*-tolyl)-λ^6-sulfanone (15a)

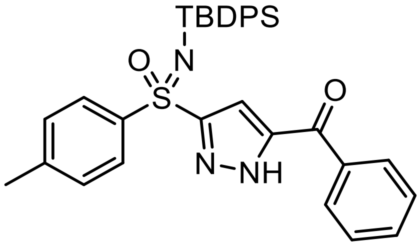

Prepared using general procedure G with α-diazo sulfoximine **8a** (86.7 mg, 0.2 mmol, 1.0 equiv) and benzoylacetylene **S15a** (130 mg, 1.0 mmol, 5.0 equiv). Purification by flash column chromatography (SiO_2, 15% to 30% EtOAc in hexane) afforded α-pyrazole sulfoximine **15a** as a colourless foam (104 mg, 92%). R_f 0.39 (30% EtOAc in hexane); IR (film)/cm^{-1} 3230, 3049, 2929, 2854, 1649 (C=O), 1596, 1319, 1294, 1156, 1104, 971, 898, 817, 729, 698, 605; ^{1}H NMR (400 MHz, $CDCl_3$) δ 11.35 (s, 1H, NH), 7.99 (d, *J* = 8.1 Hz, 2H, 2 × Ar–H), 7.82 (d, *J* = 7.7 Hz, 2H, 2 × Ar–H), 7.73 (dt, *J* = 8.1, 2.1 Hz, 4H, 4 × Ar–H), 7.67–7.62 (m, 1H, Ar–H), 7.49 (t, *J* = 7.7 Hz, 2H, 2 × Ar–H), 7.32–7.23 (m, 8H, 8 × Ar–H), 6.86 (s, 1H, Py–H), 2.39 (s, 3H, Ar–CH_3), 1.14 (s, 9H, $C(CH_3)_3$); ^{13}C NMR (126 MHz, $CDCl_3$) δ 184.1 (C=O), 143.4 (Ar–C_q), 140.9 (Ar–C_q), 136.0 (Ar–C_q), 135.7 (Ar–C_q), 135.6 (2 × Ar–C), 135.6 (2 × Ar–C), 133.6 (Ar–C), 129.5 (2 × Ar–C), 129.3 (2 × Ar–C), 128.9 (2 × Ar–C), 128.7 (2 × Ar–C), 127.9 (2 × Ar–C), 127.3 (2 × Ar–C), 127.2 (2 × Ar–C), 110.1 (Py–CH), 27.1 ($C(\mathit{C}H_3)_3$), 21.5 (Ar–CH_3), 19.4 ($\mathit{C}(CH_3)_3$); HRMS (ESI-TOF) *m/z*: Calcd. for $C_{33}H_{34}N_3O_2SSi$ $[M+H]^+$: 564.2141, found: 564.2141.

((*tert*-Butyldiphenylsilyl)imino)(5-(phenylsulfonyl)-1*H*-pyrazol-3-yl)(*p*-tolyl)-λ^6-sulfanone (16a)

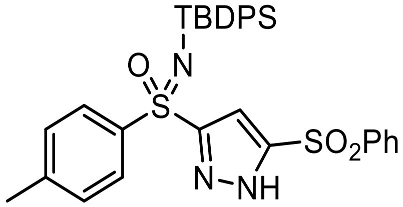

Prepared using general procedure G with α-diazo sulfoximine **8a** (86.7 mg, 0.2 mmol, 1.0 equiv) and alkynyl phenyl sulfone **S16a** (166 mg, 1.0 mmol, 5.0 equiv). Purification by flash column chromatography (SiO_2, 15% to 30% EtOAc in pentane) afforded α-pyrazole sulfoximine **16a** as a colourless foam (102 mg, 85%). R_f 0.15 (30% EtOAc in pentane); IR (film)/cm^{-1} 3068, 2929, 2856, 1592, 1322, 1294, 1151, 1088, 993, 907, 817, 727, 702, 680, 613; ^{1}H NMR (400 MHz, $CDCl_3$) δ 11.26 (s, 1H, NH), 7.92 (d, *J* = 7.3 Hz, 2H, 2 × Ar–H), 7.88 (d, *J* = 8.1 Hz, 2H, 2 × Ar–H), 7.66–7.50 (m, 7H, 7 × Ar–H), 7.31–7.29 (m, 6H, 6 × Ar–H), 7.12 (t, *J* = 7.4 Hz, 2H, 2 × Ar–H), 6.75 (s, 1H, Py–H), 2.41 (s, 3H, Ar–CH_3), 1.09 (s, 9H, $C(CH_3)_3$). ^{13}C NMR (101 MHz, $CDCl_3$) δ 144.3 (Ar–C_q), 140.1 (Ar–C_q), 139.9 (Ar–C_q), 135.6 (2 × Ar–C), 135.4 (2 × Ar–C), 134.7 (Ar–C_q), 134.5 (Ar–C_q), 133.9 (Ar–C), 129.9 (2 × Ar–C), 129.4 (Ar–C), 129.3 (Ar–C), 129.3 (2 × Ar–C), 128.1 (2 × Ar–C), 127.5 (2 × Ar–C), 127.5 (2 × Ar–C), 127.4 (2 × Ar–C), 107.6 (Py–CH), 27.0 ($C(\mathit{C}H_3)_3$), 21.5 (Ar–CH_3), 19.3 ($\mathit{C}(CH_3)_3$); HRMS (ESI-TOF) *m/z*: Calcd. for $C_{33}H_{34}N_3O_2SSi$ $[M+H]^+$: 564.2141, found: 564.2141.

3-(*N*-(*tert*-Butyldiphenylsilyl)-4-methylphenylsulfonimidoyl)-*N*-methyl-*N*-(3-phenyl-3-(4-(trifluoromethyl)phenoxy)propyl)-1*H*-pyrazole-5-carboxamide (17a)

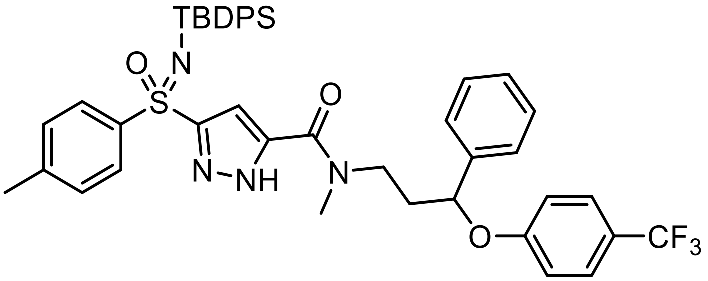

Prepared using general procedure G with α-diazo sulfoximine **8a** (86.7 mg, 0.2 mmol, 1.0 equiv) and alkyne **S17a** (144 mg, 0.4 mmol, 2.0 equiv). Purification by flash column chromatography (SiO_2, 50% EtOAc in pentane) afforded α-pyrazole sulfoximine **17a** as a colourless gum (116 mg, 73%). R_f 0.36 (50% EtOAc in pentane); IR (film)/cm^{-1} 3067, 2930, 2855, 1612 (C=O), 1322, 1245, 1158, 1108, 1066, 1005, 906, 835, 728, 698, 646, 603, 497. Major rotamer: ^{1}H NMR (400 MHz, $CDCl_3$) δ 12.03 (s, 1H, NH), 7.97–7.95 (m, 2H, 2 × Ar–H), 7.76–7.68 (m, 4H, 4 × Ar–H), 7.37–7.16 (m, 15H, 15 × Ar–H), 6.91–6.81 (m, 2H, 2 × Ar–H), 6.60 (s, 1H, Ar–H), 5.25–5.21 (m, 1H, OCH), 3.82–3.57 (m, 2H, NCH_2), 2.99 (d, *J* = 3.8 Hz, 3H, NCH_3), 2.36 (s, 3H, Ar–CH_3), 2.29–2.11 (m, 2H, NCH_2CH_2), 1.12 (s, 9H, $C(CH_3)_3$); ^{13}C NMR (101 MHz, $CDCl_3$) δ 160.0 (C=O), 156.2 (Ar–C_q), 143.0 (Ar–C_q), 141.3 (Ar–C_q), 140.3 (d, *J* = 4.7 Hz, Ar–C_q) 135.9 (d, *J* = 4.7 Hz, 2 × Ar–C_q). 135.6 (4 × Ar–C), 129.4 (2 × Ar–C), 128.8 (4 × Ar–C), 128.0 (Ar–C), 127.7 (2 × Ar–C), 127.2 (4 × Ar–C), 126.7 (2 × Ar–C), 125.6 (2 × Ar–C), 124.2 (q, *J* = 271.2 Hz, CF_3), 122.8 (q, *J* = 32.4 Hz, CCF_3), 115.6 (2 × Ar–C), 107.4 (Ar–C), 78.2 (d, *J* = 4.6 Hz, OCH), 46.4 (NCH_2), 37.0 (NCH_3), 35.9 (NCH_2), 27.1($C(CH_3)_3$), 21.4 (Ar-CH_3), 19.4 ($C(CH_3)_3$); Minor rotamer: ^{1}H NMR (400 MHz, $CDCl_3$) δ 12.03 (s, 1H, NH), 7.97–7.95 (m, 2H, 2 × Ar–H), 7.76–7.68 (m, 4H, 4 × Ar–H), 7.37–7.16 (m, 15H, 15 × Ar–H), 6.91–6.81 (m, 3H, 3 × Ar–H), 5.14–5.10 (m, 1H, OCH), 3.82–3.57 (m, 2H, NCH_2), 3.05 (d, *J* = 4.1 Hz, 3H, NCH_3), 2.36 (s, 3H, Ar–CH_3), 2.29–2.11 (m, 2H, NCH_2CH_2), 1.12 (s, 9H, $C(CH_3)_3$); ^{13}C NMR (101 MHz, $CDCl_3$) δ d 159.7 (C=O), 156.2 (Ar–C_q), 143.0 (Ar–C_q), 141.3 (d, *J* = 21.8 Hz, Ar–C_q), 139.66 (d, *J* = 14.0 Hz, Ar–C_q), 135.7 (2 × Ar–C_q), 135.6 (4 × Ar–C), 129.4 (2 × Ar–C), 129.0 (4 × Ar–C), 128.2 (Ar–C), 127.7 (2 × Ar–C), 127.2 (4 × Ar–C), 126.7 (2 × Ar–C), 125.4 (2 × Ar–C), 124.2 (q, *J* = 271.2 Hz, CF_3), 122.8 (q, *J* = 32.4 Hz, CCF_3), 115.6 (2 × Ar–C), 106.77 (d, *J* = 16.0 Hz, Ar–C), 77.4 (d, *J* = 14.3 Hz, OCH), 47.3 (d, *J* = 31.3 Hz, NCH_2), 37.3 (d, *J* = 23.4 Hz, CH_2), 34.6 (d, *J* = 15.4 Hz, NCH_3), 27.1($C(CH_3)_3$), 21.4 (Ar-CH_3), 19.4 ($C(CH_3)_3$); ^{19}F (377 MHz, $CDCl_3$) δ –61.5. HRMS (ESI-TOF) *m/z*: Calcd. for $C_{44}H_{46}N_4O_3F_3SSi$ $[M+H]^+$: 795.3012, found: 795.3003.

Note: The NMRs are observed as rotamers in a 1.7:1 ratio. The product is assumed to be formed as a 1:1 mixture of diasereoisomers The distance between the stereocenters means there is not a clear distinction by NMR of different diastereoisomers.

3-(*N*-(*tert*-Butyldiphenylsilyl)-4-methylphenylsulfonimidoyl)-*N*-(3-(10,11-dihydro-5*H*-dibenzo[*a*,*d*][7]annulen-5-ylidene)propyl)-*N*-methyl-1*H*-pyrazole-5-carboxamide (18a)

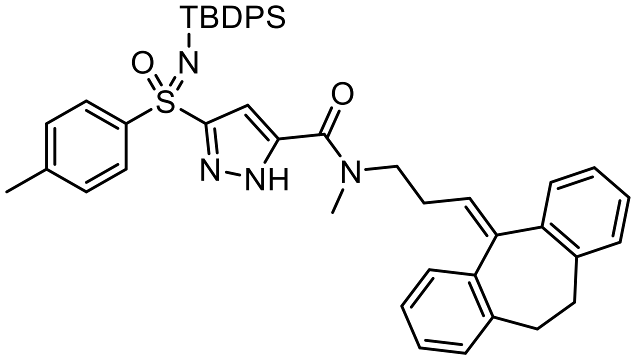

Prepared using general procedure G with α-diazo sulfoximine **8a** (43.3 mg, 0.1 mmol, 1.0 equiv) and propargylic amide **S18a** (63.0 mg, 0.2 mmol, 2.0 equiv). Purification by flash column chromatography (SiO_2, 100% CH_2Cl_2 to 3% EtOAc in CH_2Cl_2) afforded α-pyrazole sulfoximine **18a** as a colourless foam (46.1 mg, 60%). R_f 0.18 (3% EtOAc in CH_2Cl_2); IR (film)/cm^{-1} 3143, 3063, 2927, 2854, 1611 (C=O), 1485, 1323, 1293, 1158, 1105, 1000, 907, 817, 728, 700, 648, 604, 497; ^{1}H NMR (400 MHz, $CDCl_3$, observed as a mixture of 2 rotamers) δ 11.69 (s, 1H, NH), 7.97–7.89 (m, 2H, 2 × Ar–H), 7.76–7.71 (m, 4H, 4 × Ar–H), 7.31–6.99 (m, 16H, 16 × Ar–H), 6.60–6.52 (m, 1H, Ar–H), 5.85–5.64 (m, 1H, CCH), 3.60–3.44 (m, 2H, CH_2), 3.25–3.14 (m, 2H, CH_2), 2.98–2.63 (m, 5H, NCH_3 and CH_2), 2.45–2.35 (m, 5H, Ar–CH_3 and CH_2), 1.12–1.11 (m, 9H, $C(CH_3)_3$); ^{13}C NMR (101 MHz, $CDCl_3$, observed as a mixture of 2 rotamers) δ 159.2 (C=O), 146.0 and 145.1 (C_q), 143.0 (C_q), 141.3 (C_q), 140.7 and 140.4 (C_q), 139.5 (C_q), 139.3 and 139.3 (C_q), 136.9 (C_q), 135.9 and 135.8 (2 × C_q), 135.6–135.5 (m, 4 × Ar–C), 130.1 and 130.0 (Ar–C), 129.4 (2 × Ar–C), 128.8 (2 × Ar–C), 128.5–127.6 (m, 6 × Ar–C), 127.2–126.9 (m, 4 × Ar–C and C*C*H), 126.0 (Ar–C), 125.8 (Ar–C), 107.1 (Py–CH), 50.1 and 40.5 (NCH_2), 36.4 and 34.4 (NCH_3), 33.6 and 31.9 (2 × Ar–CH_2), 28.4 and 27.1 (NCH_2*C*H_2), 27.1 (C(*C*H_3)$_3$), 21.4 (Ar–CH_3), 19.4 (*C*(CH_3)$_3$); HRMS (ESI-TOF) *m/z*: Calcd. for $C_{46}H_{49}N_4O_2SSi$ $[M+H]^+$: 749.3346, found: 749.3368.

4-((*S*)-2-((*tert*-Butoxycarbonyl)amino)-3-methoxy-3-oxopropyl)phenyl 3-(*N*-(*tert*-butyldiphenylsilyl)-4-methylphenylsulfonimidoyl)-1*H*-pyrazole-5-carboxylate (19a)

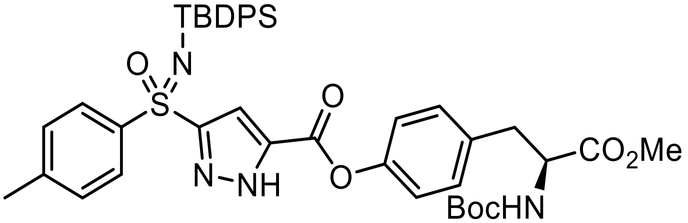

Prepared using general procedure G with α-diazo sulfoximine **8a** (43.3 mg, 0.1 mmol, 1.0 equiv) and propargylic ester **S19a** (69 mg, 0.2 mmol, 2.0 equiv). Purification by flash column chromatography (SiO_2, 40% EtOAc in pentane) afforded α-pyrazole sulfoximine **19a** as a colourless gum (71.4 mg, 91%). R_f 0.33 (40% EtOAc in pentane); IR (film)/cm^{-1} 3069, 2953, 2931, 2855, 1743 (C=O), 1712 (C=O), 1503, 1346, 1293, 1154, 1106, 996, 816, 751, 700, 604, 496; ^{1}H NMR (400 MHz, $CDCl_3$) δ 7.94 (d, *J* = 7.8 Hz, 2H, 2 × Ar–H), 7.74–7.70 (m, 4H, 4 × Ar–H), 7.39–7.16 (m, 10H, 10 × Ar–H), 7.08 (d, *J* = 8.1 Hz, 2H, 2 × Ar–H), 6.98 (s, 1H, Py–H), 5.22–5.09 (m, 1H, NH), 4.64–4.38 (m, 1H,

NCH), 3.71 (s, 3H, CO_2CH_3), 3.16–3.02 (m, 2H, Ar–CH_2), 2.38 (s, 3H, Ar–CH_3), 1.42 (s, 9H, $C(CH_3)_3$), 1.13 (s, 9H, $C(CH_3)_3$); ^{13}C NMR (101 MHz, $CDCl_3$) δ 172.2 (C=O), 157.5 (C=O), 155.1 (C=O), 148.8 (Ar–C_q), 143.4 (Ar–C_q), 141.0 (Ar–C_q), 135.6 (2 × Ar–C), 135.5 (2 × Ar–C), 135.5 (2 × Ar–C_q), 134.3 (Ar–C_q), 130.4 (2 × Ar–C), 129.5 (2 × Ar–C), 129.0 (2 × Ar–C), 127.7 (2 × Ar–C), 127.3 (4 × Ar–C), 121.3 (2 × Ar–C), 109.9 (Py–CH), 80.1 (*C*$(CH_3)_3$), 54.3 (*C*CO_2Me), 52.3 (CO_2*C*H_3), 37.7 (Ar–CH_2), 28.2 (C(*C*$H_3)_3$), 27.1 (C(*C*$H_3)_3$), 21.4 (Ar–CH_3), 19.4 (*C*$(CH_3)_3$); HRMS (ESI-TOF) *m/z*: Calcd. for $C_{42}H_{49}N_4O_7SiS$ $[M+H]^+$: 781.3091, found: 781.3054.

Note: The product is assumed to be formed as a 1:1 mixture of diasereoisomers The distance between the stereocenters means there is not a clear distinction by NMR of different diastereoisomers.

((*tert*-Butyldiphenylsilyl)imino)(5-(4-(2-chlorodibenzo[*b,f*][1,4]oxazepin-11-yl)piperazine-1-carbonyl)-1*H*-pyrazol-3-yl)(*p*-tolyl)-λ^6-sulfanone (20a)

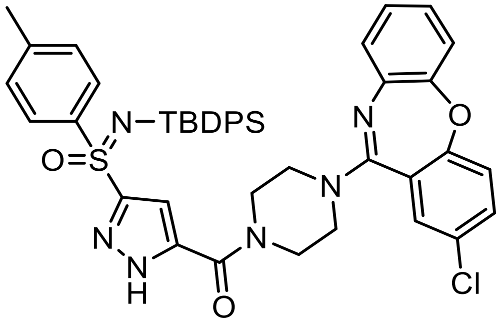

Prepared using general procedure G with α-diazo sulfoximine **8a** (43.3 mg, 0.1 mmol, 1.0 equiv) and propargylic amide **S20a** (73.2 mg, 0.2 mmol, 2.0 equiv). Purification by flash column chromatography (SiO_2, 30% EtOAc in hexane) afforded α-pyrazole sulfoximine **20a** as a white solid (53.2 mg, 67%). R_f 0.16 (30% EtOAc in pentane); Mp: 151–154 °C; IR (film)/cm^{-1} 3066, 2928, 2854, 1591 (C=O), 1558, 1467, 1286, 1156, 1105, 899, 821, 701, 604, 495; ^{1}H NMR (400 MHz, $CDCl_3$) δ 12.20 (s, 1H, NH), 7.96 (d, *J* = 7.9 Hz, 2H, 2 × Ar–H), 7.73 (d, *J* = 6.9 Hz, 4H, 4 × Ar–H), 7.47 (dd, *J* = 8.8, 2.5 Hz, 1H, Ar–H), 7.35–7.20 (m, 11H, 11 × Ar–H), 7.16–7.12 (m, 2H, 2 × Ar–H), 7.08–7.04 (m, 1H, Ar–H), 6.56 (s, 1H, Py–H), 3.80–3.47 (m, 8H, 4 × CH_2), 2.37 (s, 3H, Ar–CH_3), 1.11 (s, 9H, $C(CH_3)_3$); ^{13}C NMR (101 MHz, $CDCl_3$) δ 159.4 (Ar–C_q), 158.4 (C=N), 151.7 (C=O), 143.2 (Ar–C_q), 141.0 (Ar–C_q), 135.7 (Ar–C_q), 135.7 (Ar–C_q), 135.6 (2 × Ar–C), 135.5 (2 × Ar–C), 133.0 (Ar–C), 130.5 (Ar–C_q), 129.5 (2 × Ar–C), 128.9 (2 × Ar–C), 128.8 (Ar–C), 127.7 (2 × Ar–C), 127.3 (2 × Ar–C), 127.3 (2 × Ar–C), 127.1 (Ar–C), 125.9 (Ar–C), 125.2 (Ar–C), 124.5 (Ar–C_q), 122.9 (Ar–C), 120.2 (Ar–C), 107.3 (Py–CH), 46.2 (2 × CH_2), 42.2 (2 × CH_2), 27.0 (C(*C*$H_3)_3$), 21.4 (Ar–CH_3), 19.4 (*C*$(CH_3)_3$); HRMS (ESI-TOF) *m/z*: Calcd. for $C_{44}H_{44}N_6O_3SiSCl$ $[M+H]^+$: 799.2653, found: 799.2643.

Dimethyl (*E*)-3-(*N*-(*tert*-butyldiphenylsilyl)-4-methylphenylsulfonimidoyl)-1-(1,4-dimethoxy-1,4-dioxobut-2-en-2-yl)-1*H*-pyrazole-4,5-dicarboxylate (21)

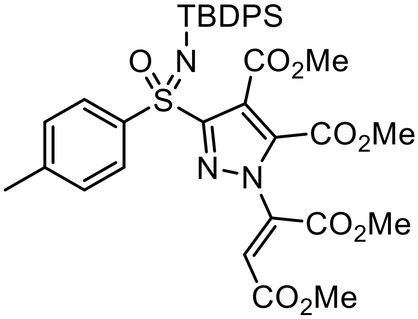

Dimethyl acetylenedicarboxylate (123 □L, 1.0 mmol, 5.0 equiv) was added to a stirred solution of α-diazo sulfoximine **8a** (86.7 mg, 0.2 mmol, 1.0 equiv) in MeCN (1.0 mL). The reaction mixture was stirred at rt for 2 h. The reaction mixture was concentrated under reduced pressure and purified by flash column chromatography to afford the pyrazole alkyne adduct **21** as a colourless gum (106.0 mg, 74%, 52:48 *dr*). R_f 0.4 (40% EtOAc in pentane); IR (film)/cm^{-1} 3019, 2952, 2889, 2855, 1731 (C=O), 1437, 1344, 1234, 1165, 1105, 1072, 753, 701.

Diastereomer A: ^{1}H NMR (400 MHz, $CDCl_3$) δ□8.03 (d, *J* = 8.1 Hz, 2H, 2 × Ar–H), 7.88–7.82 (m, 2H, 2 × Ar–H), 7.72–7.66 (m, 2H, 2 × Ar–H), 7.10 (s, 1H, HC=C), 3.81 (s, 3H, CO_2CH_3), 3.80 (s, 3H, CO_2CH_3), 3.77 (s, 3H, CO_2CH_3), 3.24 (s, 3H, CO_2CH_3), 2.36 (s, 3H, Ar–CH_3), 1.09 (s, 9H, $C(CH_3)_3$); ^{13}C NMR (101 MHz, $CDCl_3$) δ□162.1 (C=O), 161.8 (C=O), 161.3 (C=O), 157.7 (C=O), 154.2 (Ar–C_q), 143.0 (Ar–C_q), 141.3 (Ar–C_q), 137.6 (Ar–C_q), 135.6 (Ar–C_q), 135.4 (4 × Ar–C), 133.2 (Ar–C_q), 129.1 (2 × Ar–C), 128.7 (2 × Ar–C), 127.9 (H*C*=C), 127.8 (2 × Ar–C), 127.1 (4 × Ar–C), 118.5 (HC=*C*), 53.4 ($CO_2\mathit{C}H_3$), 52.9 ($CO_2\mathit{C}H_3$), 52.8 ($CO_2\mathit{C}H_3$), 52.0 ($CO_2\mathit{C}H_3$), 26.9 ($C(\mathit{C}H_3)_3$), 21.3 (Ar–CH_3), 19.4 ($\mathit{C}(CH_3)_3$).

Diastereomer B: ^{1}H NMR (400 MHz, $CDCl_3$) δ□δ 7.91 (d, *J* = 8.1 Hz, 2H, 2 × Ar–H), 7.81–7.77 (m, 2H, 2 × Ar–H), 7.77–7.73 (m, 2H, 2 × Ar–H), 6.06 (s, 1H, HC=C), 3.85 (s, 3H, CO_2CH_3), 3.85 (s, 3H, CO_2CH_3), 3.80 (s, 3H, CO_2CH_3), 3.70 (s, 3H, CO_2CH_3), 2.40 (s, 3H, Ar–CH_3), 1.14 (s, 9H, $C(CH_3)_3$); ^{13}C NMR (101 MHz, $CDCl_3$) δ□163.6 (C=O), 162.0 (C=O), 160.5 (C=O), 157.7 (C=O), 154.2 (Ar–C_q), 143.5 (Ar–C_q), 140.3 (Ar–C_q), 135.6 (Ar–C_q), 135.5 (4 × Ar–C), 135.3 (Ar–C_q), 132.7 (Ar–C_q), 129.4 (2 × Ar–C), 128.8 (Ar–C), 128.8 (Ar–C), 128.1 (2 × Ar–C), 127.6 (H*C*=C), 127.2 (2 × Ar–C), 127.1 (2 × Ar–C), 119.4 (HC=*C*), 53.0 ($CO_2\mathit{C}H_3$), 52.9 ($CO_2\mathit{C}H_3$), 52.9 ($CO_2\mathit{C}H_3$), 52.5 ($CO_2\mathit{C}H_3$), 26.9 ($C(\mathit{C}H_3)_3$), 21.4 (Ar–CH_3), 19.3($\mathit{C}(CH_3)_3$).□

HRMS (ESI-TOF) *m/z*: Calcd. for $C_{44}H_{44}N_6O_3SiSCl$ $[M+H]^+$: 718.2255, found: 718.2255.

General Procedure H for Cycloaddition between α-diazo sulfoximines and di-substituted alkynes

Alkyne (0.2 mmol, 1.0 equiv) was added to a stirred solution of a-diazo sulfoximine (0.24 mmol, 1.2 equiv) in anhydrous THF (1.0 mL, 0.2 M) at rt. The resulting mixture was stirred at rt until TLC analysis showed the complete consumption of the alkyne. The reaction mixture was concentrated under reduced pressure followed by purification by flash column chromatography (SiO_2) to afford the corresponding pyrazolesulfoximine.

Dimethyl 3-(*N*-(*tert*-butyldiphenylsilyl)-4-methylphenylsulfonimidoyl)-1*H*-pyrazole-4,5-dicarboxylate (22a)

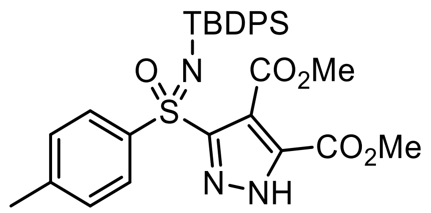

Prepared using general procedure H with α-diazo sulfoximine **8a** (104 mg, 0.24 mmol, 1.2 equiv) and dimethyl acetylenedicarboxylate (24.6 □L, 0.2 mmol, 1.0 equiv). Purification by flash column chromatography (SiO_2, 30% Et_2O and 40% CH_2Cl_2 in hexane) afforded α-pyrazole sulfoximine **22a** as a colourless foam (88.2 mg, 77%). R_f 0.33 (30% Et_2O and 40% CH_2Cl_2 in hexane); IR (film)/cm^{-1} 3069, 2953, 2929, 2855, 1733 (C=O), 1562, 1593, 1430, 1339, 1293, 1157, 1089, 1010, 81, 701, 657, 604; ^{1}H NMR (400 MHz, $CDCl_3$) δ□11.56 (s, 1H, NH), 7.93 (d, *J* = 8.2 Hz, 2H, 2 × Ar–H), 7.69–7.67 (m, 4H, 4 × Ar–H), 7.33–7.22 (m, 8H, 8 × Ar–H), 3.83 (s, 3H, CO_2CH_3), 3.69 (s, 3H, CO_2CH_3), 2.38 (s, 3H, Ar–CH_3), 1.09 (s, 9H, $C(CH_3)_3$); ^{13}C NMR (101 MHz, $CDCl_3$) δ□162.0 (C=O), 159.3 (C=O), 143.7 (Ar–C_q), 140.4 (Ar–C_q), 135.5 (4 × Ar–C), 135.0 (Ar–C_q), 135.0 (Ar–C_q), 129.4 (2 × Ar–C), 129.0 (2 × Ar–C), 128.1 (2 × Ar–C), 127.3 (4 × Ar–C), 115.8 (Py–C_q), 52.8 ($CO_2\mathit{C}H_3$), 52.6 ($CO_2\mathit{C}H_3$), 27.0 ($C(\mathit{C}H_3)_3$), 21.5 (Ar–CH_3), 19.3 ($\mathit{C}(CH_3)_3$); HRMS (ESI-TOF) *m/z*: Calcd. for $C_{30}H_{34}N_3O_5SSi$ [M+H]$^+$: 576.1983, found: 576.1996.

Dimethyl 3-(*N*-(*tert*-butyldiphenylsilyl)-4-methoxyphenylsulfonimidoyl)-1*H*-pyrazole-4,5-dicarboxylate (22b)

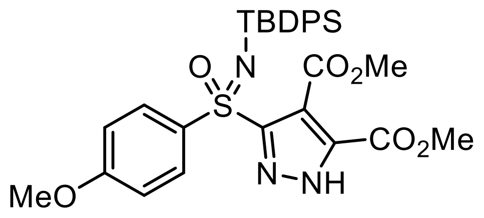

Prepared using general procedure H with α-diazo sulfoximine **8b** (135 mg, 0.36 mmol, 1.2 equiv) and dimethyl acetylenedicarboxylate (36.9 □L, 0.3 mmol, 1.0 equiv). Purification by flash column chromatography (SiO_2, 20% Et_2O and 30% CH_2Cl_2 in hexane) afforded α-pyrazole sulfoximine **22b** as a colourless foam (133 mg, 75%). R_f 0.2 (20% Et_2O and 30% CH_2Cl_2 in hexane); IR (film)/cm^{-1} 3071, 2953, 2890, 2855, 1733 (C=O), 1592, 1494, 1433, 1357, 1290, 1258, 1155, 1091, 1009, 804, 702, 661, 605, 494; ^{1}H NMR (400 MHz, $CDCl_3$) δ□10.91 (s, 1H, NH), 7.98–7.96 (m, 2H, 2 × Ar–H), 7.67 (d, *J* = 7.1 Hz, 4H, 4 × Ar–H), 7.35–7.24 (m, 6H, 6 × Ar–H), 6.93–6.91 (m, 2H, 2 × Ar–H), 3.86 (s, 3H, CO_2CH_3), 3.84 (s, 3H, CO_2CH_3) 3.70 (s, 3H, OCH_3), 1.09 (s, 9H, $C(CH_3)_3$); ^{13}C NMR (101 MHz, $CDCl_3$) δ□163.2 (C=O), 161.9 (Ar–C_q), 159.4 (C=O), 135.5 (4 × Ar–C), 135.1 (Ar–C_q), 135.0 (Ar–C_q), 134.8 (Ar–C_q), 130.4 (2 × Ar–C), 129.1 (Ar–C), 129.1 (Ar–C), 127.3 (4 × Ar–C), 115.4 (Py–C_q), 114.0 (2 × Ar–C), 55.6 (OCH_3), 52.8 ($CO_2\mathit{C}H_3$), 52.6 ($CO_2\mathit{C}H_3$), 27.0 ($C(\mathit{C}H_3)_3$), 19.4 ($\mathit{C}(CH_3)_3$); HRMS (ESI-TOF) *m/z*: Calcd. for $C_{32}H_{30}N_5O_3SiS$ [M+H]$^+$: 592.1839, found: 592.1862.

Dimethyl (*R*)-3-(4-bromo-*N*-(*tert*-butyldiphenylsilyl)phenylsulfonimidoyl)-1*H*-pyrazole-4,5-dicarboxylate ((*R*)-22d)

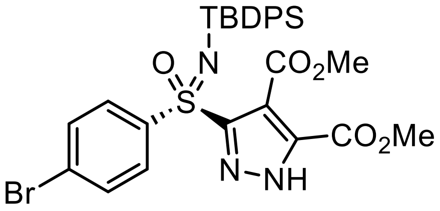

Prepared using general procedure H with α-diazo sulfoximine **(*R*)-8d** (120 mg, 0.24 mmol, 1.2 equiv) and dimethyl acetylenedicarboxylate (24.6 □L, 0.2 mmol, 1.0 equiv). Purification by flash column chromatography (SiO_2, 10% Et_2O and 30% CH_2Cl_2 in hexane to 20% Et_2O and 30% CH_2Cl_2 in hexane) afforded α-pyrazole sulfoximine **(*R*)-22d** as a colourless foam (87.8 mg, 69%, 97% *ee*). R_f 0.25 (20% Et_2O and 30% CH_2Cl_2 in hexane); IR (film)/cm^{-1} 3070, 2953, 2931, 2856, 1731 (C=O), 1568, 1357, 1309, 1156, 1089, 1008, 909, 821, 727, 700, 498; ^1H NMR (400 MHz, $CDCl_3$) δ□12.03 (s, 1H, NH), 7.89 (d, *J* = 8.5 Hz, 2H, 2 × Ar–H), 7.67 (d, *J* = 6.9 Hz, 4H, 4 × Ar–H), 7.54 (d, *J* = 8.6 Hz, 2H, 2 × Ar–H), 7.33–7.23 (m, 6H, 6 × Ar–H), 3.84 (s, 3H, CO_2CH_3), 3.73 (s, 3H, CO_2CH_3), 1.09 (s, 9H, $C(CH_3)_3$); ^{13}C NMR (101 MHz, $CDCl_3$) δ□162.1 (C=O), 158.8 (C=O), 142.4 (Ar–C_q), 135.4 (4 × Ar–C), 134.8 (Ar–C_q), 134.7 (Ar–C_q), 131.9 (2 × Ar–C), 129.5 (2 × Ar–C), 129.0 (2 × Ar–C), 127.8 (Ar–C_q), 127.2 (4 × Ar–C), 116.3 (Py-C_q), 52.8 (CO_2CH_3), 52.8 (CO_2CH_3), 26.9 ($C(CH_3)_3$), 19.3 ($C(CH_3)_3$); HRMS (ESI-TOF) *m/z*: Calcd. for $C_{29}H_{31}N_3O_5SiSBr$ $[M+H]^+$: 640.0937, found: 640.0941; $[\alpha]^{22}_D$ = +2 (c 1.0, $CHCl_3$).

Methyl 4-bromo-3-(*N*-(*tert*-butyldiphenylsilyl)-4-methylphenylsulfonimidoyl)-1*H*-pyrazole-5-carboxylate (23a)

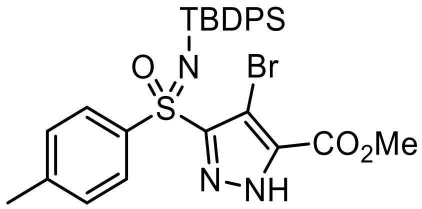

Prepared using general procedure G with α-diazo sulfoximine **8a** (86.7 mg, 0.2 mmol, 1.0 equiv) and methyl 3-bromopropiolate **S23a** (163 mg, 1.0 mmol, 5.0 equiv). Purification by flash column chromatography (SiO_2, 20% to 40% EtOAc in hexane) afforded α-pyrazole sulfoximine **23a** as a colourless foam (59.1 mg, 50%). R_f 0.11 (30% EtOAc in hexane); IR (film)/cm^{-1} 3067, 2954, 2928, 2855, 1723 (C=O), 1320, 1295, 1154, 1107, 984, 907, 813, 727, 699, 605, 496; ^1H NMR (400 MHz, $CDCl_3$) δ□11.40 (s, 1H, NH), 7.95 (d, *J* = 8.2 Hz, 2H, 2 × Ar–H), 7.69 (ddd, *J* = 8.0, 4.2, 1.6 Hz, 4H, 4 × Ar–H), 7.33–7.22 (m, 8H, 8 × Ar–H), 3.87 (s, 3H, CO_2CH_3), 2.41 (s, 3H, Ar–CH_3), 1.14 (s, 9H, $C(CH_3)_3$); ^{13}C NMR (101 MHz, $CDCl_3$) δ□159.6 (C=O), 144.0 (Ar–C_q), 139.4 (Ar–C_q), 135.6 (2 × Ar–C), 135.6 (2 × Ar–C), 134.8 (Ar–C_q), 134.7 (Ar–C_q), 129.5 (2 × Ar–C), 129.1 (Ar–C), 129.1 (Ar–C), 128.3 (2 × Ar–C), 127.2 (4 × Ar–C), 97.1 (CBr), 52.4 (CO_2CH_3), 27.1 ($C(CH_3)_3$), 21.5 (Ar–CH_3), 19.4 ($C(CH_3)_3$); HRMS (ESI-TOF) *m/z*: Calcd. for $C_{28}H_{31}N_3O_3SiSBr$ $[M+H]^+$: 596.1039, found: 596.1021.

Methyl 3-(*N*-(*tert*-butyldiphenylsilyl)-4-methylphenylsulfonimidoyl)-4-iodo-1*H*-pyrazole-5-carboxylate (24a)

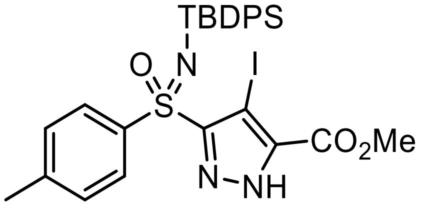

Prepared using general procedure G with α-diazo sulfoximine **8a** (86.7 mg, 0.2 mmol, 1.0 equiv) and methyl 3-bromopropiolate **S24a** (210 mg, 1.0 mmol, 5.0 equiv). Purification by flash column chromatography (SiO_2, 15% to 30% EtOAc in hexane) afforded α-pyrazole sulfoximine **24a** as a colourless foam (22.1 mg, 14%); R_f 0.22 (30% EtOAc in hexane); IR (film)/cm^{-1} 3067, 2953, 2854, 1723 (C=O), 1320, 1294, 1155, 1106, 1043, 978, 812, 701, 605, 475; ^{1}H NMR (400 MHz, $CDCl_3$) δ 11.37 (s, 1H, NH), 7.93 (d, *J* = 8.4 Hz, 2H, 2 × Ar–H), 7.69 (ddd, *J* = 7.4, 5.7, 1.6 Hz, 4H, 4 × Ar–H), 7.33–7.22 (m, 8H, 8 × Ar–H), 3.88 (s, 3H, CO_2CH_3), 2.41 (s, 3H, Ar–CH_3), 1.13 (s, 9H, $C(CH_3)_3$); ^{13}C NMR (101 MHz, $CDCl_3$) δ 159.9 (C=O), 144.0 (Ar–C_q), 139.3 (Ar–C_q), 135.6 (4 × Ar–C), 134.8 (Ar–C_q), 134.7 (Ar–C_q), 129.4 (2 × Ar–C), 129.1 (Ar–C), 129.1 (Ar–C), 128.4 (2 × Ar–C), 127.2 (4 × Ar–C), 62.3 (CI), 52.4 (CO_2CH_3), 27.2 ($C(CH_3)_3$), 21.6 (Ar–CH_3), 19.5 ($C(CH_3)_3$); HRMS (ESI-TOF) *m/z*: Calcd. for $C_{28}H_{31}N_3O_3SiSI$ $[M+H]^+$: 644.0900, found: 644.0903.

Ethyl 3-(*N*-(*tert*-butyldiphenylsilyl)-4-methylphenylsulfonimidoyl)-4-(piperidine-1-carbonyl)-1*H*-pyrazole-5-carboxylate (25a)

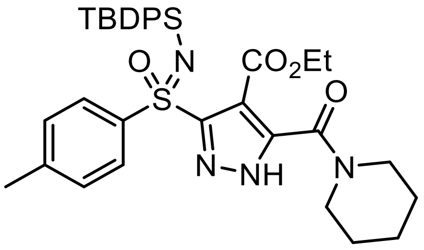

Prepared using general procedure H with α-diazo sulfoximine **8a** (104 mg, 0.24 mmol, 1.2 equiv) and alkyne **S25a** (41.8 mg 0.2 mmol, 1.0 equiv). Purification by flash column chromatography (SiO_2, 30% CH_2Cl_2 and 30% Et_2O in hexane then 50% EtOAc in hexane) afforded α-pyrazole sulfoximine **25a** as a colourless gum (55.8 mg, 43%). R_f 0.05 (30% CH_2Cl_2 and 30% Et_2O in hexane); IR (film)/cm^{-1} 3070, 2931, 2855, 1731 (C=O), 1625 (C=O), 1469, 1327, 1292, 1215, 1159, 1099, 1018, 908, 816, 728, 700, 649, 488; ^{1}H NMR (400 MHz, $CDCl_3$) δ 11.23 (s, 1H, NH), 7.92 (d, *J* = 8.0 Hz, 2H, 2 × Ar–H), 7.69–7.64 (m, 4H, 4 × Ar–H), 7.34-7.24 (m, 8H, 8 × Ar–H), 4.10–3.98 (m, 2H, CH_2CH_3), 3.64 (d, *J* = 5.6 Hz, 2H, 2 × NC*H*H), 3.00 (d, *J* = 5.6 Hz, 2H, 2 × NCH*H*), 2.41 (s, 3H, Ar–CH_3), 1.63–1.60 (m, 4H, 2 × CH_2), 1.45 (d, *J* = 7.6 Hz, 2H, CH_2), 1.11–1.07 (m, 3H, CH_2CH_3), 1.08 (s, 9H, $C(CH_3)_3$). ^{13}C NMR (101 MHz, $CDCl_3$) δ 161.2 (C=O), 159.8 (C=O), 143.8 (Ar–C_q), 139.6 (Ar–C_q), 135.5 (2 × Ar–C), 135.5 (2 × Ar–C), 135.1 (Ar–C_q), 134.9 (Ar–C_q), 129.1 (4 × Ar–C), 128.4 (2 × Ar–C), 127.3 (4 × Ar–C), 110.4 (Py–C_q), 60.9 (CH_2CH_3), 47.9 (NCH_2), 42.6 (NCH_2), 27.0 ($C(CH_3)_3$), 26.0 (CH_2), 25.2 (CH_2), 24.4 (CH_2), 19.4 ($C(CH_3)_3$), 14.0 (CH_2CH_3); HRMS (ESI-TOF) *m/z*: Calcd. for $C_{35}H_{43}N_4O_4SSi$ $[M+H]^+$: 643.2774, found: 643.2797.

Derivatisation of pyrazolesulfoximines

Ester reduction

((*tert*-Butyldiphenylsilyl)imino)(5-(hydroxymethyl)-1*H*-pyrazol-3-yl)(*p*-tolyl)-λ^6-sulfanone (26)

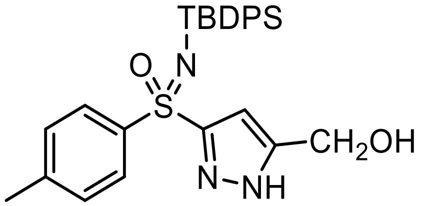

$LiBH_4$ (11.9 mg, 0.5 mmol, 5.0 equiv) was added to a stirred solution of α-pyrazole sulfoximine **9a** (51.8 mg, 0.1 mmol, 1.0 equiv) in THF (0.2 mL). The resulting mixture was stirred at 65 °C for 15 min. The reaction mixture was quenched with MeOH (5 mL), diluted with H_2O (5 mL) and extracted with CH_2Cl_2 (3 × 5 mL). The combined organic layer was dried over Na_2SO_4, concentrated under reduced pressure. Purification by flash column chromatography (SiO_2, 50% EtOAc in pentane) afforded alcohol **26** as a colourless oil (39.6 mg, 81%). R_f 0.43 (60% EtOAc in pentane). IR (film)/cm^{-1} 3069, 2956, 2930, 2855, 1593, 1467, 1426, 1322, 1293, 1149, 1105, 1005, 816, 731, 700, 603, 498; ^{1}H NMR (400 MHz, $CDCl_3$) δ 7.79 (d, *J* = 8.0 Hz, 2H, 2 × Ar–H), 7.65 (ddd, *J* = 7.8, 5.7, 1.7 Hz, 4H, 4 × Ar–H), 7.25–7.18 (m, 6H, 6 × Ar–H), 7.13 (d, *J* = 7.9 Hz, 2H, 2 × Ar–H), 6.23 (s, 1H, Py–H), 4.53–4.45 (m, 2H, CH_2), 2.33 (s, 3H, Ar–CH_3), 1.09 (s, 9H, $C(CH_3)_3$); ^{13}C NMR (101 MHz, $CDCl_3$) δ 142.9 (Ar–C_q), 141.7 (Ar–C_q), 135.7 (Ar–C_q), 135.7 (Ar–C_q), 135.5 (2 × Ar–C), 135.5 (2 × Ar–C), 129.4 (2 × Ar–C), 128.9 (2 × Ar–C), 127.2 (4 × Ar–C), 127.2 (2 × Ar–C), 104.2 (Py–CH), 55.6 (CH_2), 27.1 ($C(\mathit{C}H_3)_3$), 21.4 (Ar–CH_3), 19.4 ($\mathit{C}(CH_3)_3$); HRMS (ESI-TOF) *m/z*: Calcd. for $C_{27}H_{32}N_3O_2SiS$ $[M+H]^+$: 490.1985, found: 490.1977.

Ester hydrolysis

3-(*N*-(*tert*-Butyldiphenylsilyl)-4-methylphenylsulfonimidoyl)-1*H*-pyrazole-5-carboxylic acid (27)

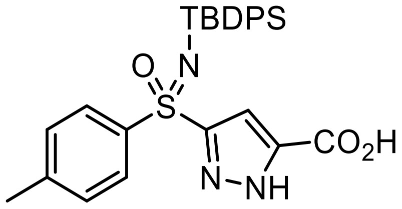

1 M aqueous NaOH solution was added to a stirred solution of α-pyrazole sulfoximine **9a** (51.8 mg, 0.1 mmol, 1.0 equiv) in THF/MeOH (1:1, 1 mL) at rt. The resulting mixture was stirred at rt for 18 h. The reaction mixture was concentrated under reduced pressure and 1 M HCl was added to adjust the pH to around 3. The mixture was extracted with EtOAc (3 × 5 mL) and the combined organic layer was dried over Na_2SO_4, concentrated under reduced pressure. Purification by flash column chromatography (SiO_2, 5% MeOH in CH_2Cl_2) afforded carboxylic acid **27** as a colourless foam (44.3 mg, 92%). R_f 0.3 (5% MeOH in CH_2Cl_2), IR (film)/cm^{-1} 3069, 2928, 2854, 1706 (C=O), 1465, 1426, 1156, 1104, 1080, 1035, 840, 815, 700; ^{1}H NMR (400 MHz, CD_3OD) δ 7.82–7.80 (m, 2H, 2 × Ar–

H), 7.71–7.67 (m, 4H, 4 × Ar–H), 7.32–7.21 (m, 8H, 8 × Ar–H), 6.92 (s, 1H, Py–H), 2.35 (s, 3H, Ar–CH_3), 1.06 (s, 9H, $C(CH_3)_3$); ^{13}C NMR (101 MHz, CD_3OD) δ□144.4 (Ar–C_q), 143.4 (Ar–C_q), 137.2 (2 × Ar–C_q), 136.7 (4 × Ar–C), 130.4 (2 × Ar–C), 130.0 (2 × Ar–C), 128.5 (2 × Ar–C), 127.7 (4 × Ar–C), 108.5 (Py–CH), 27.7 (C(CH$_3$)$_3$), 21.4 (Ar–CH_3), 20.3 (C(CH_3)$_3$); HRMS (ESI-TOF) *m/z*: Calcd. for $C_{27}H_{30}N_3O_3SSi$ $[M+H]^+$: 504.1777, found: 504.1763.

TBDPS Deprotection

Methyl 3-(4-methylphenylsulfonimidoyl)-1*H*-pyrazole-5-carboxylate (28)

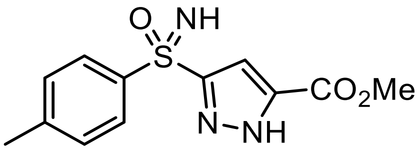

TBAF (1.0 M in THF, 0.3 mL, 1.5 equiv) was added to a stirred solution of α-pyrazole sulfoximine **9a** (104 mg, 0.2 mmol, 1.0 equiv) in THF (1.0 mL). The resulting mixture was stirred at 70 °C for 24 h. The reaction mixture was diluted with brine solution (5 mL) and extracted with EtOAc (3 × 5 mL). The combined organic layer was dried over Na_2SO_4 and concentrated under reduced pressure. Purification by flash column chromatography (SiO_2, 60% EtOAc in hexane) afforded α-pyrazole NH-sulfoximine **28** as a white solid (52.8 mg, 95%). R_f 0.1 (60% EtOAc in hexane); Mp: 181–184 C; IR (film)/cm^{-1} 3293, 3266, 3135, 3014, 2954, 2729, 1721 (C=O), 1437, 1412, 1350, 1305, 1252, 1225, 1175, 1028, 1007, 824, 683, 649, 593; ^{1}H NMR (400 MHz, DMSO-d$_6$) δ□14.7 (s, 1H, NH), 7.88 (d, *J* = 8.2 Hz, 2H, 2 × Ar–H), 7.36 (d, *J* = 8.0 Hz, 2H, 2 × Ar–H) 7.14 (s, 1H, Py–H), 5.19 (s, 1H, NH), 3.82 (s, 3H, CO_2CH_3), 2.35 (s, 3H, Ar–CH_3); ^{13}C NMR (101 MHz, DMSO-d$_6$) δ□159.3 (C=O), 143.2 (Ar–C_q), 139.7 (Ar–C_q), 129.6 (2 × Ar–C), 127.9 (2 × Ar–C), 109.4 (Py–CH), 52.3 ($CO_2$$CH_3$), 20.9 (Ar–$CH_3$); HRMS (ESI-TOF) *m/z*: Calcd. for $C_{12}H_{14}N_3O_3S$ $[M+H]^+$: 280.0756, found: 280.0750.

Benzylation

Methyl 1-benzyl-3-(*N*-(*tert*-butyldiphenylsilyl)-4-methylphenylsulfonimidoyl)-1*H*-pyrazole-5-carboxylate (29)

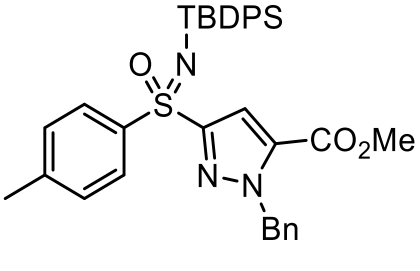

NaH (60% in mineral oil, 9.6 mg, 0.24 mmol, 1.2 equiv) was added to a stirred solution of α-pyrazole sulfoximine **9a** (104 mg, 0.2 mmol, 1.0 equiv) in THF (1.0 mL, 0.2 M) at 0 °C. The resulting mixture was stirred at 0 °C for 1 h. Benzyl bromide (29 □L, 0.24 mmol, 1.2 equiv) was added and the resulting mixture was slowly warmed to rt for 96 h. The reaction mixture was quenched with H_2O (3 mL) and extracted with EtOAc (3 × 5 mL). The combined organic layer was dried over Na_2SO_4 and concentrated under reduced pressure. Purification by flash column chromatography (SiO_2, 20% EtOAc in hexane) afforded *N*-benzyl pyrazole sulfoximine **29** as a colourless gum (72.3 mg, 59%, 92:8 *r.r.*). R_f 0.3 (20% EtOAc in hexane); IR (film)/cm^{-1} 3066, 2954, 2854, 1729 (C=O), 1594, 1319, 1253,

1164, 1101, 818, 726, 700; ^{1}H NMR (400 MHz, $CDCl_3$) δ 7.96–7.94 (m, 2H, 2 × Ar–H), 7.78–7.72 (m, 4H, 4 × Ar–H), 7.34–7.14 (m, 13H, 13 × Ar–H), 6.92 (s, 1H, Py–H), 5.67 (d, *J* = 14.4 Hz, 1H, C*H*H), 5.56 (d, *J* = 14.4 Hz, 1H, CH*H*), 3.80 (s, 3H, CO_2CH_3), 2.40 (s, 3H, Ar–CH_3), 1.13 (s, 9H, $C(CH_3)_3$); ^{13}C NMR (101 MHz, $CDCl_3$) 159.1 (C=O), 154.9 (Ar–C_q), 142.8 (Ar–C_q), 141.7 (Ar–C_q), 136.0 (Ar–C_q), 135.9 (Ar–C_q), 135.6 (2 × Ar–C), 135.6 (2 × Ar–C), 129.3 (2 × Ar–C), 128.7 (Ar–C), 128.7 (Ar–C), 128.4 (2 × Ar–C), 127.9 (3 × Ar–C), 127.7 (2 × Ar–C), 127.2 (2 × Ar–C), 127.1 (2 × Ar–C), 112.0 (Py–CH), 55.6 (CH_2), 52.1 ($CO_2\mathit{C}H_3$), 27.1 ($C(\mathit{C}H_3)_3$), 21.4 (Ar–CH_3), 19.4 ($\mathit{C}(CH_3)_3$); HRMS (ESI-TOF) *m/z*: Calcd. for $C_{35}H_{38}N_3O_3SSi$ $[M+H]^+$: 608.2403, found: 608.2401.

^{1}H and ^{13}C Spectra of Selected Compounds

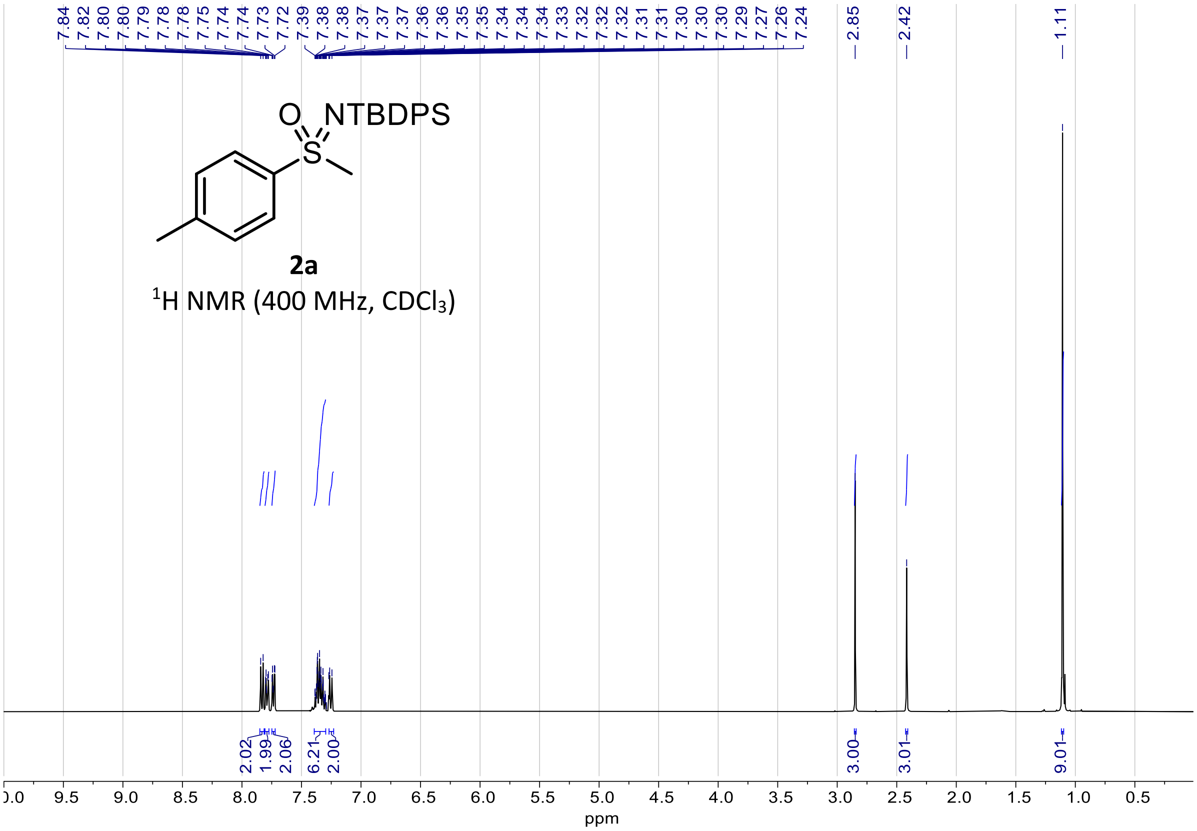
7.84 7.82 7.80 7.80 7.79 7.78 7.75 7.74 7.74 7.73 7.72 7.39 7.38 7.38 7.37 7.37 7.37 7.36 7.36 7.35 7.35 7.34 7.34 7.34 7.33 7.32 7.32 7.32 7.31 7.31 7.30 7.30 7.30 7.29 7.27 7.26 7.24
2.85
2.42
1.11
O NTBDPS
S
2a
^{1}H NMR (400 MHz, CDCl$_3$)
2.02 1.99 2.06 6.21 2.00 3.00 3.01 9.01
ppm

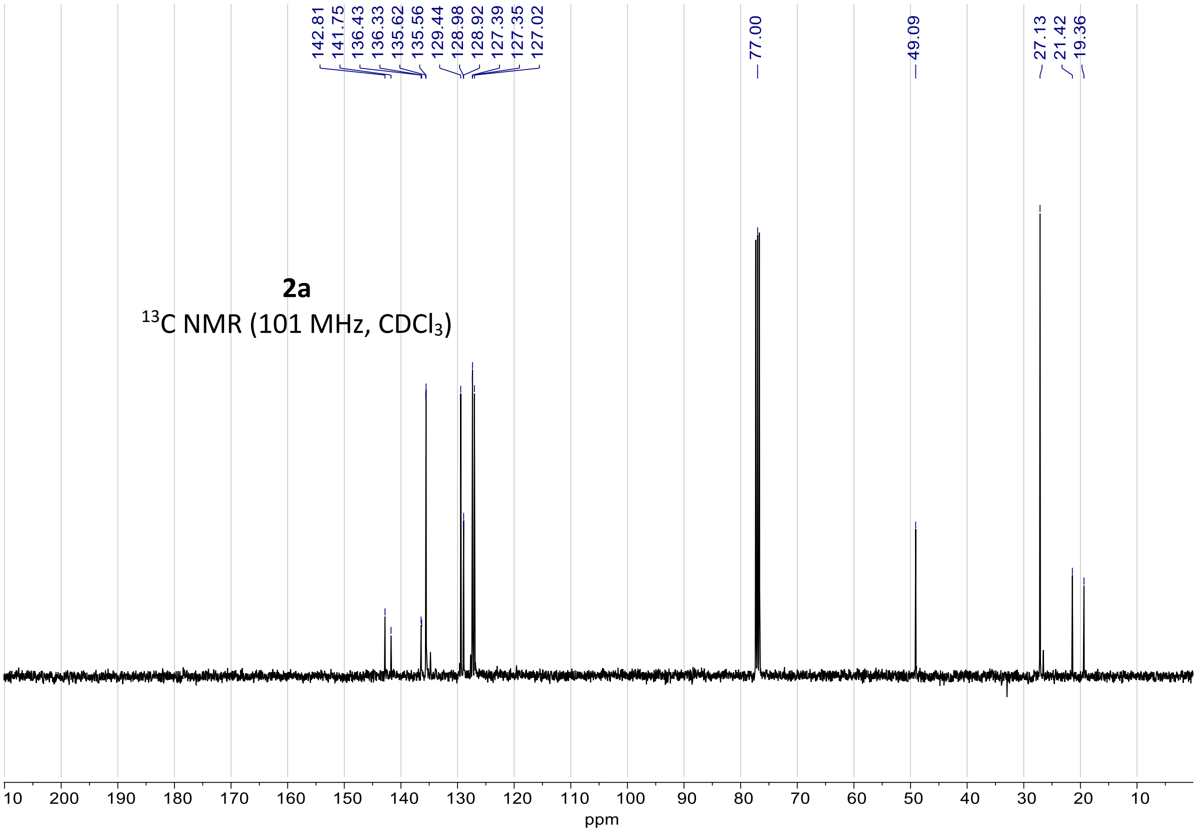
142.81 141.75 136.43 136.33 135.62 135.56 129.44 128.98 128.92 127.39 127.35 127.02
77.00
49.09
27.13 21.42 19.36
2a
^{13}C NMR (101 MHz, CDCl$_3$)
ppm

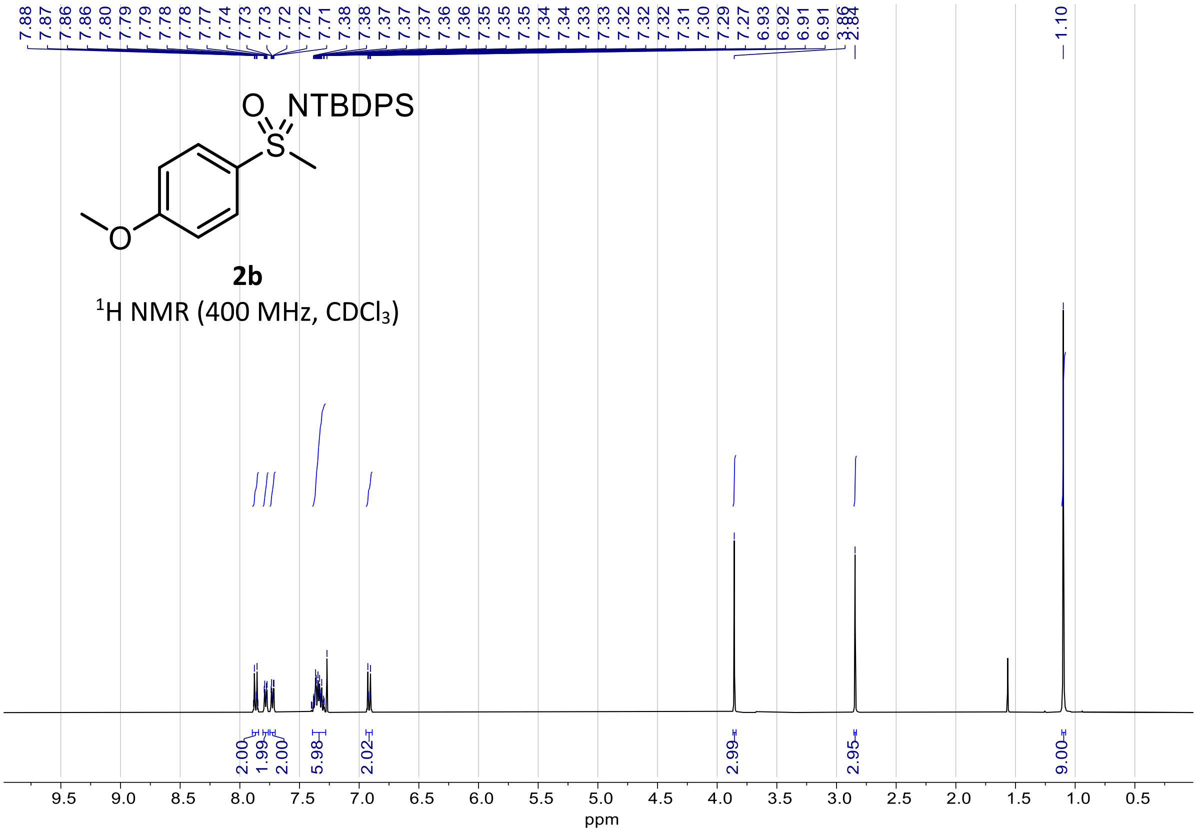
7.88
7.87
7.86
7.86
7.80
7.79
7.79
7.78
7.78
7.77
7.74
7.73
7.73
7.72
7.72
7.71
7.38
7.38
7.37
7.37
7.37
7.36
7.36
7.35
7.35
7.35
7.34
7.34
7.33
7.33
7.32
7.32
7.32
7.31
7.30
7.29
7.27
6.93
6.92
6.91
6.91
3.86
2.84
1.10
O
NTBDPS
S
O
2b
1H NMR (400 MHz, CDCl3)
2.00
1.99
2.00
5.98
2.02
2.99
2.95
9.00
9.5
9.0
8.5
8.0
7.5
7.0
6.5
6.0
5.5
5.0
4.5
4.0
3.5
3.0
2.5
2.0
1.5
1.0
0.5
ppm

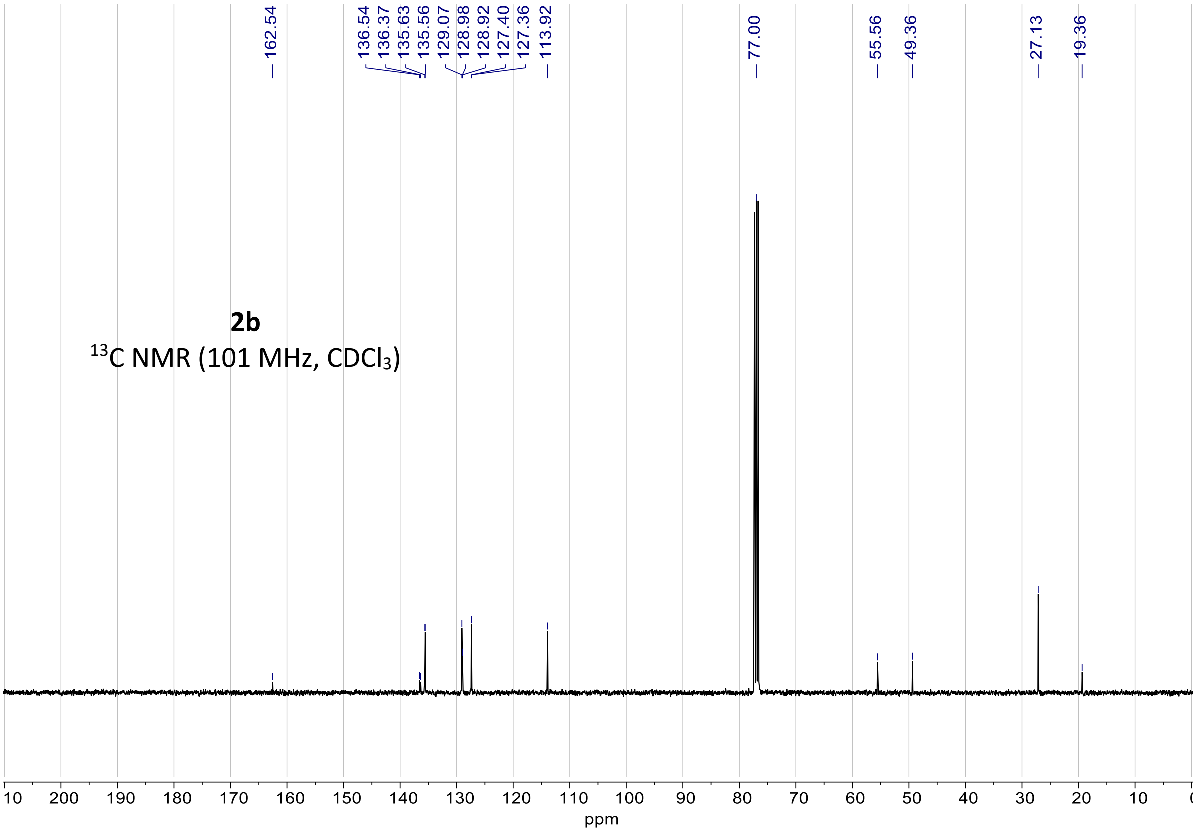
162.54
136.54
136.37
135.63
135.56
129.07
128.98
128.92
127.40
127.36
113.92
77.00
55.56
49.36
27.13
19.36
2b
13C NMR (101 MHz, CDCl3)
200
190
180
170
160
150
140
130
120
110
100
90
80
70
60
50
40
30
20
10
ppm

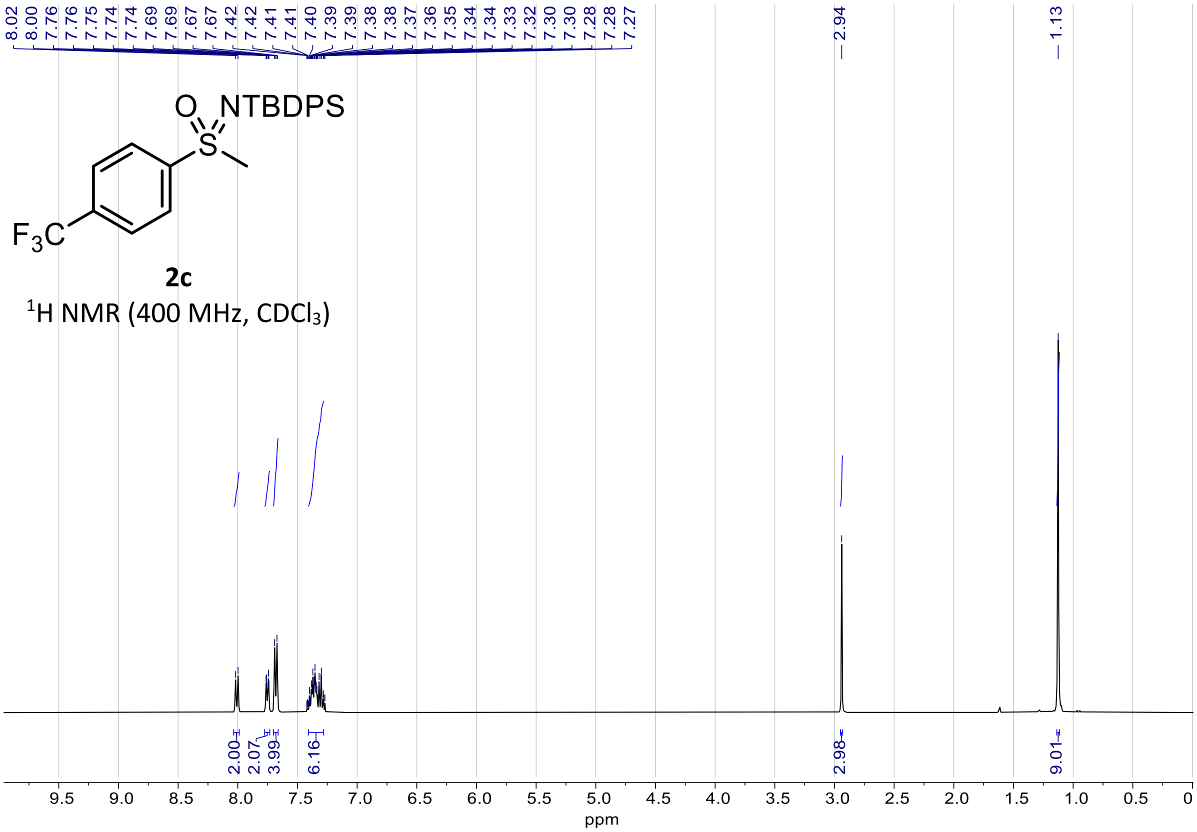
8.02 8.00 7.76 7.76 7.75 7.74 7.74 7.69 7.69 7.67 7.67 7.42 7.42 7.41 7.41 7.40 7.39 7.39 7.38 7.38 7.37 7.36 7.35 7.34 7.34 7.33 7.32 7.30 7.30 7.28 7.28 7.27
2.94
1.13
O NTBDPS
S
F3C
2c
1H NMR (400 MHz, CDCl3)
2.00 2.07 3.99 6.16 2.98 9.01
9.5 9.0 8.5 8.0 7.5 7.0 6.5 6.0 5.5 5.0 4.5 4.0 3.5 3.0 2.5 2.0 1.5 1.0 0.5 0
ppm

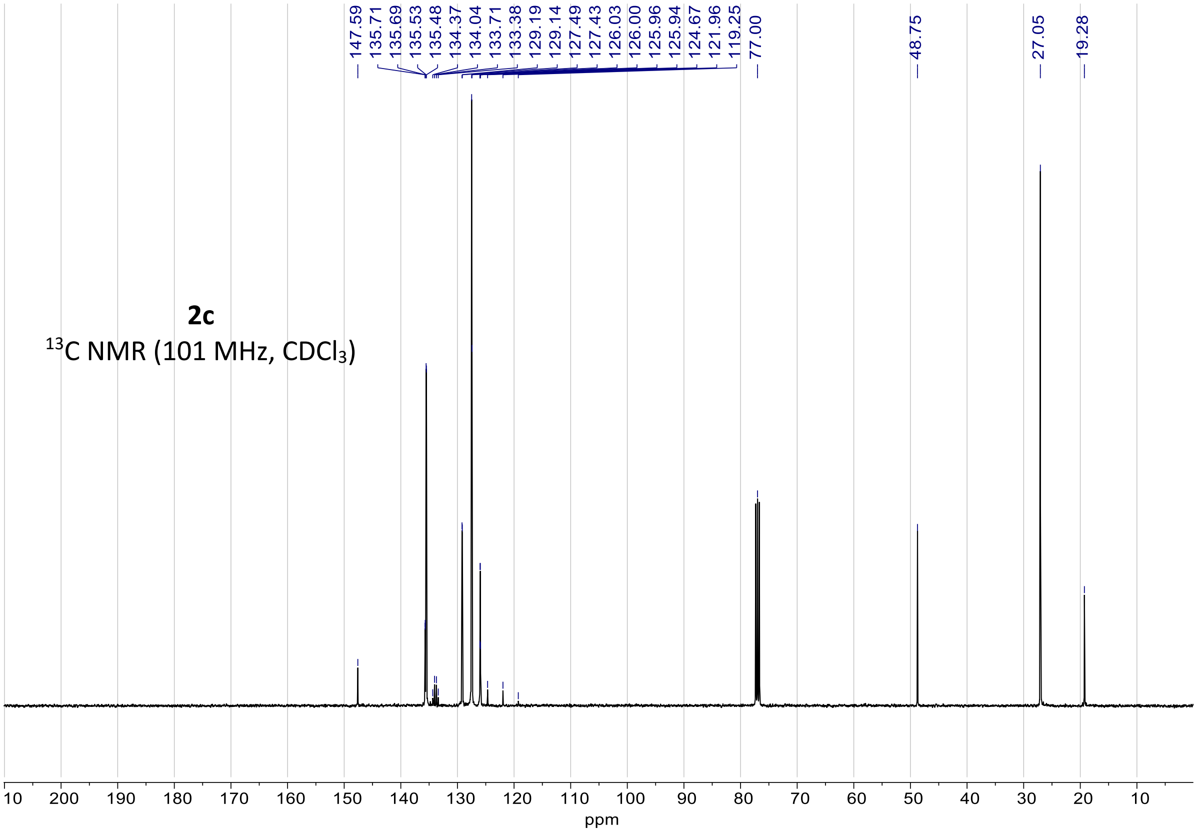
147.59 135.71 135.69 135.53 135.48 134.37 134.04 133.71 133.38 129.19 129.14 127.49 127.43 126.03 126.00 125.96 125.94 124.67 121.96 119.25 77.00
48.75
27.05
19.28
2c
13C NMR (101 MHz, CDCl3)
10 200 190 180 170 160 150 140 130 120 110 100 90 80 70 60 50 40 30 20 10
ppm

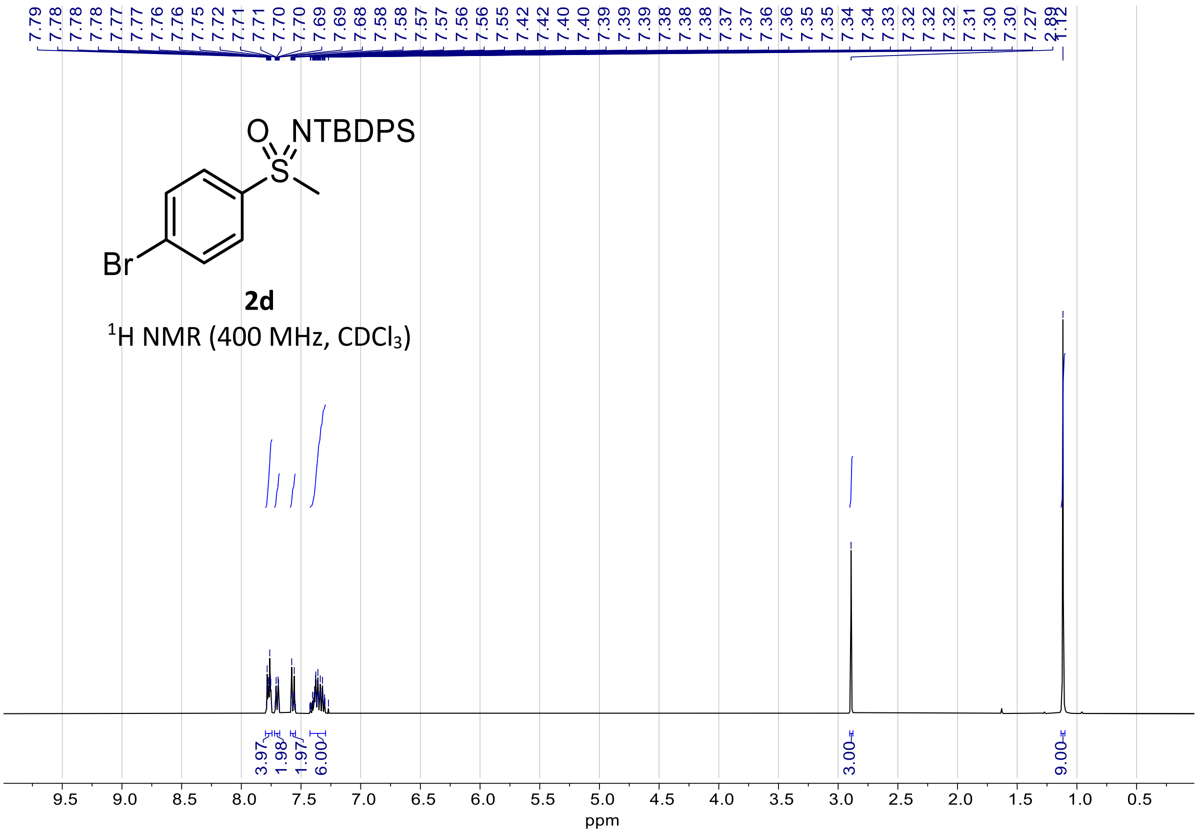
7.79 7.78 7.78 7.78 7.77 7.77 7.76 7.76 7.75 7.72 7.71 7.71 7.70 7.70 7.69 7.69 7.68 7.58 7.58 7.57 7.57 7.56 7.56 7.55 7.42 7.42 7.40 7.40 7.39 7.39 7.39 7.38 7.38 7.38 7.37 7.37 7.36 7.36 7.35 7.35 7.34 7.34 7.33 7.32 7.32 7.32 7.32 7.31 7.30 7.30 7.27 2.89 1.12
O NTBDPS
S
Br
2d
^{1}H NMR (400 MHz, $CDCl_3$)
3.97
1.98
1.97
6.00
3.00
9.00
9.5 9.0 8.5 8.0 7.5 7.0 6.5 6.0 5.5 5.0 4.5 4.0 3.5 3.0 2.5 2.0 1.5 1.0 0.5
ppm

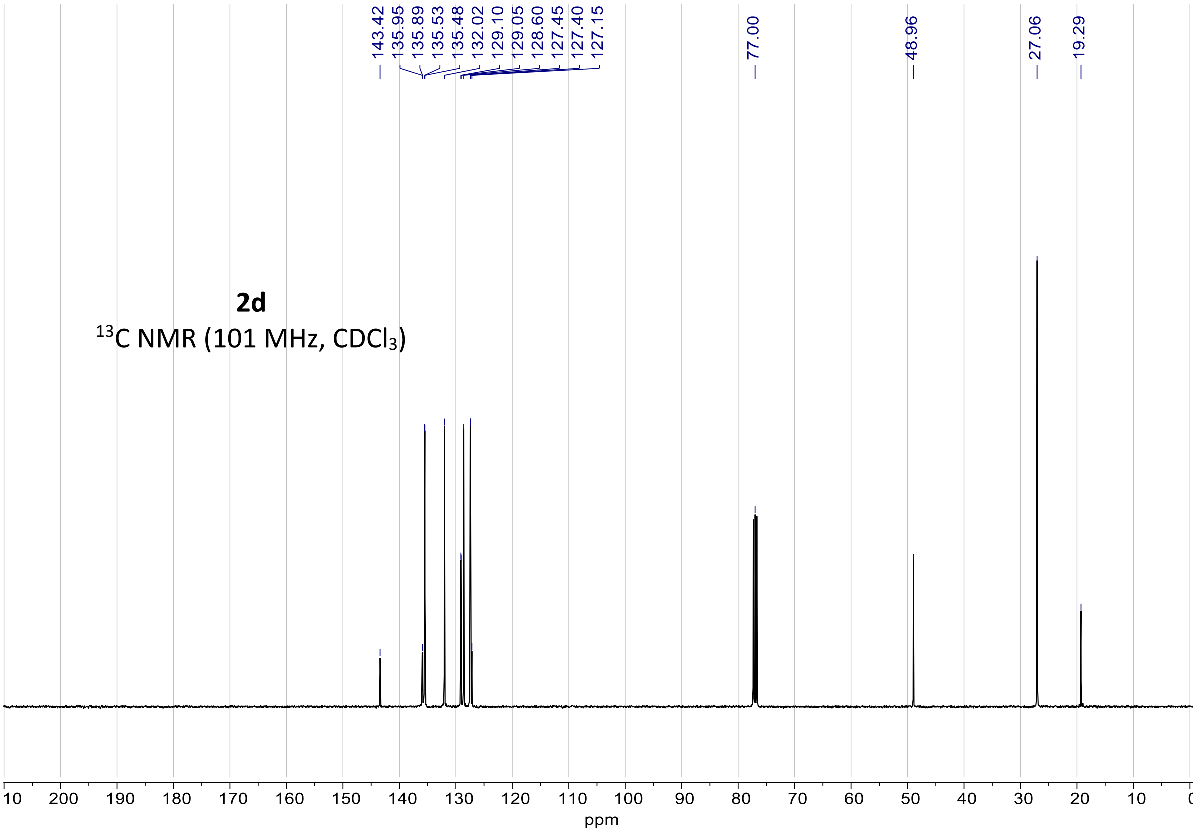
143.42 135.95 135.89 135.53 135.48 132.02 129.10 129.05 128.60 127.45 127.40 127.15
77.00
48.96
27.06
19.29
2d
^{13}C NMR (101 MHz, $CDCl_3$)
10 200 190 180 170 160 150 140 130 120 110 100 90 80 70 60 50 40 30 20 10
ppm

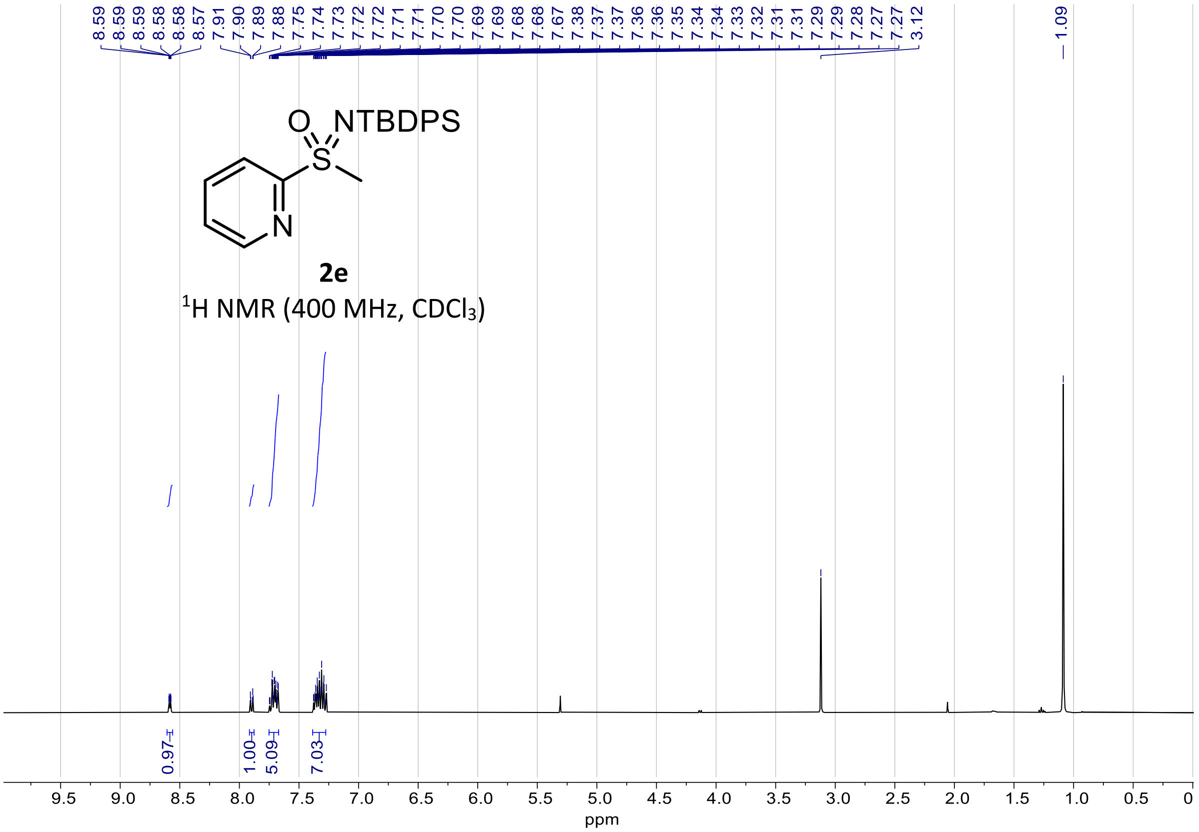
8.59
8.59
8.59
8.58
8.58
8.57
7.91
7.90
7.89
7.88
7.75
7.74
7.73
7.72
7.72
7.71
7.71
7.70
7.70
7.69
7.69
7.68
7.68
7.67
7.38
7.37
7.37
7.36
7.35
7.34
7.34
7.33
7.32
7.31
7.31
7.29
7.29
7.28
7.27
7.27
3.12
1.09
O NTBDPS
S
N
2e
1H NMR (400 MHz, CDCl3)
0.97
1.00
5.09
7.03
9.5 9.0 8.5 8.0 7.5 7.0 6.5 6.0 5.5 5.0 4.5 4.0 3.5 3.0 2.5 2.0 1.5 1.0 0.5 0
ppm

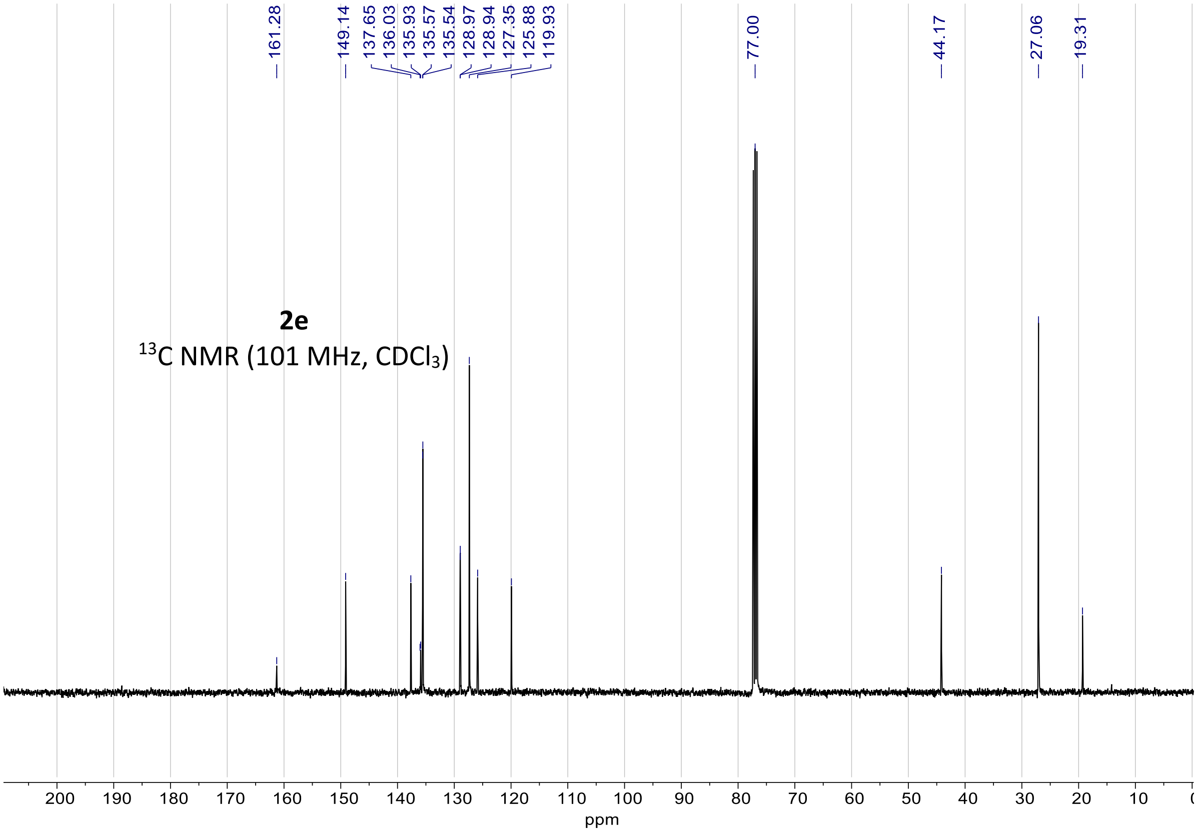
161.28
149.14
137.65
136.03
135.93
135.57
135.54
128.97
128.94
127.35
125.88
119.93
77.00
44.17
27.06
19.31
2e
13C NMR (101 MHz, CDCl3)
200 190 180 170 160 150 140 130 120 110 100 90 80 70 60 50 40 30 20 10
ppm

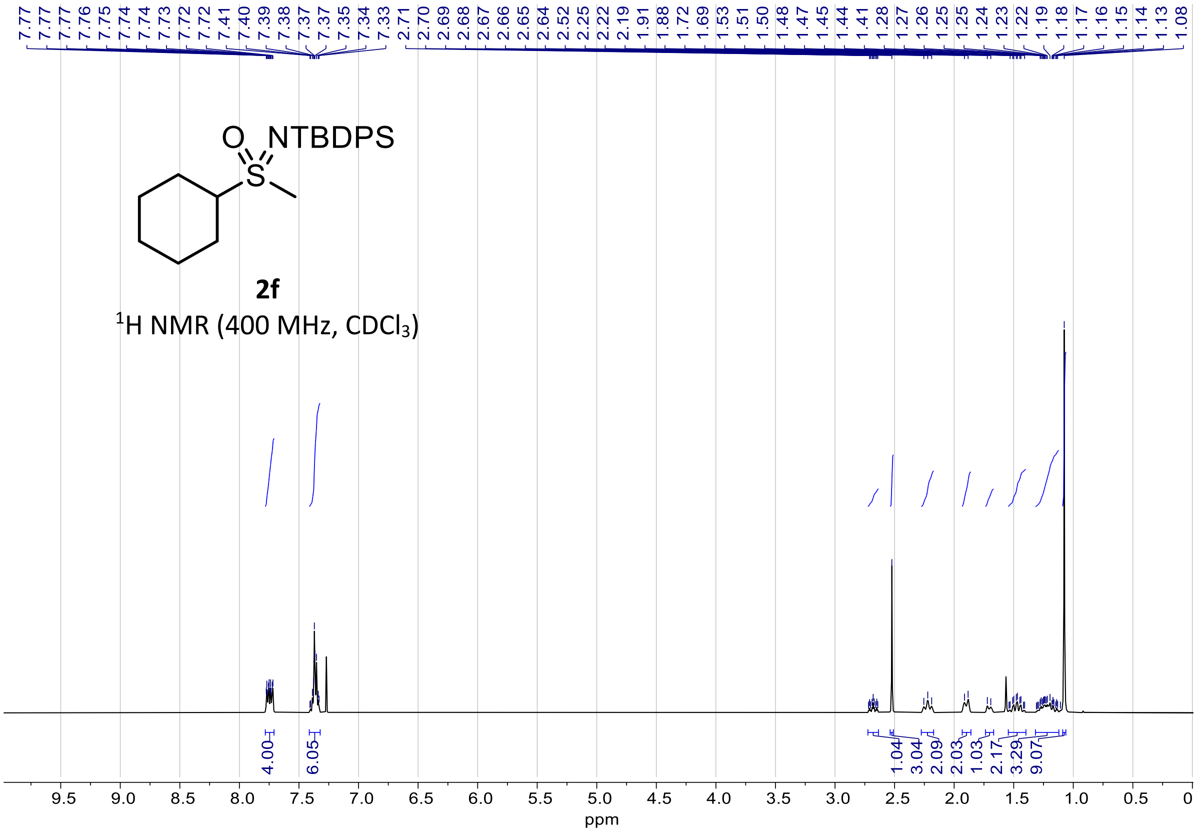

O NTBDPS
S
2f
1H NMR (400 MHz, CDCl3)
7.77 7.77 7.77 7.76 7.75 7.74 7.74 7.73 7.72 7.72 7.41 7.40 7.39 7.38 7.37 7.37 7.35 7.34 7.33 2.71 2.70 2.69 2.68 2.67 2.66 2.65 2.64 2.52 2.25 2.22 2.19 1.91 1.88 1.72 1.69 1.53 1.51 1.50 1.48 1.47 1.45 1.44 1.41 1.28 1.27 1.26 1.25 1.25 1.24 1.23 1.22 1.19 1.18 1.17 1.16 1.15 1.14 1.13 1.08
4.00 6.05 1.04 3.04 2.09 2.03 1.03 2.17 3.29 9.07
9.5 9.0 8.5 8.0 7.5 7.0 6.5 6.0 5.5 5.0 4.5 4.0 3.5 3.0 2.5 2.0 1.5 1.0 0.5 0
ppm

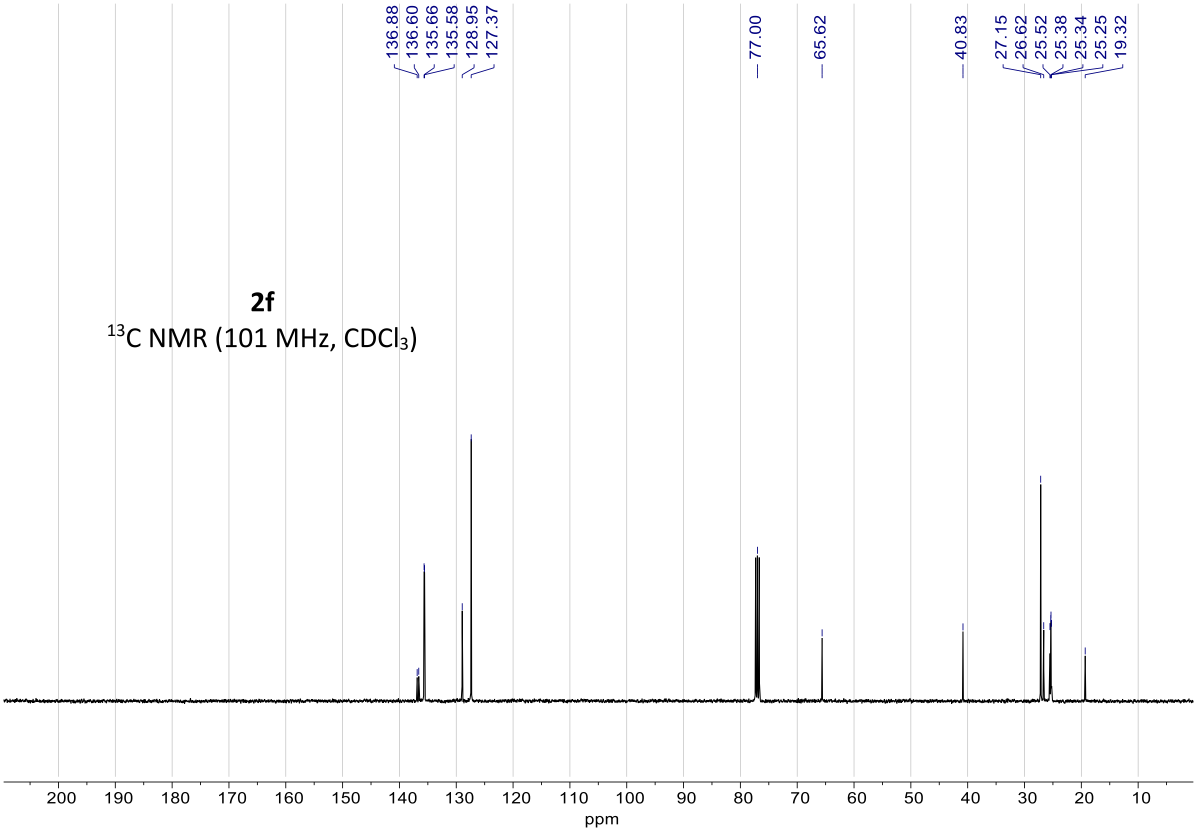

2f
13C NMR (101 MHz, CDCl3)
136.88 136.60 135.66 135.58 128.95 127.37
77.00
65.62
40.83
27.15 26.62 25.52 25.38 25.34 25.25 19.32
200 190 180 170 160 150 140 130 120 110 100 90 80 70 60 50 40 30 20 10
ppm

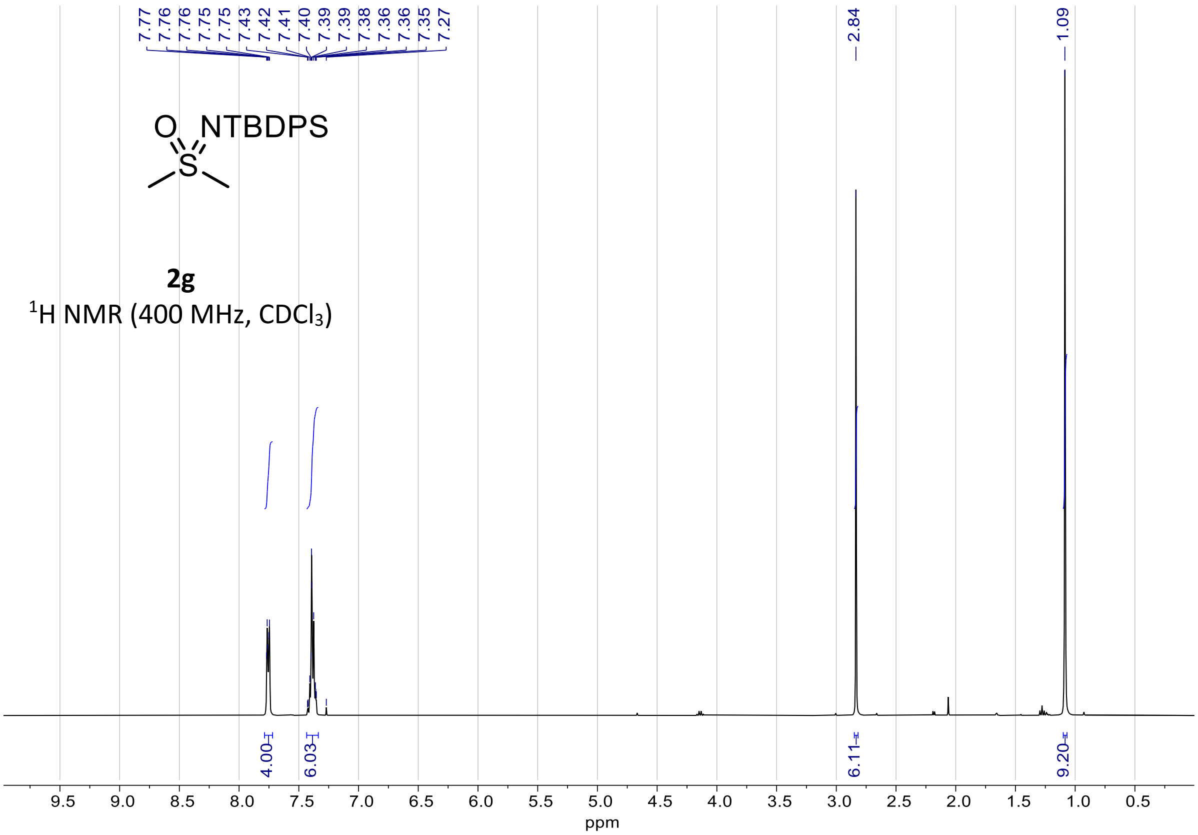
7.77
7.76
7.76
7.75
7.75
7.43
7.42
7.41
7.40
7.39
7.39
7.38
7.36
7.36
7.35
7.27
2.84
1.09
O NTBDPS
S
2g
^{1}H NMR (400 MHz, $CDCl_3$)
4.00
6.03
6.11
9.20
9.5 9.0 8.5 8.0 7.5 7.0 6.5 6.0 5.5 5.0 4.5 4.0 3.5 3.0 2.5 2.0 1.5 1.0 0.5
ppm

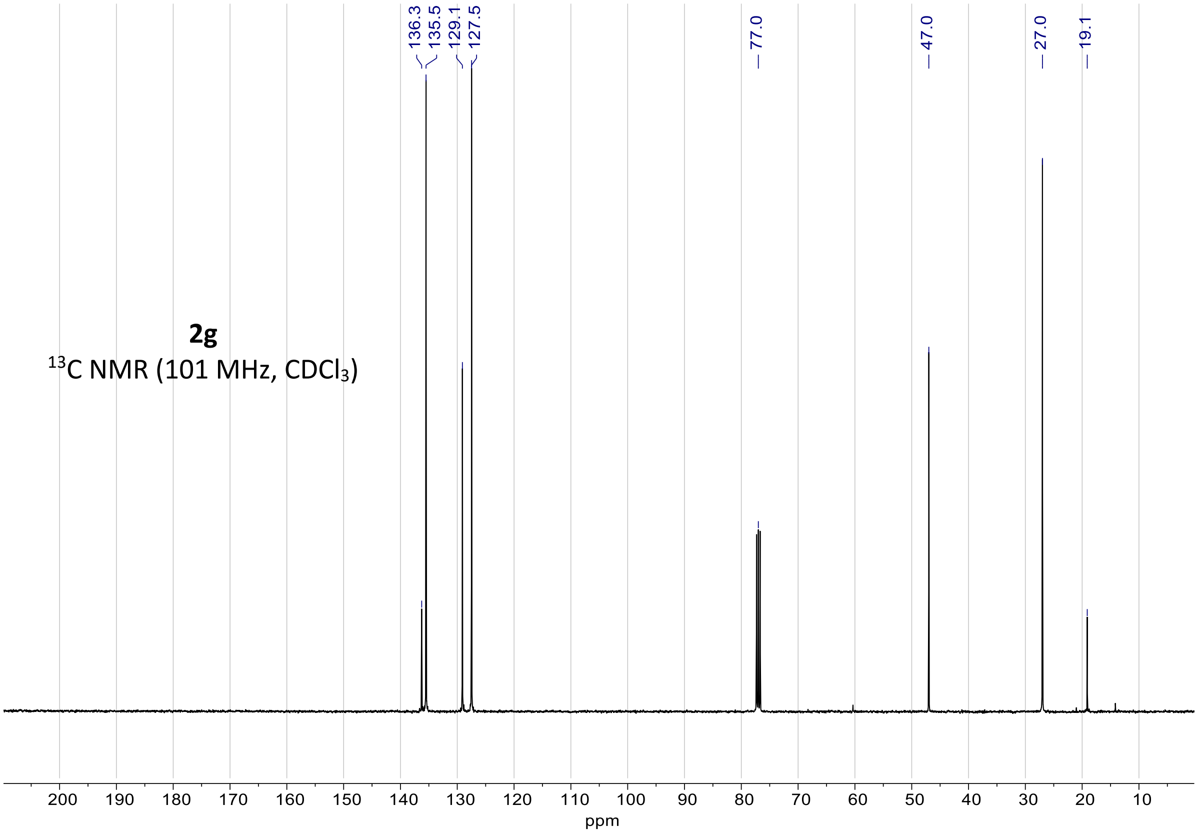
136.3
135.5
129.1
127.5
77.0
47.0
27.0
19.1
2g
^{13}C NMR (101 MHz, $CDCl_3$)
200 190 180 170 160 150 140 130 120 110 100 90 80 70 60 50 40 30 20 10
ppm

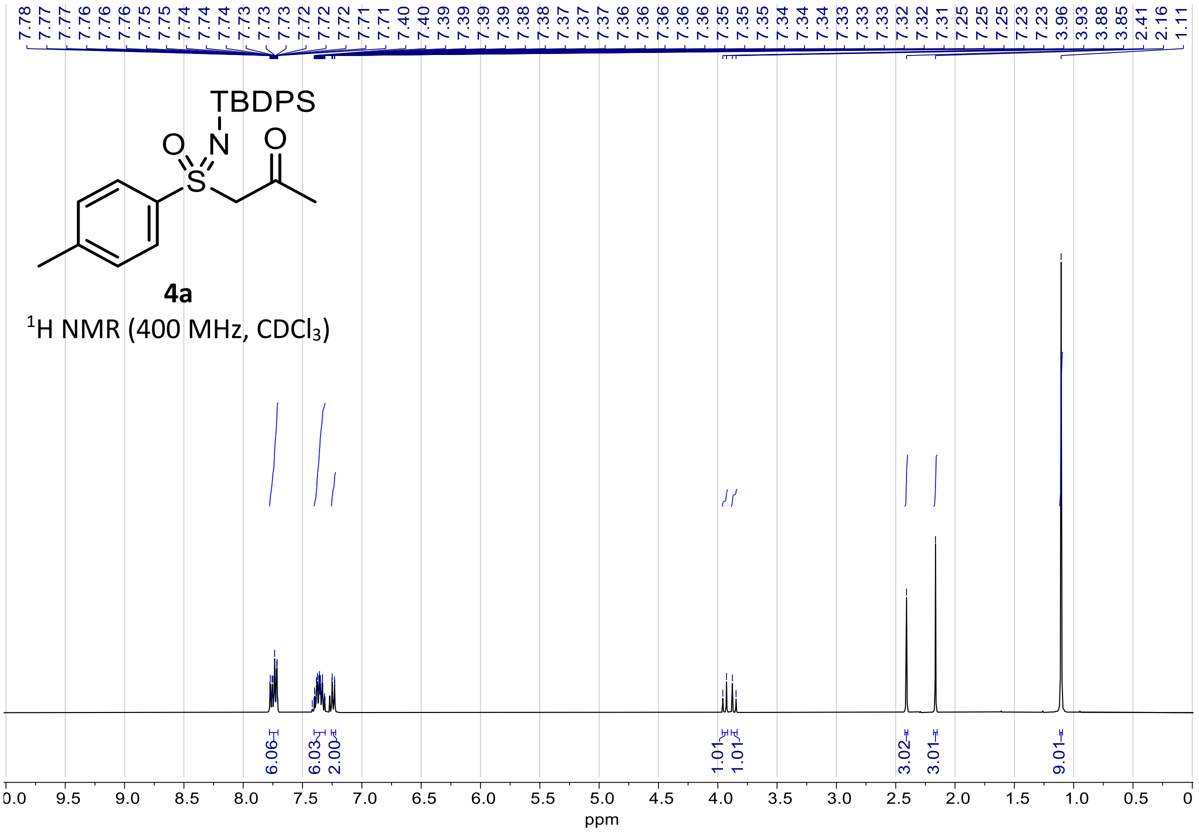
7.78 7.77 7.77 7.76 7.76 7.76 7.75 7.75 7.74 7.74 7.74 7.73 7.73 7.73 7.72 7.72 7.72 7.71 7.71 7.40 7.39 7.39 7.39 7.39 7.38 7.38 7.37 7.37 7.37 7.36 7.36 7.36 7.36 7.35 7.35 7.35 7.34 7.34 7.34 7.33 7.33 7.33 7.32 7.32 7.31 7.25 7.25 7.25 7.23 7.23 3.96 3.93 3.88 3.85 2.41 2.16 1.11
TBDPS
O N O
S
4a
1H NMR (400 MHz, CDCl3)
6.06 6.03 2.00 1.01 1.01 3.02 3.01 9.01
10.0 9.5 9.0 8.5 8.0 7.5 7.0 6.5 6.0 5.5 5.0 4.5 4.0 3.5 3.0 2.5 2.0 1.5 1.0 0.5 0
ppm

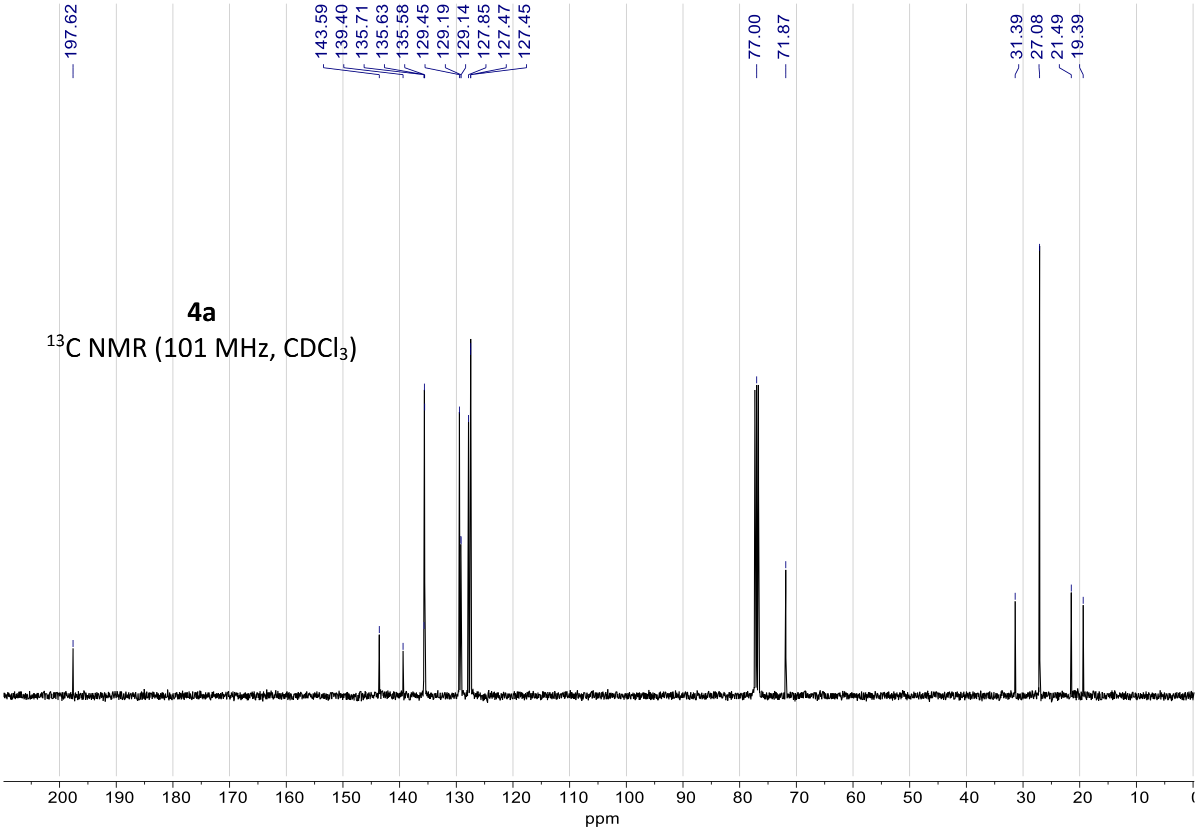
197.62
143.59 139.40 135.71 135.63 135.58 129.45 129.19 129.14 127.85 127.47 127.45
77.00 71.87
31.39 27.08 21.49 19.39
4a
13C NMR (101 MHz, CDCl3)
200 190 180 170 160 150 140 130 120 110 100 90 80 70 60 50 40 30 20 10 0
ppm

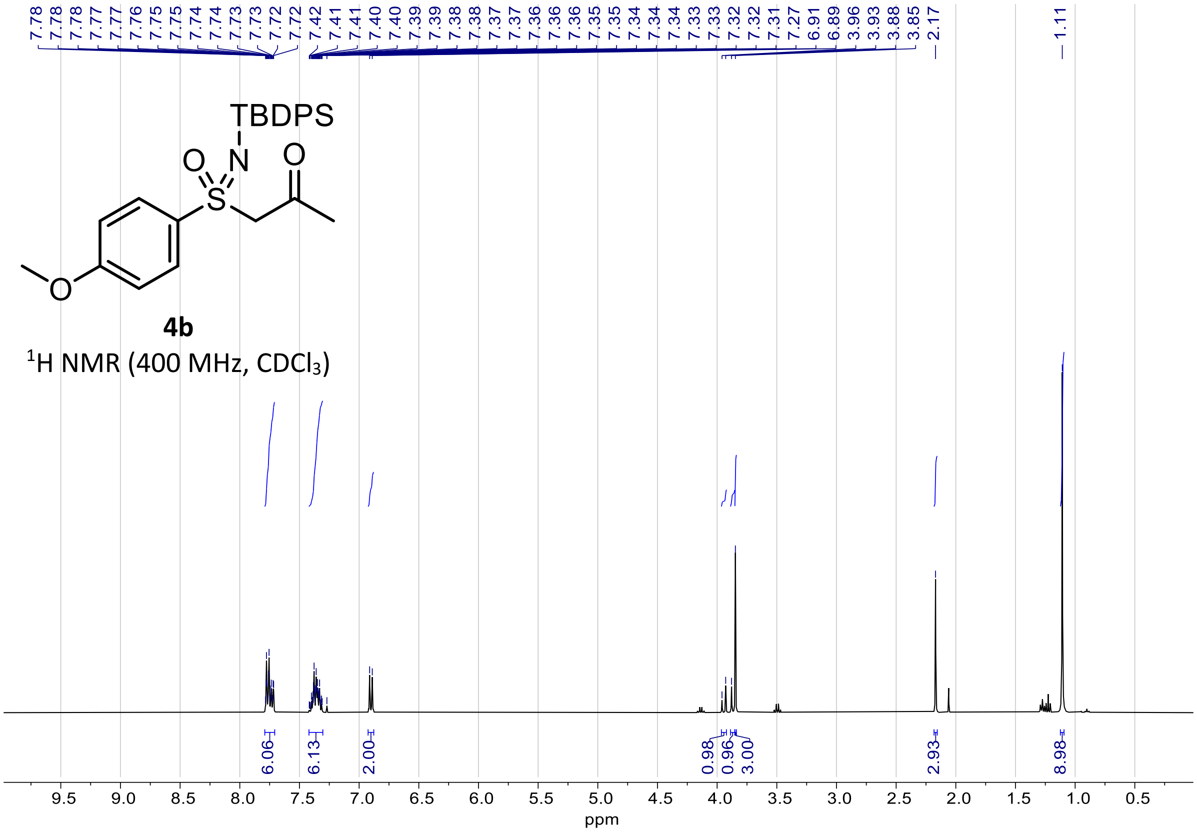

4b

^{1}H NMR (400 MHz, $CDCl_3$)

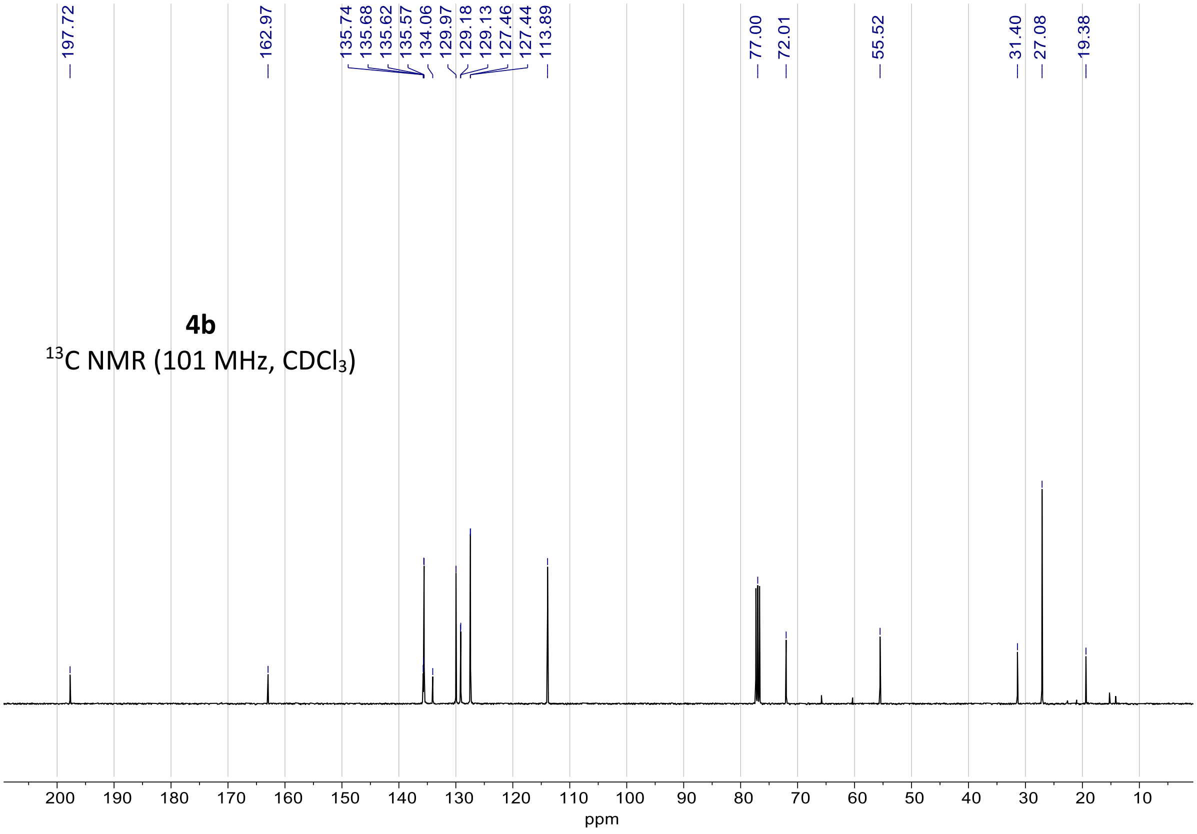

4b

^{13}C NMR (101 MHz, $CDCl_3$)

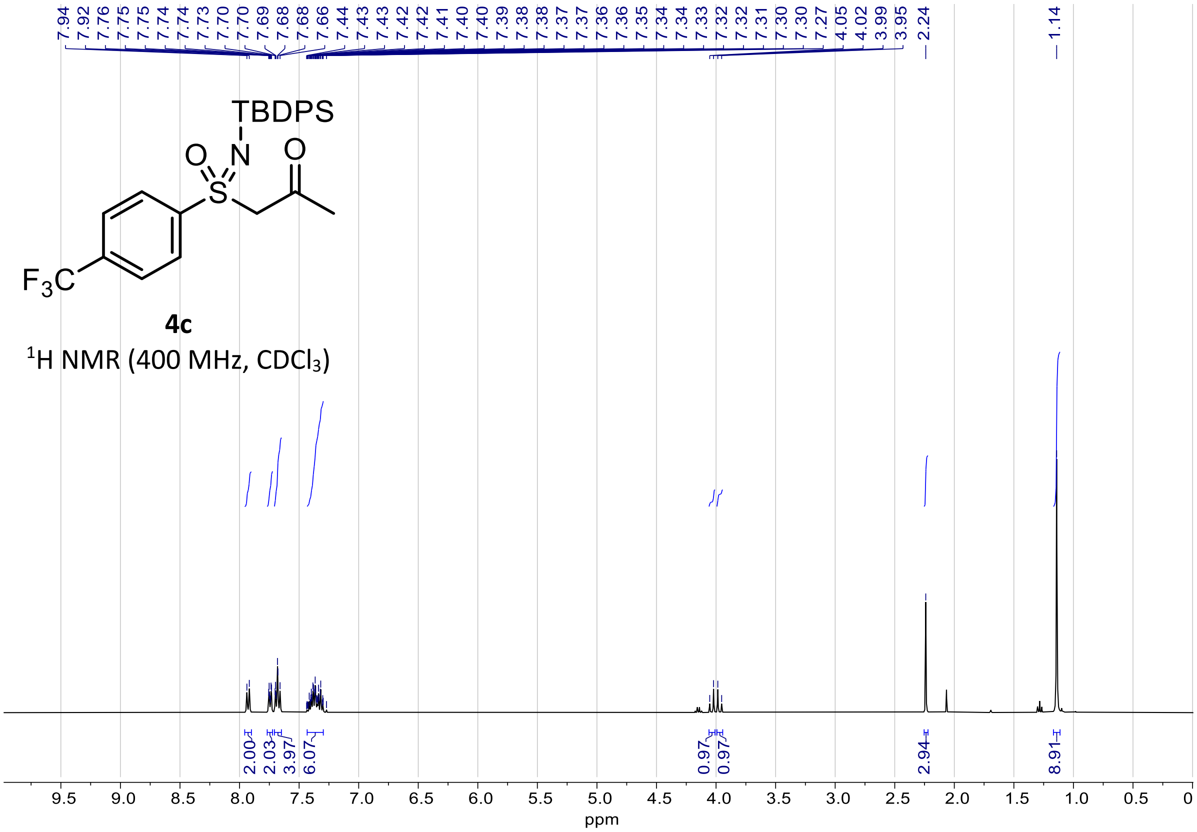

4c

^{1}H NMR (400 MHz, $CDCl_3$)

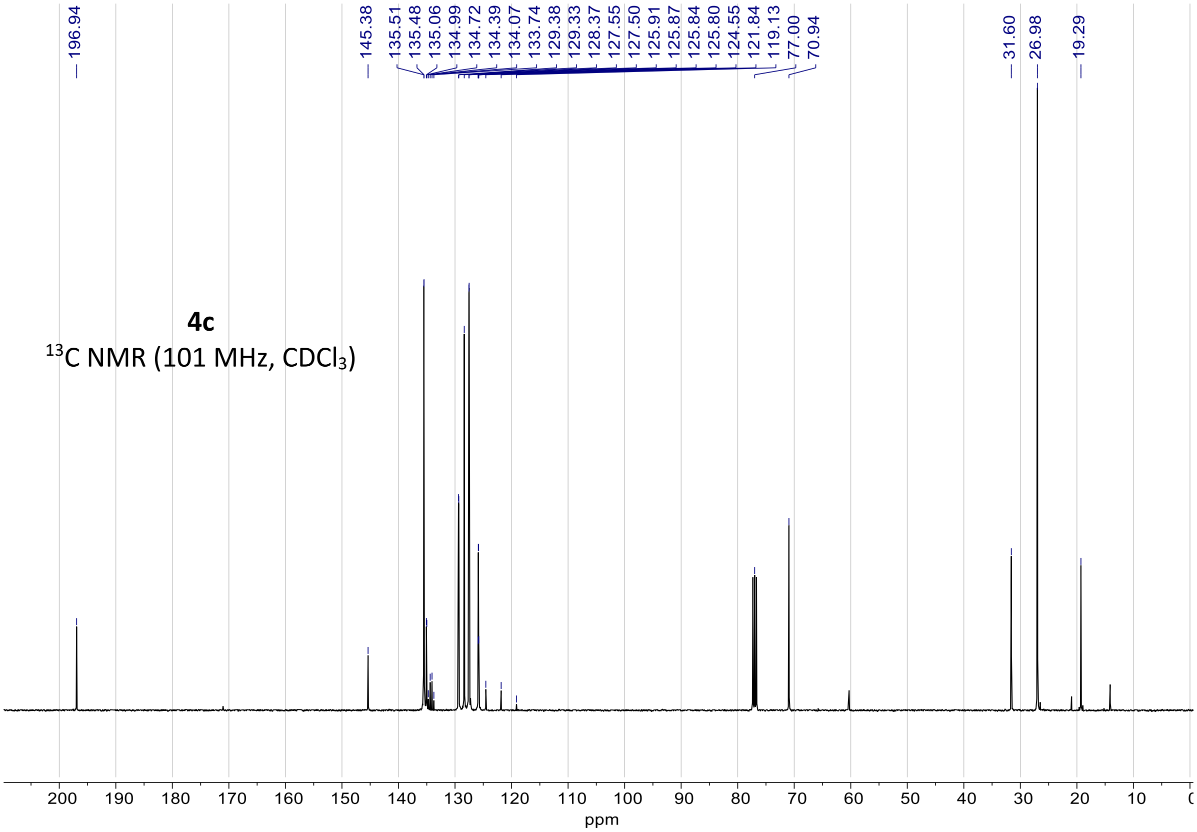

4c

^{13}C NMR (101 MHz, $CDCl_3$)

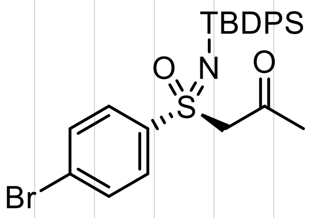

(*S*)-4d

^{1}H NMR (400 MHz, $CDCl_3$)

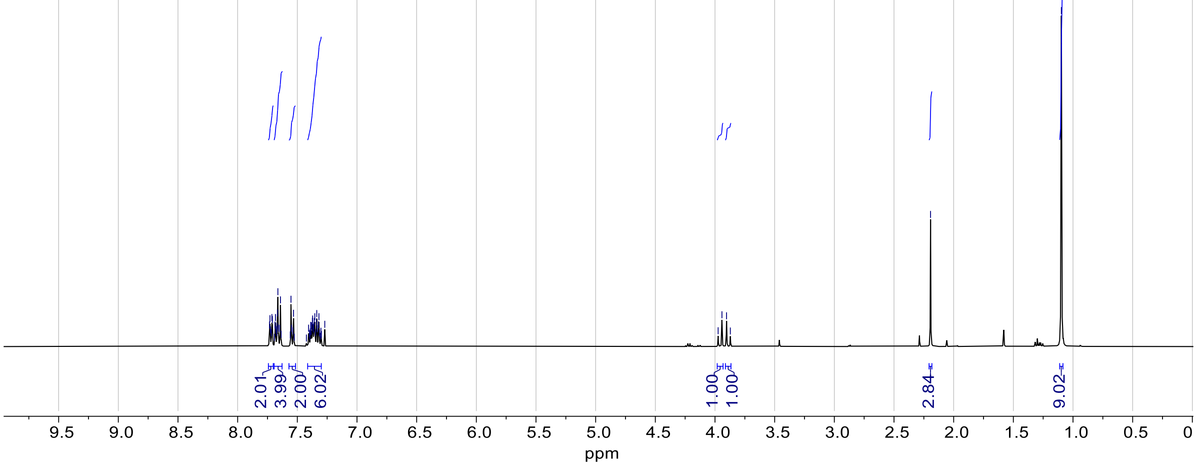

(*S*)-4d

^{13}C NMR (101 MHz, $CDCl_3$)

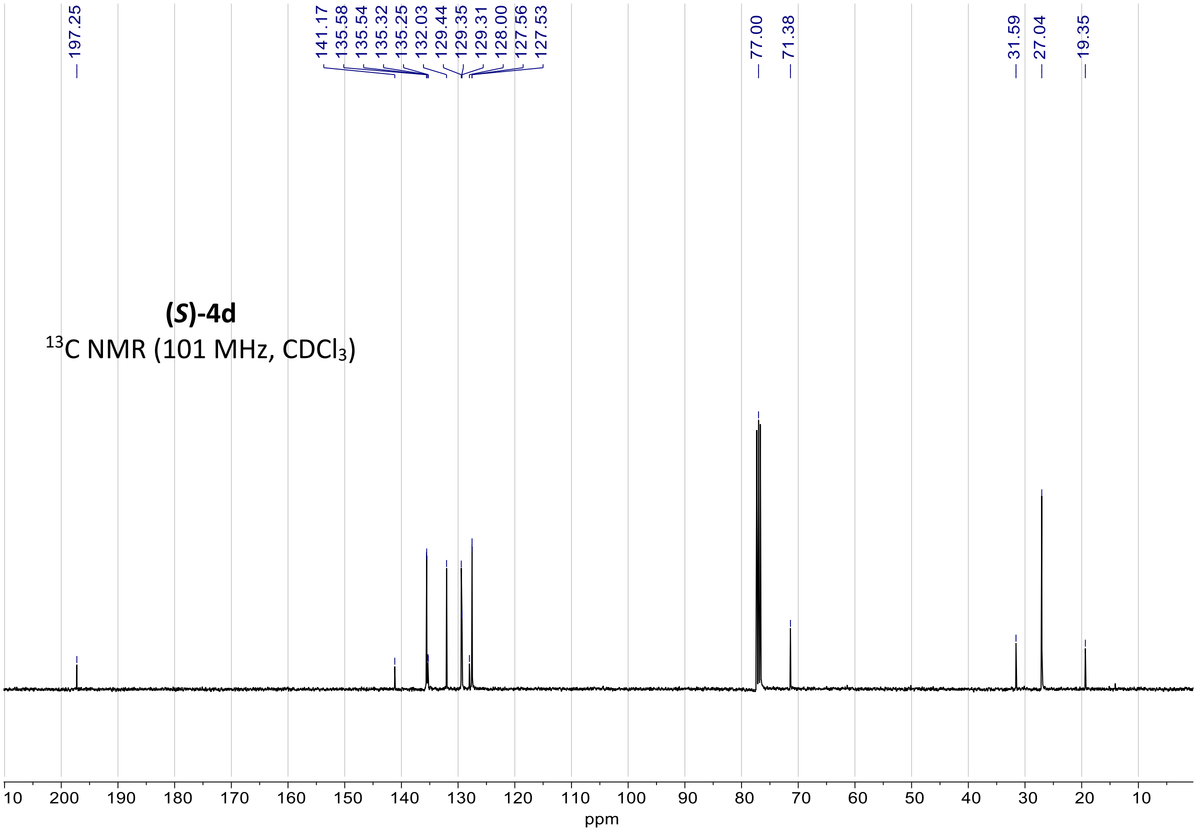

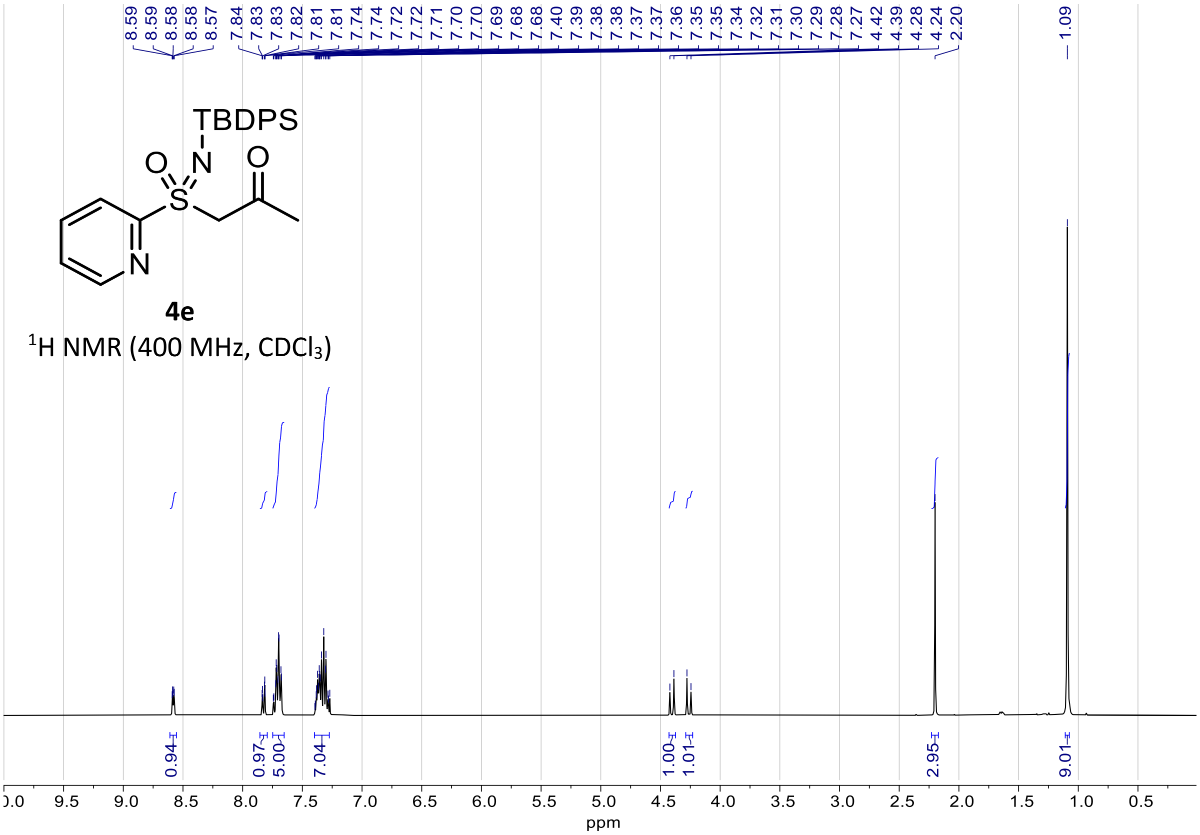
8.59 8.59 8.58 8.58 8.57 7.84 7.83 7.83 7.82 7.81 7.81 7.74 7.74 7.72 7.72 7.71 7.70 7.70 7.69 7.68 7.40 7.39 7.38 7.38 7.37 7.37 7.36 7.35 7.35 7.34 7.32 7.31 7.30 7.29 7.28 7.27 4.42 4.39 4.28 4.24 2.20
1.09
TBDPS
N
O
O
S
N
4e
^{1}H NMR (400 MHz, $CDCl_3$)
0.94
0.97
5.00
7.04
1.00
1.01
2.95
9.01
ppm

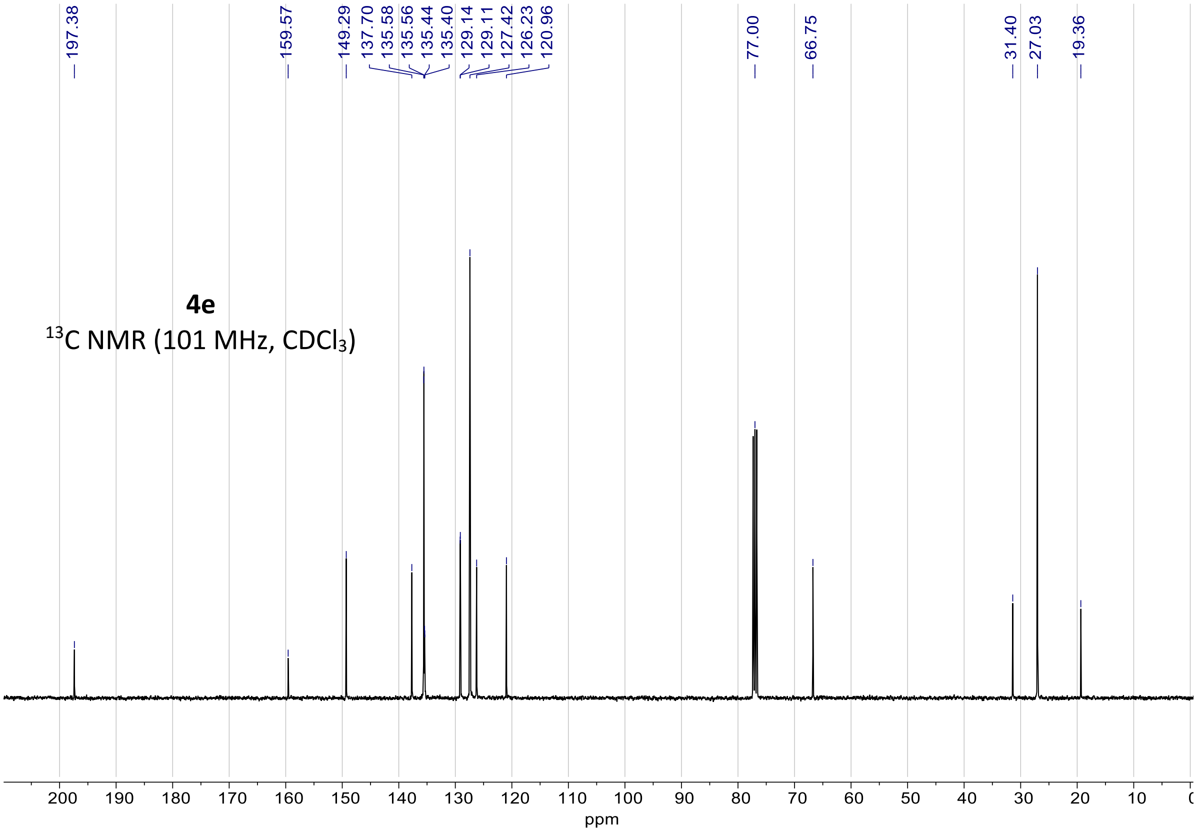
197.38
159.57
149.29
137.70 135.58 135.56 135.44 135.40 129.14 129.11 127.42 126.23 120.96
77.00
66.75
31.40
27.03
19.36
4e
^{13}C NMR (101 MHz, $CDCl_3$)
ppm

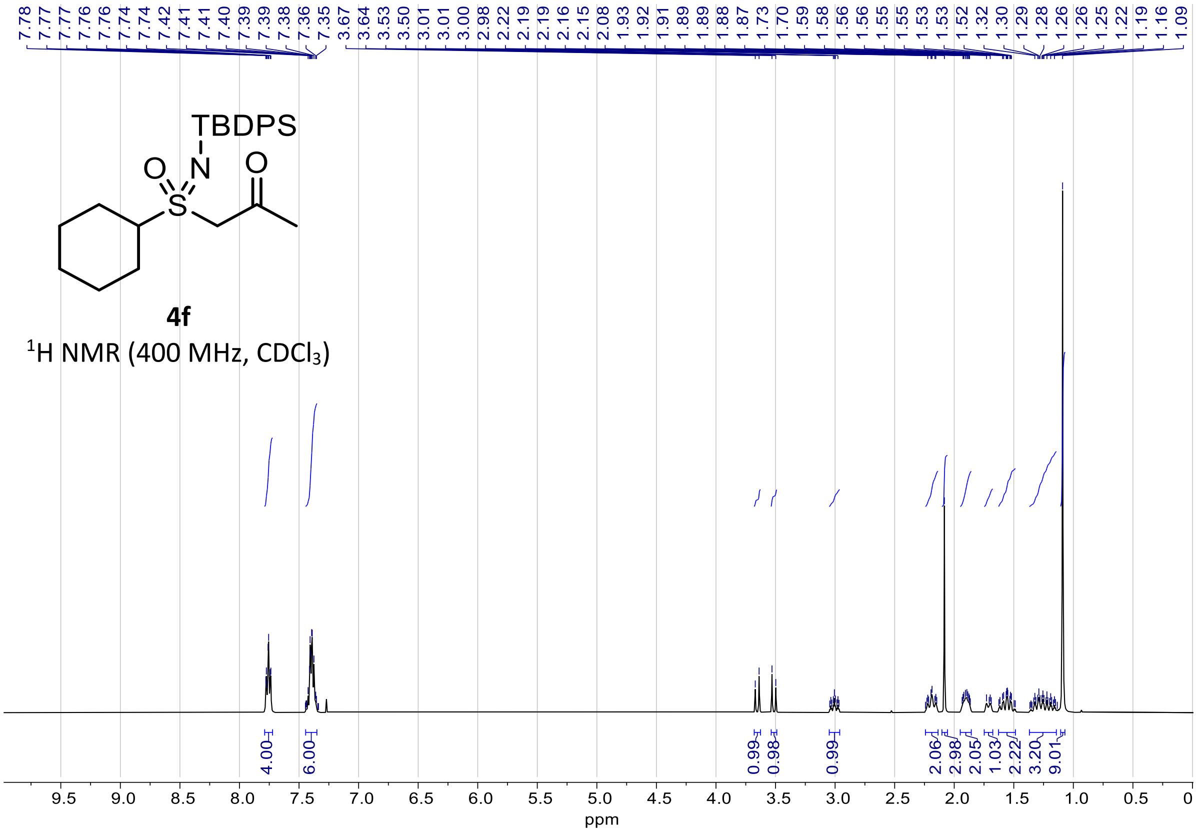
7.78 7.77 7.77 7.76 7.76 7.74 7.74 7.42 7.41 7.41 7.40 7.39 7.39 7.38 7.36 7.35 3.67 3.64 3.53 3.50 3.01 3.01 3.00 2.98 2.22 2.19 2.19 2.16 2.15 2.08 1.93 1.92 1.91 1.89 1.89 1.88 1.87 1.73 1.70 1.59 1.58 1.56 1.56 1.55 1.55 1.53 1.53 1.52 1.32 1.30 1.29 1.28 1.26 1.26 1.25 1.22 1.19 1.16 1.09
TBDPS
N
O
S
O
4f
^{1}H NMR (400 MHz, CDCl$_3$)
4.00 6.00 0.99 0.98 0.99 2.06 2.98 2.05 1.03 2.22 3.20 9.01
9.5 9.0 8.5 8.0 7.5 7.0 6.5 6.0 5.5 5.0 4.5 4.0 3.5 3.0 2.5 2.0 1.5 1.0 0.5 0
ppm

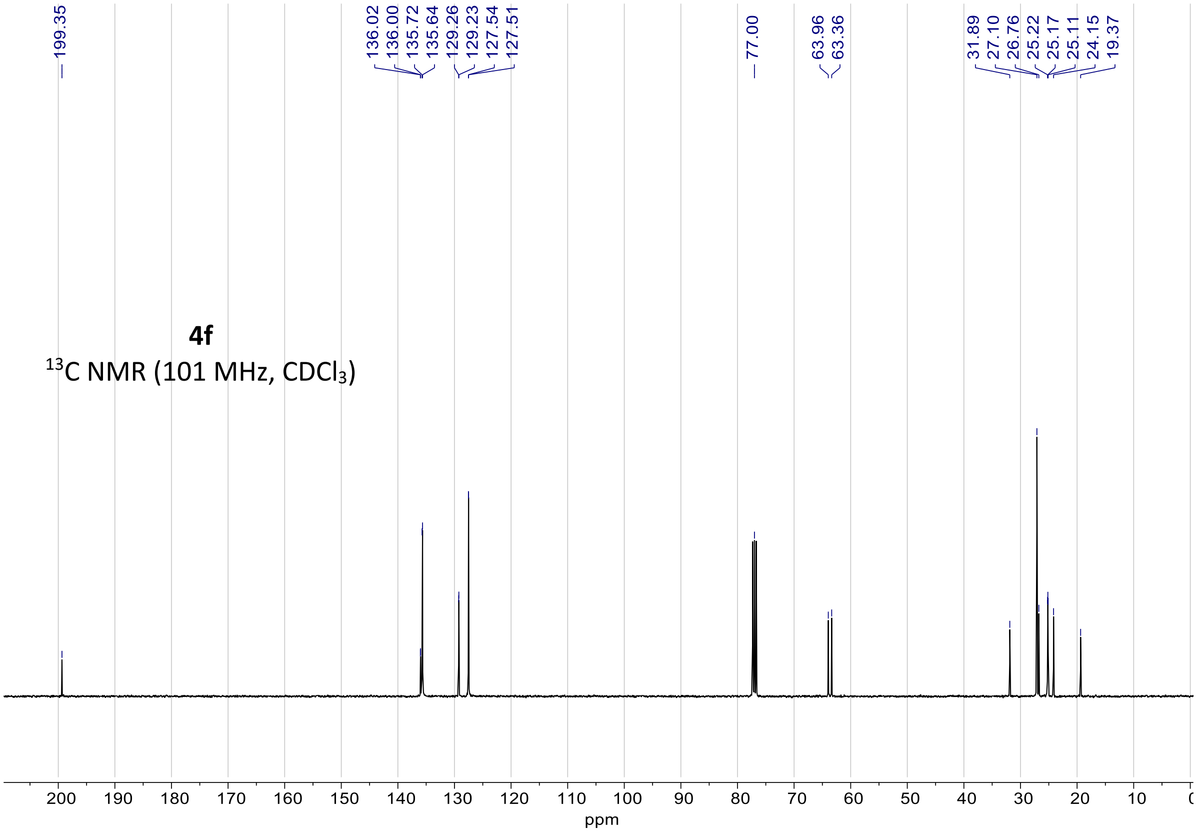
199.35
136.02 136.00 135.72 135.64 129.26 129.23 127.54 127.51
77.00
63.96 63.36
31.89 27.10 26.76 25.22 25.17 25.11 24.15 19.37
4f
^{13}C NMR (101 MHz, CDCl$_3$)
200 190 180 170 160 150 140 130 120 110 100 90 80 70 60 50 40 30 20 10 0
ppm

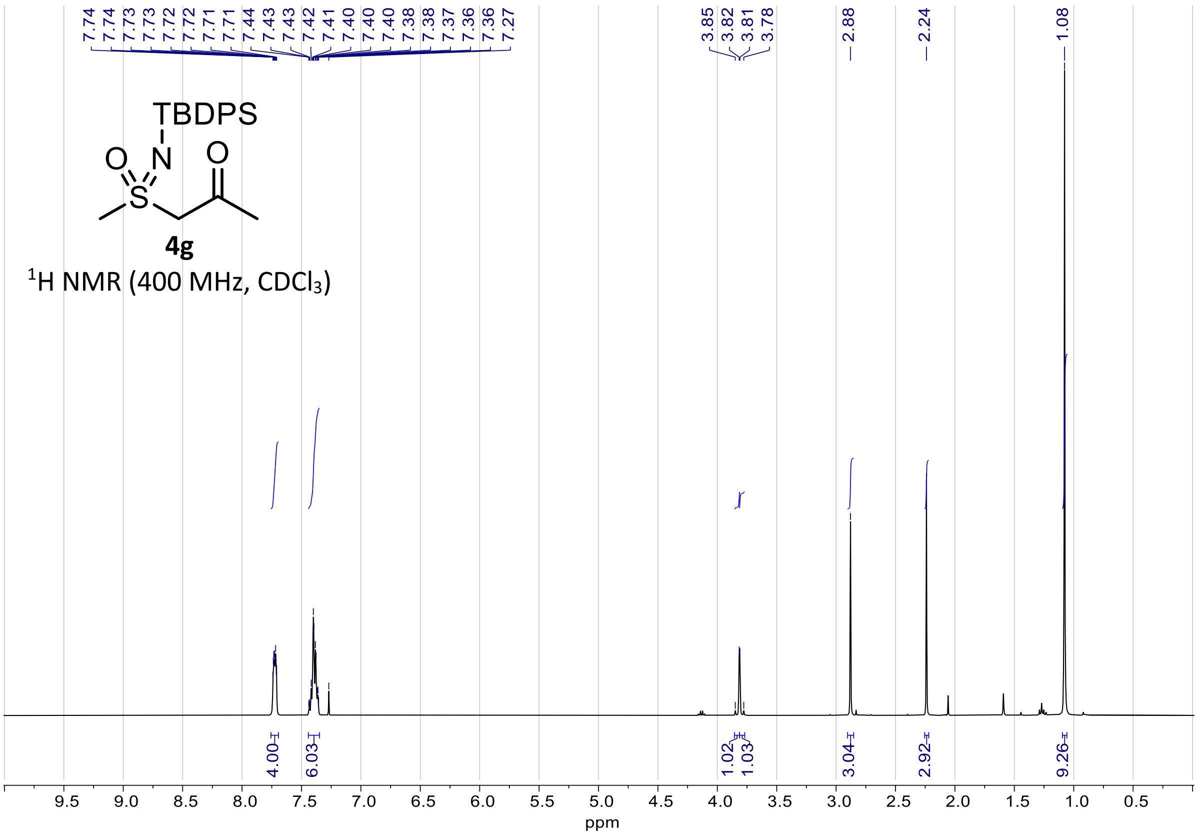
7.74 7.74 7.73 7.73 7.72 7.72 7.71 7.71 7.44 7.43 7.43 7.42 7.41 7.40 7.40 7.38 7.38 7.37 7.36 7.36 7.27
3.85 3.82 3.81 3.78
2.88
2.24
1.08
TBDPS
4g
^{1}H NMR (400 MHz, $CDCl_3$)
4.00
6.03
1.02
1.03
3.04
2.92
9.26
ppm

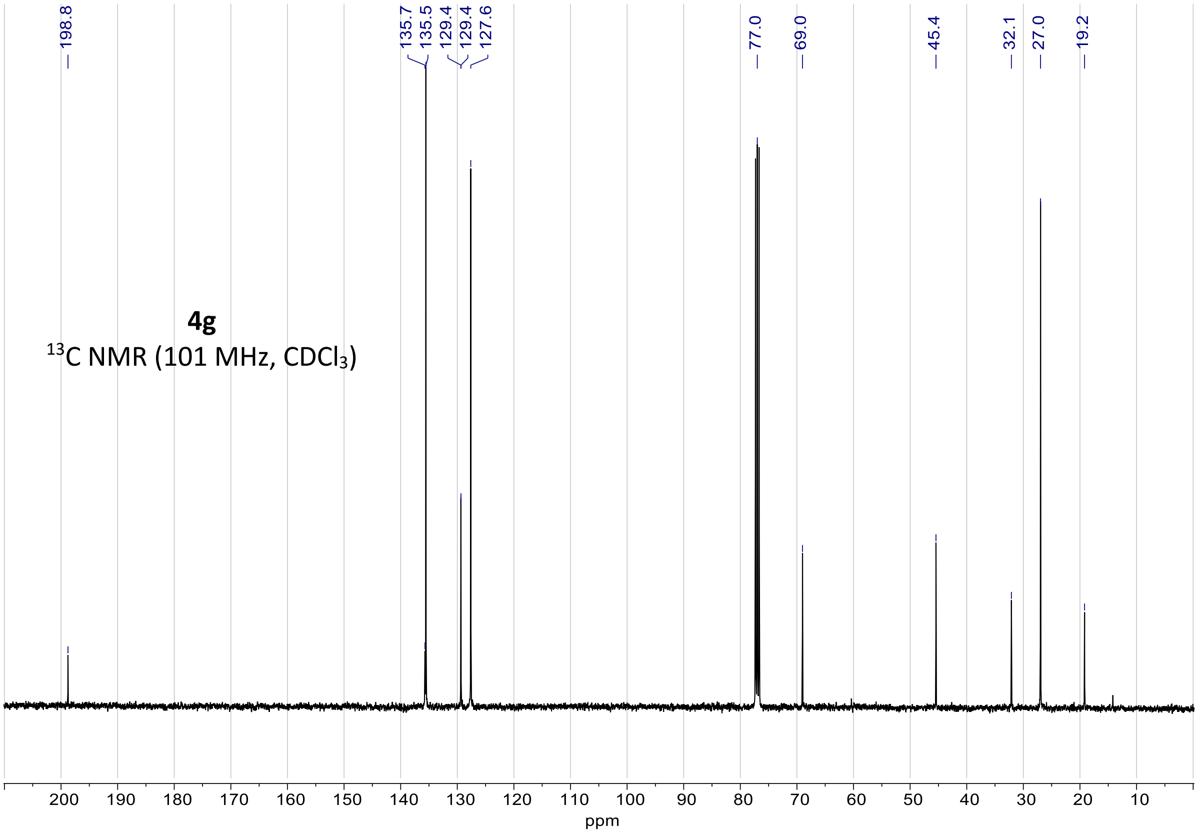
198.8
135.7 135.5 129.4 129.4 127.6
77.0
69.0
45.4
32.1
27.0
19.2
4g
^{13}C NMR (101 MHz, $CDCl_3$)
ppm

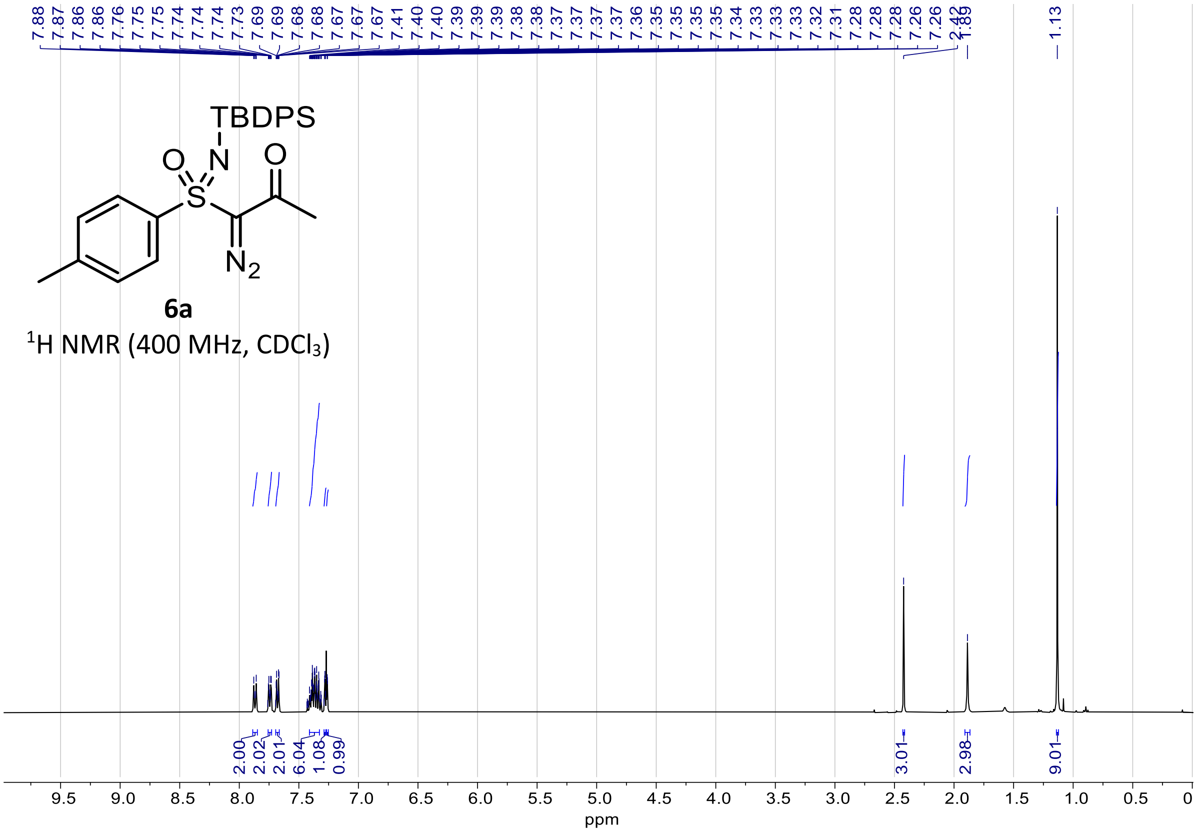
7.88 7.87 7.86 7.76 7.75 7.75 7.74 7.74 7.74 7.73 7.69 7.69 7.68 7.68 7.67 7.67 7.41 7.40 7.40 7.39 7.39 7.39 7.38 7.38 7.37 7.37 7.37 7.37 7.36 7.35 7.35 7.35 7.35 7.34 7.33 7.33 7.33 7.32 7.31 7.28 7.28 7.28 7.26 7.26 2.42 1.89
1.13
TBDPS
N
O
O
S
N2
6a
1H NMR (400 MHz, CDCl3)
2.00 2.02 2.01 6.04 1.08 0.99
3.01
2.98
9.01
ppm

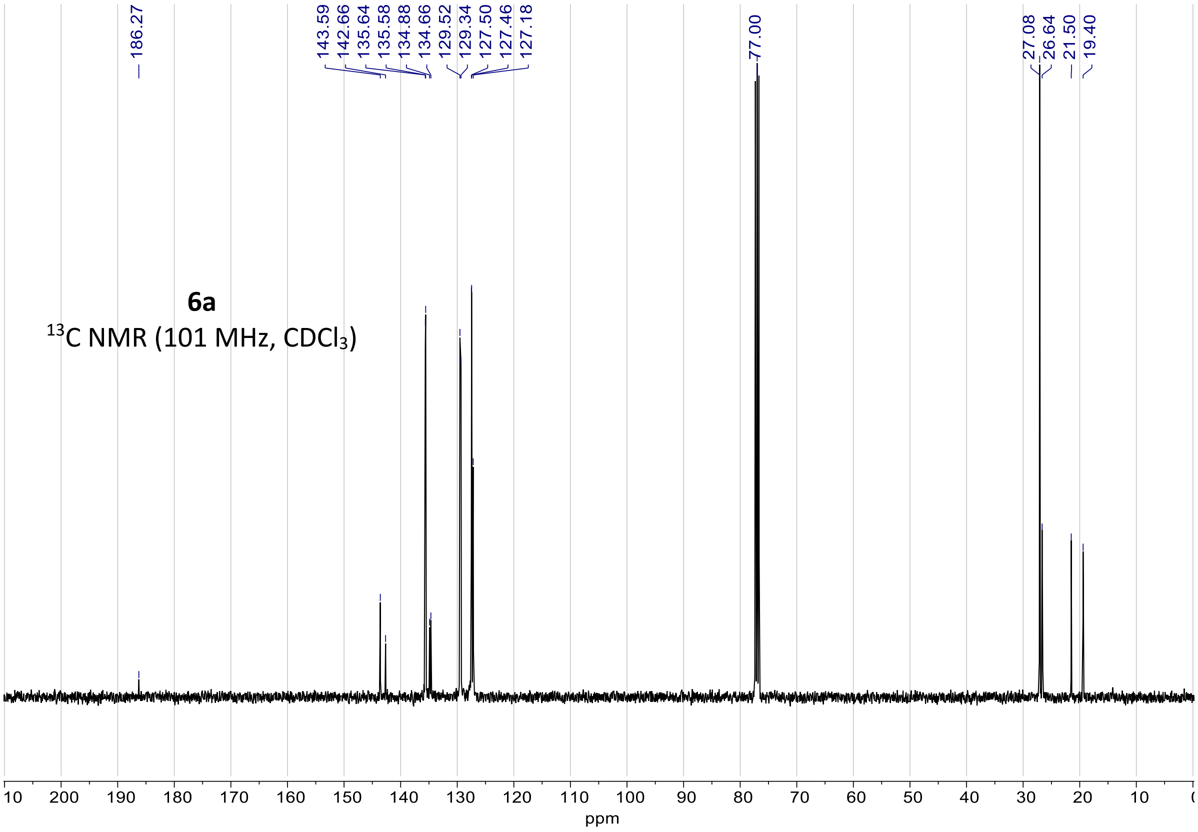
186.27
143.59 142.66 135.64 135.58 134.88 134.66 129.52 129.34 127.50 127.46 127.18
77.00
27.08 26.64 21.50 19.40
6a
13C NMR (101 MHz, CDCl3)
ppm

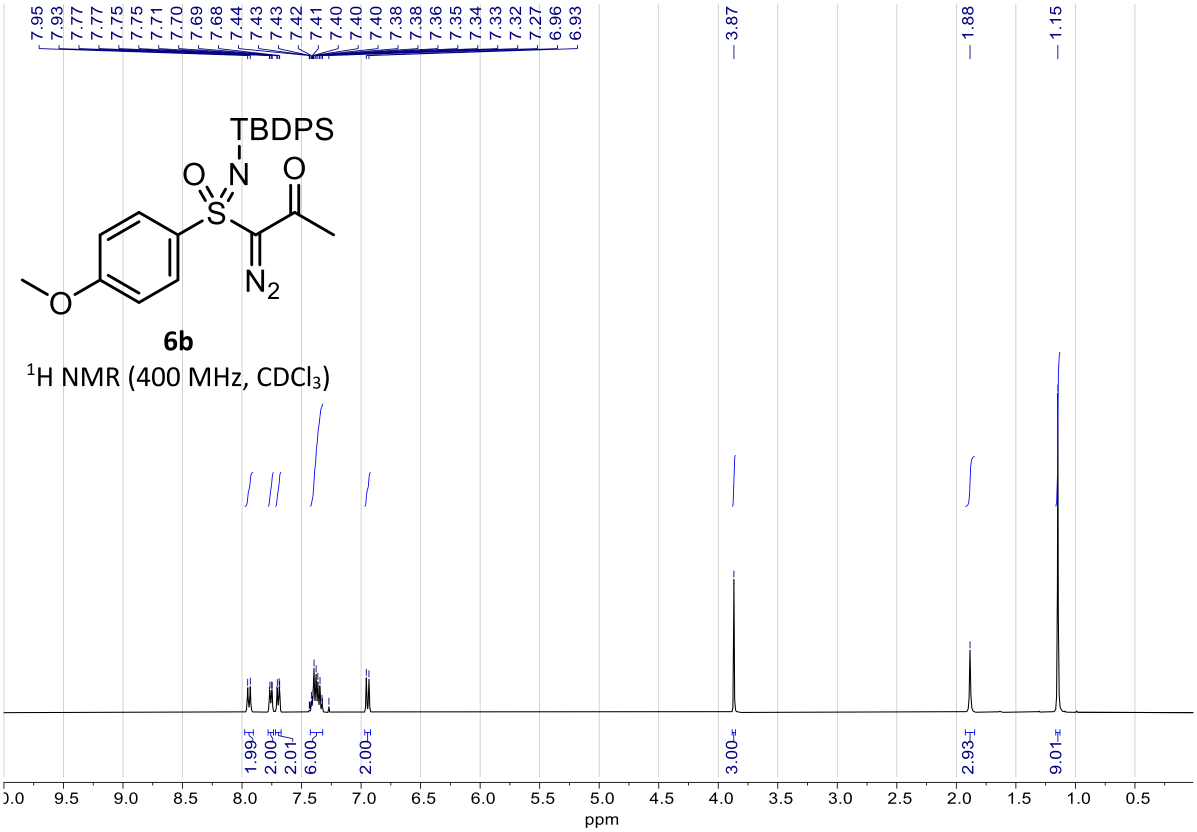
7.95 7.93 7.77 7.77 7.75 7.75 7.71 7.70 7.69 7.68 7.44 7.43 7.43 7.42 7.41 7.40 7.40 7.40 7.38 7.38 7.36 7.35 7.34 7.33 7.32 7.27 6.96 6.93
3.87
1.88
1.15
TBDPS
N
O
S
O
N2
O
6b
1H NMR (400 MHz, CDCl3)
1.99
2.00
2.01
6.00
2.00
3.00
2.93
9.01
ppm

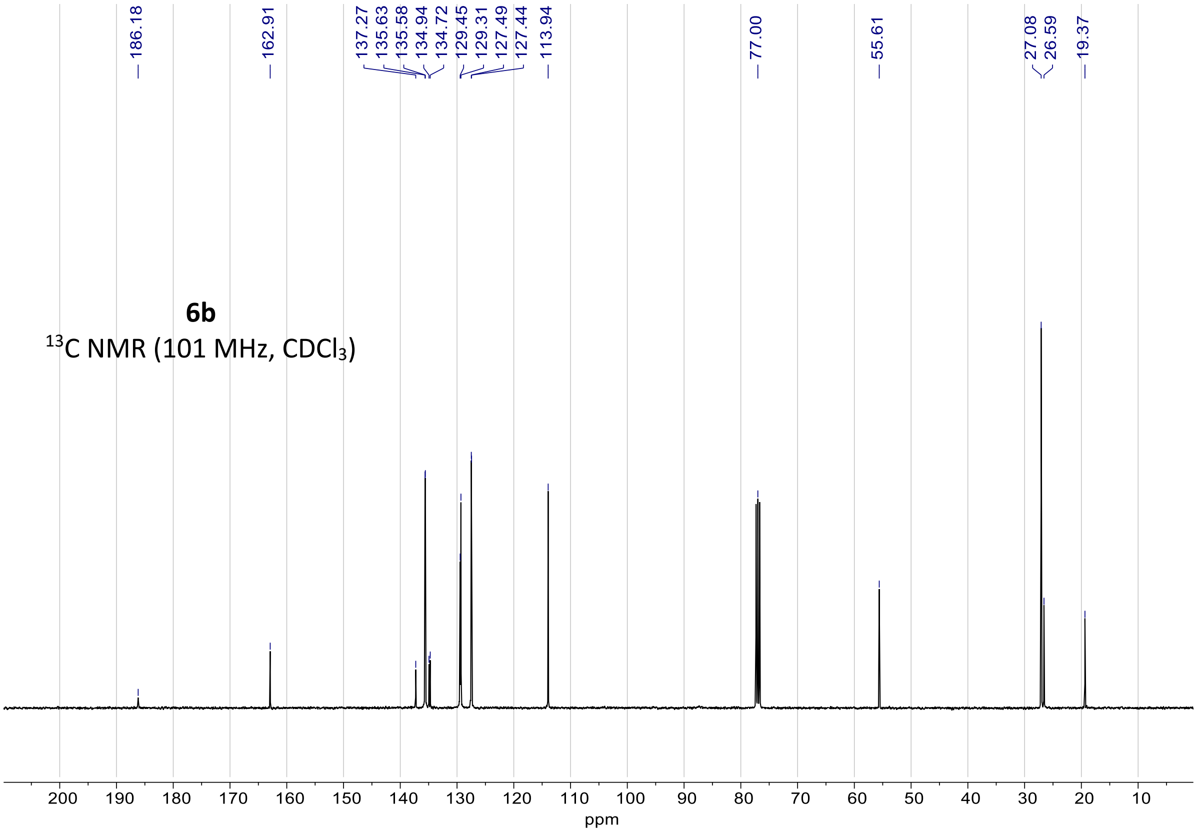
186.18
162.91
137.27 135.63 135.58 134.94 134.72 129.45 129.31 127.49 127.44
113.94
77.00
55.61
27.08 26.59
19.37
6b
13C NMR (101 MHz, CDCl3)
ppm

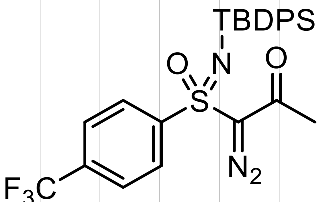

6c

^{1}H NMR (400 MHz, $CDCl_3$)

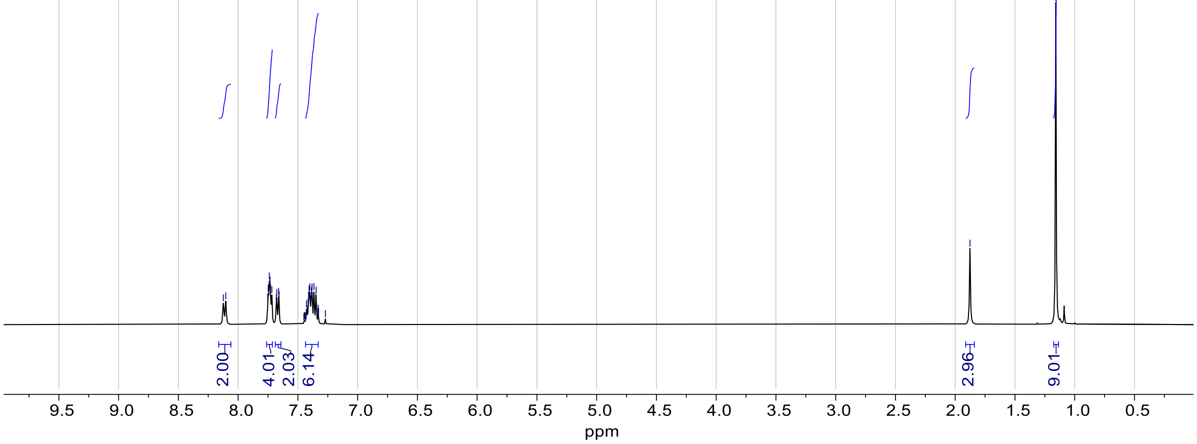

6c

^{13}C NMR (101 MHz, $CDCl_3$)

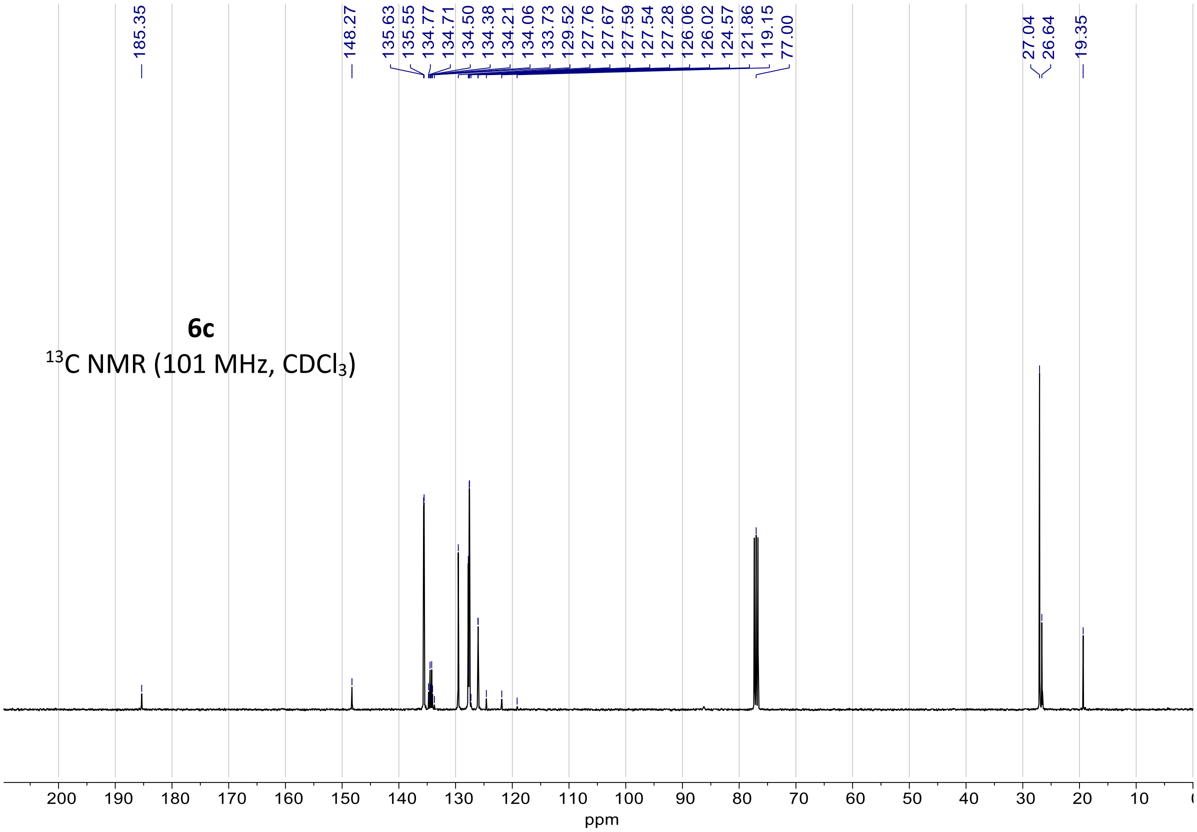

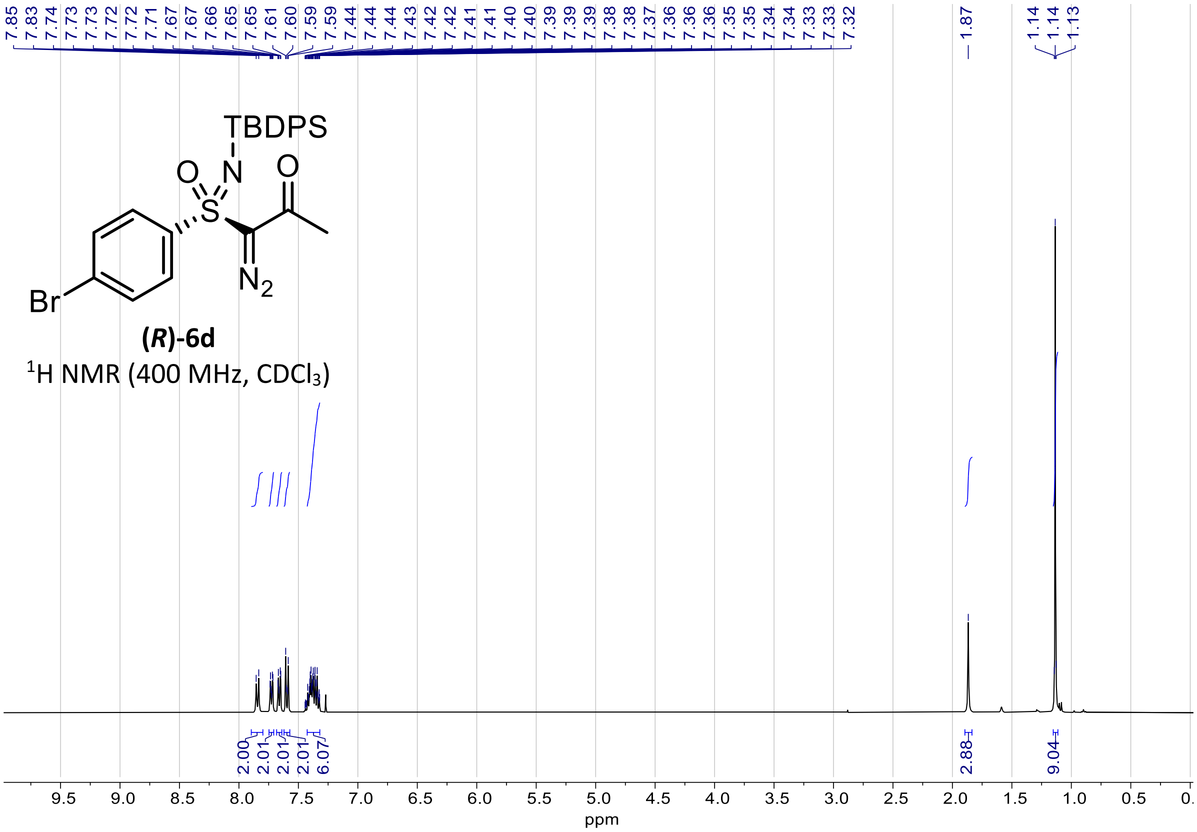
7.85 7.83 7.74 7.73 7.73 7.72 7.72 7.71 7.67 7.67 7.66 7.65 7.65 7.61 7.60 7.59 7.59 7.44 7.44 7.43 7.42 7.42 7.41 7.41 7.40 7.40 7.39 7.39 7.38 7.38 7.37 7.36 7.36 7.35 7.35 7.34 7.34 7.33 7.33 7.32
1.87
1.14 1.14 1.13
TBDPS
O
N
O
S
Br
N2
(*R*)-6d
1H NMR (400 MHz, CDCl3)
2.00 2.01 2.01 2.01 6.07
2.88
9.04
9.5 9.0 8.5 8.0 7.5 7.0 6.5 6.0 5.5 5.0 4.5 4.0 3.5 3.0 2.5 2.0 1.5 1.0 0.5
ppm

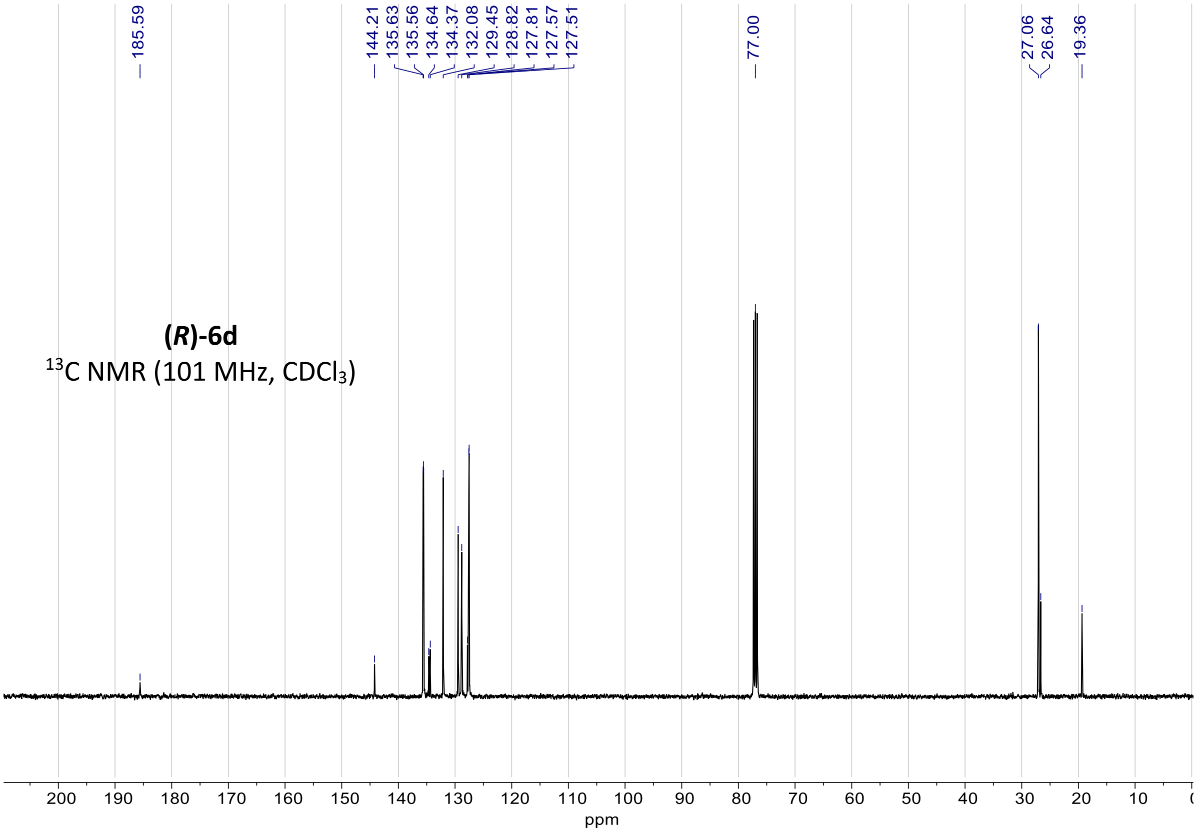
185.59
144.21 135.63 135.56 134.64 134.37 132.08 129.45 128.82 127.81 127.57 127.51
77.00
27.06 26.64 19.36
(*R*)-6d
13C NMR (101 MHz, CDCl3)
200 190 180 170 160 150 140 130 120 110 100 90 80 70 60 50 40 30 20 10
ppm

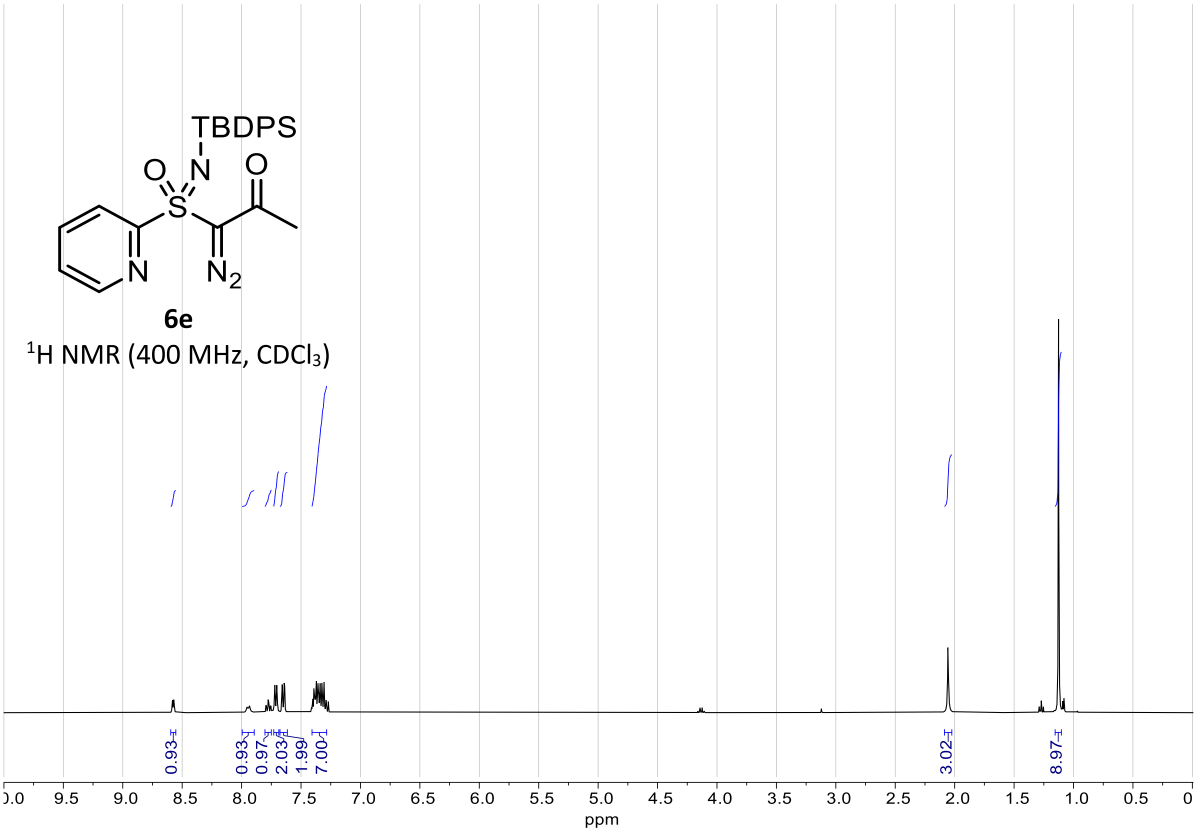
TBDPS
6e
1H NMR (400 MHz, CDCl3)
0.93
0.93
0.97
2.03
1.99
7.00
3.02
8.97
ppm

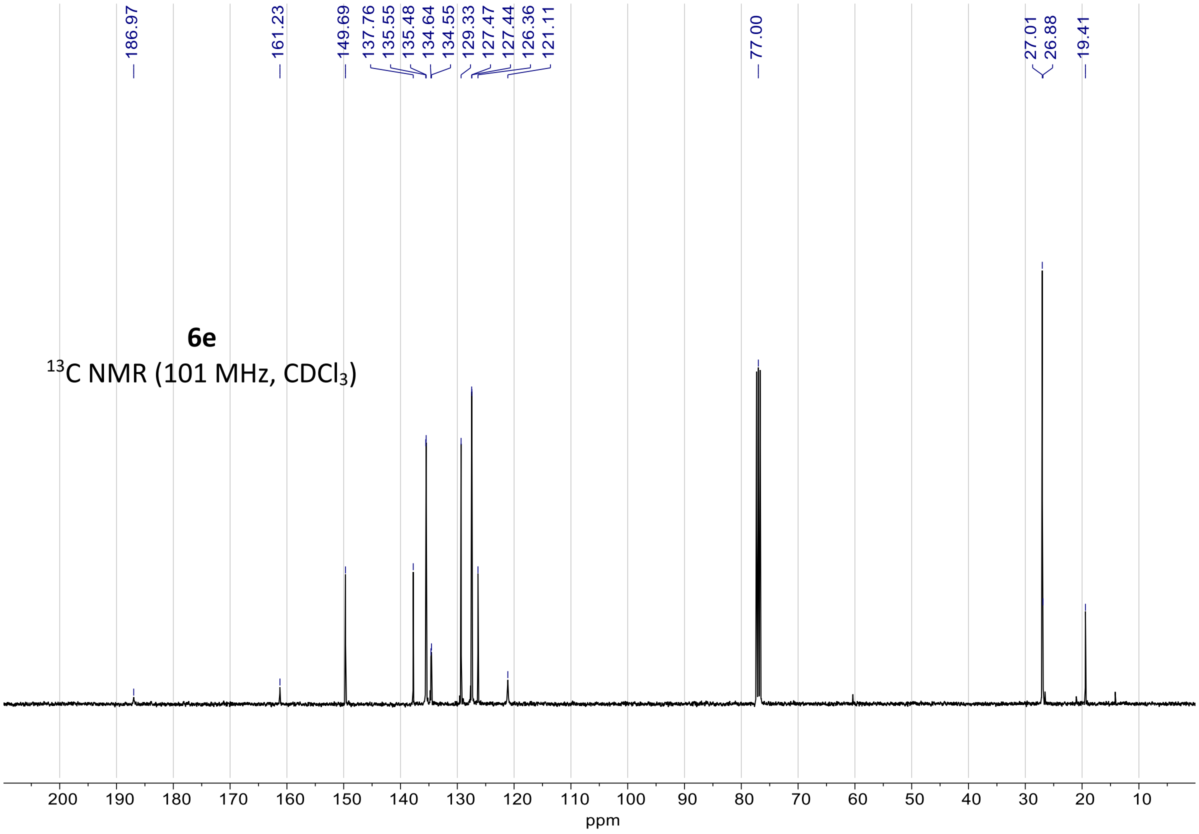
186.97
161.23
149.69
137.76
135.55
135.48
134.64
134.55
129.33
127.47
127.44
126.36
121.11
77.00
27.01
26.88
19.41
6e
13C NMR (101 MHz, CDCl3)
ppm

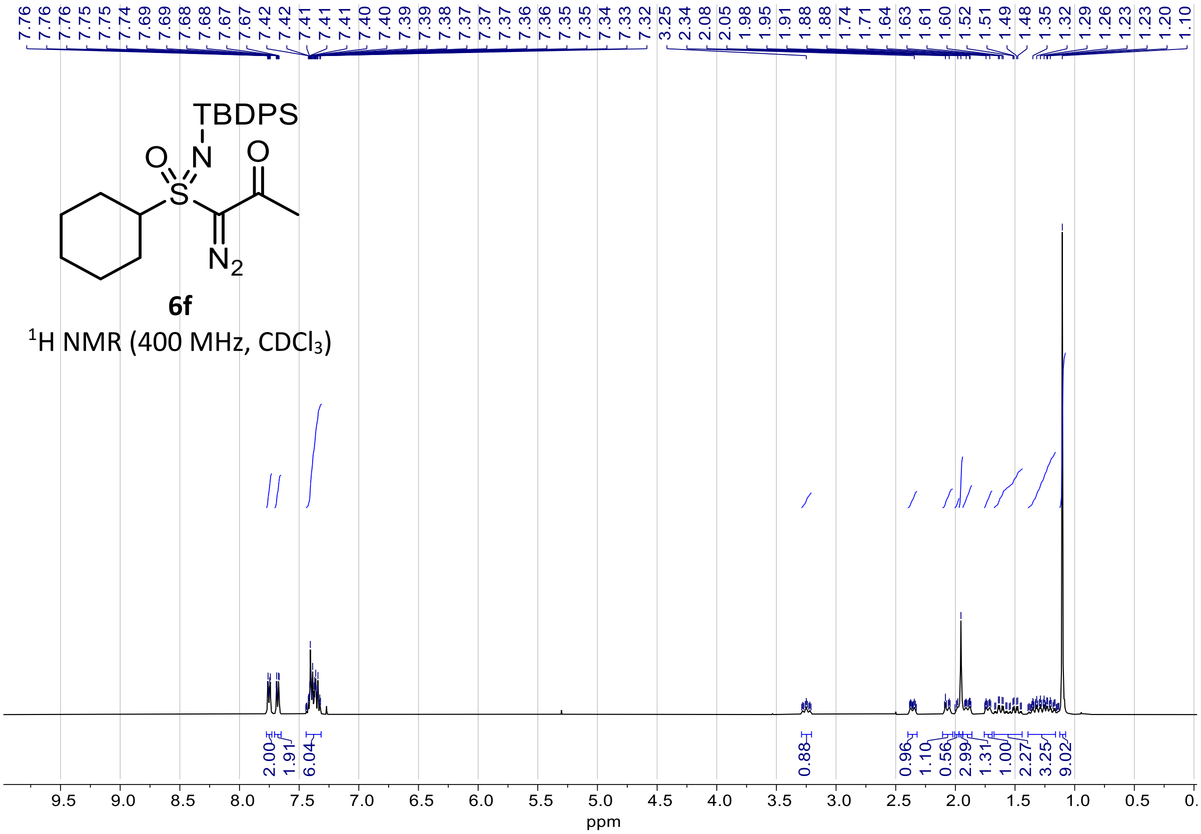
7.76 7.76 7.76 7.75 7.75 7.74 7.69 7.69 7.68 7.68 7.67 7.67 7.42 7.42 7.41 7.41 7.41 7.40 7.40 7.39 7.39 7.38 7.37 7.37 7.37 7.36 7.36 7.35 7.35 7.34 7.33 7.32 3.25 2.34 2.08 2.05 1.98 1.95 1.91 1.88 1.88 1.74 1.71 1.64 1.63 1.61 1.60 1.52 1.51 1.49 1.48 1.35 1.32 1.29 1.26 1.23 1.23 1.20 1.10
TBDPS
O N O
S
N2
6f
¹H NMR (400 MHz, CDCl₃)
2.00 1.91 6.04 0.88 0.96 1.10 0.56 2.99 1.31 1.00 2.27 3.25 9.02
9.5 9.0 8.5 8.0 7.5 7.0 6.5 6.0 5.5 5.0 4.5 4.0 3.5 3.0 2.5 2.0 1.5 1.0 0.5 0.
ppm

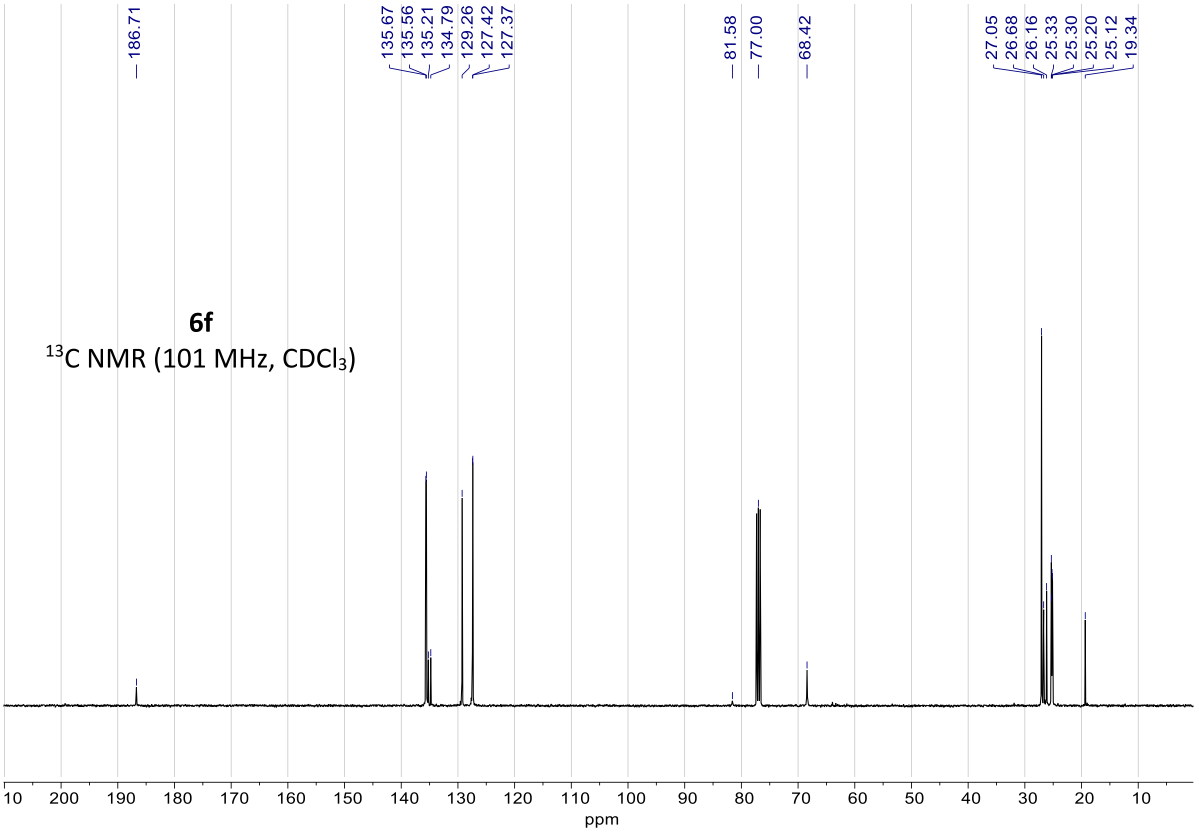
186.71
135.67 135.56 135.21 134.79 129.26 127.42 127.37
81.58 77.00 68.42
27.05 26.68 26.16 25.33 25.30 25.20 25.12 19.34
6f
¹³C NMR (101 MHz, CDCl₃)
10 200 190 180 170 160 150 140 130 120 110 100 90 80 70 60 50 40 30 20 10
ppm

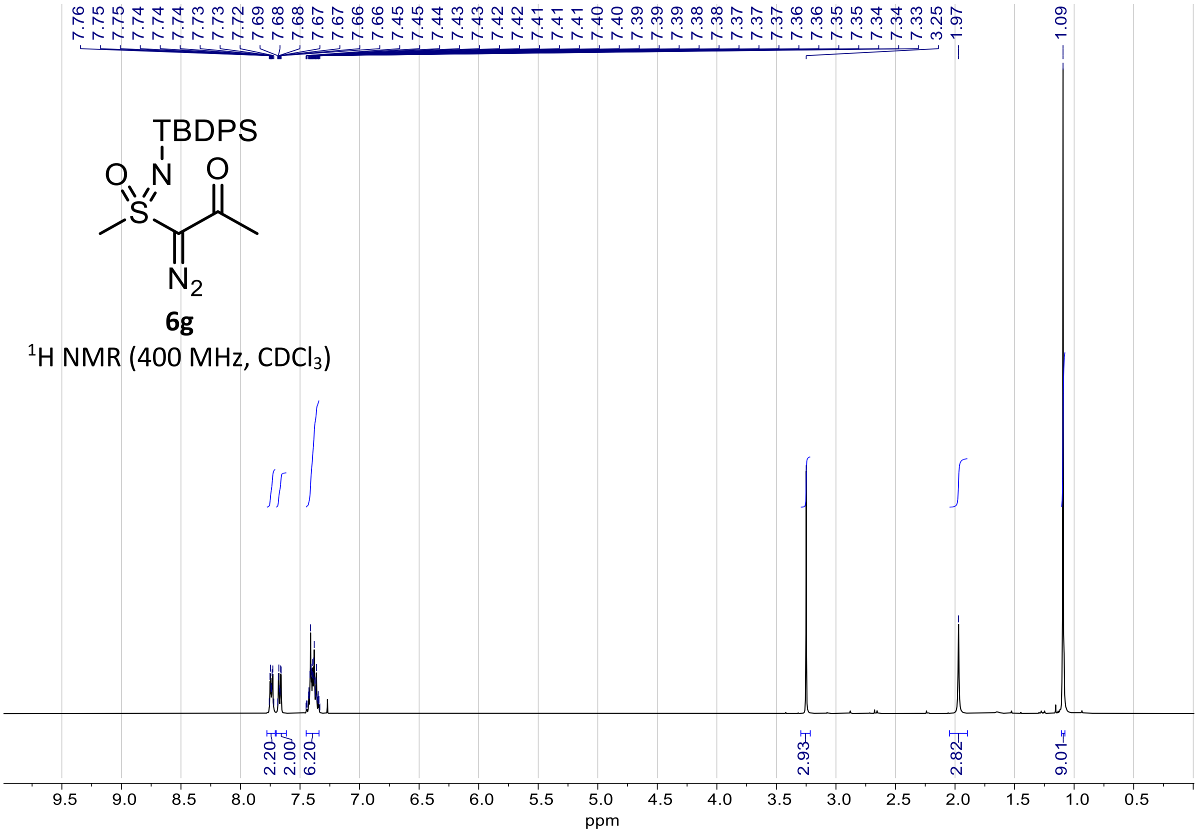

6g

^{1}H NMR (400 MHz, $CDCl_3$)

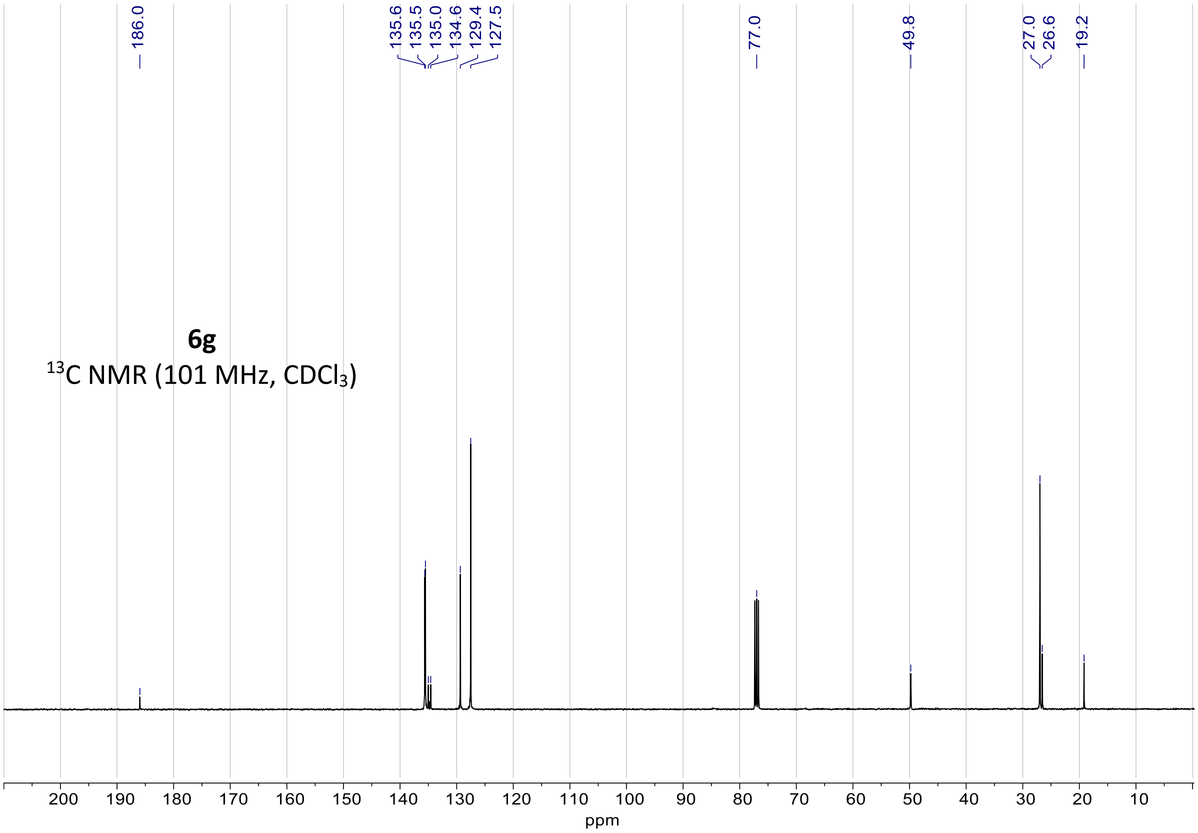

6g

^{13}C NMR (101 MHz, $CDCl_3$)

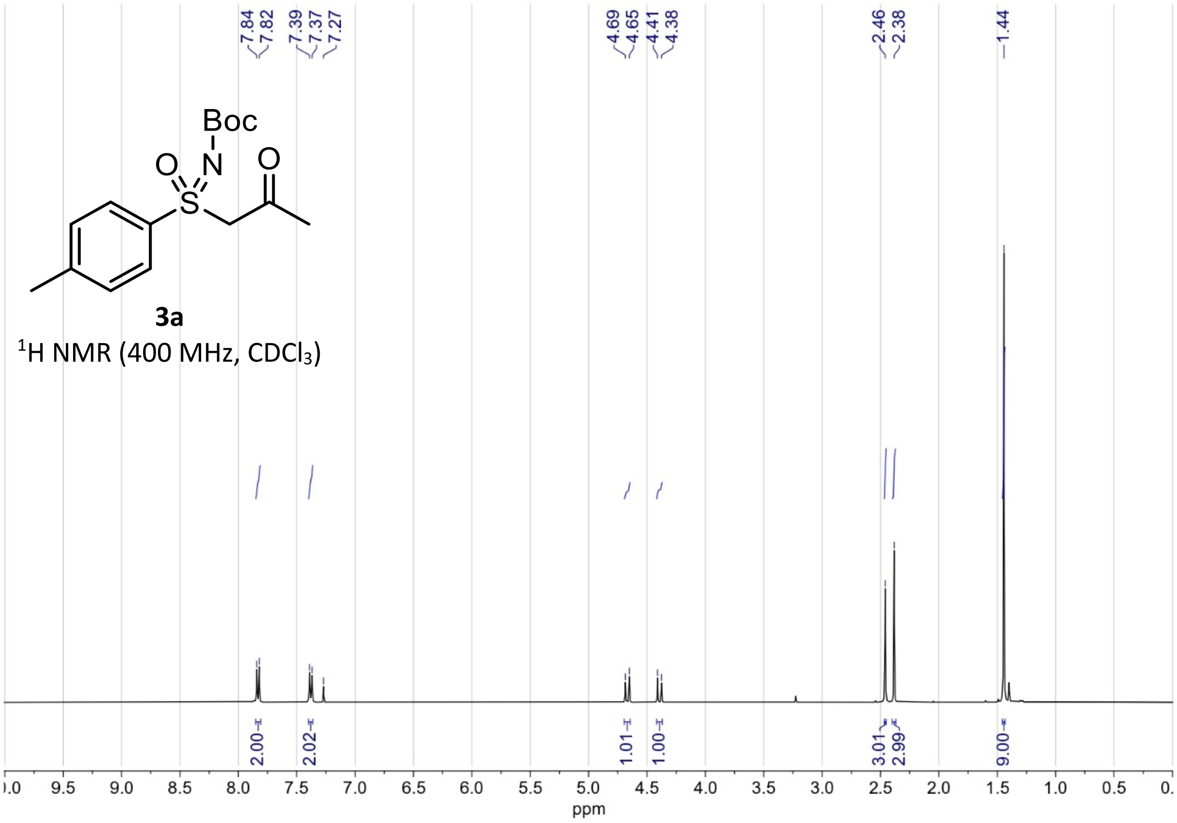
7.84
7.82
7.39
7.37
7.27
4.69
4.65
4.41
4.38
2.46
2.38
1.44
Boc
N
O
S
O
3a
¹H NMR (400 MHz, CDCl₃)
2.00
2.02
1.01
1.00
3.01
2.99
9.00
ppm

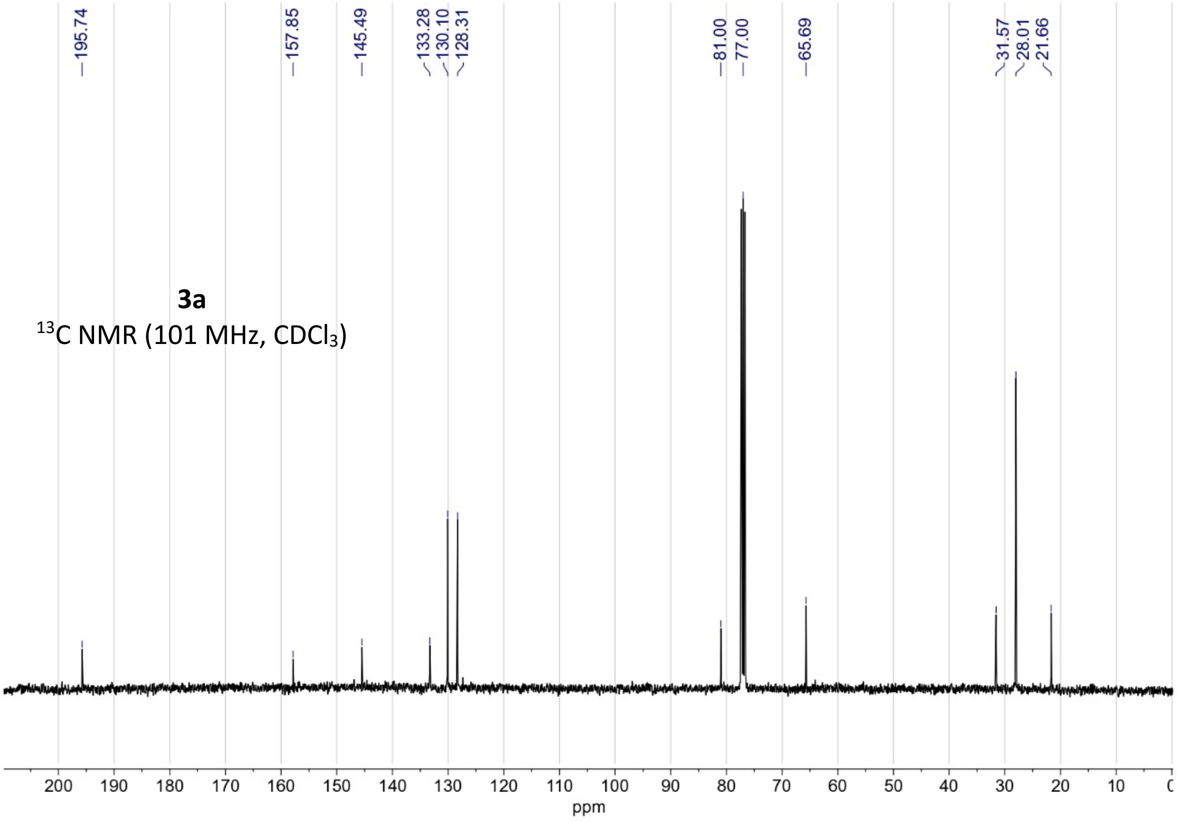
195.74
157.85
145.49
133.28
130.10
128.31
81.00
77.00
65.69
31.57
28.01
21.66
3a
¹³C NMR (101 MHz, CDCl₃)
ppm

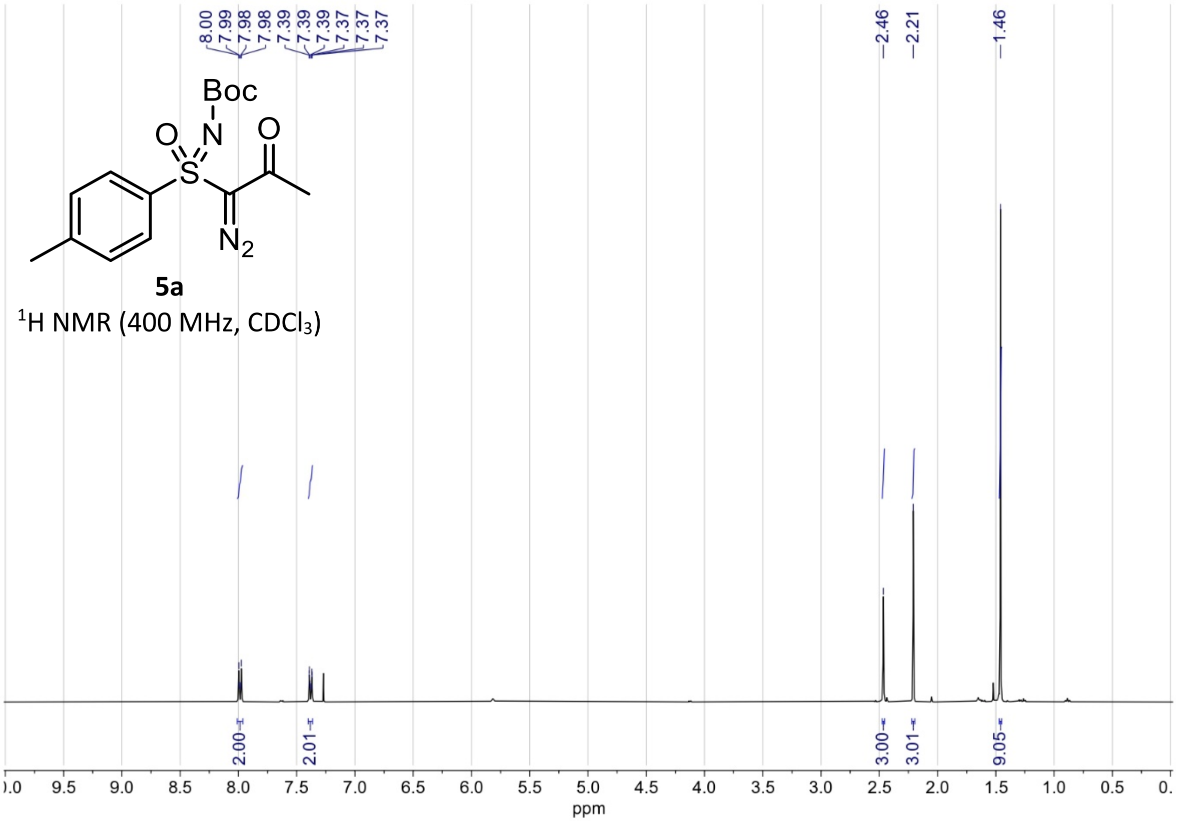

5a

^{1}H NMR (400 MHz, $CDCl_3$)

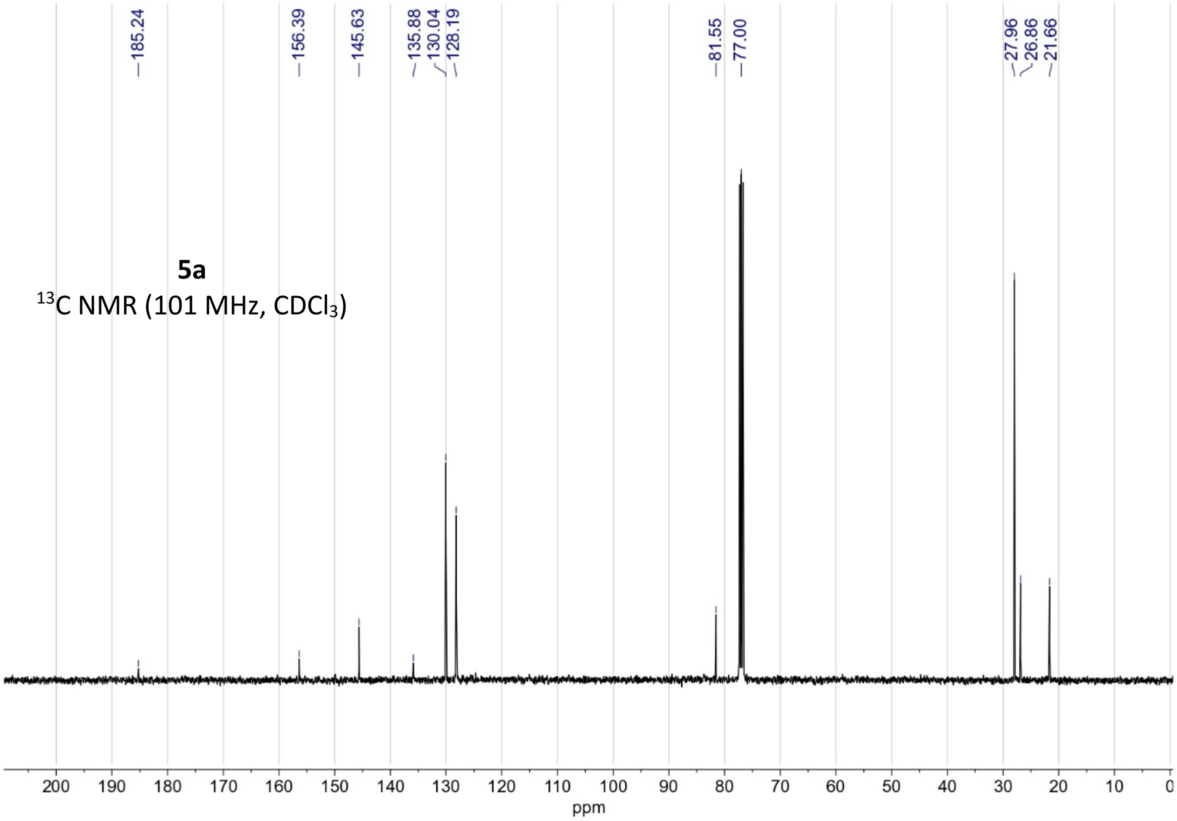

5a

^{13}C NMR (101 MHz, $CDCl_3$)

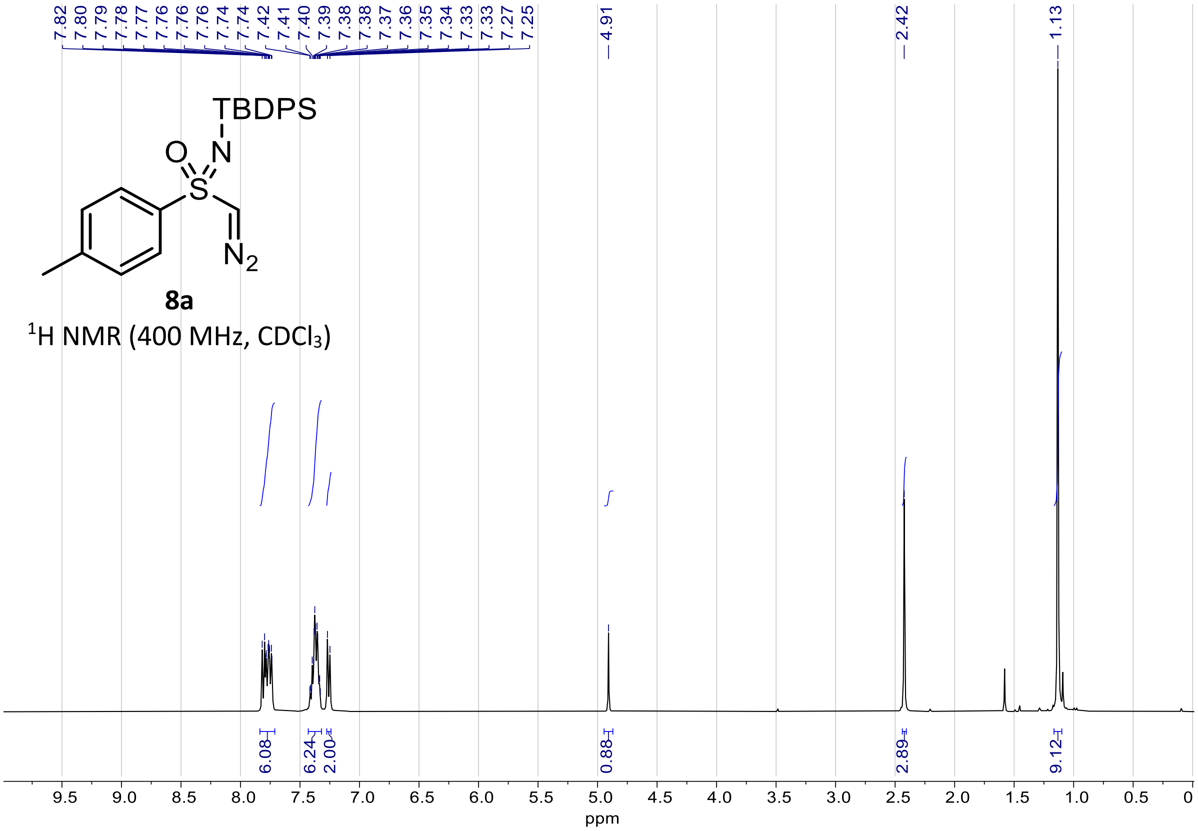

8a

^{1}H NMR (400 MHz, $CDCl_3$)

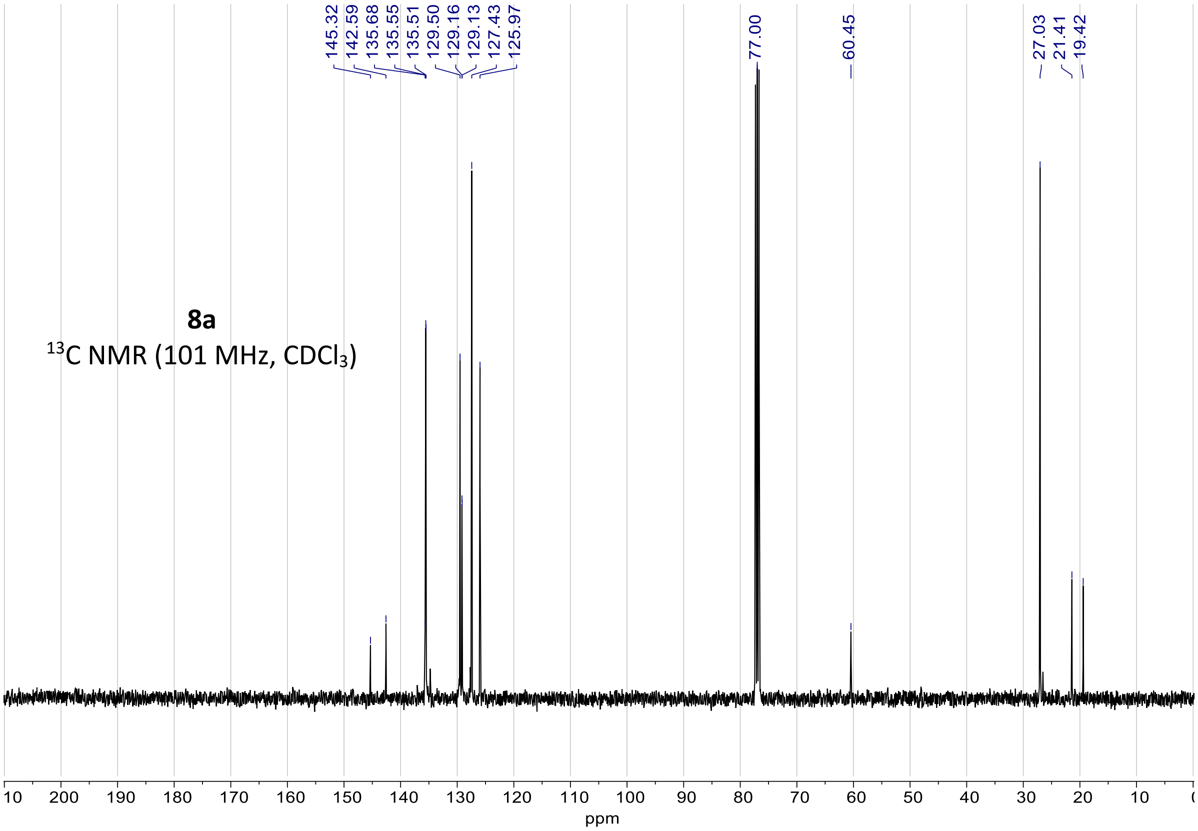

8a

^{13}C NMR (101 MHz, $CDCl_3$)

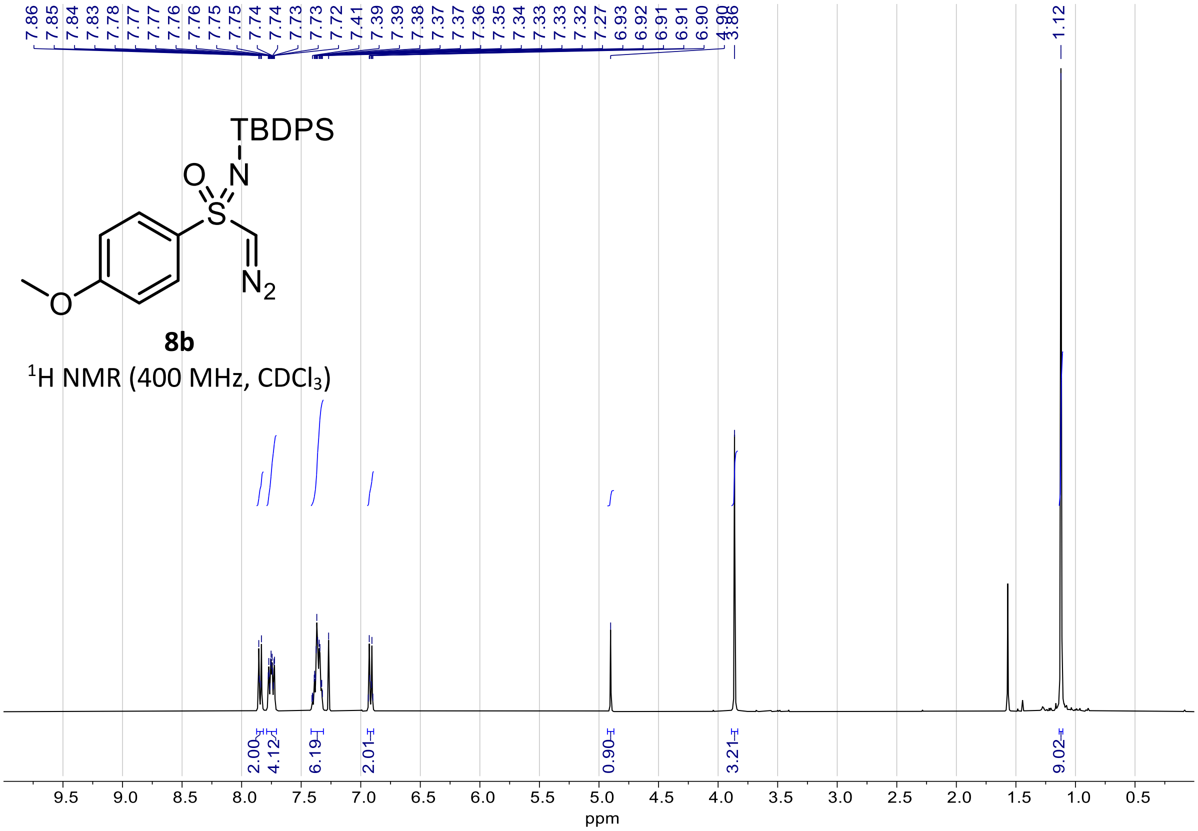

8b
^{1}H NMR (400 MHz, CDCl$_3$)

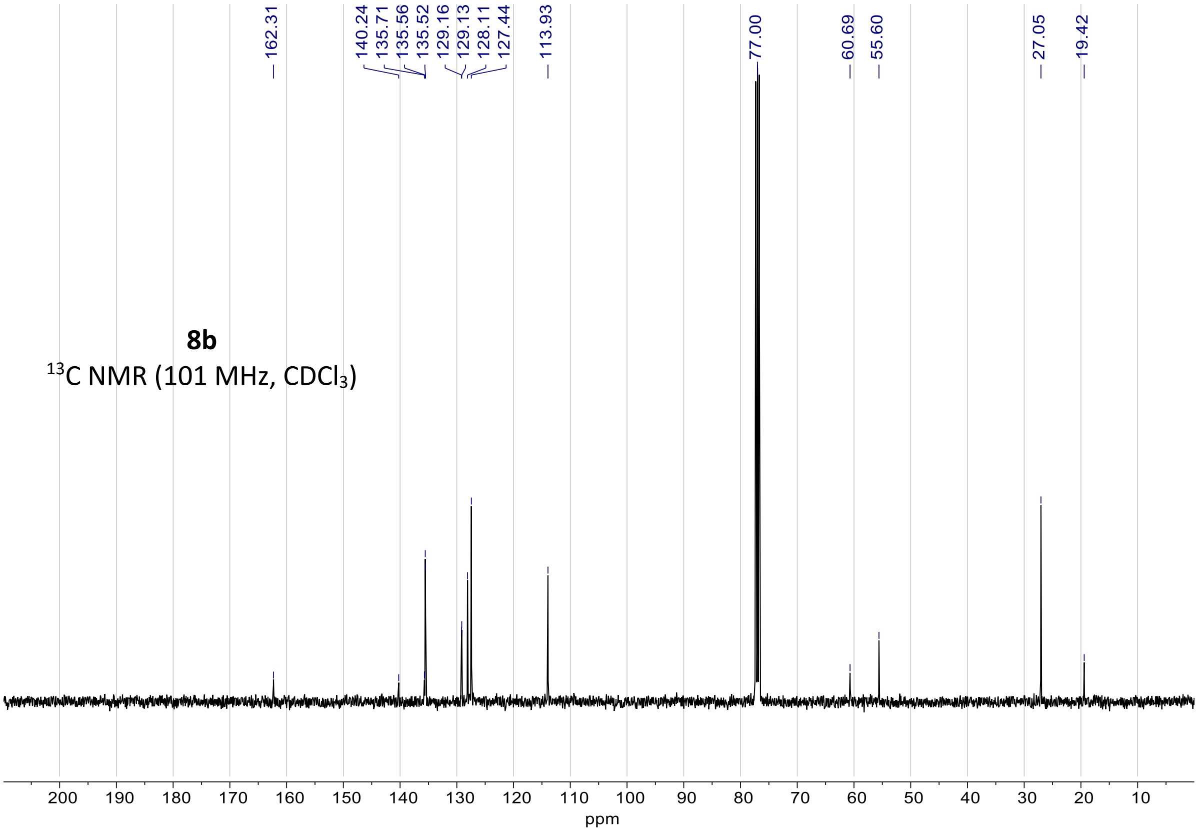

8b
^{13}C NMR (101 MHz, CDCl$_3$)

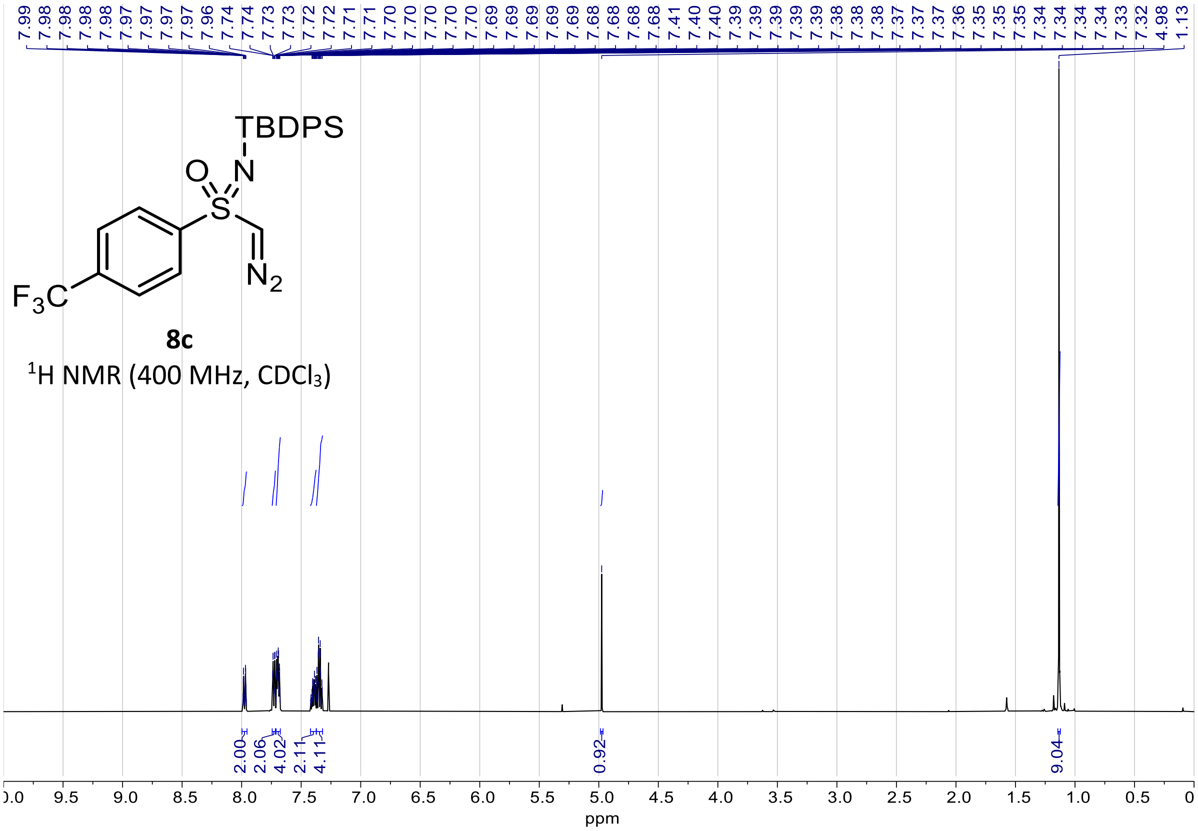
7.99 7.98 7.98 7.98 7.98 7.97 7.97 7.97 7.96 7.74 7.74 7.73 7.73 7.72 7.72 7.71 7.71 7.70 7.70 7.70 7.70 7.70 7.69 7.69 7.69 7.69 7.69 7.68 7.68 7.68 7.68 7.41 7.40 7.40 7.39 7.39 7.39 7.39 7.39 7.38 7.38 7.38 7.37 7.37 7.37 7.36 7.35 7.35 7.35 7.34 7.34 7.34 7.34 7.33 7.32 4.98 1.13
TBDPS
O
N
S
N_2
F_3C
8c
^{1}H NMR (400 MHz, $CDCl_3$)
2.00 2.06 4.02 2.11 4.11 0.92 9.04
ppm

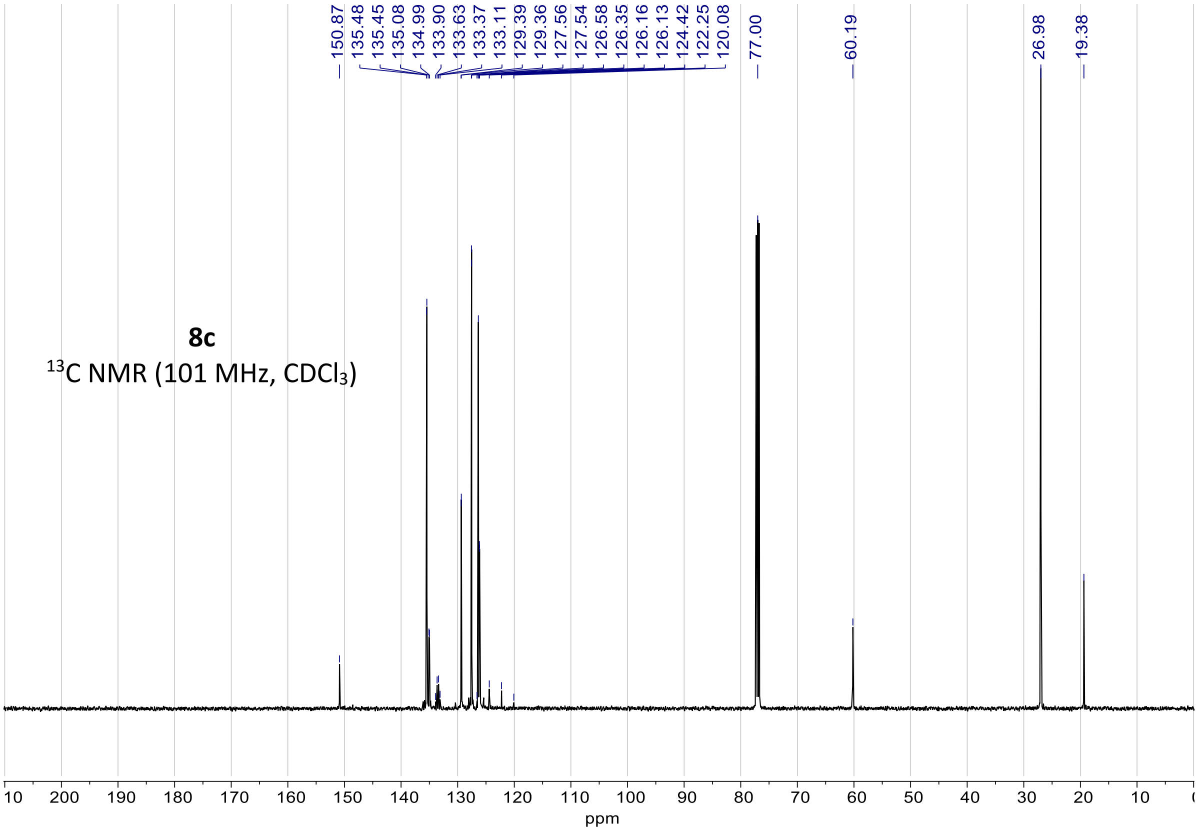
150.87 135.48 135.45 135.08 134.99 133.90 133.63 133.37 133.11 129.39 129.36 127.56 127.54 126.58 126.35 126.16 126.13 124.42 122.25 120.08 77.00 60.19 26.98 19.38
8c
^{13}C NMR (101 MHz, $CDCl_3$)
ppm

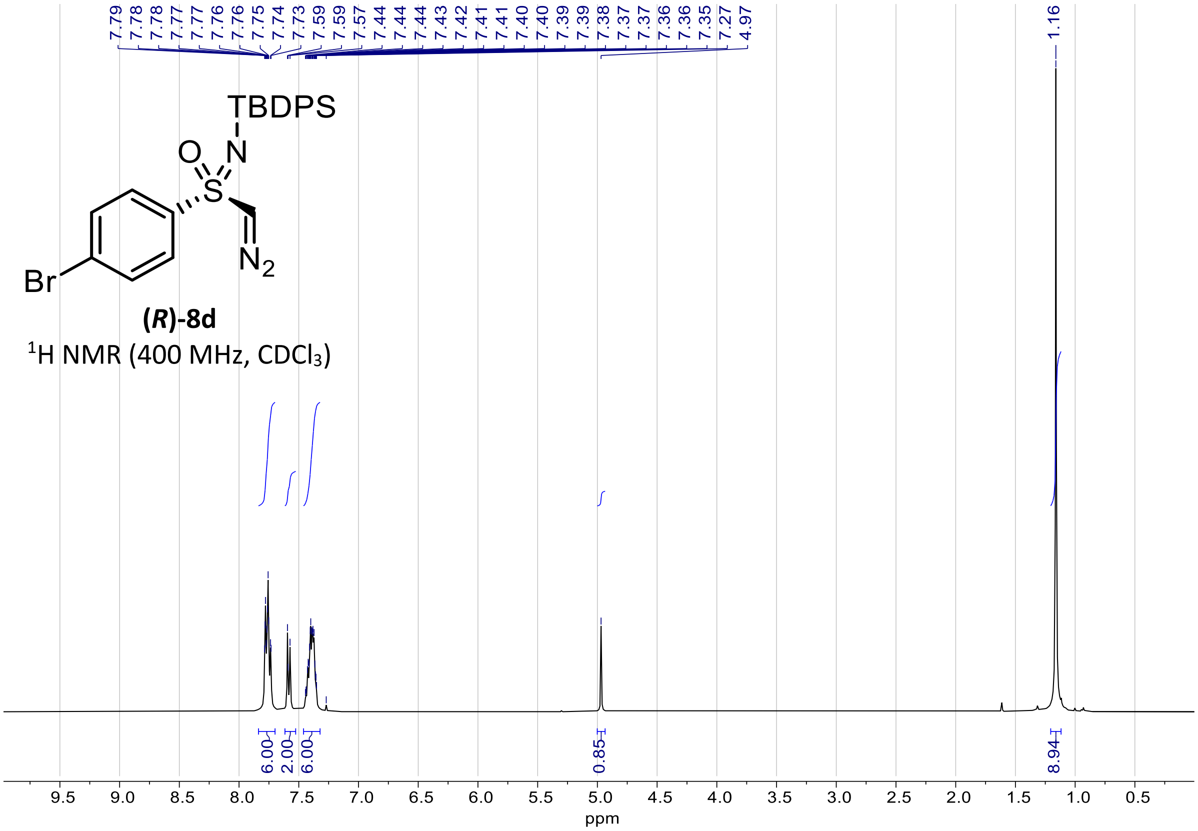
TBDPS
N
O
S
Br
N2
(*R*)-8d
1H NMR (400 MHz, CDCl3)
ppm

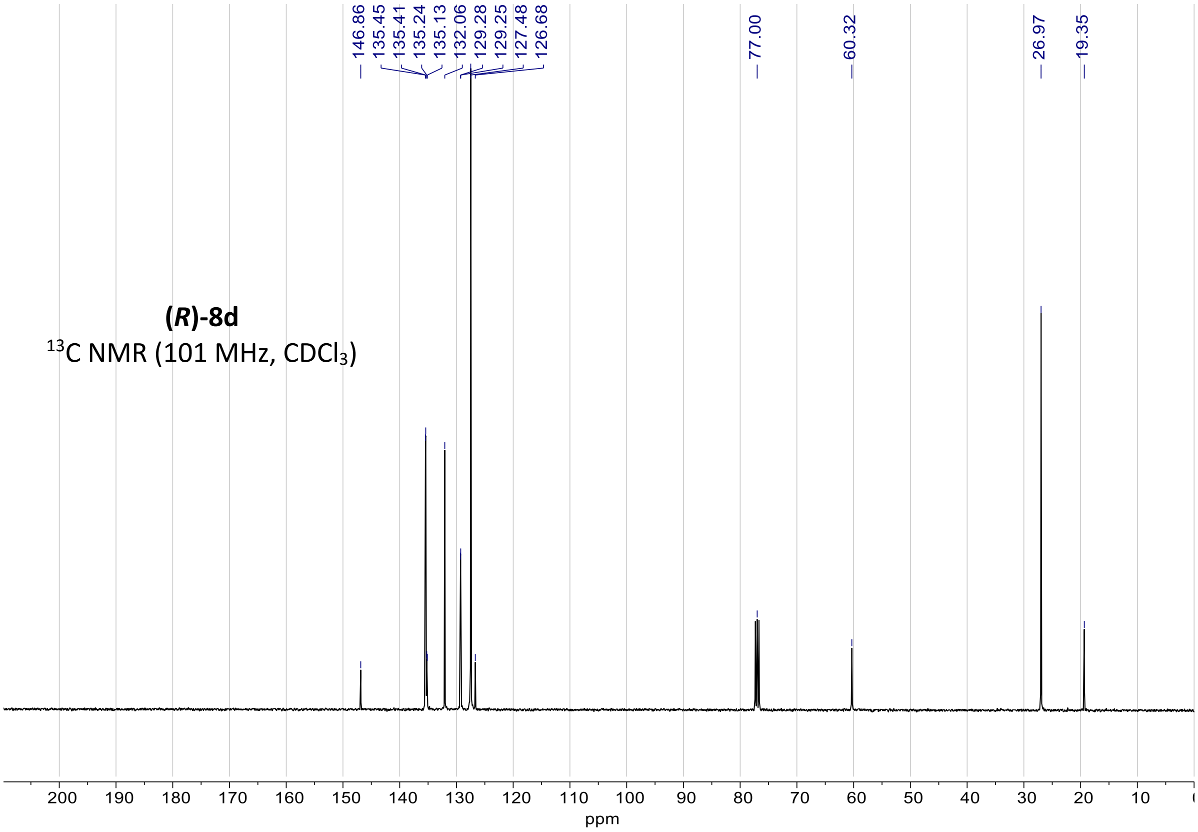
(*R*)-8d
13C NMR (101 MHz, CDCl3)
ppm

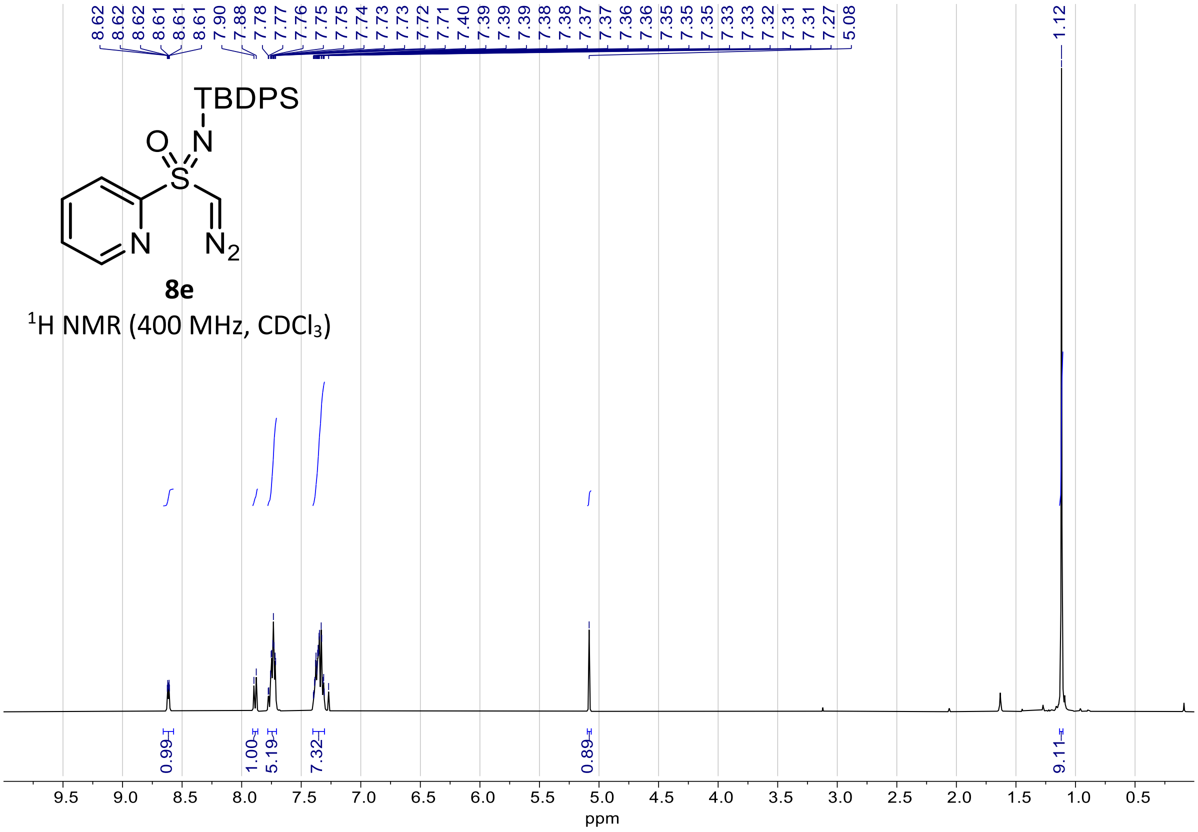
8.62
8.62
8.62
8.61
8.61
8.61
7.90
7.88
7.78
7.77
7.76
7.75
7.75
7.74
7.73
7.73
7.72
7.71
7.40
7.39
7.39
7.39
7.38
7.38
7.37
7.37
7.36
7.36
7.35
7.35
7.35
7.33
7.33
7.32
7.31
7.31
7.27
5.08
1.12
TBDPS
O
N
S
N
N2
8e
1H NMR (400 MHz, CDCl3)
0.99
1.00
5.19
7.32
0.89
9.11
9.5
9.0
8.5
8.0
7.5
7.0
6.5
6.0
5.5
5.0
4.5
4.0
3.5
3.0
2.5
2.0
1.5
1.0
0.5
ppm

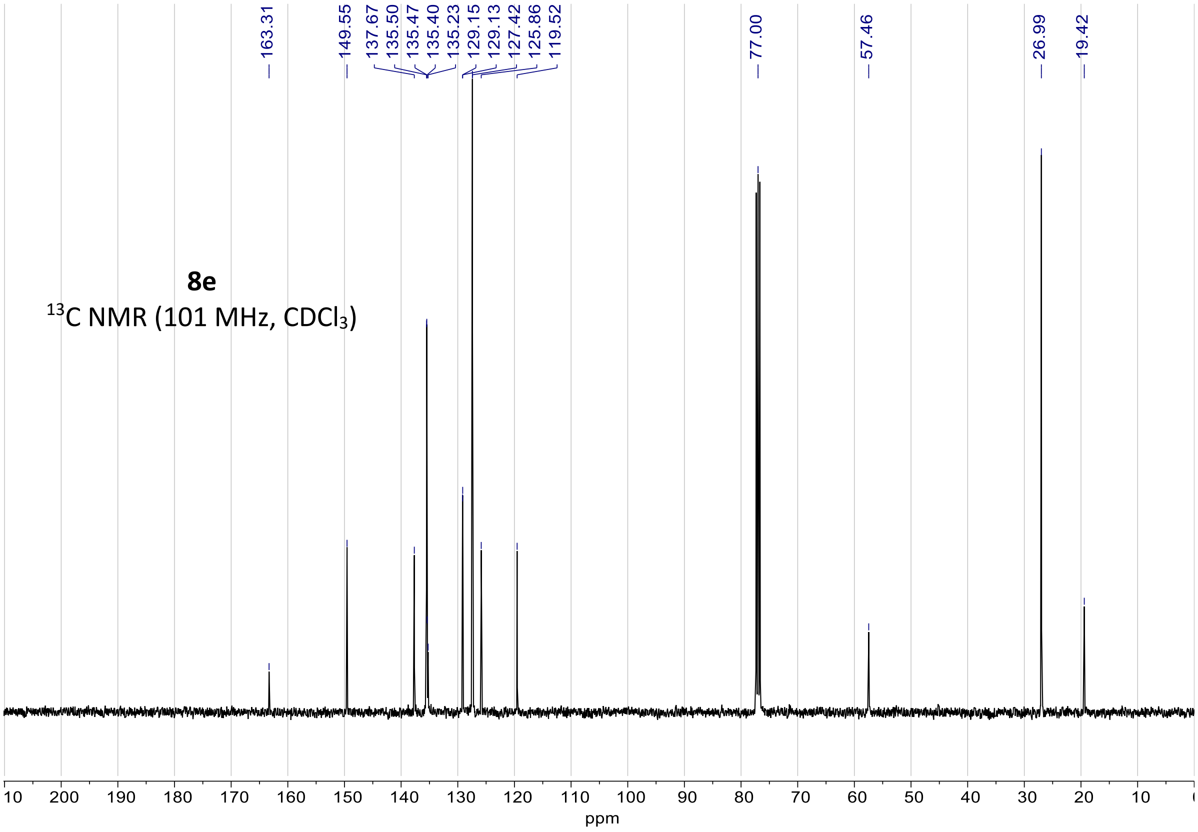
163.31
149.55
137.67
135.50
135.47
135.40
135.23
129.15
129.13
127.42
125.86
119.52
77.00
57.46
26.99
19.42
8e
13C NMR (101 MHz, CDCl3)
10
200
190
180
170
160
150
140
130
120
110
100
90
80
70
60
50
40
30
20
10
ppm

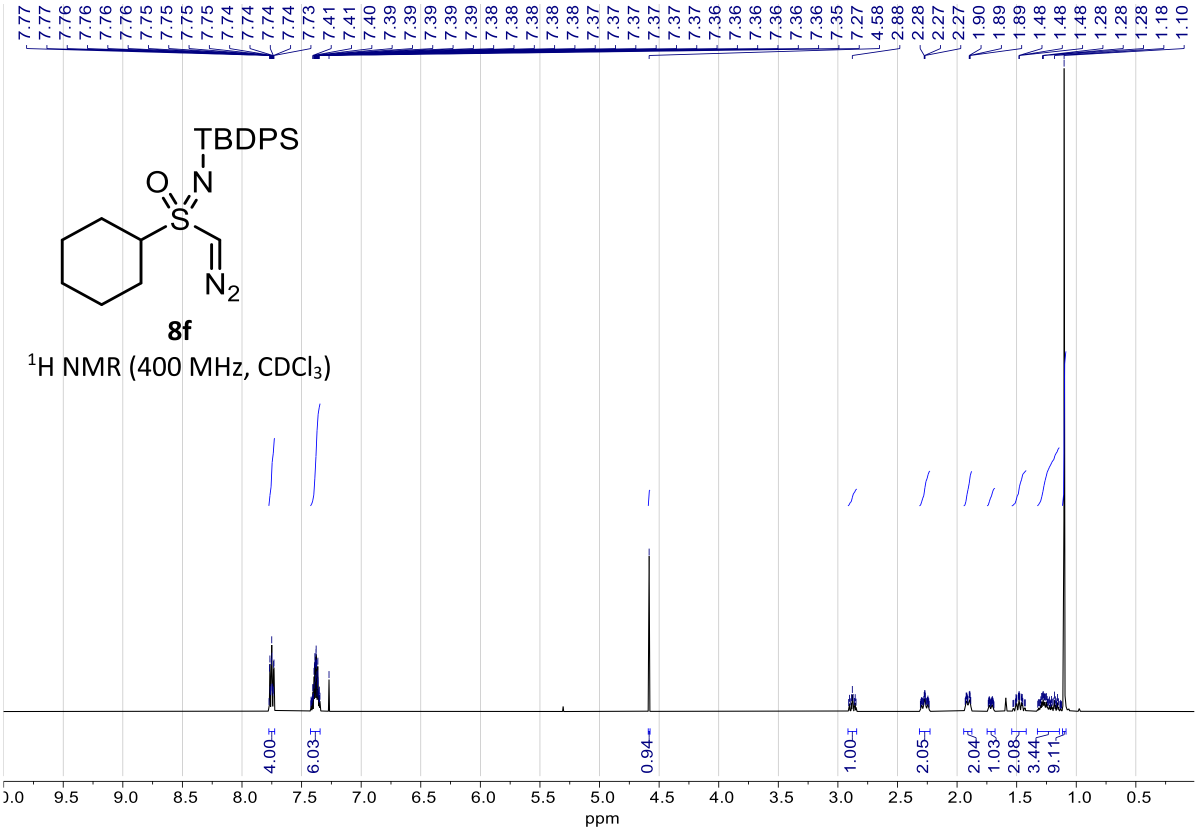
TBDPS
O
N
S
N2
8f
1H NMR (400 MHz, CDCl3)
ppm

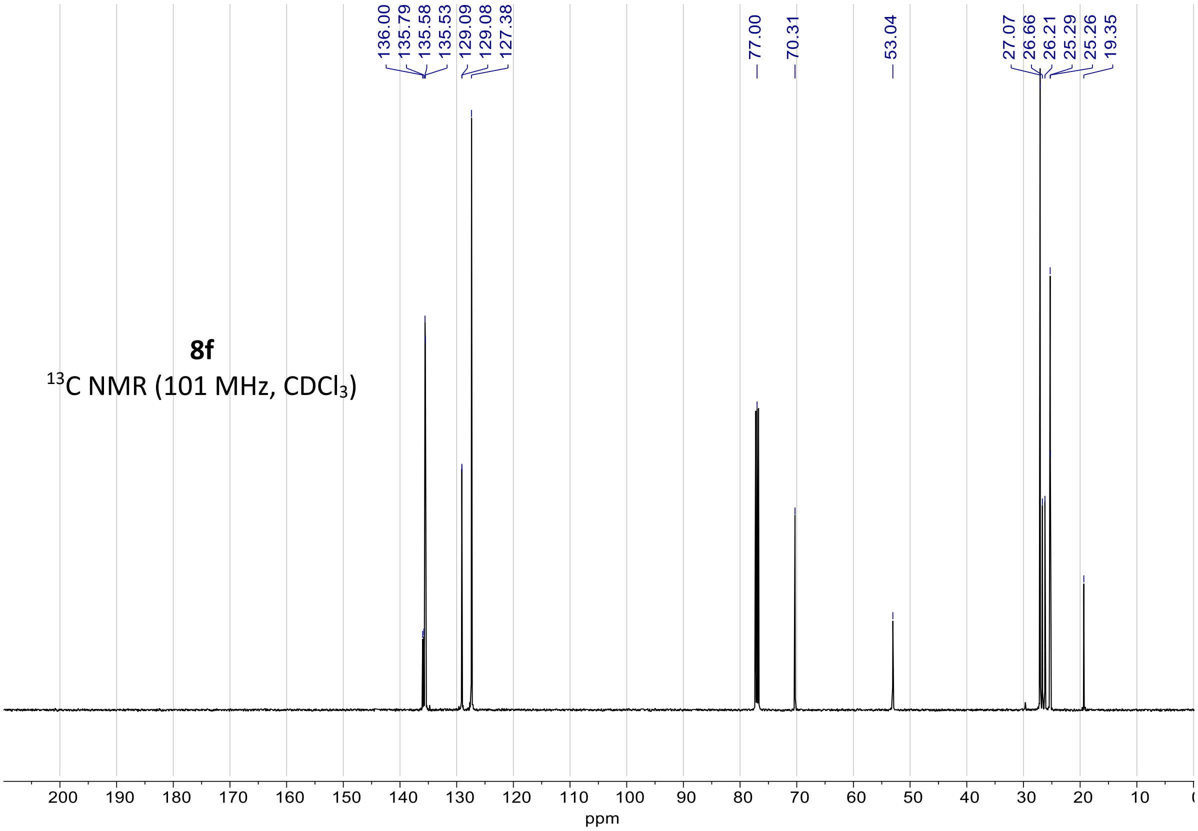
8f
13C NMR (101 MHz, CDCl3)
136.00
135.79
135.58
135.53
129.09
129.08
127.38
77.00
70.31
53.04
27.07
26.66
26.21
25.29
25.26
19.35
ppm

8g

^{1}H NMR (400 MHz, $CDCl_3$)

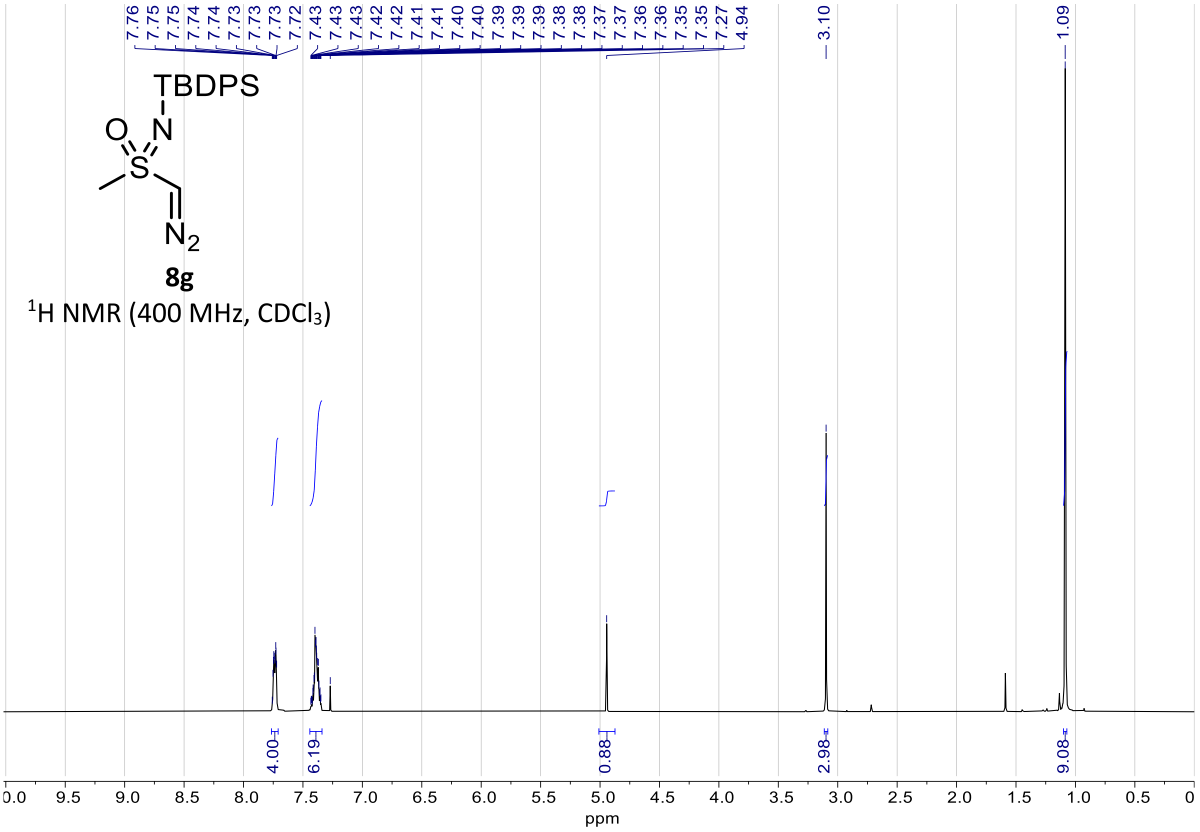

8g

^{13}C NMR (101 MHz, $CDCl_3$)

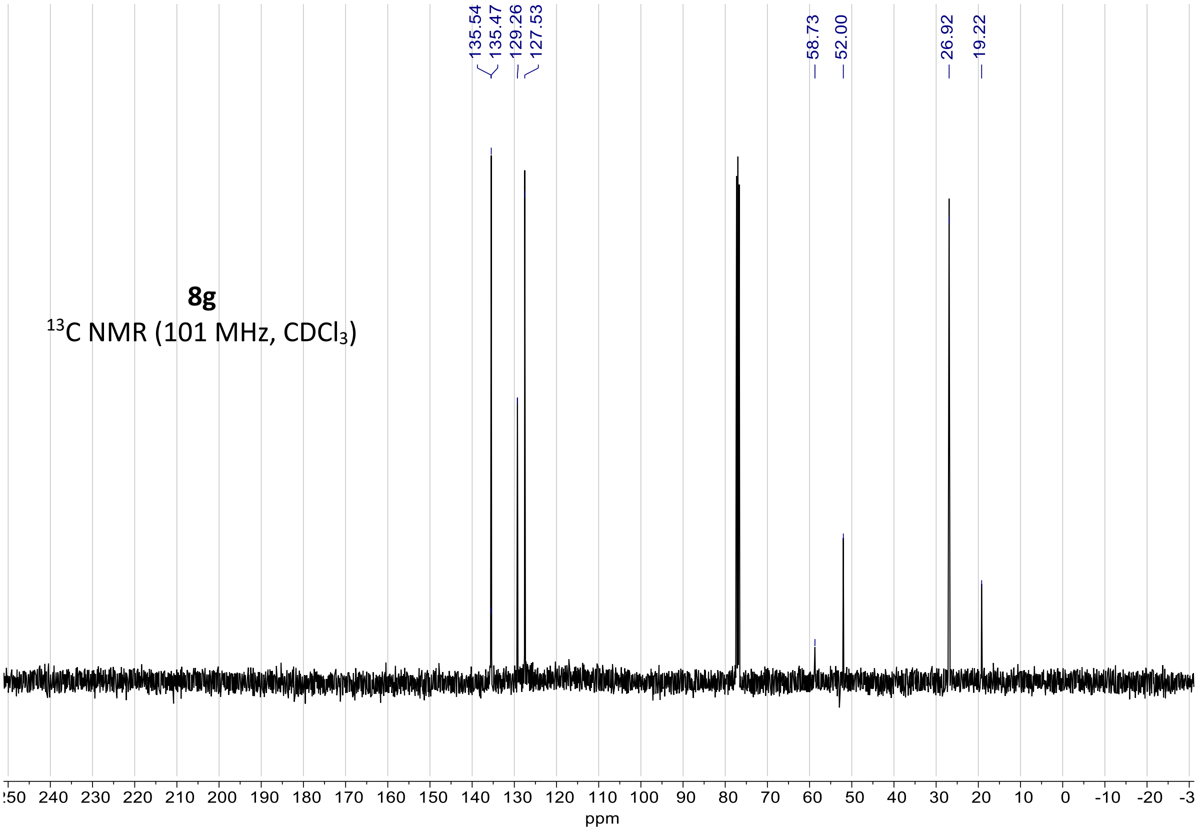

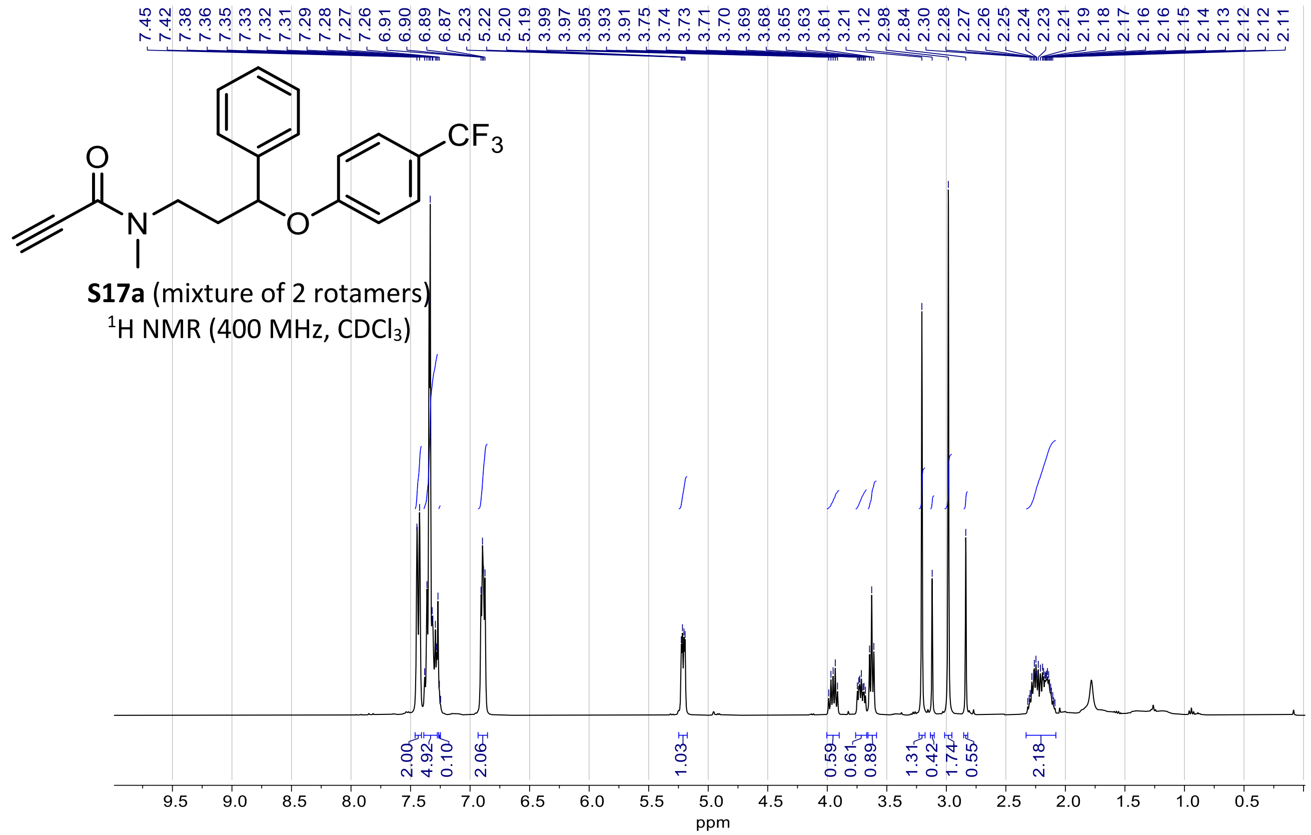
7.45 7.42 7.38 7.36 7.35 7.33 7.32 7.31 7.29 7.28 7.27 7.26 6.91 6.90 6.89 6.87 5.23 5.22 5.20 5.19 3.99 3.97 3.95 3.93 3.91 3.75 3.74 3.73 3.71 3.70 3.69 3.68 3.65 3.63 3.61 3.21 3.12 2.98 2.84 2.30 2.28 2.27 2.26 2.25 2.24 2.23 2.21 2.19 2.18 2.17 2.16 2.16 2.15 2.14 2.13 2.12 2.12 2.11
CF3
O
N
O
S17a (mixture of 2 rotamers)
1H NMR (400 MHz, CDCl3)
2.00 4.92 0.10 2.06 1.03 0.59 0.61 0.89 1.31 0.42 1.74 0.55 2.18
9.5 9.0 8.5 8.0 7.5 7.0 6.5 6.0 5.5 5.0 4.5 4.0 3.5 3.0 2.5 2.0 1.5 1.0 0.5
ppm

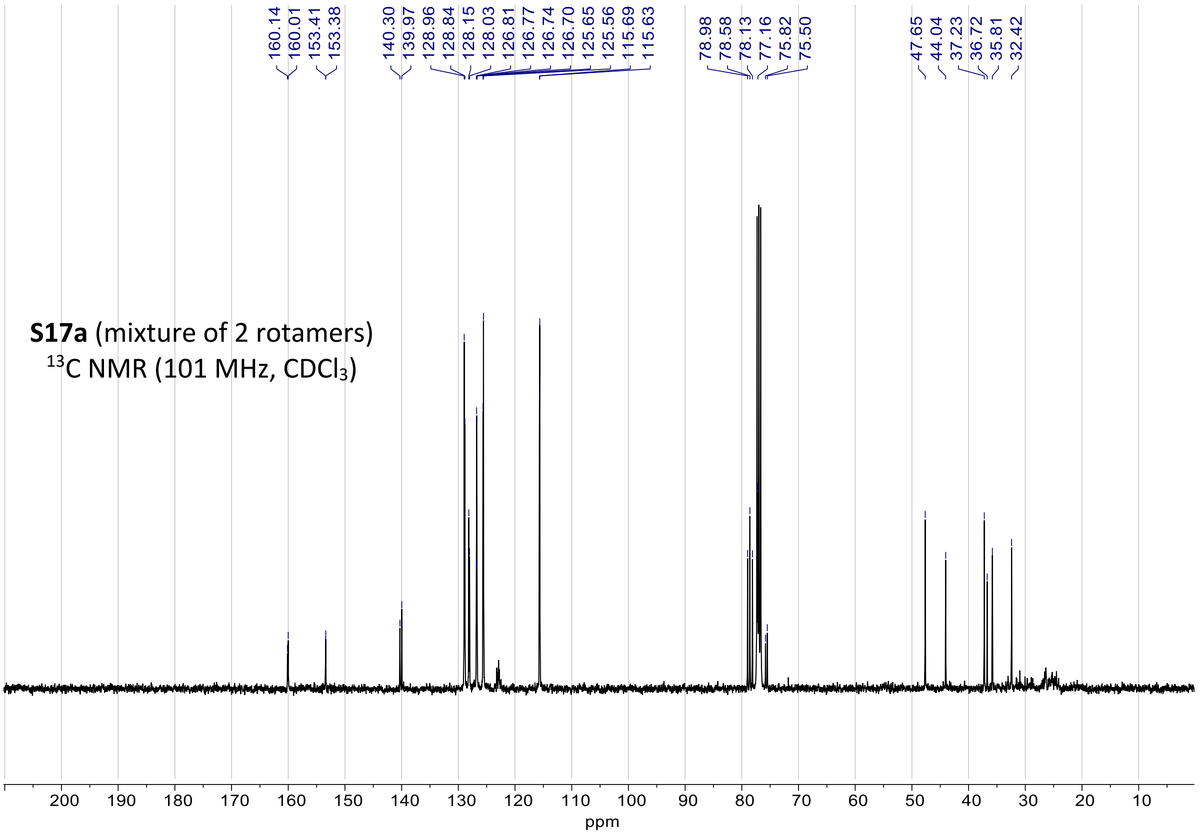
160.14 160.01 153.41 153.38 140.30 139.97 128.96 128.84 128.15 128.03 126.81 126.77 126.74 126.70 125.65 125.56 115.69 115.63 78.98 78.58 78.13 77.16 75.82 75.50 47.65 44.04 37.23 36.72 35.81 32.42
S17a (mixture of 2 rotamers)
13C NMR (101 MHz, CDCl3)
200 190 180 170 160 150 140 130 120 110 100 90 80 70 60 50 40 30 20 10
ppm

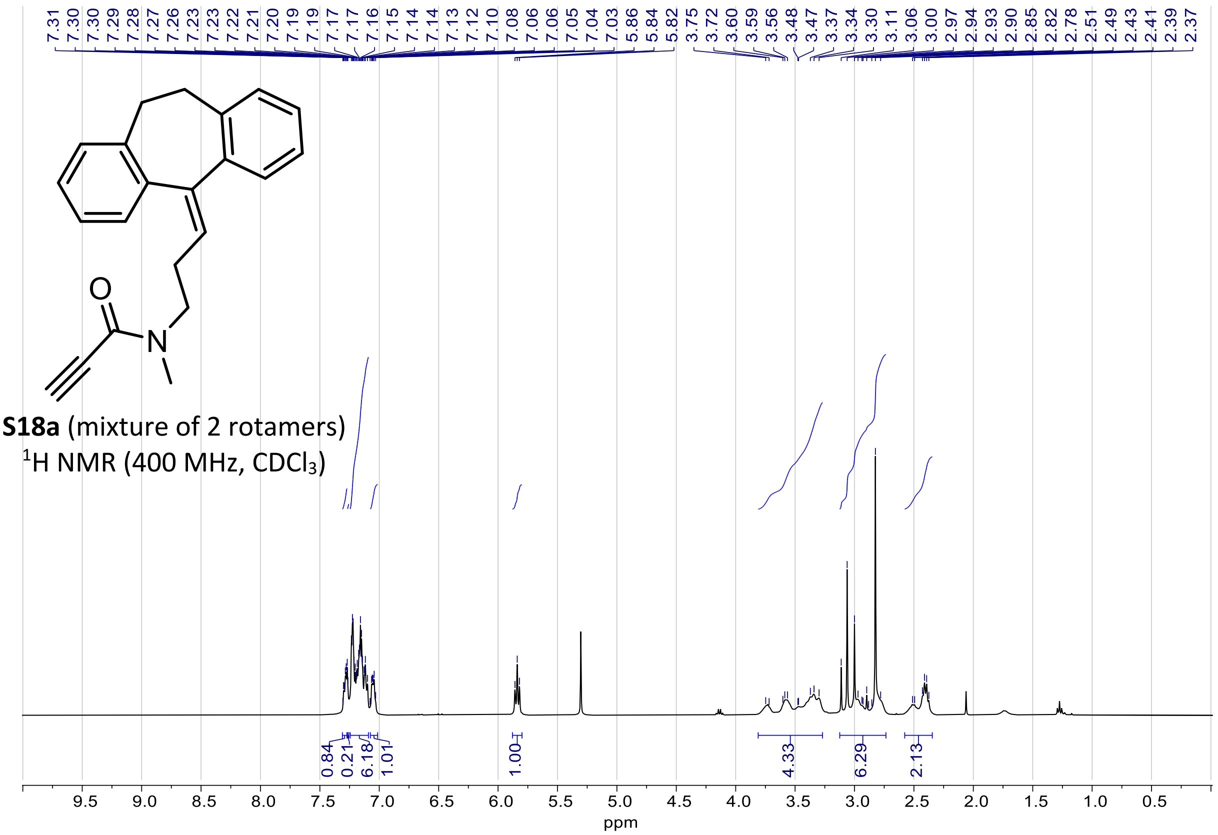
7.31 7.30 7.30 7.29 7.28 7.27 7.26 7.23 7.23 7.22 7.21 7.20 7.19 7.19 7.17 7.17 7.16 7.15 7.14 7.14 7.13 7.12 7.10 7.08 7.06 7.06 7.05 7.04 7.03 5.86 5.84 5.82 3.75 3.72 3.60 3.59 3.56 3.48 3.47 3.37 3.34 3.30 3.11 3.06 3.00 2.97 2.94 2.93 2.90 2.85 2.82 2.78 2.51 2.49 2.43 2.41 2.39 2.37
O
N
S18a (mixture of 2 rotamers)
^{1}H NMR (400 MHz, CDCl$_3$)
0.84 0.21 6.18 1.01 1.00 4.33 6.29 2.13
9.5 9.0 8.5 8.0 7.5 7.0 6.5 6.0 5.5 5.0 4.5 4.0 3.5 3.0 2.5 2.0 1.5 1.0 0.5
ppm

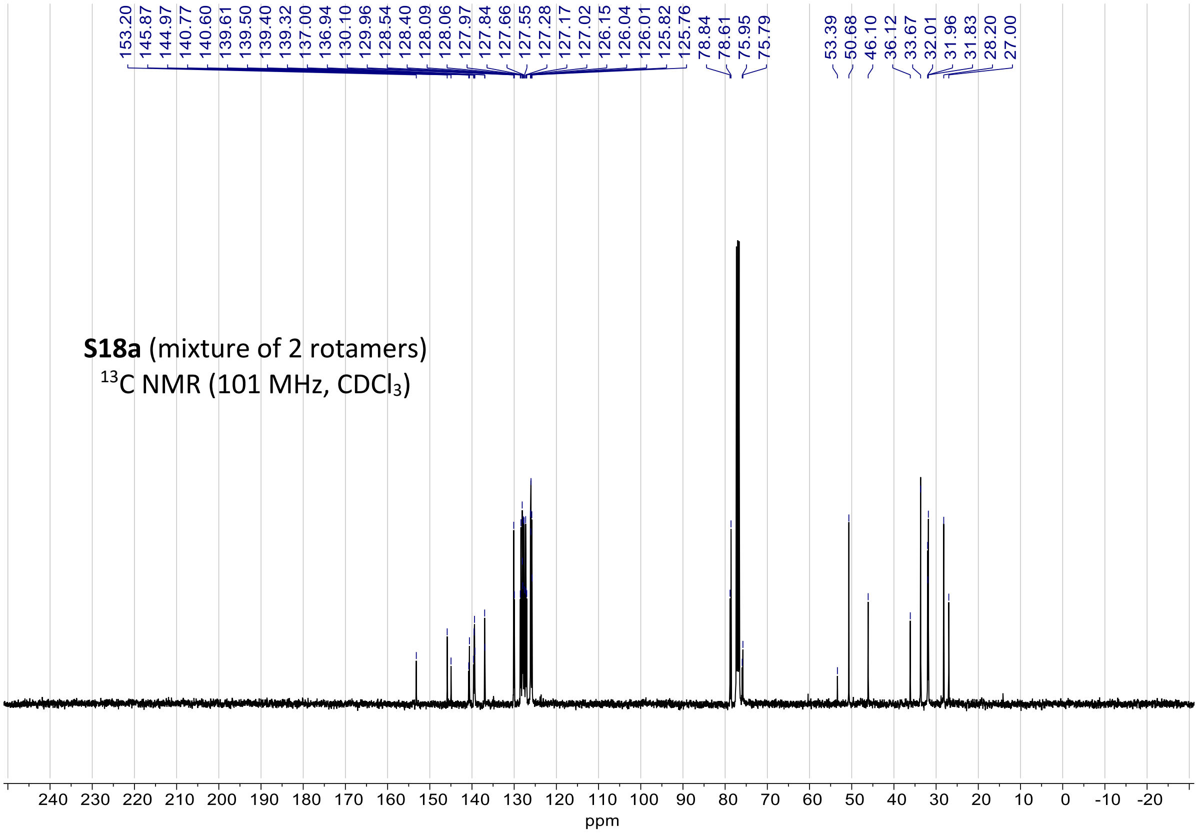
153.20 145.87 144.97 140.77 140.60 139.61 139.50 139.40 139.32 137.00 136.94 130.10 129.96 128.54 128.40 128.09 128.06 127.97 127.84 127.66 127.55 127.28 127.17 127.02 126.15 126.04 126.01 125.82 125.76 78.84 78.61 75.95 75.79 53.39 50.68 46.10 36.12 33.67 32.01 31.96 31.83 28.20 27.00
S18a (mixture of 2 rotamers)
^{13}C NMR (101 MHz, CDCl$_3$)
240 230 220 210 200 190 180 170 160 150 140 130 120 110 100 90 80 70 60 50 40 30 20 10 0 -10 -20
ppm

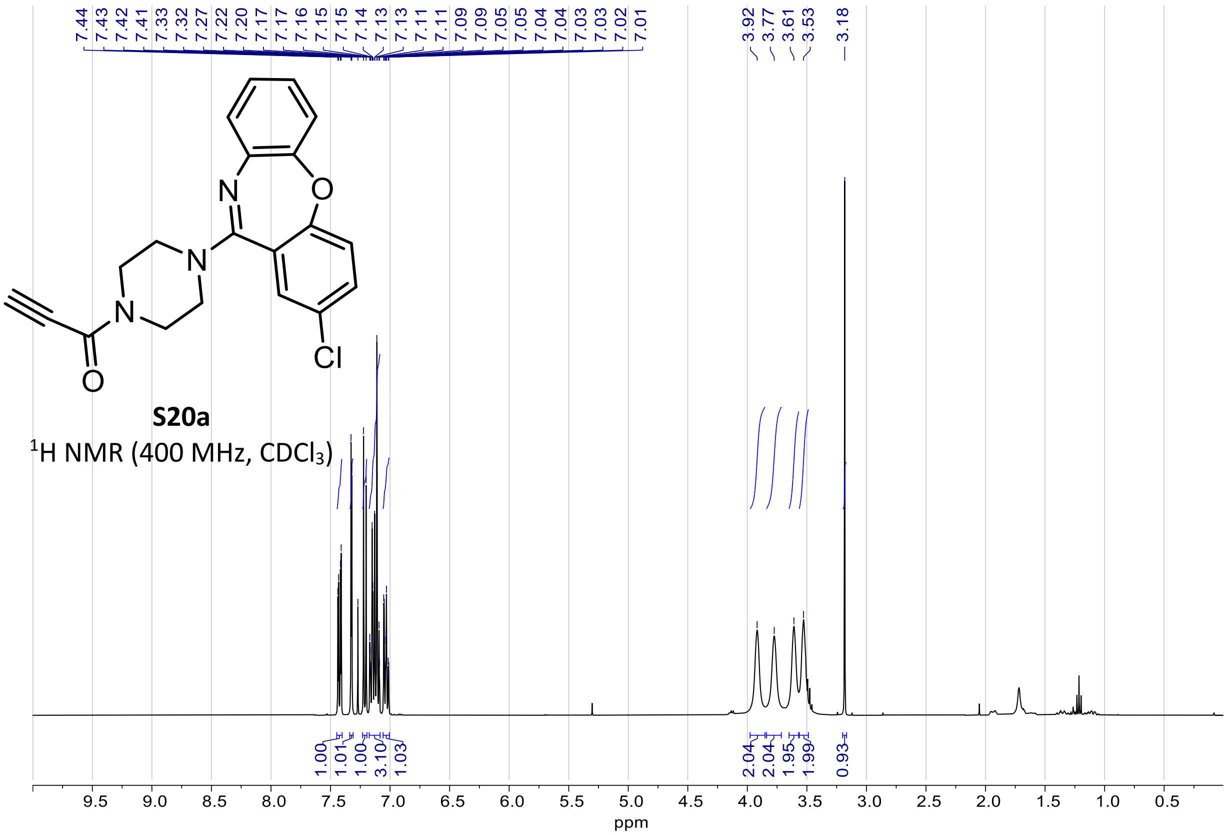
7.44 7.43 7.42 7.41 7.33 7.32 7.27 7.22 7.20 7.17 7.17 7.16 7.15 7.15 7.14 7.13 7.13 7.11 7.11 7.09 7.09 7.05 7.05 7.04 7.04 7.03 7.03 7.02 7.01
3.92 3.77 3.61 3.53
3.18
N
O
N
N
Cl
O
S20a
¹H NMR (400 MHz, CDCl₃)
1.00 1.01 1.00 3.10 1.03
2.04 2.04 1.95 1.99
0.93
9.5 9.0 8.5 8.0 7.5 7.0 6.5 6.0 5.5 5.0 4.5 4.0 3.5 3.0 2.5 2.0 1.5 1.0 0.5
ppm

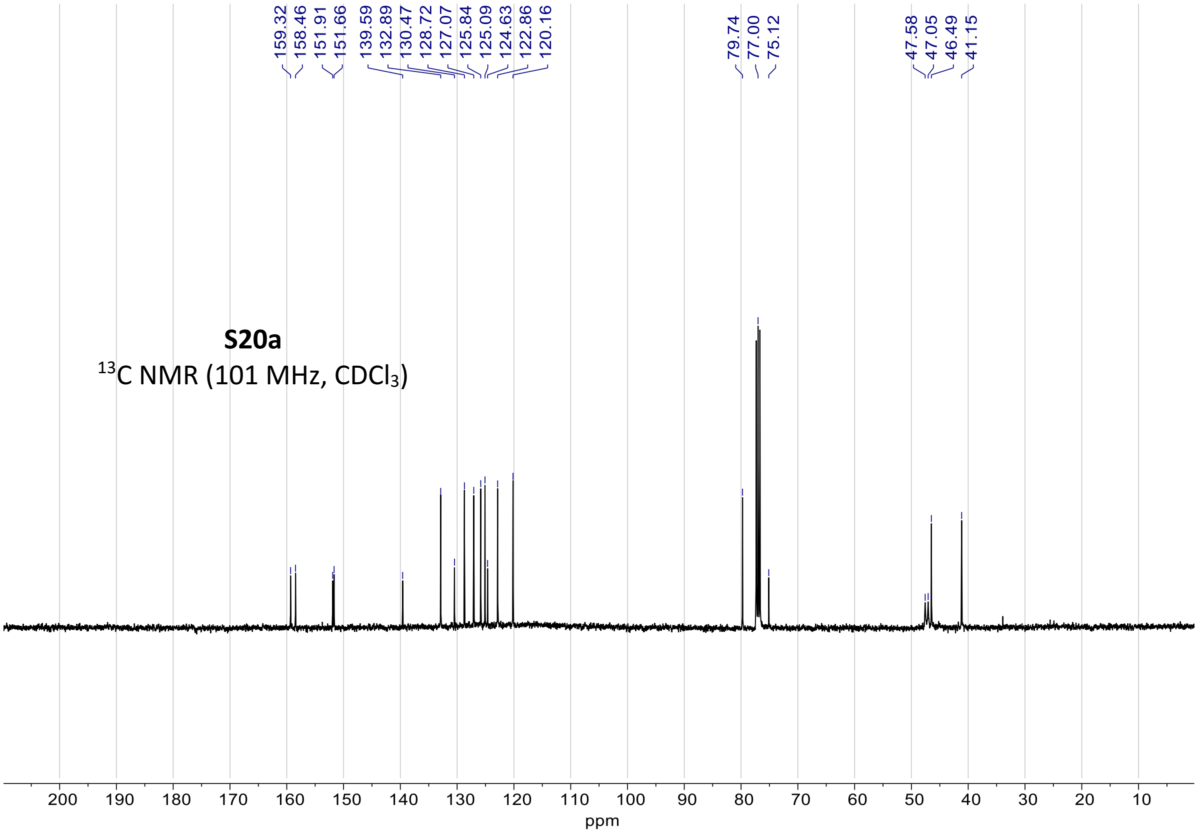
159.32 158.46 151.91 151.66 139.59 132.89 130.47 128.72 127.07 125.84 125.09 124.63 122.86 120.16
79.74 77.00 75.12
47.58 47.05 46.49 41.15
S20a
¹³C NMR (101 MHz, CDCl₃)
200 190 180 170 160 150 140 130 120 110 100 90 80 70 60 50 40 30 20 10
ppm

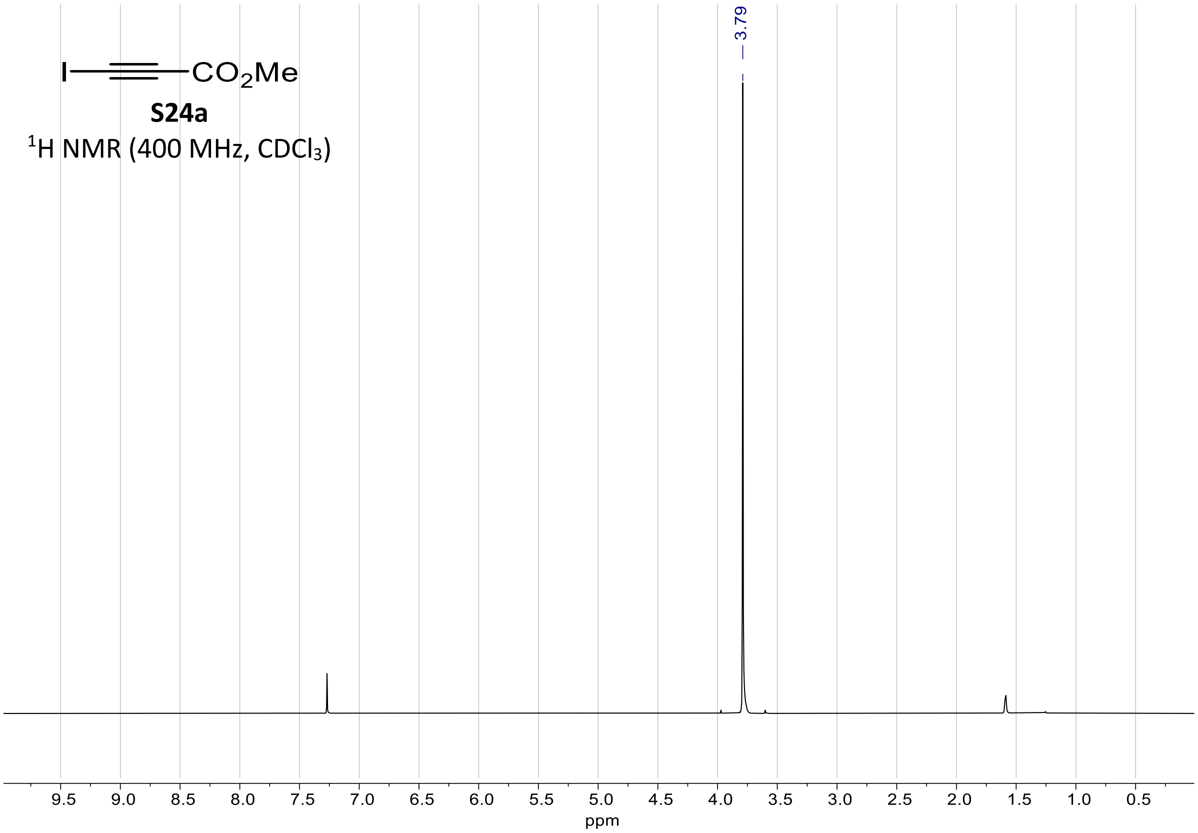
I—≡—CO2Me
S24a
1H NMR (400 MHz, CDCl3)
3.79
9.5 9.0 8.5 8.0 7.5 7.0 6.5 6.0 5.5 5.0 4.5 4.0 3.5 3.0 2.5 2.0 1.5 1.0 0.5
ppm

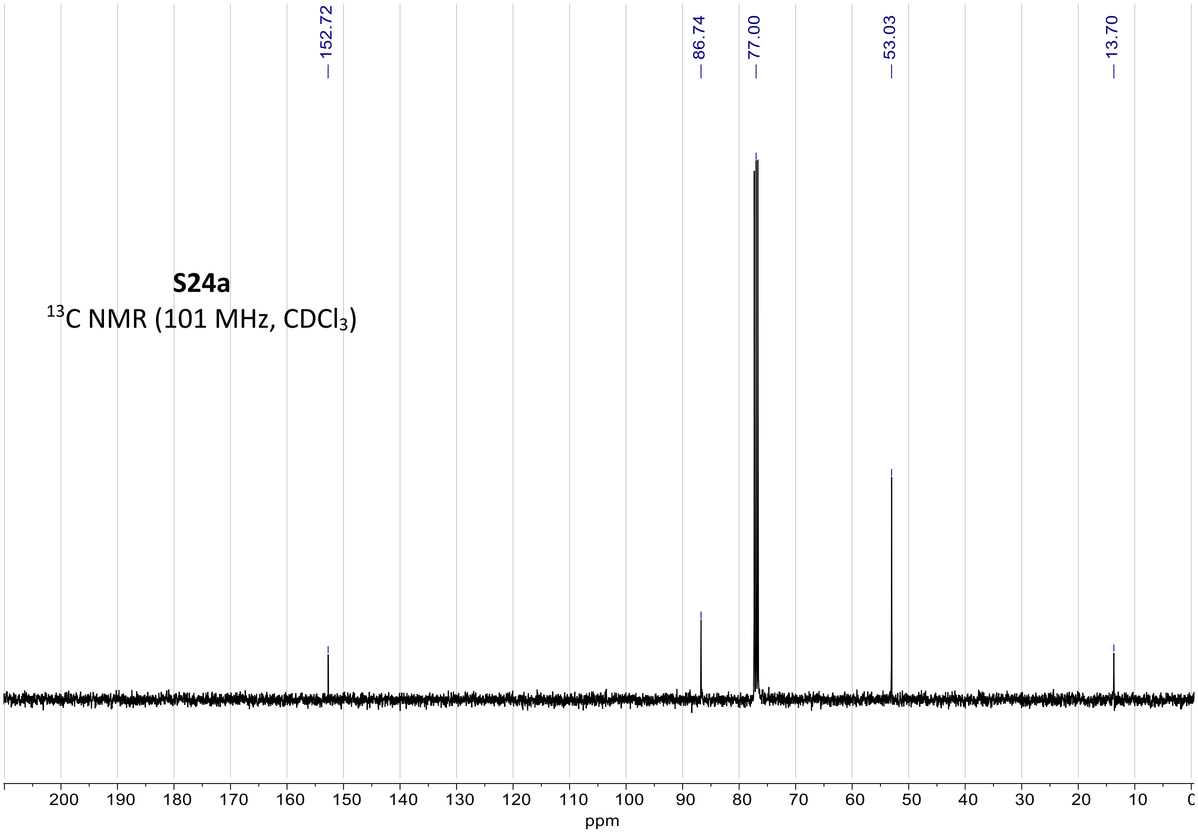
S24a
13C NMR (101 MHz, CDCl3)
152.72
86.74
77.00
53.03
13.70
200 190 180 170 160 150 140 130 120 110 100 90 80 70 60 50 40 30 20 10 0
ppm

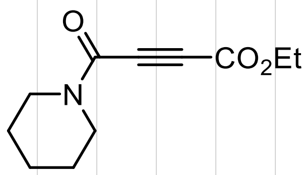

S25a

^{1}H NMR (400 MHz, $CDCl_3$)

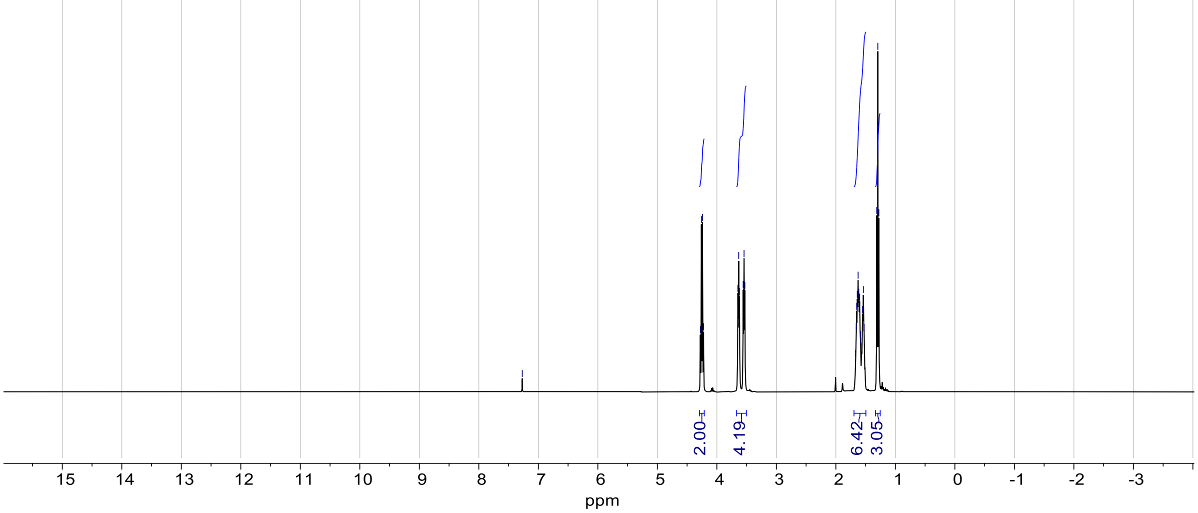

S25a

^{13}C NMR (101 MHz, $CDCl_3$)

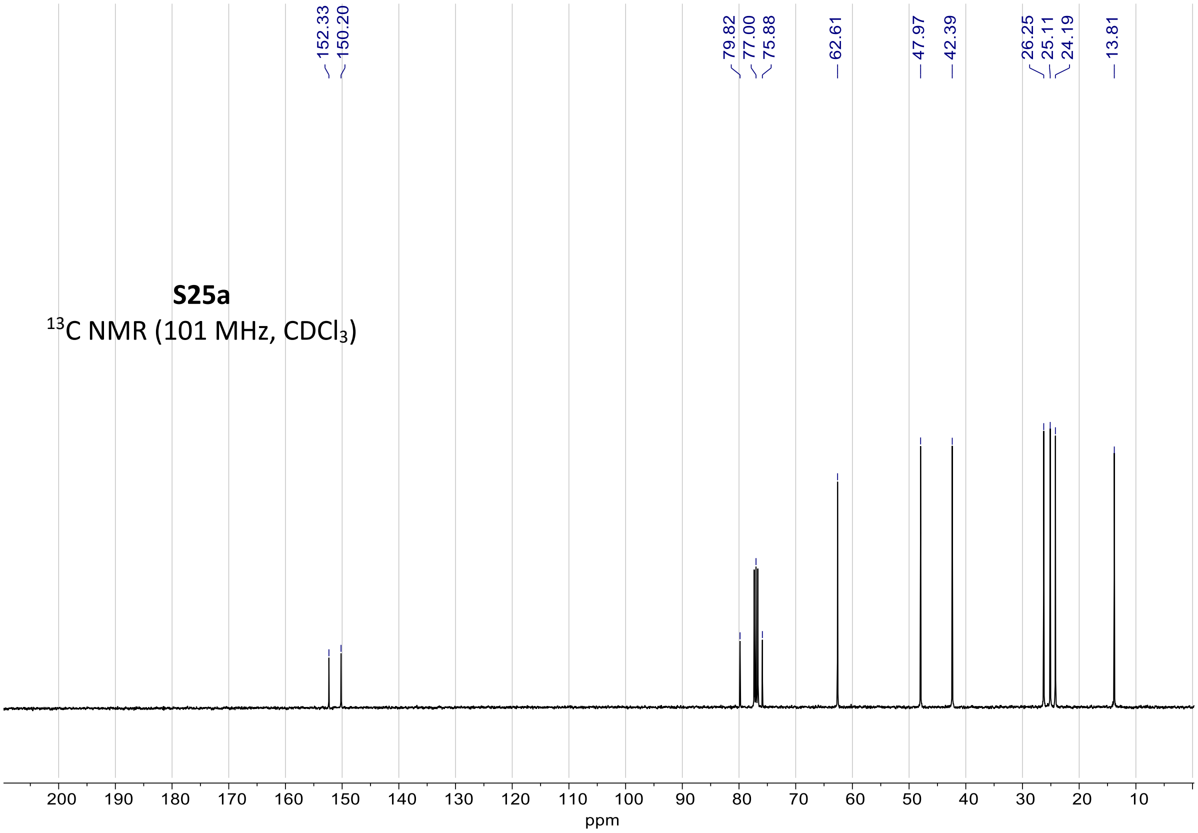

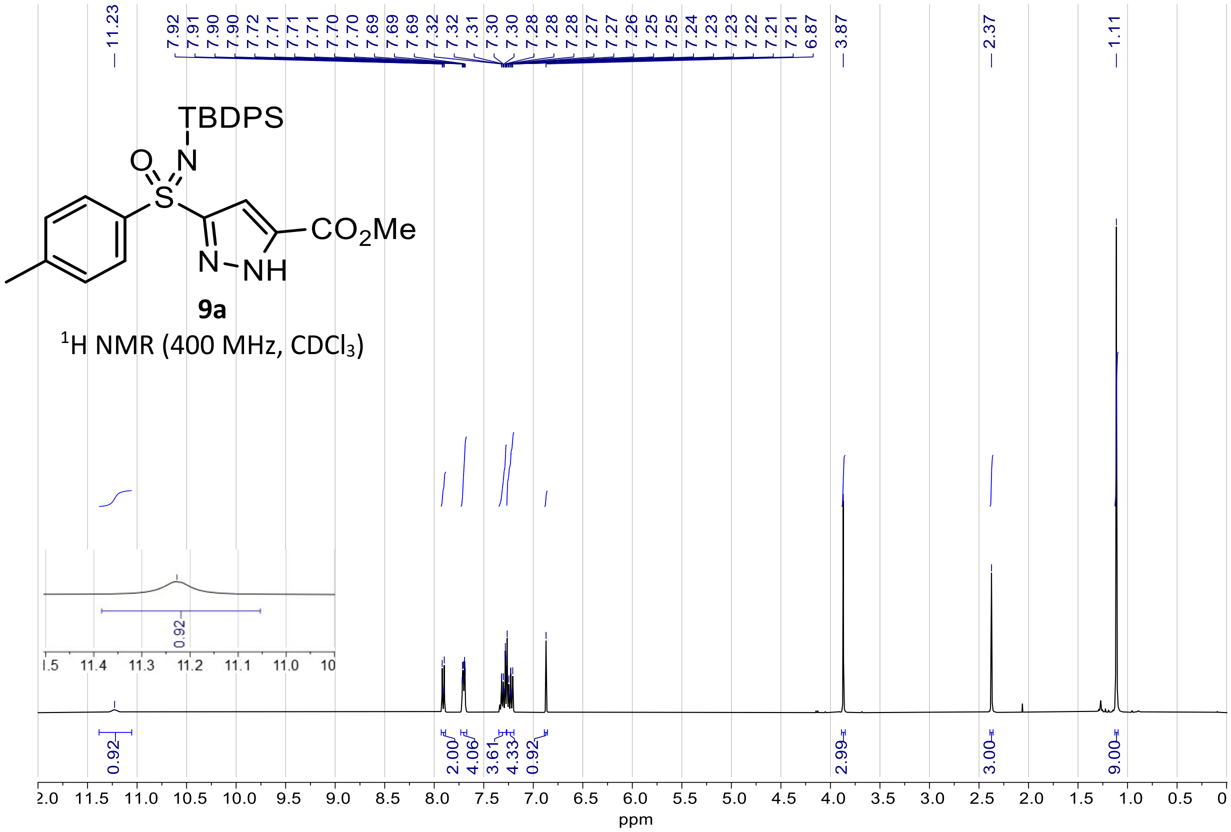
TBDPS
N
O
S
CO2Me
N
NH
9a
1H NMR (400 MHz, CDCl3)
ppm

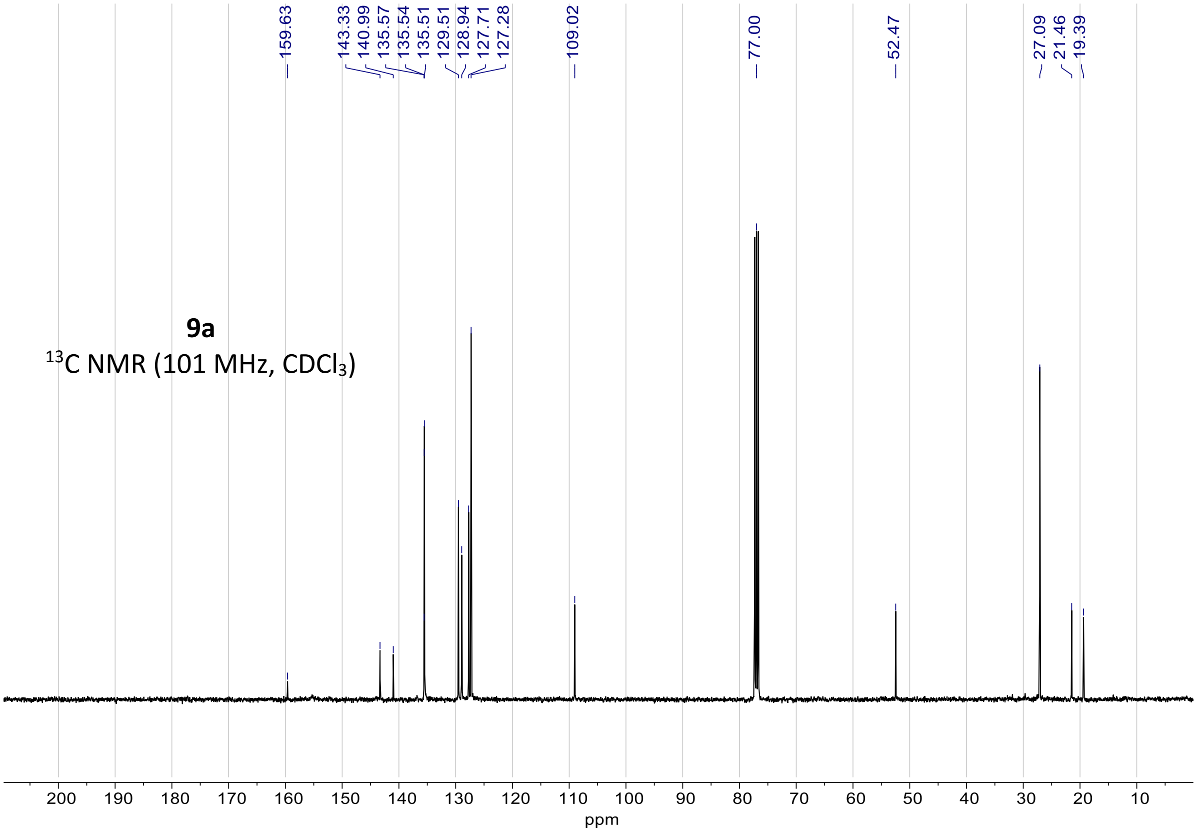
9a
13C NMR (101 MHz, CDCl3)
ppm

9a
HSQC ($CDCl_3$)

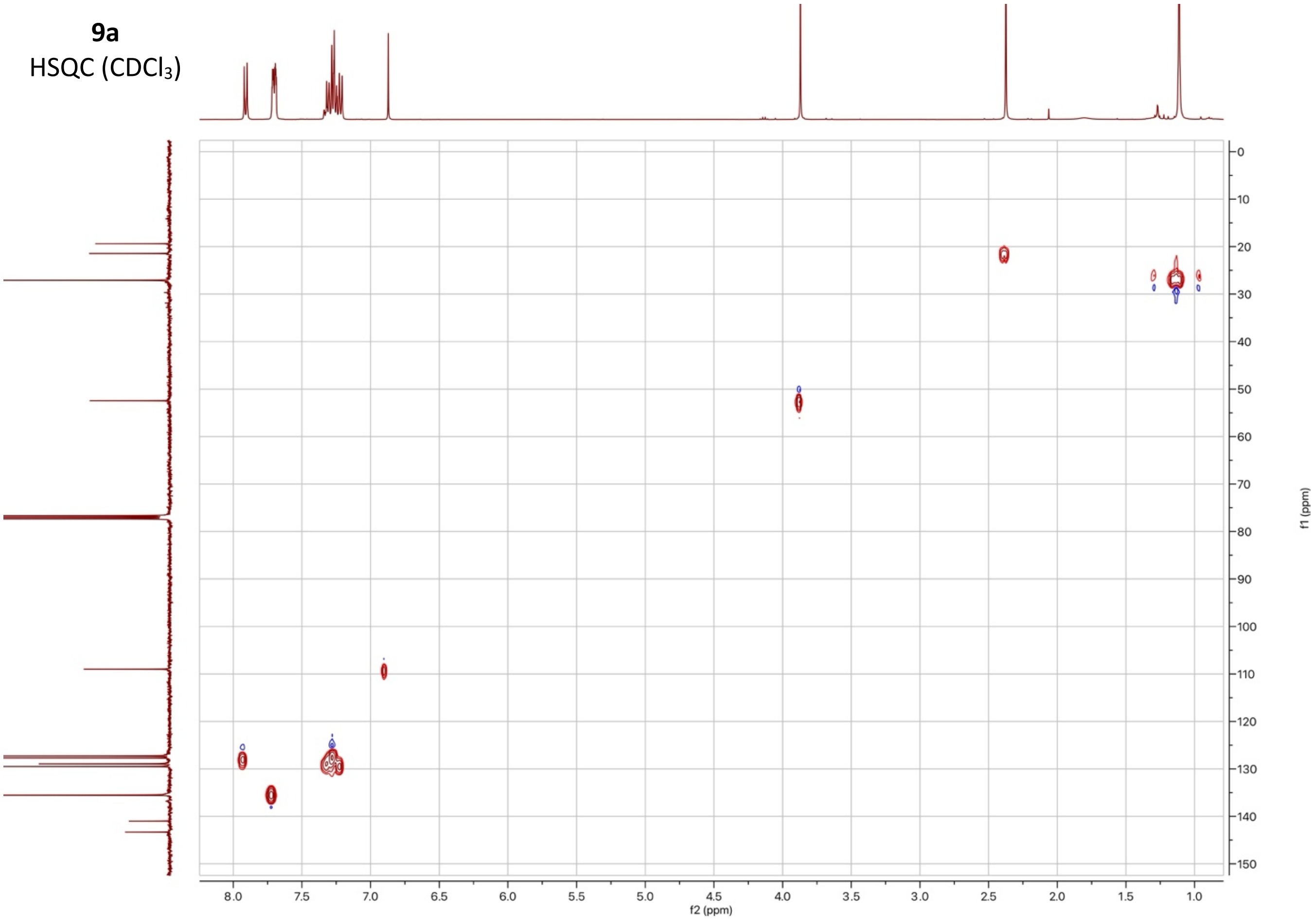

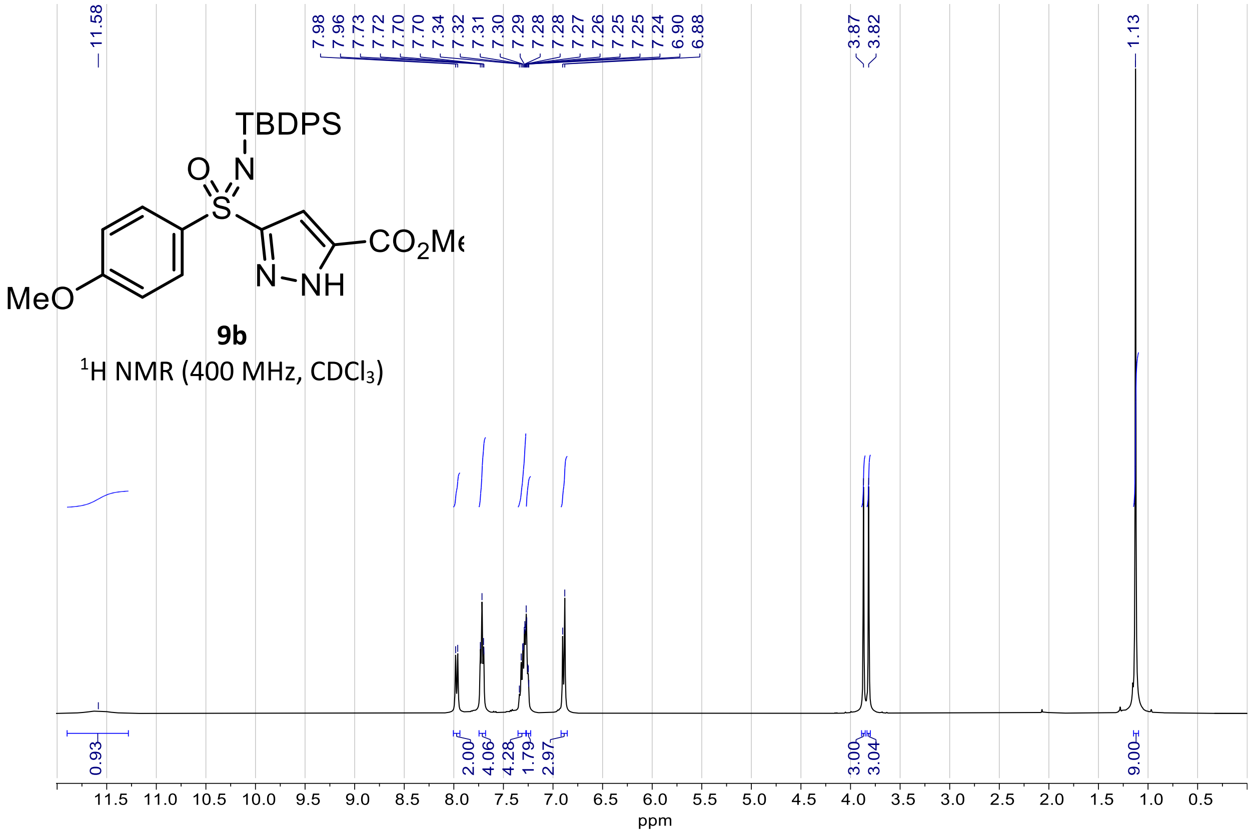

9b

^{1}H NMR (400 MHz, $CDCl_3$)

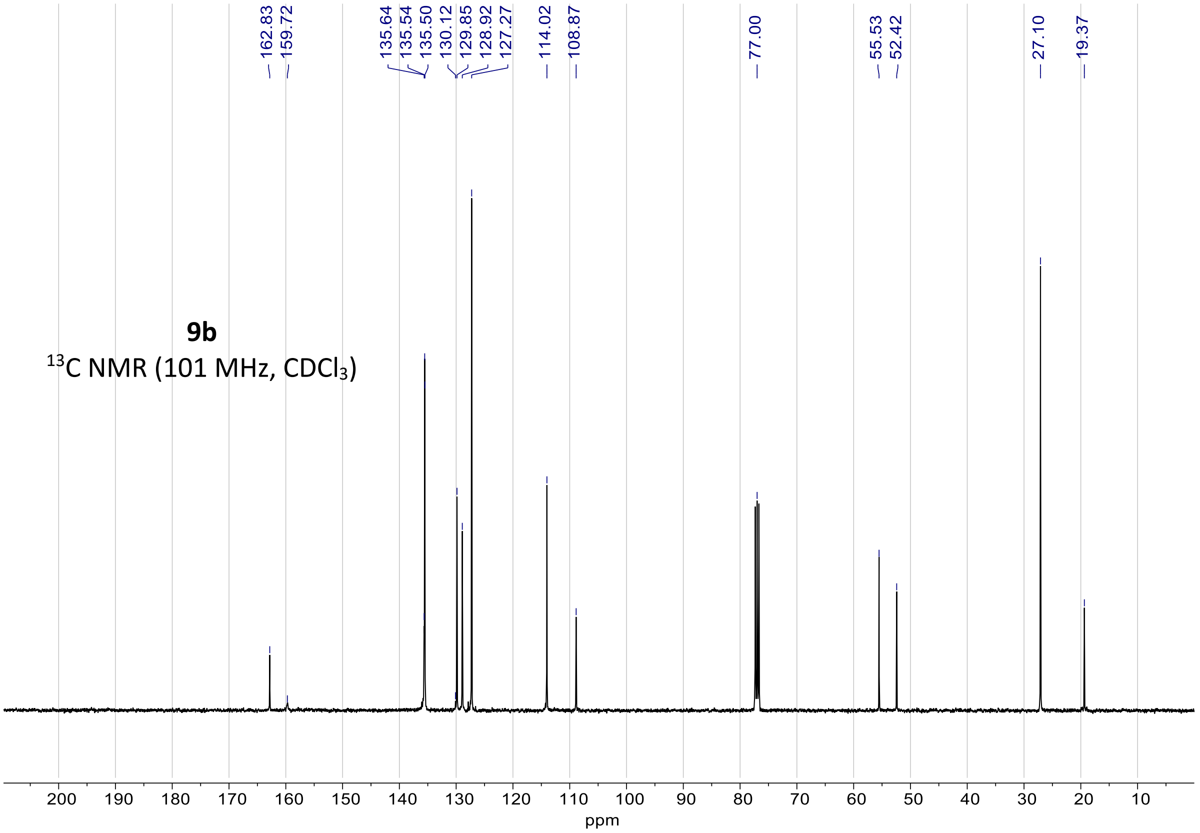

9b

^{13}C NMR (101 MHz, $CDCl_3$)

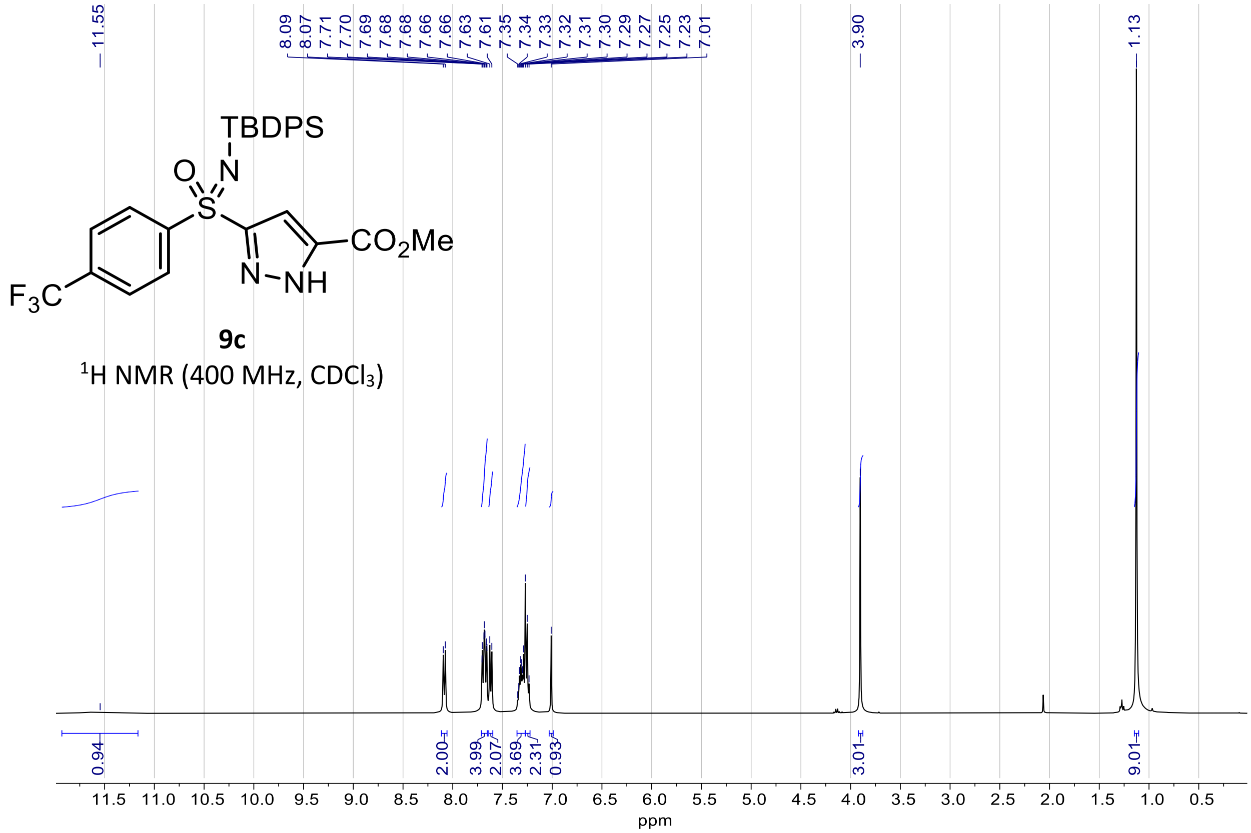
11.55
8.09
8.07
7.71
7.70
7.69
7.68
7.68
7.66
7.66
7.63
7.61
7.35
7.34
7.33
7.32
7.31
7.30
7.29
7.27
7.25
7.23
7.01
3.90
1.13
TBDPS
O
N
S
CO2Me
N
NH
F3C
9c
1H NMR (400 MHz, CDCl3)
0.94
2.00
3.99
2.07
3.69
2.31
0.93
3.01
9.01
ppm

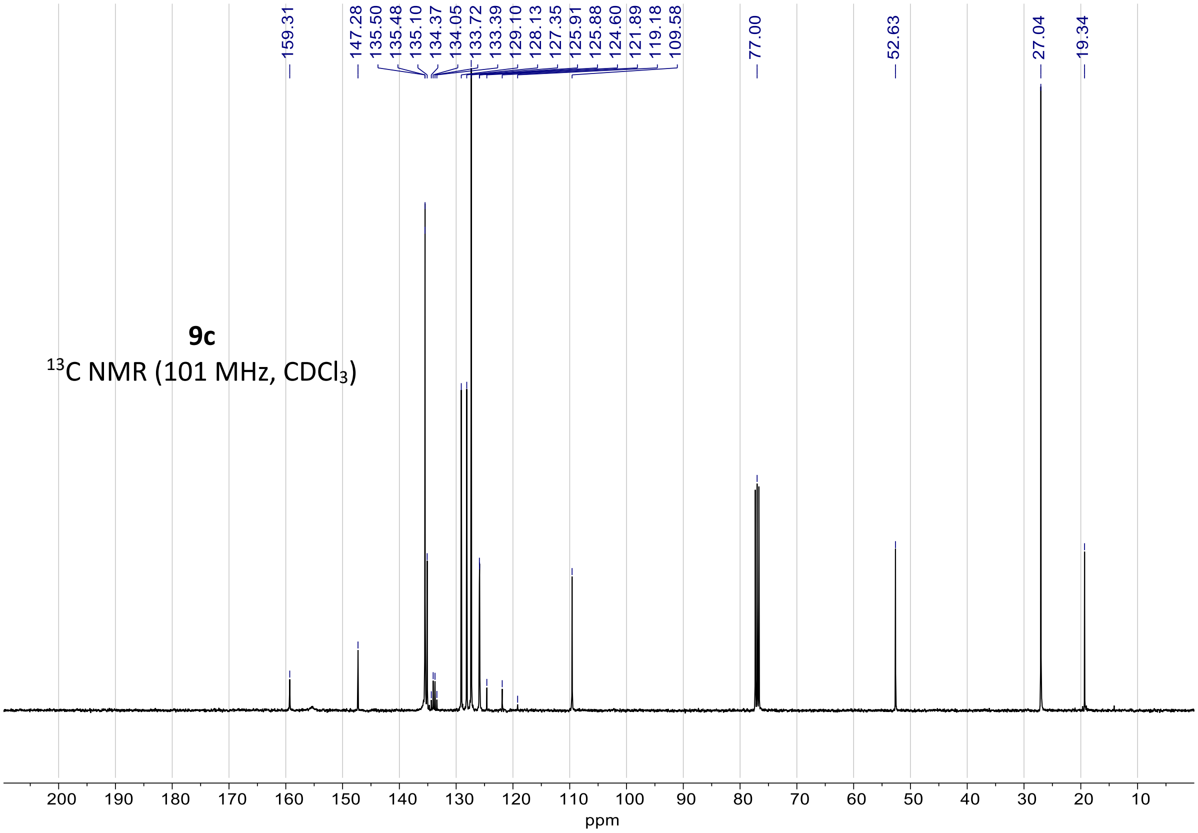
159.31
147.28
135.50
135.48
135.10
134.37
134.05
133.72
133.39
129.10
128.13
127.35
125.91
125.88
124.60
121.89
119.18
109.58
77.00
52.63
27.04
19.34
9c
13C NMR (101 MHz, CDCl3)
ppm

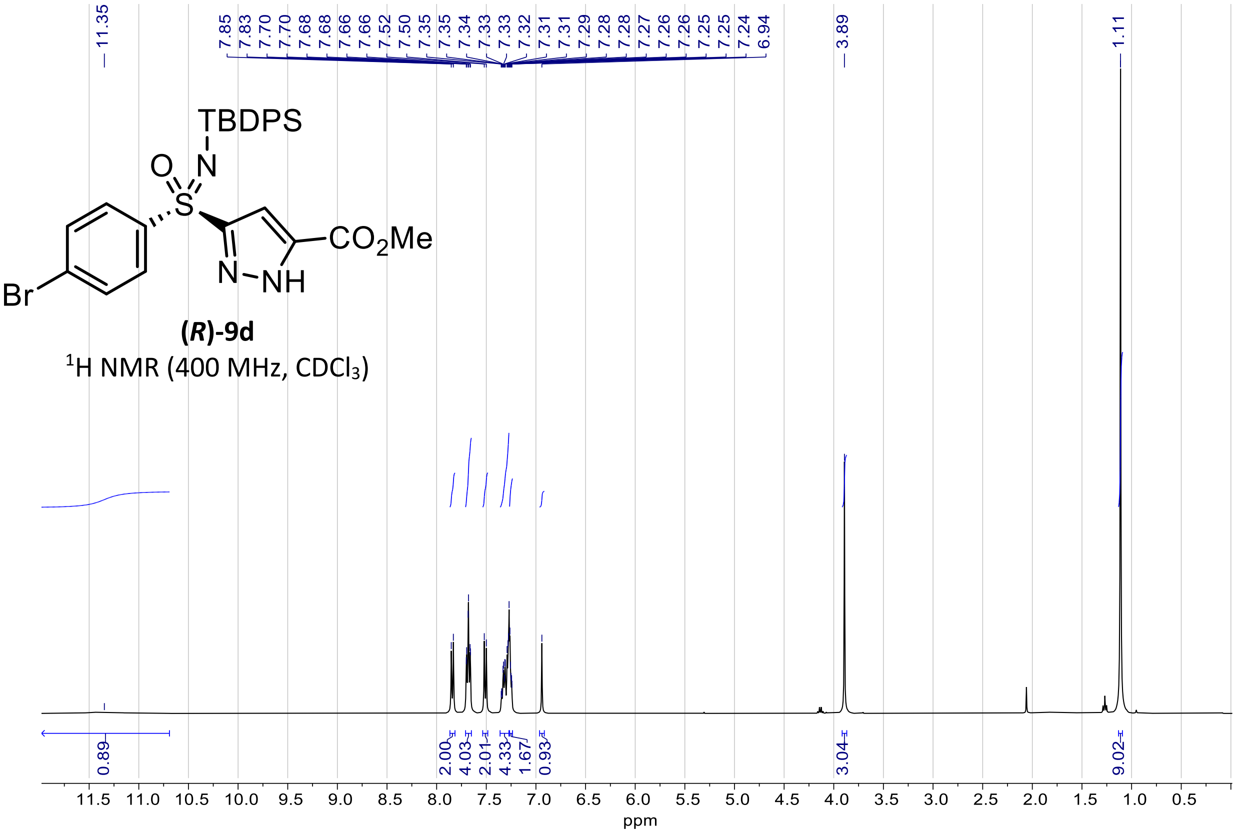

(*R*)-9d
^{1}H NMR (400 MHz, CDCl$_3$)

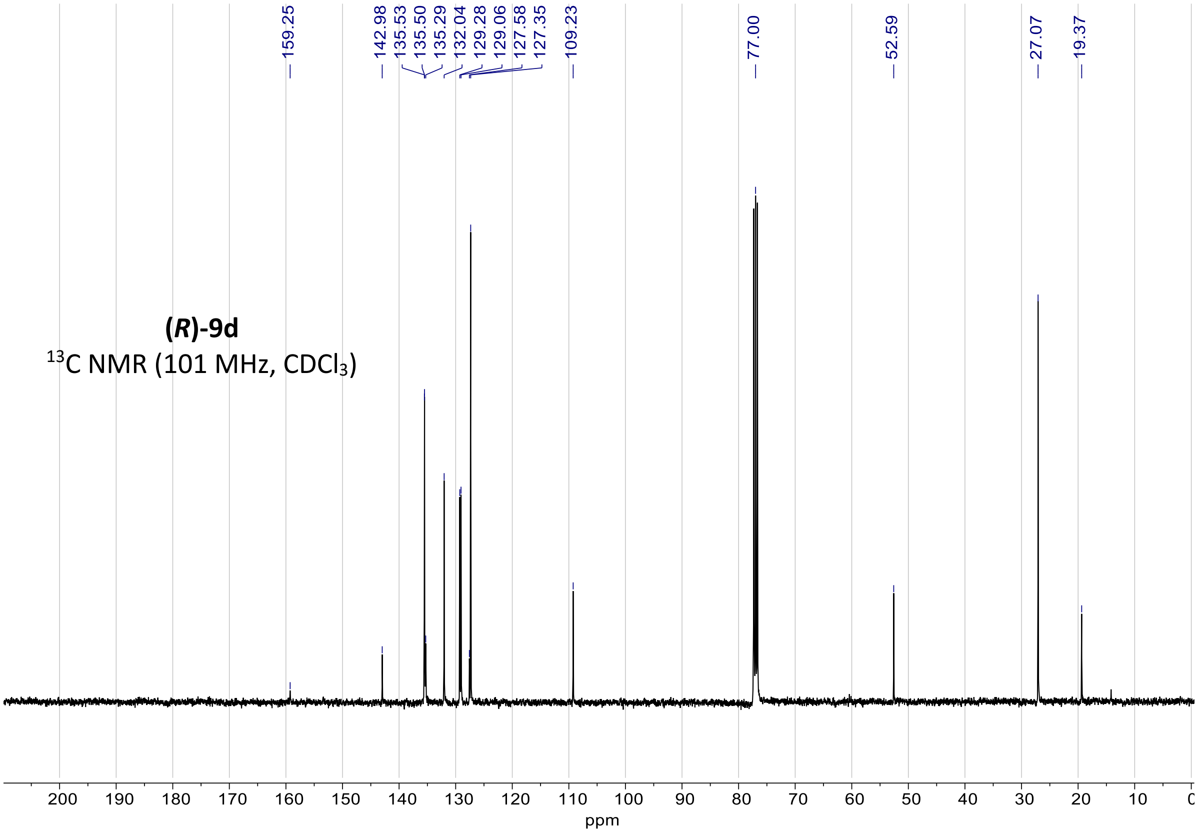

(*R*)-9d
^{13}C NMR (101 MHz, CDCl$_3$)

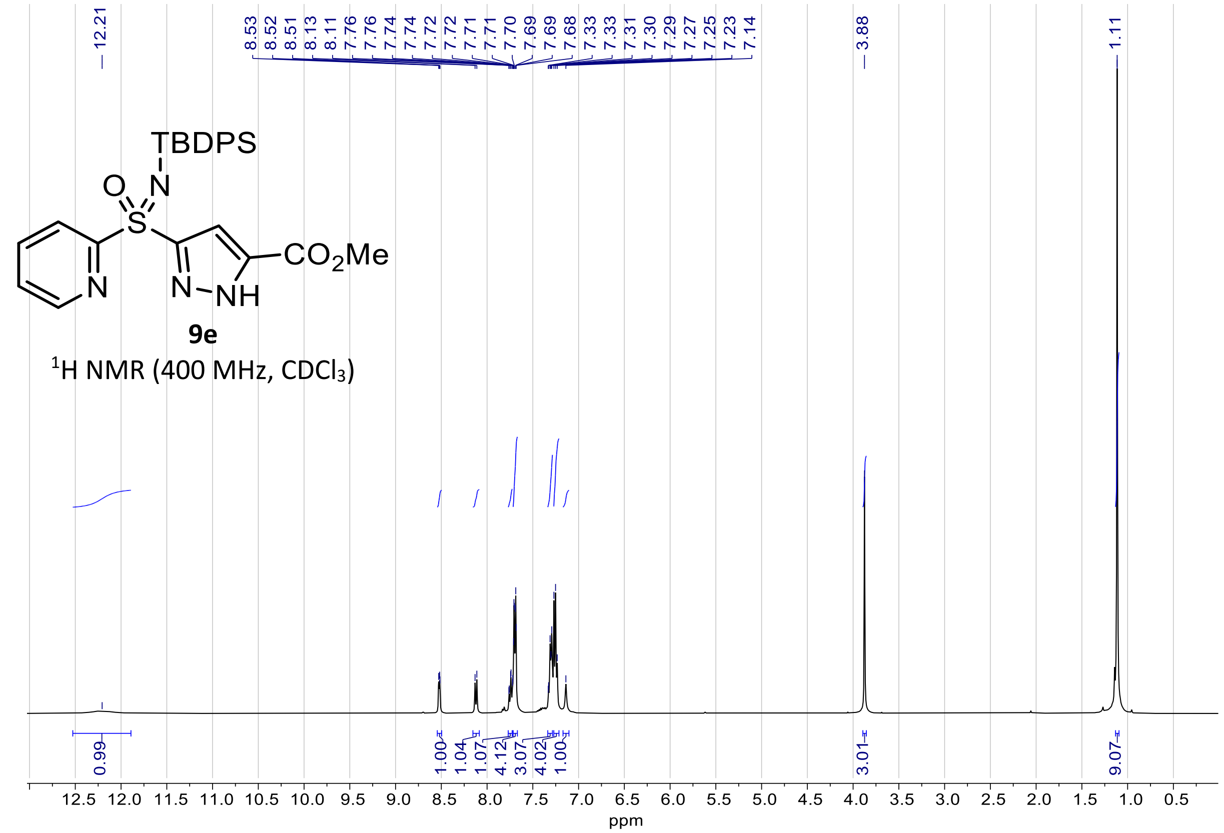
12.21
8.53 8.52 8.51 8.13 8.11 7.76 7.76 7.74 7.74 7.72 7.72 7.71 7.71 7.70 7.69 7.69 7.68 7.33 7.33 7.31 7.30 7.29 7.27 7.25 7.23 7.14
3.88
1.11
TBDPS
O
N
S
CO2Me
N
N
NH
9e
1H NMR (400 MHz, CDCl3)
0.99
1.00 1.04 1.07 4.12 3.07 4.02 1.00
3.01
9.07
12.5 12.0 11.5 11.0 10.5 10.0 9.5 9.0 8.5 8.0 7.5 7.0 6.5 6.0 5.5 5.0 4.5 4.0 3.5 3.0 2.5 2.0 1.5 1.0 0.5
ppm

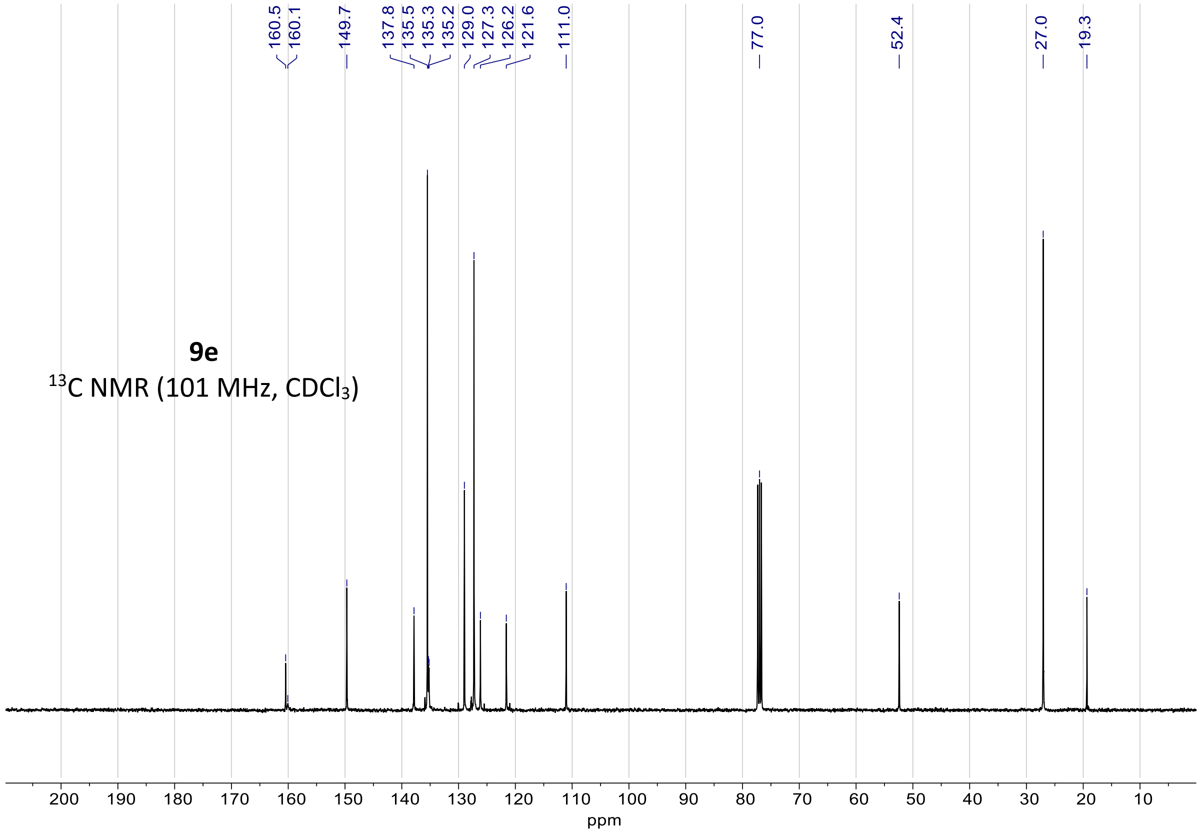
160.5 160.1
149.7
137.8 135.5 135.3 135.2 129.0 127.3 126.2 121.6
111.0
77.0
52.4
27.0
19.3
9e
13C NMR (101 MHz, CDCl3)
200 190 180 170 160 150 140 130 120 110 100 90 80 70 60 50 40 30 20 10
ppm

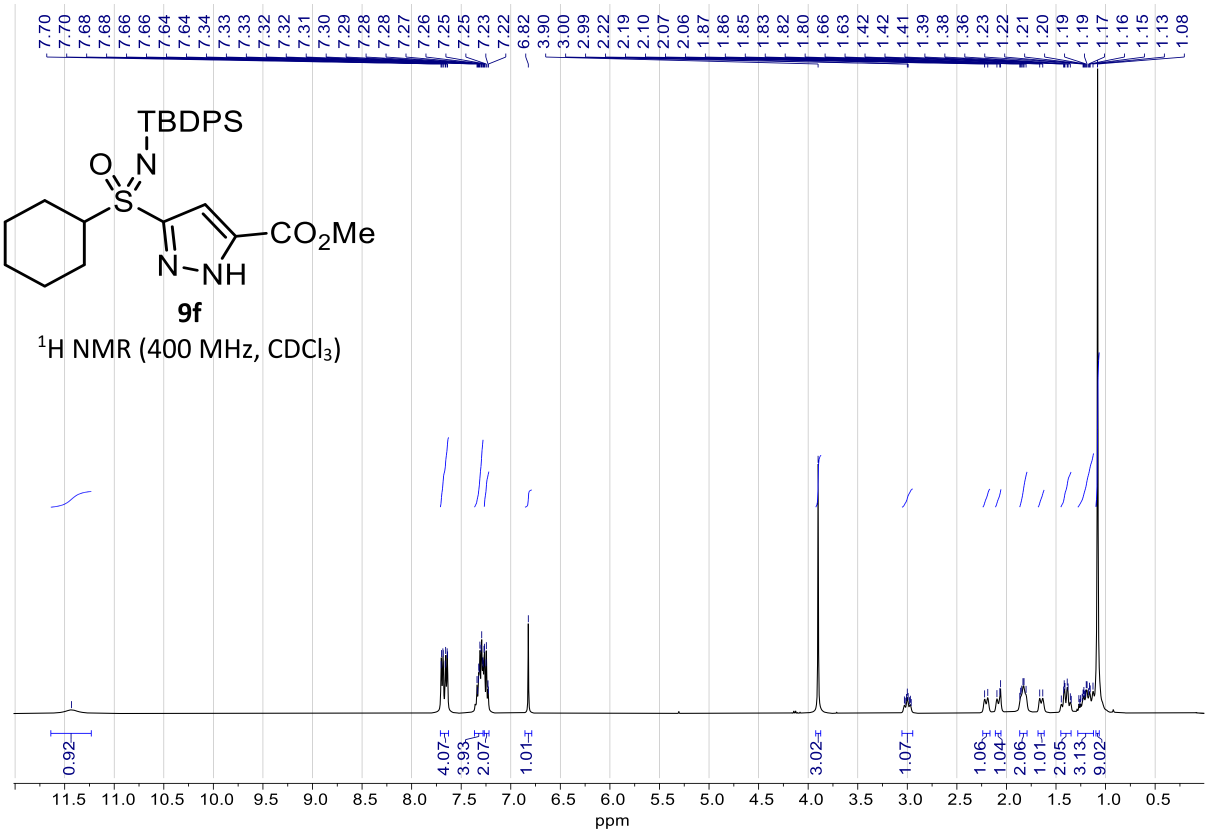
7.70
7.70
7.68
7.66
7.66
7.64
7.64
7.34
7.33
7.33
7.32
7.32
7.31
7.30
7.29
7.28
7.28
7.27
7.26
7.25
7.25
7.23
7.22
6.82
3.90
3.00
2.99
2.22
2.19
2.10
2.07
2.06
1.87
1.86
1.85
1.83
1.82
1.80
1.66
1.63
1.42
1.42
1.41
1.39
1.38
1.36
1.23
1.22
1.21
1.20
1.19
1.19
1.17
1.16
1.15
1.13
1.08
TBDPS
N
O
S
CO2Me
N
NH
9f
1H NMR (400 MHz, CDCl3)
0.92
4.07
3.93
2.07
1.01
3.02
1.07
1.06
1.04
2.06
1.01
2.05
3.13
9.02
ppm

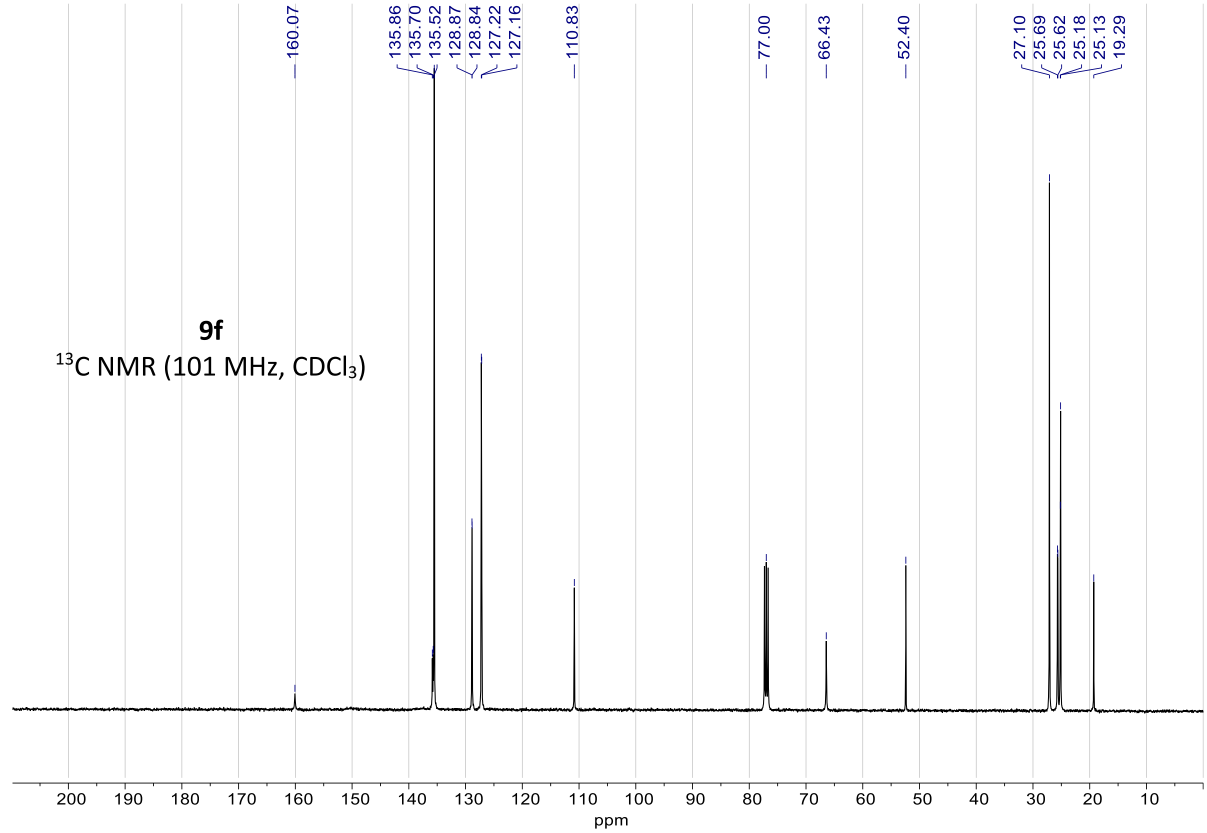
160.07
135.86
135.70
135.52
128.87
128.84
127.22
127.16
110.83
77.00
66.43
52.40
27.10
25.69
25.62
25.18
25.13
19.29
9f
13C NMR (101 MHz, CDCl3)
ppm

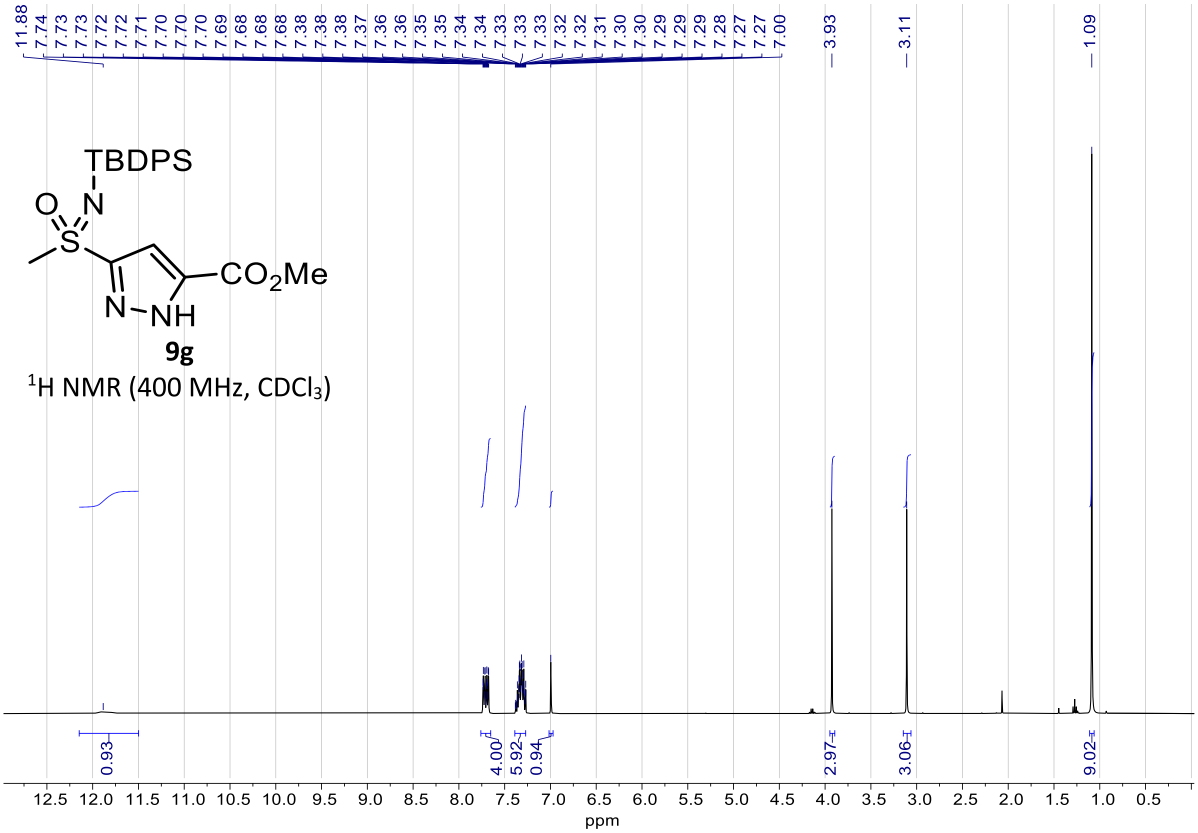
11.88 7.74 7.73 7.73 7.72 7.72 7.71 7.70 7.70 7.69 7.68 7.68 7.38 7.38 7.38 7.37 7.36 7.36 7.35 7.35 7.34 7.34 7.33 7.33 7.33 7.32 7.32 7.31 7.30 7.30 7.29 7.29 7.28 7.27 7.27 7.00
3.93
3.11
1.09
TBDPS
O
N
S
CO2Me
N
NH
9g
1H NMR (400 MHz, CDCl3)
0.93
4.00
5.92
0.94
2.97
3.06
9.02
12.5 12.0 11.5 11.0 10.5 10.0 9.5 9.0 8.5 8.0 7.5 7.0 6.5 6.0 5.5 5.0 4.5 4.0 3.5 3.0 2.5 2.0 1.5 1.0 0.5
ppm

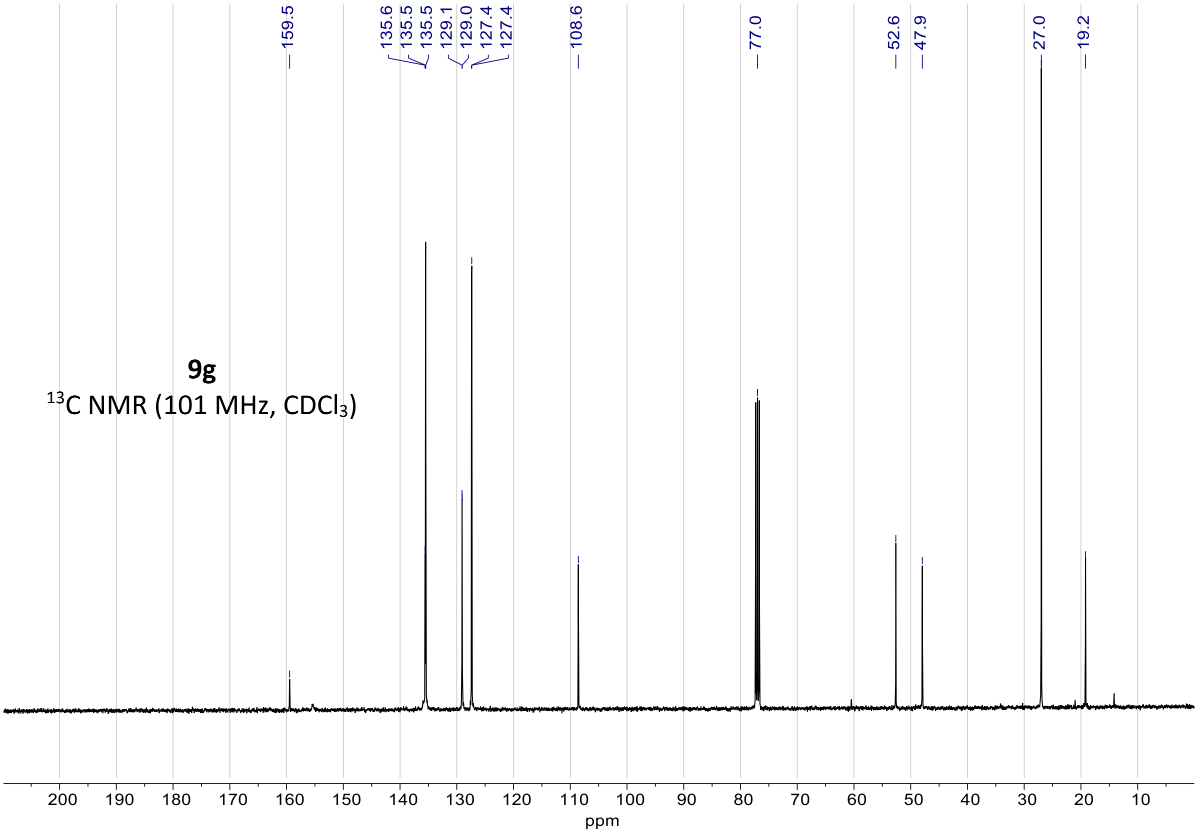
159.5
135.6 135.5 135.5 129.1 129.0 127.4 127.4
108.6
77.0
52.6
47.9
27.0
19.2
9g
13C NMR (101 MHz, CDCl3)
200 190 180 170 160 150 140 130 120 110 100 90 80 70 60 50 40 30 20 10
ppm

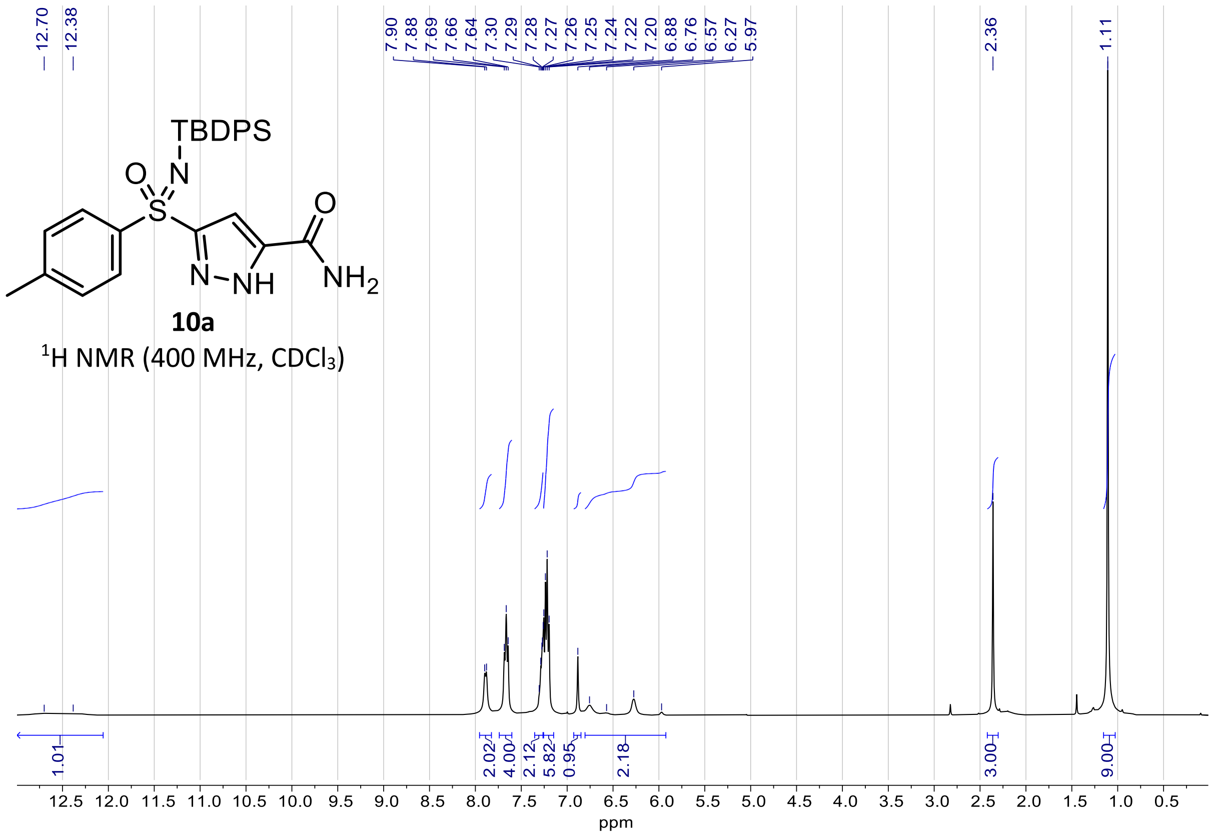

10a

^{1}H NMR (400 MHz, $CDCl_3$)

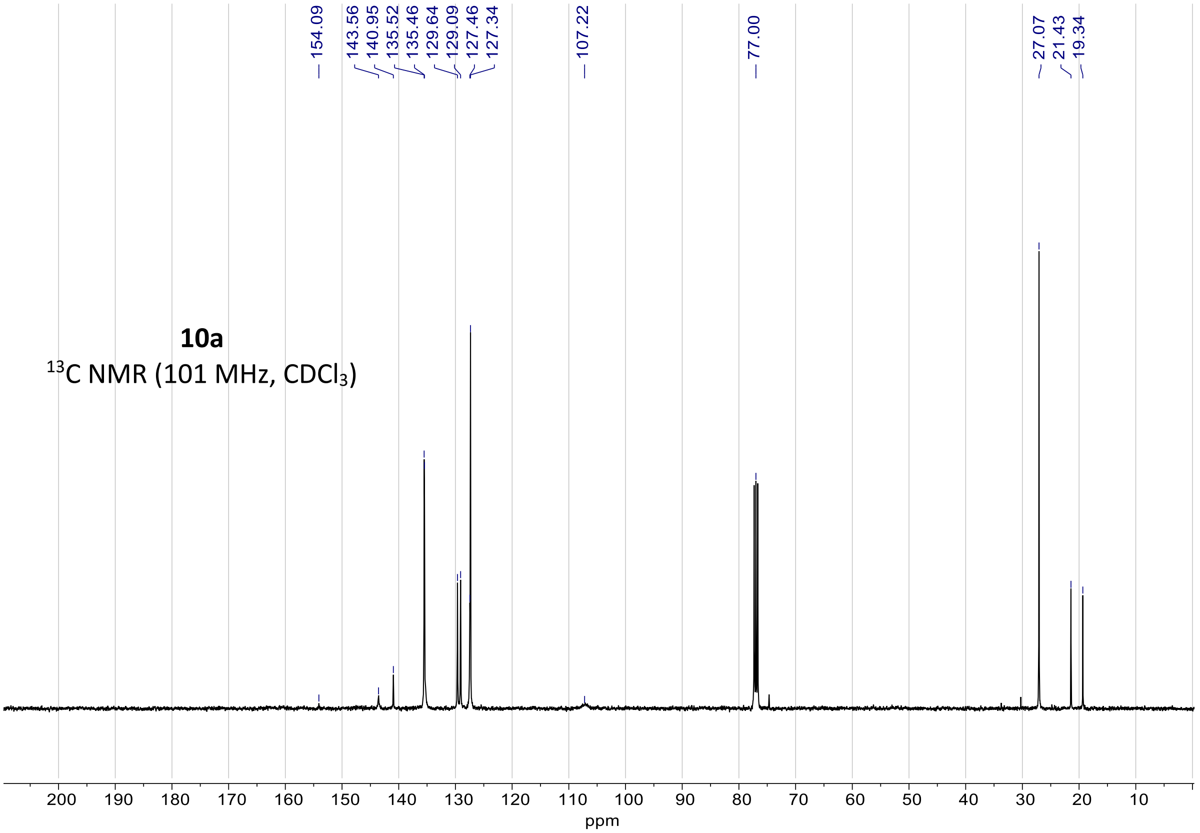

10a

^{13}C NMR (101 MHz, $CDCl_3$)

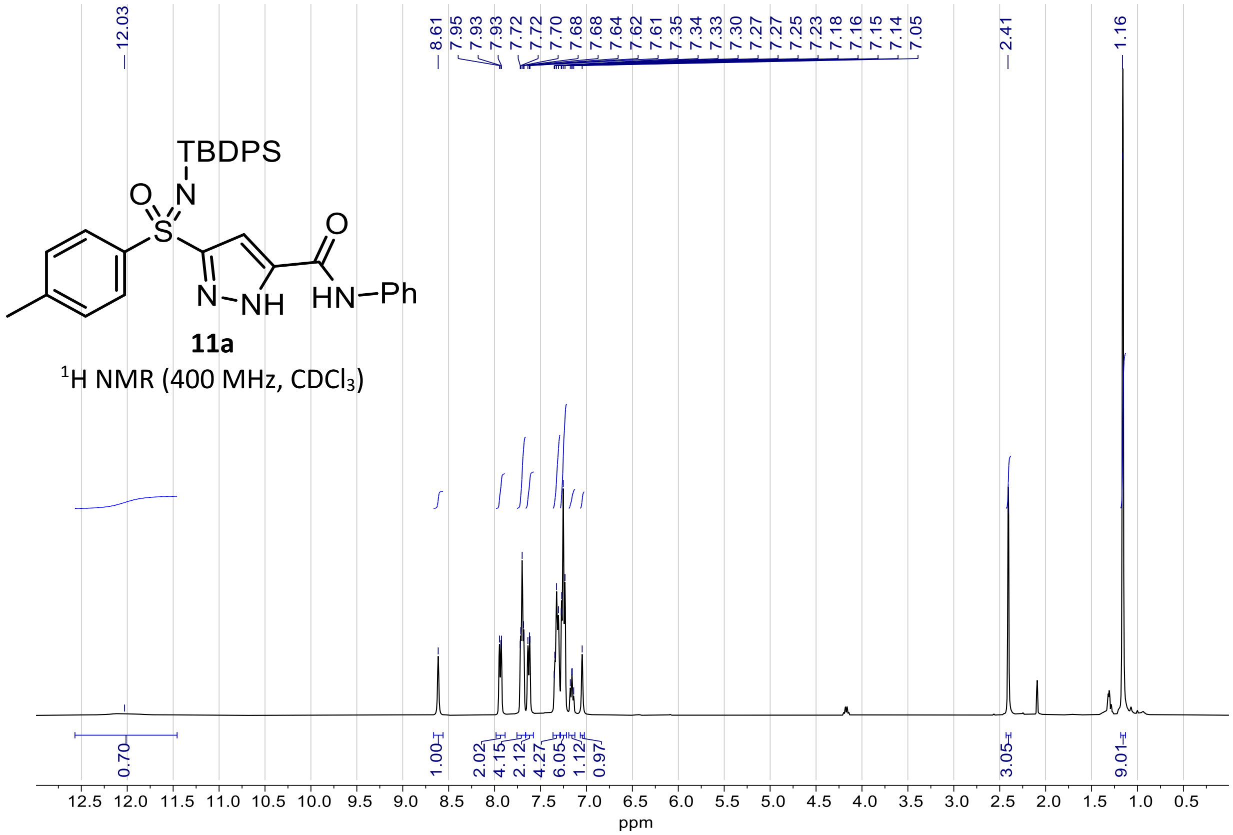
12.03
8.61 7.95 7.93 7.93 7.72 7.72 7.70 7.68 7.68 7.64 7.62 7.61 7.35 7.34 7.33 7.30 7.27 7.27 7.25 7.23 7.18 7.16 7.15 7.14 7.05
2.41
1.16
TBDPS
O
N
S
O
N
NH
HN–Ph
11a
1H NMR (400 MHz, CDCl3)
0.70
1.00
2.02
4.15
2.12
4.27
6.05
1.12
0.97
3.05
9.01
12.5 12.0 11.5 11.0 10.5 10.0 9.5 9.0 8.5 8.0 7.5 7.0 6.5 6.0 5.5 5.0 4.5 4.0 3.5 3.0 2.5 2.0 1.5 1.0 0.5
ppm

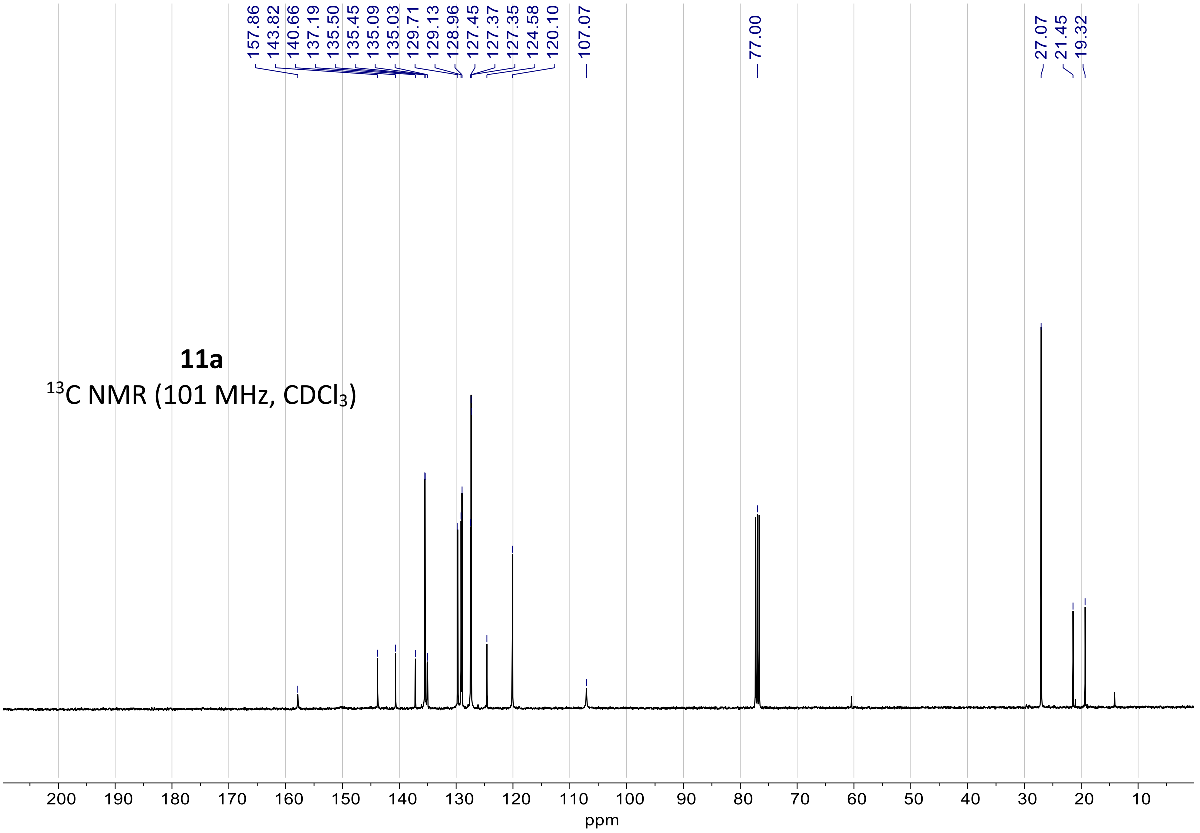
157.86 143.82 140.66 137.19 135.50 135.45 135.09 135.03 129.71 129.13 128.96 127.45 127.37 127.35 124.58 120.10
107.07
77.00
27.07 21.45 19.32
11a
13C NMR (101 MHz, CDCl3)
200 190 180 170 160 150 140 130 120 110 100 90 80 70 60 50 40 30 20 10
ppm

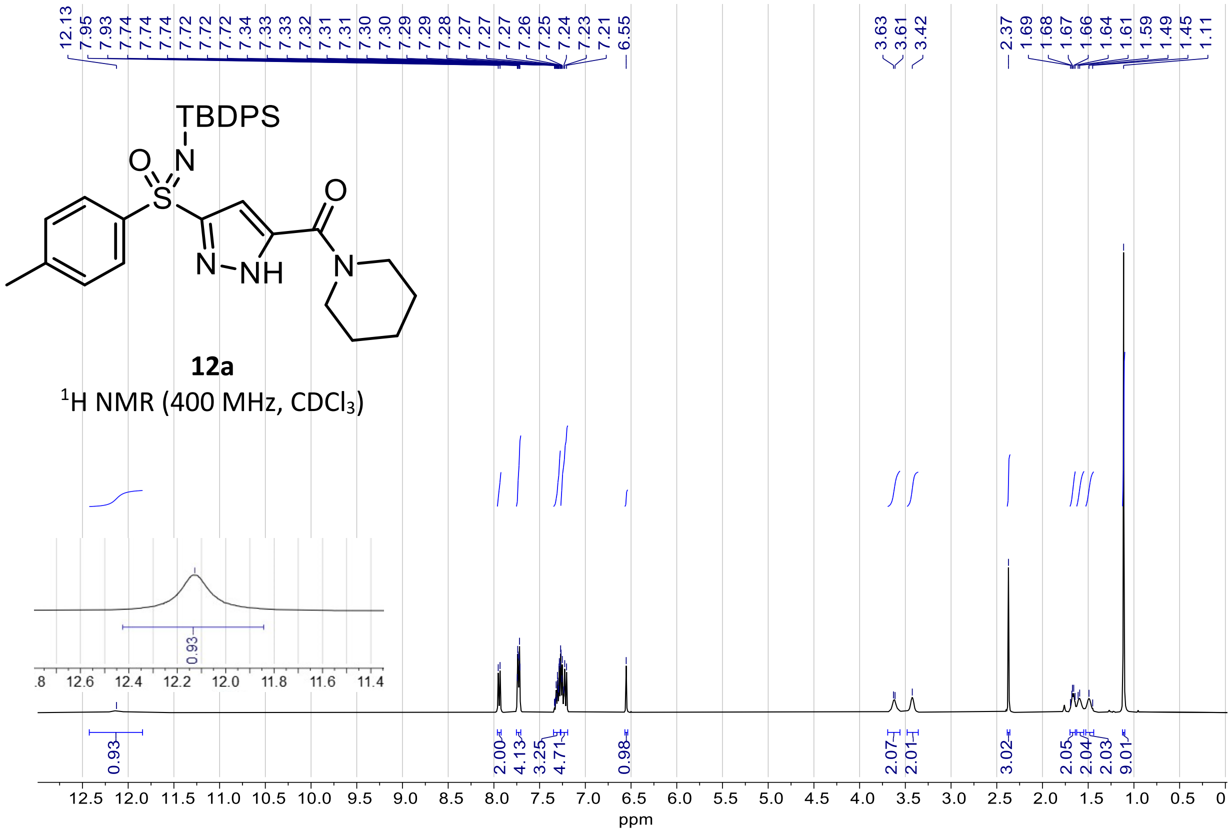
12.13
7.95
7.93
7.74
7.74
7.74
7.72
7.72
7.72
7.34
7.33
7.33
7.32
7.31
7.31
7.30
7.30
7.29
7.29
7.28
7.27
7.27
7.27
7.26
7.25
7.24
7.23
7.21
6.55
3.63
3.61
3.42
2.37
1.69
1.68
1.67
1.66
1.64
1.61
1.59
1.49
1.45
1.11
TBDPS
O
N
S
O
N
NH
N
O
12a
^{1}H NMR (400 MHz, CDCl3)
0.93
2.00
4.13
3.25
4.71
0.98
2.07
2.01
3.02
2.05
2.04
2.03
9.01
ppm

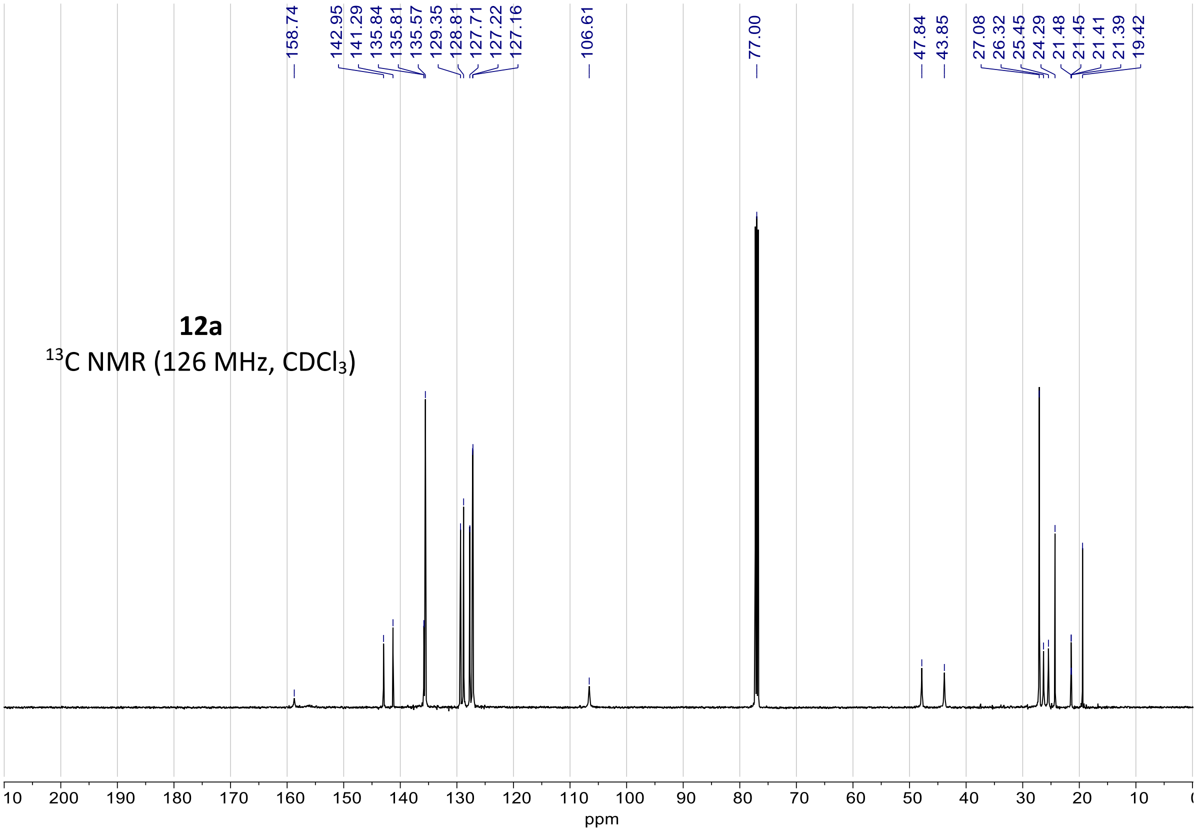
158.74
142.95
141.29
135.84
135.81
135.57
129.35
128.81
127.71
127.22
127.16
106.61
77.00
47.84
43.85
27.08
26.32
25.45
24.29
21.48
21.45
21.41
21.39
19.42
12a
^{13}C NMR (126 MHz, CDCl3)
ppm

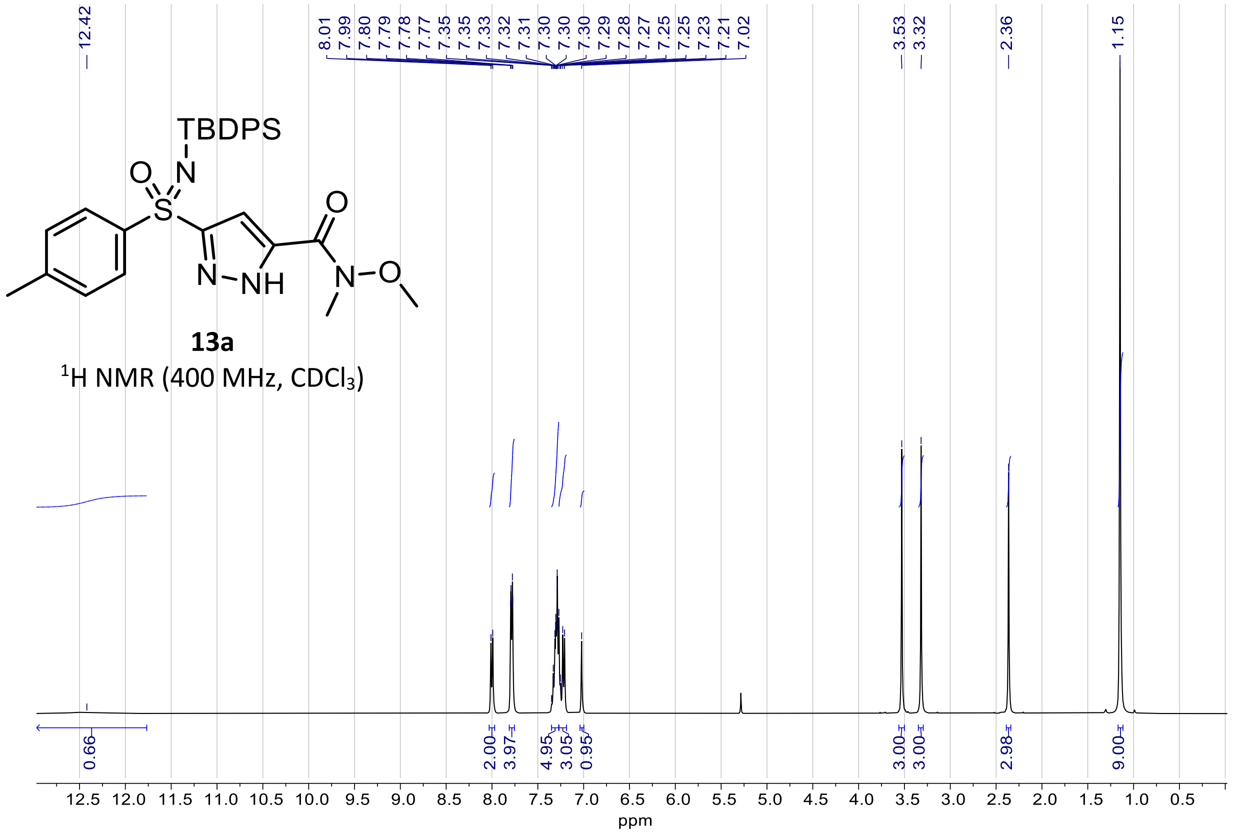
TBDPS
13a
1H NMR (400 MHz, CDCl3)
12.42
8.01 7.99 7.80 7.79 7.78 7.77 7.35 7.35 7.33 7.32 7.31 7.30 7.30 7.30 7.29 7.28 7.27 7.25 7.25 7.23 7.21 7.02
3.53 3.32 2.36 1.15
0.66 2.00 3.97 4.95 3.05 0.95 3.00 3.00 2.98 9.00
ppm

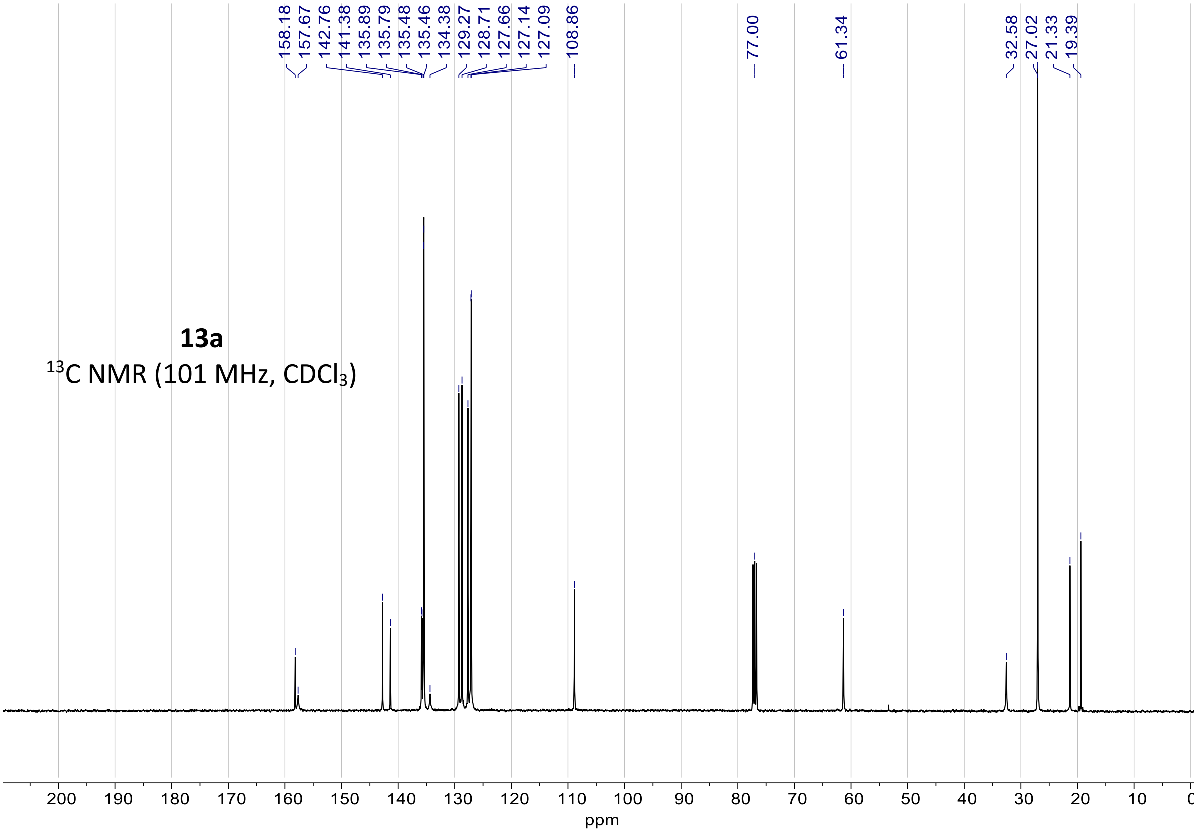
158.18 157.67 142.76 141.38 135.89 135.79 135.48 135.46 134.38 129.27 128.71 127.66 127.14 127.09 108.86
77.00 61.34 32.58 27.02 21.33 19.39
13a
13C NMR (101 MHz, CDCl3)
ppm

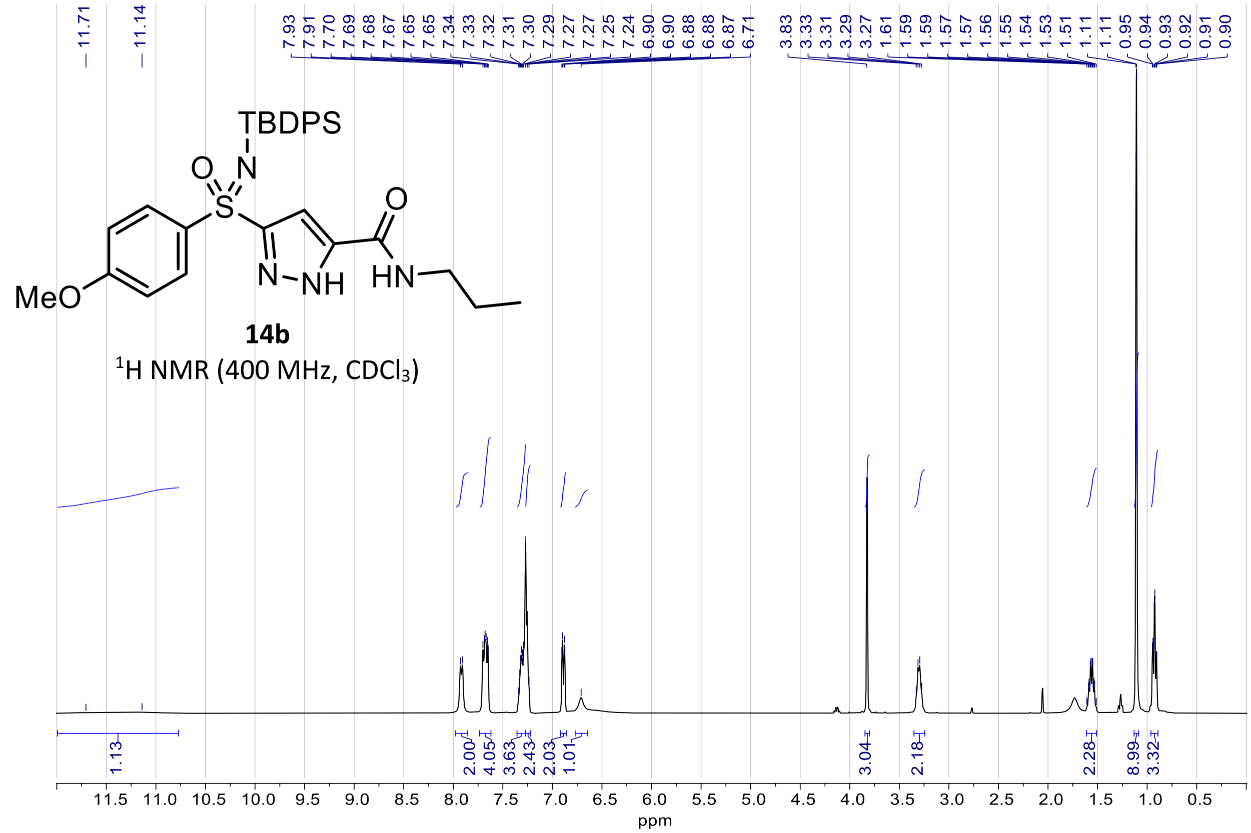
TBDPS
MeO
N
O
S
N
NH
O
HN
14b
^{1}H NMR (400 MHz, CDCl$_3$)
ppm

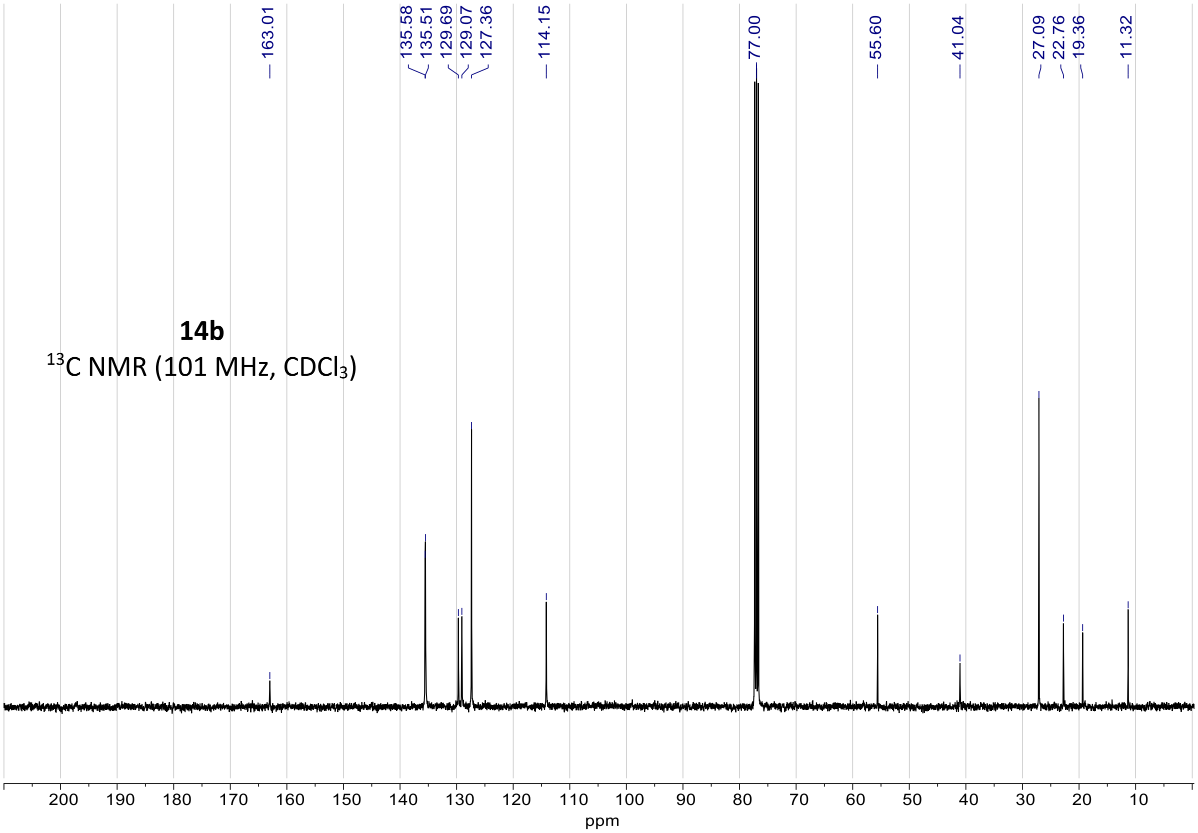
14b
^{13}C NMR (101 MHz, CDCl$_3$)
ppm

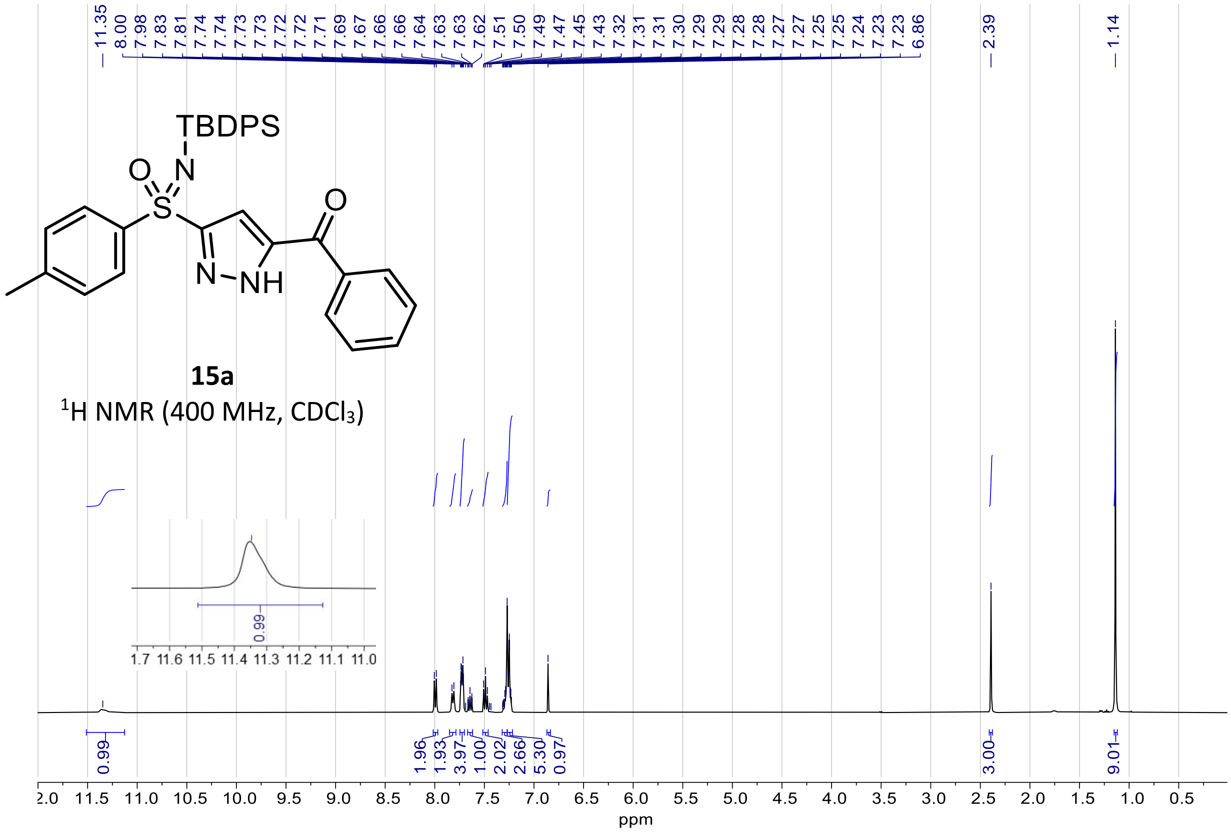

15a

^{1}H NMR (400 MHz, $CDCl_3$)

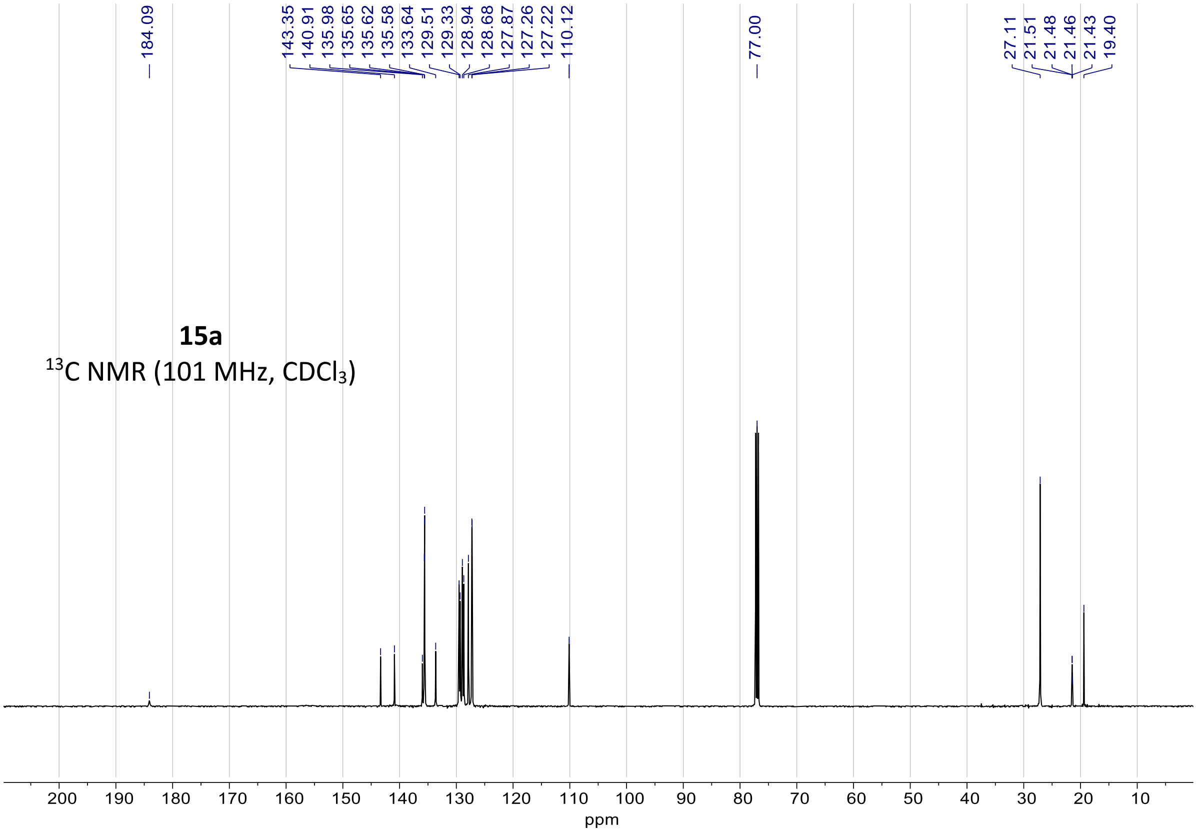

15a

^{13}C NMR (101 MHz, $CDCl_3$)

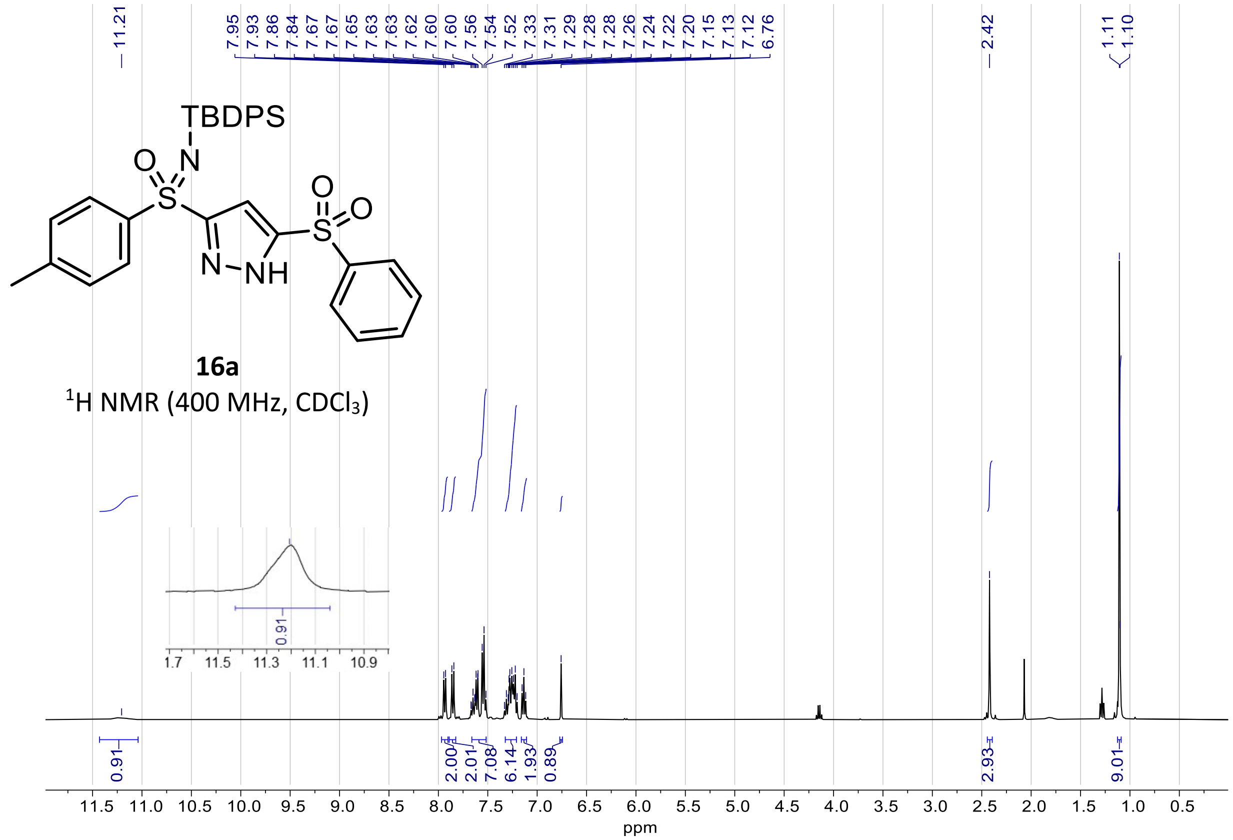

TBDPS
16a
^{1}H NMR (400 MHz, CDCl$_3$)
11.21
7.95 7.93 7.86 7.84 7.67 7.67 7.65 7.63 7.63 7.62 7.60 7.60 7.56 7.54 7.52 7.33 7.31 7.29 7.28 7.28 7.26 7.24 7.22 7.20 7.15 7.13 7.12 6.76
2.42
1.11 1.10
0.91 2.00 2.01 7.08 6.14 1.93 0.89 2.93 9.01
ppm

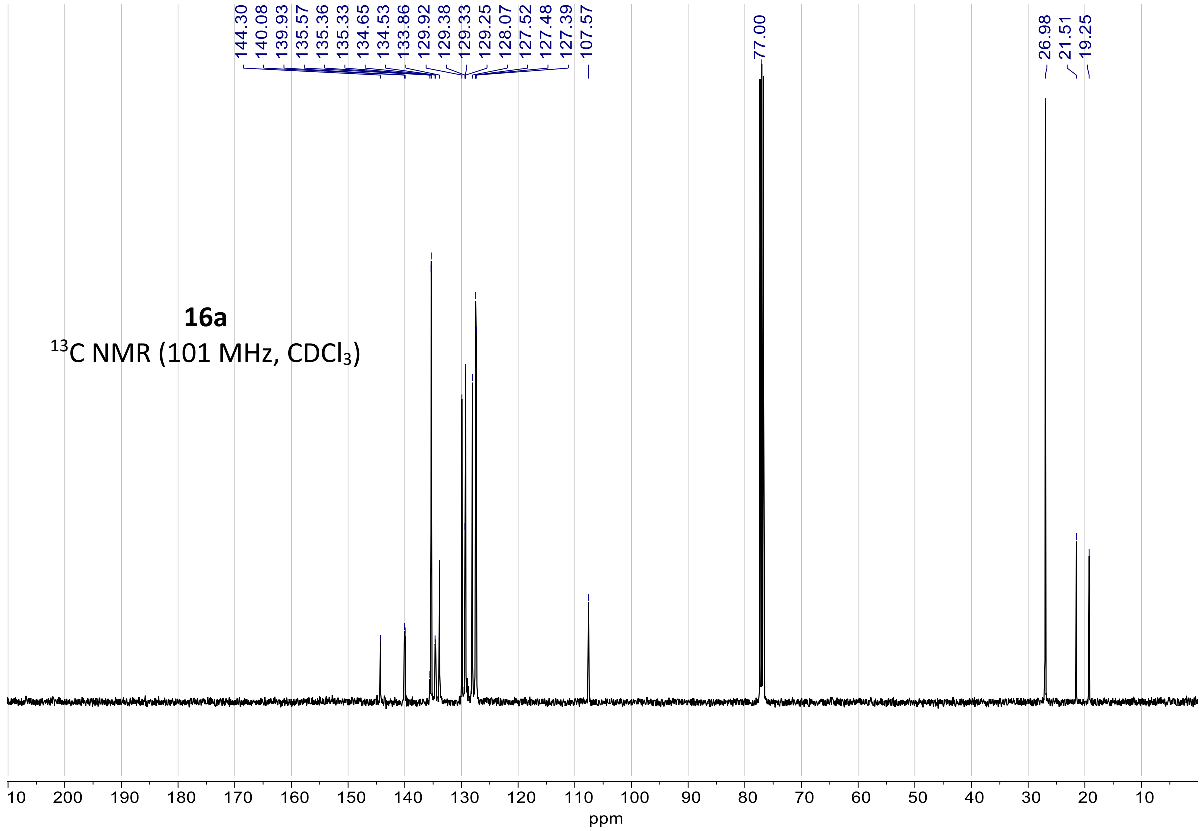

16a
^{13}C NMR (101 MHz, CDCl$_3$)
144.30 140.08 139.93 135.57 135.36 135.33 134.65 134.53 133.86 129.92 129.38 129.33 129.25 128.07 127.52 127.48 127.39 107.57
77.00
26.98 21.51 19.25
ppm

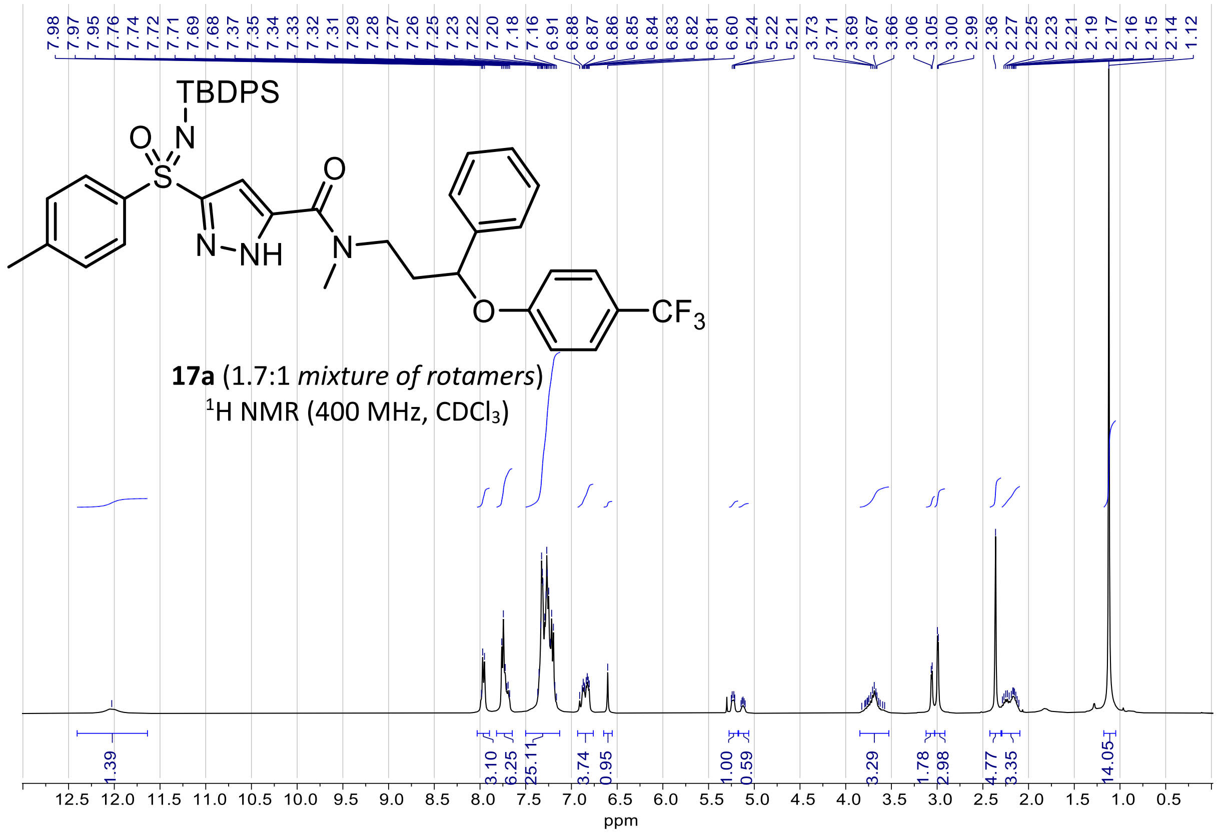
TBDPS
CF3
17a (1.7:1 mixture of rotamers)
1H NMR (400 MHz, CDCl3)
ppm

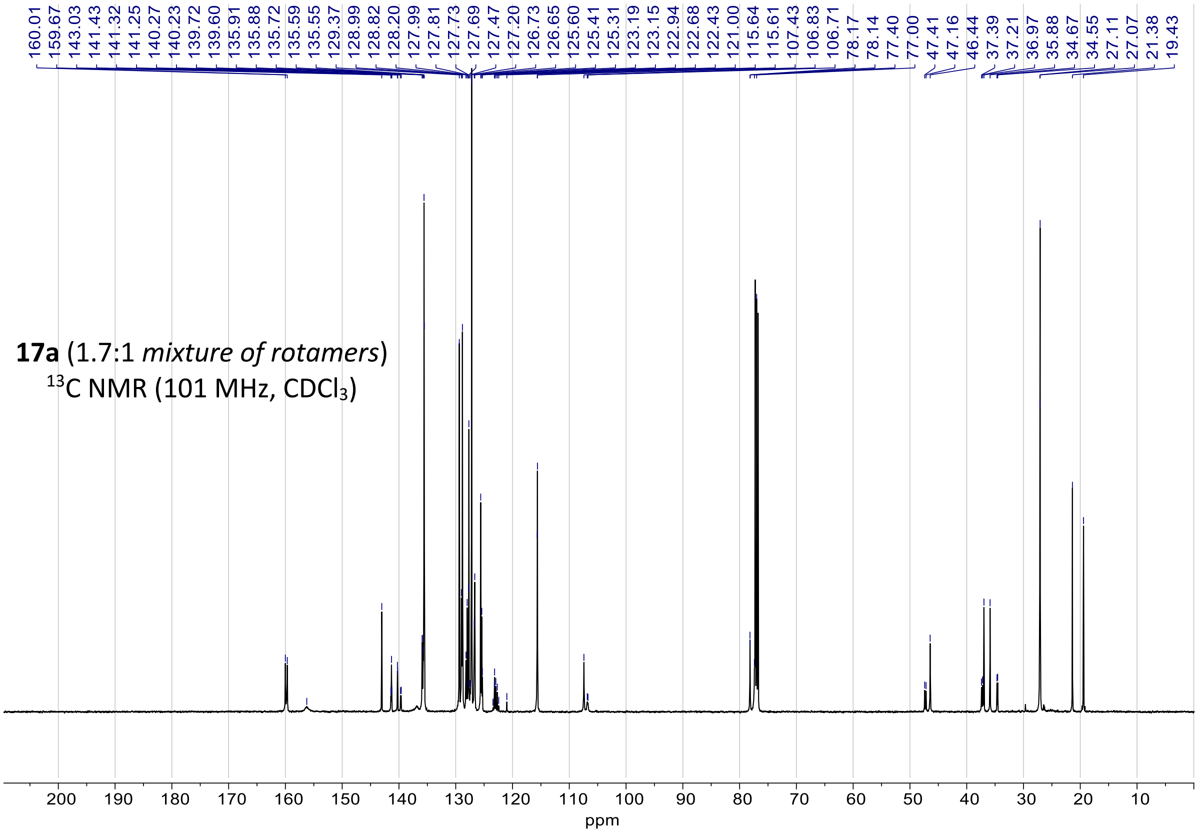
17a (1.7:1 mixture of rotamers)
13C NMR (101 MHz, CDCl3)
ppm

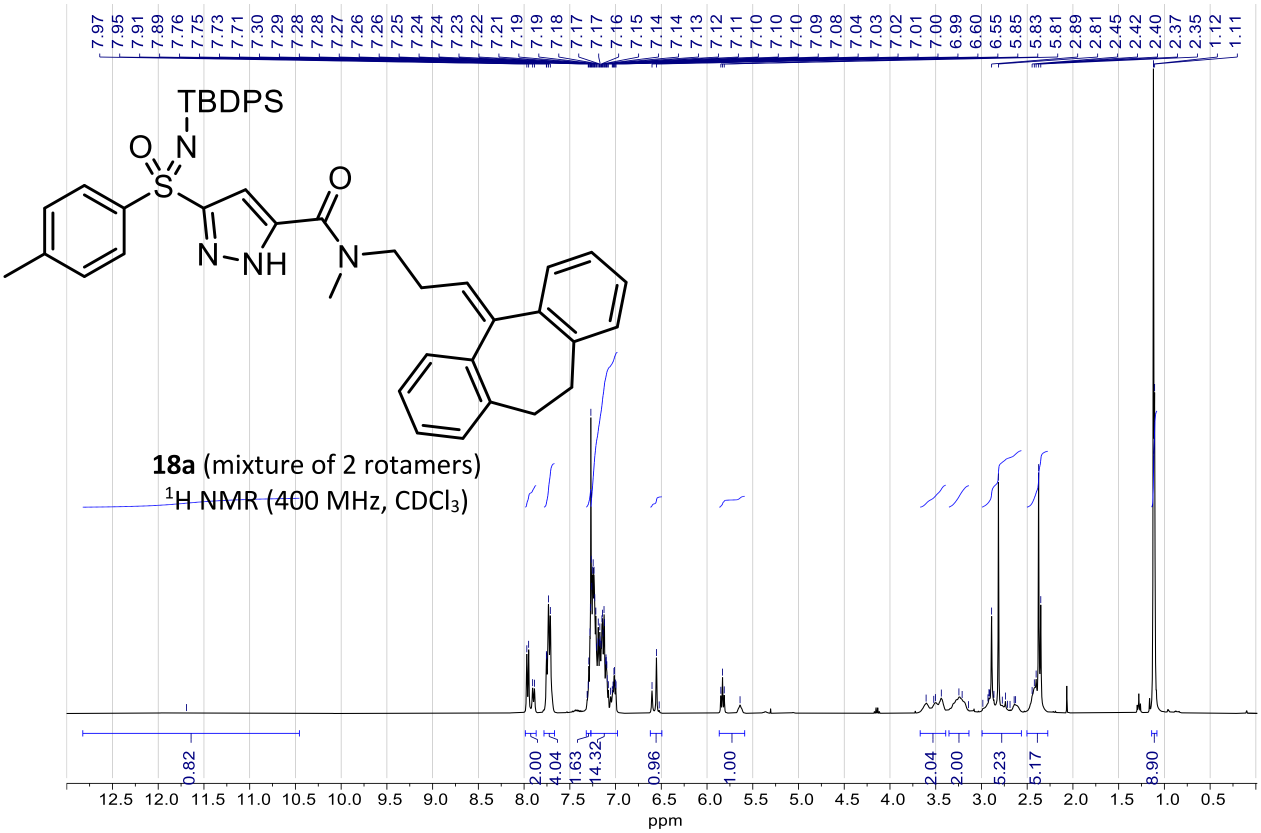
7.97 7.95 7.91 7.89 7.76 7.75 7.73 7.71 7.30 7.29 7.28 7.28 7.27 7.26 7.26 7.25 7.24 7.24 7.23 7.22 7.21 7.19 7.19 7.18 7.17 7.17 7.16 7.15 7.14 7.14 7.13 7.12 7.11 7.10 7.10 7.10 7.09 7.08 7.04 7.03 7.02 7.01 7.00 6.99 6.60 6.55 5.85 5.83 5.81 2.89 2.81 2.45 2.42 2.40 2.37 2.35 1.12 1.11
TBDPS
O
N
S
O
N
NH
N
18a (mixture of 2 rotamers)
¹H NMR (400 MHz, CDCl₃)
0.82 2.00 4.04 1.63 14.32 0.96 1.00 2.04 2.00 5.23 5.17 8.90
12.5 12.0 11.5 11.0 10.5 10.0 9.5 9.0 8.5 8.0 7.5 7.0 6.5 6.0 5.5 5.0 4.5 4.0 3.5 3.0 2.5 2.0 1.5 1.0 0.5
ppm

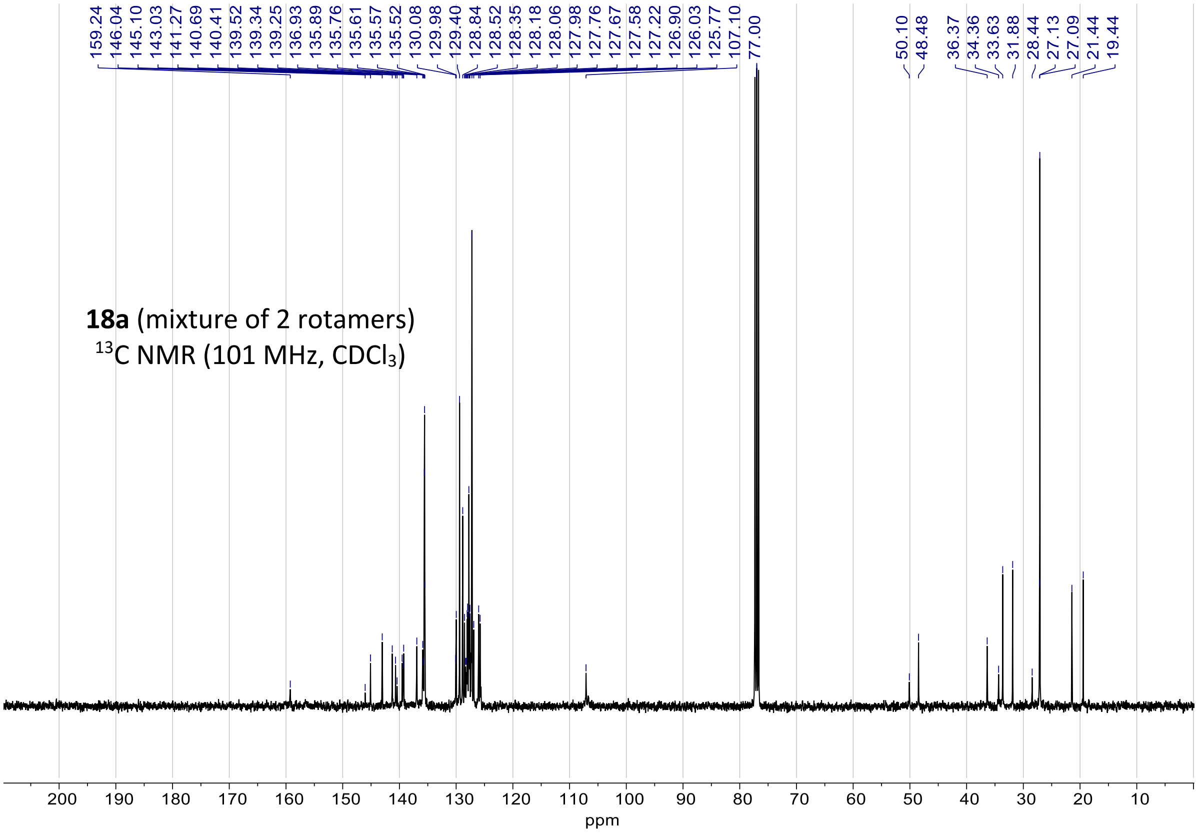
159.24 146.04 145.10 143.03 141.27 140.69 140.41 139.52 139.34 139.25 136.93 135.89 135.76 135.61 135.57 135.52 130.08 129.98 129.40 128.84 128.52 128.35 128.18 128.06 127.98 127.76 127.67 127.58 127.22 126.90 126.03 125.77 107.10 77.00
50.10 48.48 36.37 34.36 33.63 31.88 28.44 27.13 27.09 21.44 19.44
18a (mixture of 2 rotamers)
¹³C NMR (101 MHz, CDCl₃)
200 190 180 170 160 150 140 130 120 110 100 90 80 70 60 50 40 30 20 10
ppm

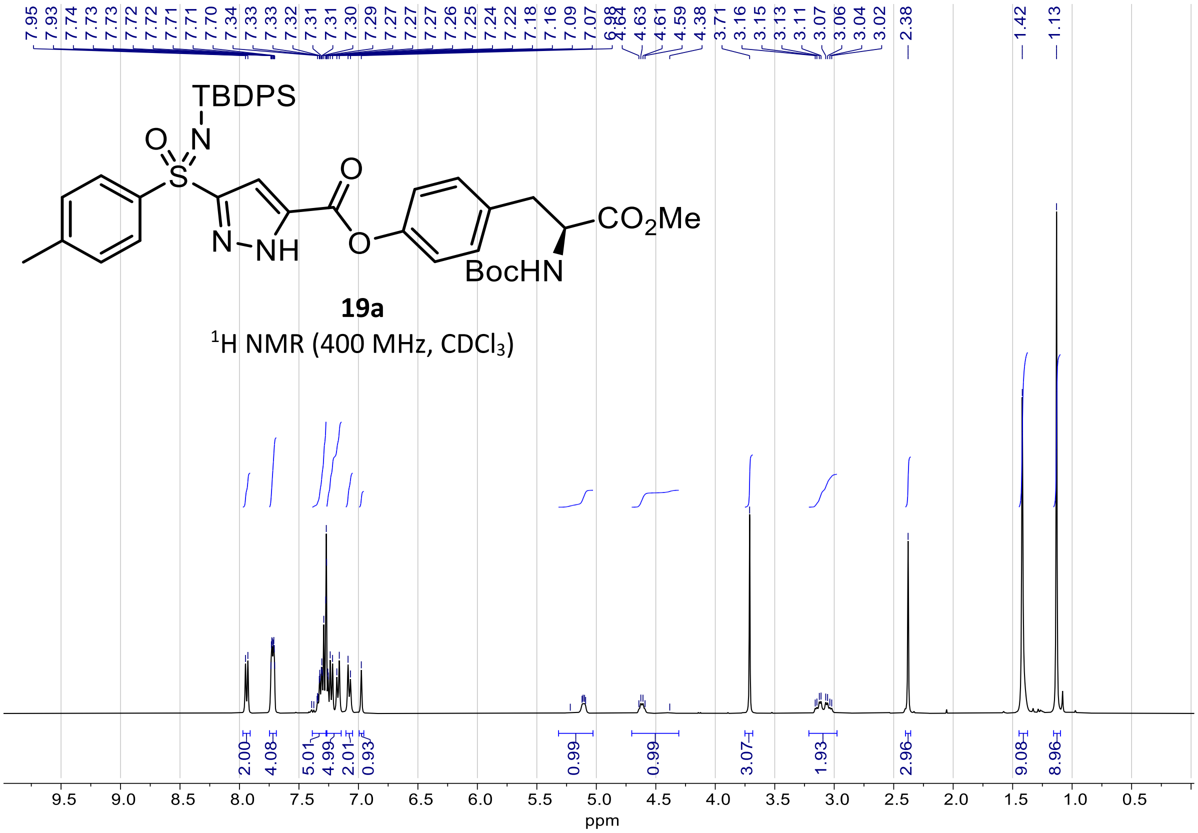
TBDPS
19a
^{1}H NMR (400 MHz, CDCl3)
ppm

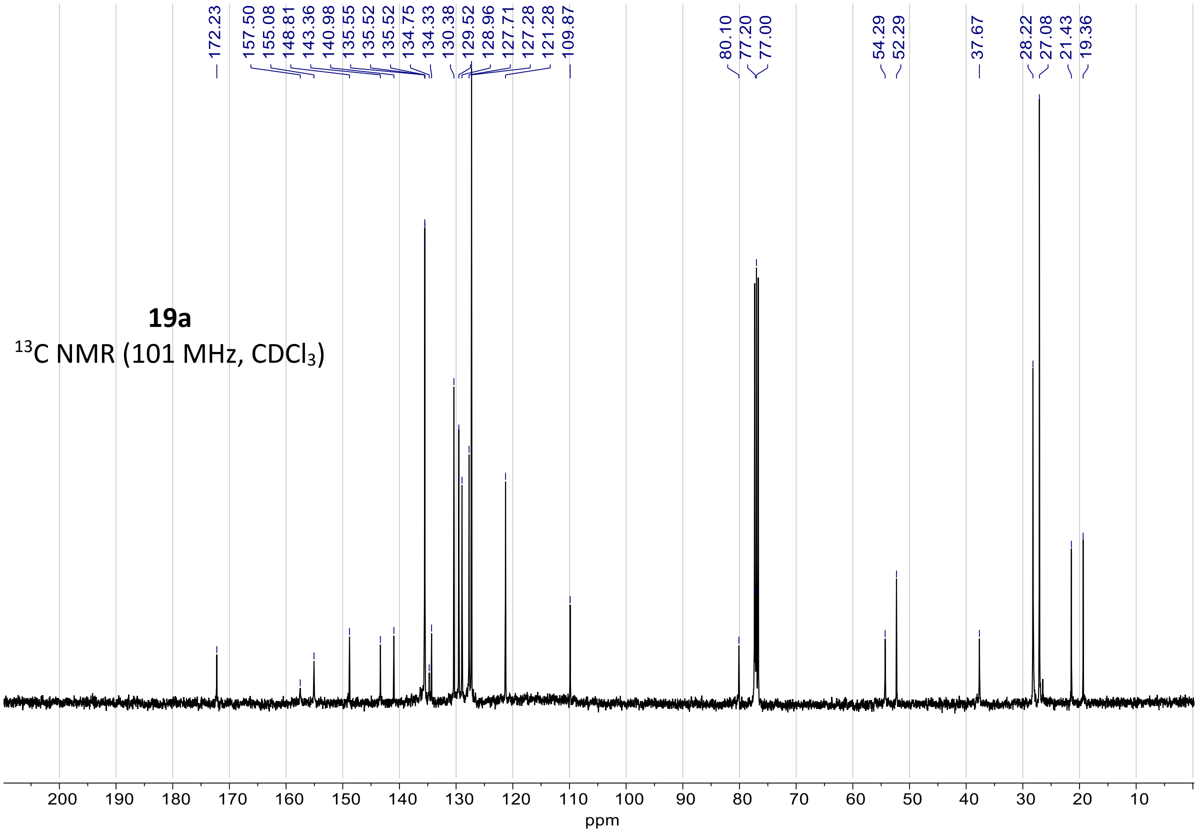
19a
^{13}C NMR (101 MHz, CDCl3)
ppm

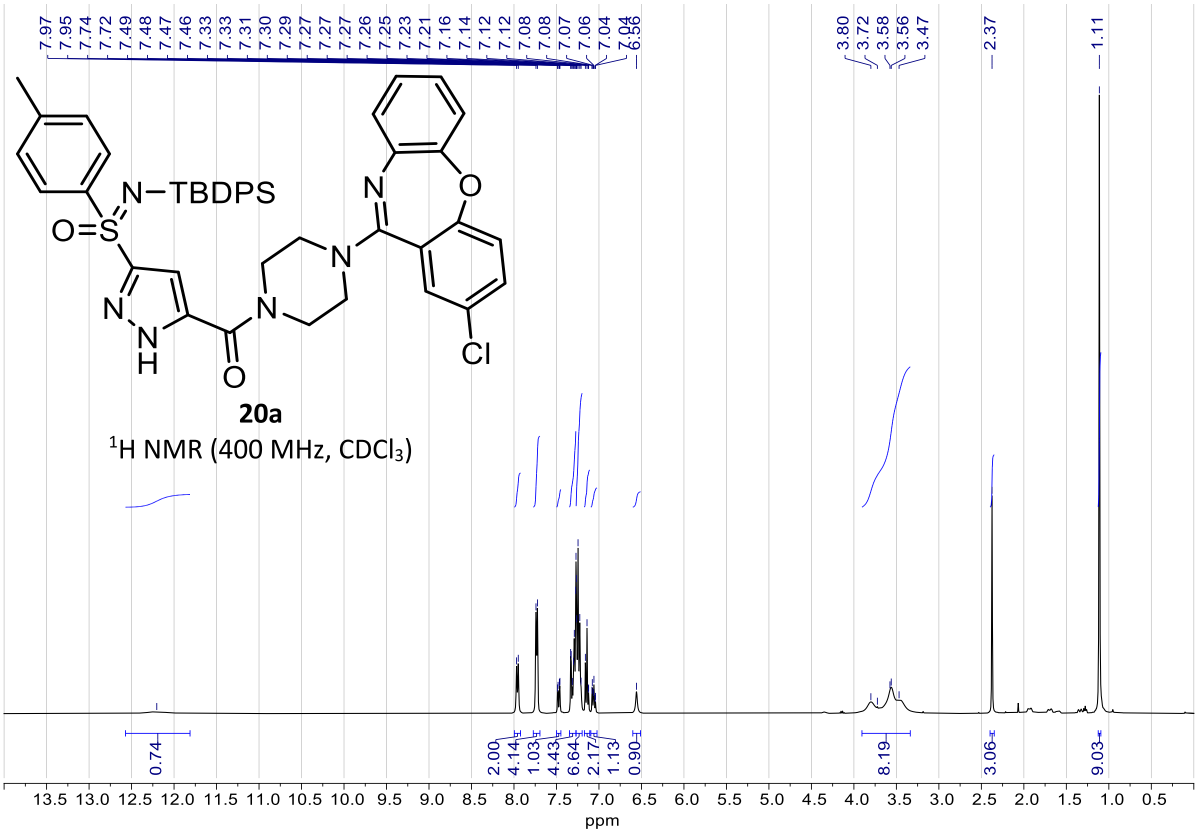

20a

^{1}H NMR (400 MHz, CDCl$_3$)

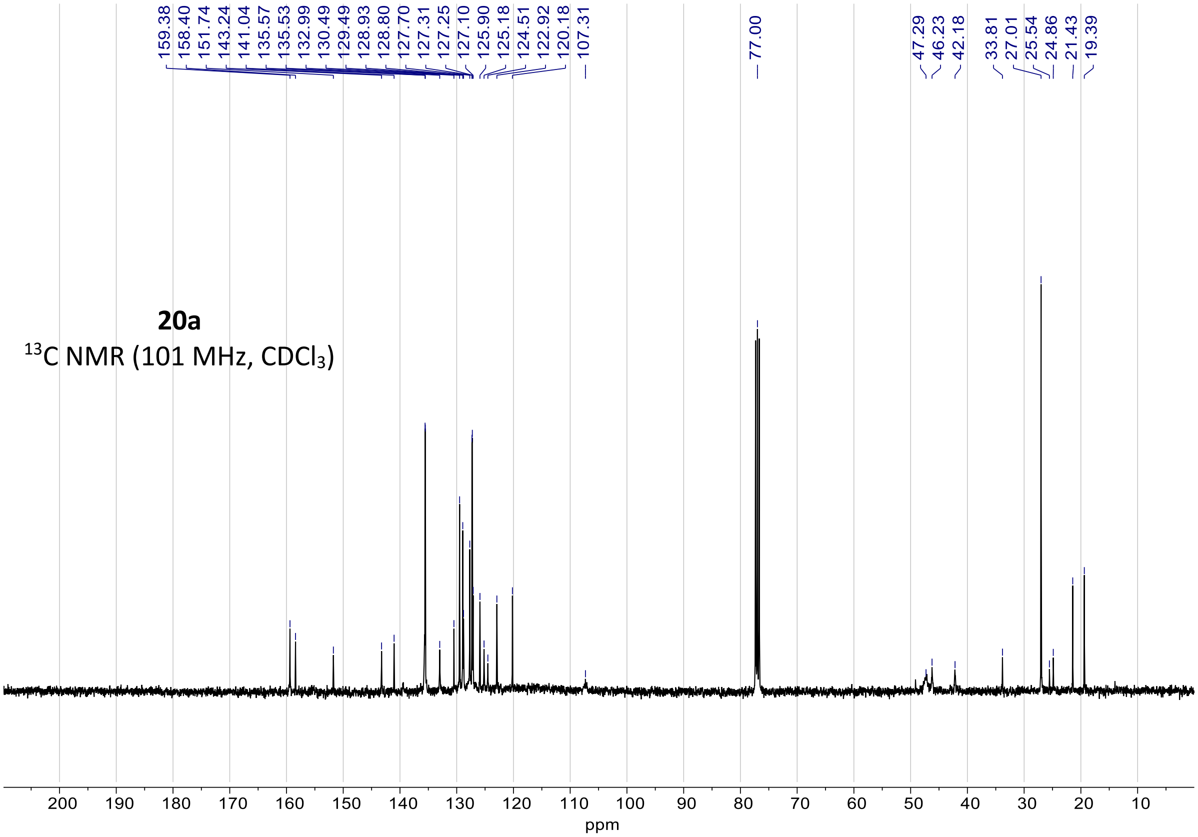

20a

^{13}C NMR (101 MHz, CDCl$_3$)

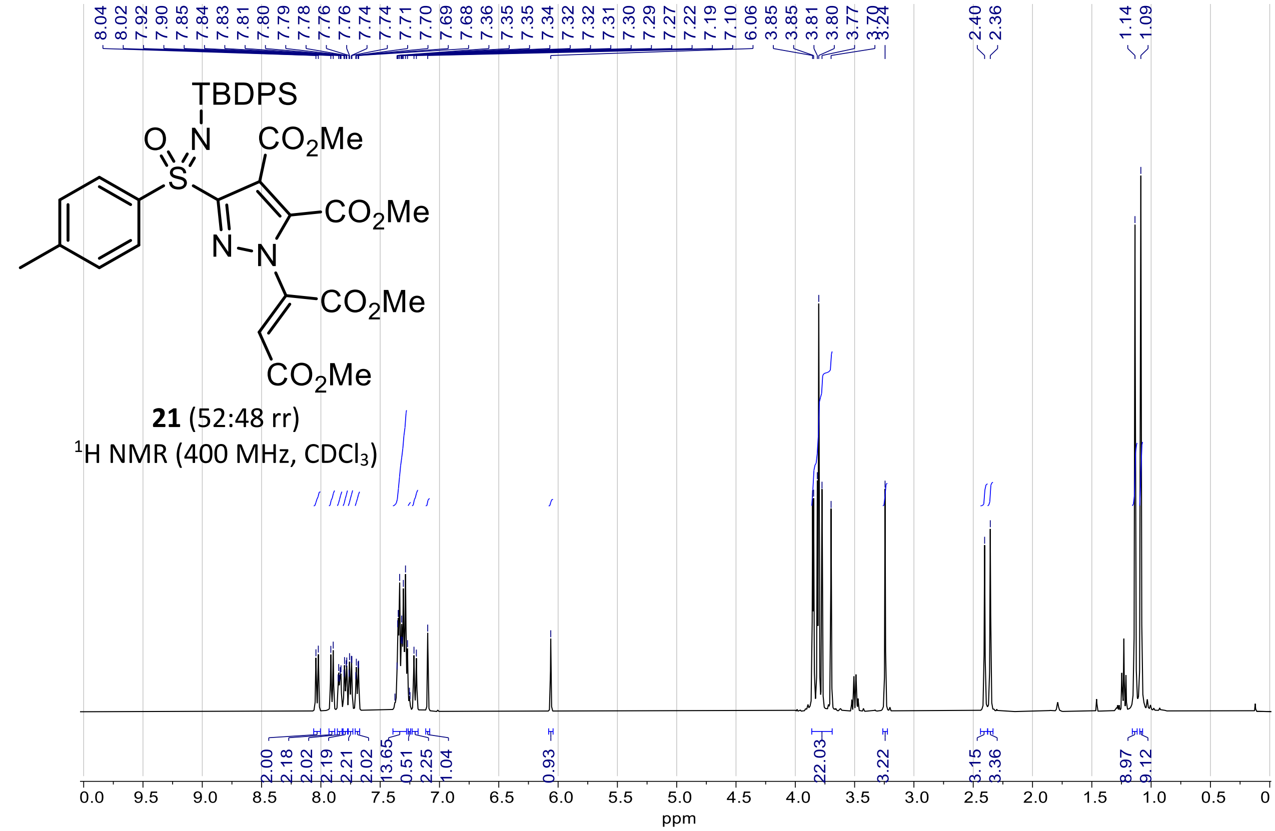
TBDPS
N
O
S
CO2Me
CO2Me
N
N
CO2Me
CO2Me
21 (52:48 rr)
1H NMR (400 MHz, CDCl3)
8.04 8.02 7.92 7.90 7.85 7.84 7.83 7.81 7.80 7.79 7.78 7.76 7.76 7.74 7.74 7.71 7.70 7.69 7.68 7.36 7.35 7.35 7.34 7.32 7.32 7.31 7.30 7.29 7.27 7.22 7.19 7.10 6.06 3.85 3.85 3.81 3.80 3.77 3.70 3.24 2.40 2.36 1.14 1.09
2.00 2.18 2.02 2.19 2.21 2.02 13.65 0.51 2.25 1.04 0.93 22.03 3.22 3.15 3.36 8.97 9.12
0.0 9.5 9.0 8.5 8.0 7.5 7.0 6.5 6.0 5.5 5.0 4.5 4.0 3.5 3.0 2.5 2.0 1.5 1.0 0.5 0
ppm

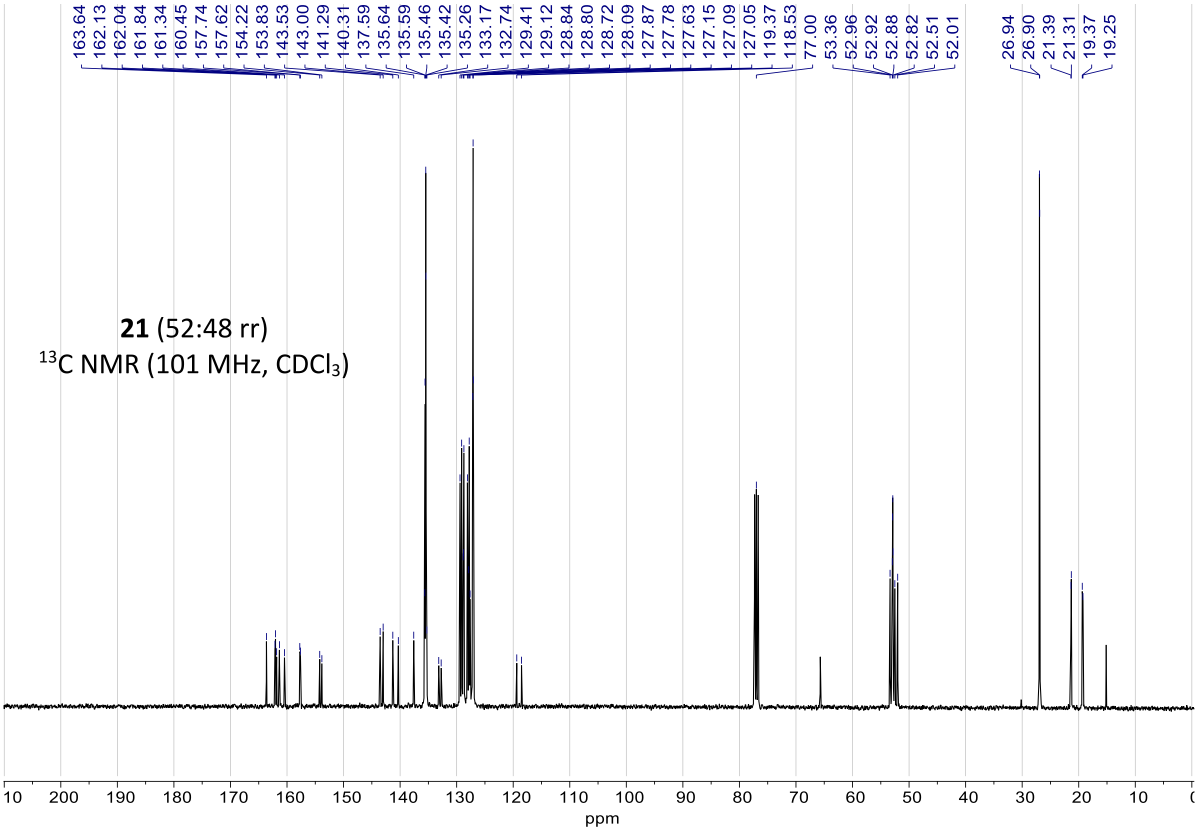
21 (52:48 rr)
13C NMR (101 MHz, CDCl3)
163.64 162.13 162.04 161.84 161.34 160.45 157.74 157.62 154.22 153.83 143.53 143.00 141.29 140.31 137.59 135.64 135.59 135.46 135.42 135.26 133.17 132.74 129.41 129.12 128.84 128.80 128.72 128.09 127.87 127.78 127.63 127.15 127.09 127.05 119.37 118.53 77.00 53.36 52.96 52.92 52.88 52.82 52.51 52.01 26.94 26.90 21.39 21.31 19.37 19.25
10 200 190 180 170 160 150 140 130 120 110 100 90 80 70 60 50 40 30 20 10
ppm

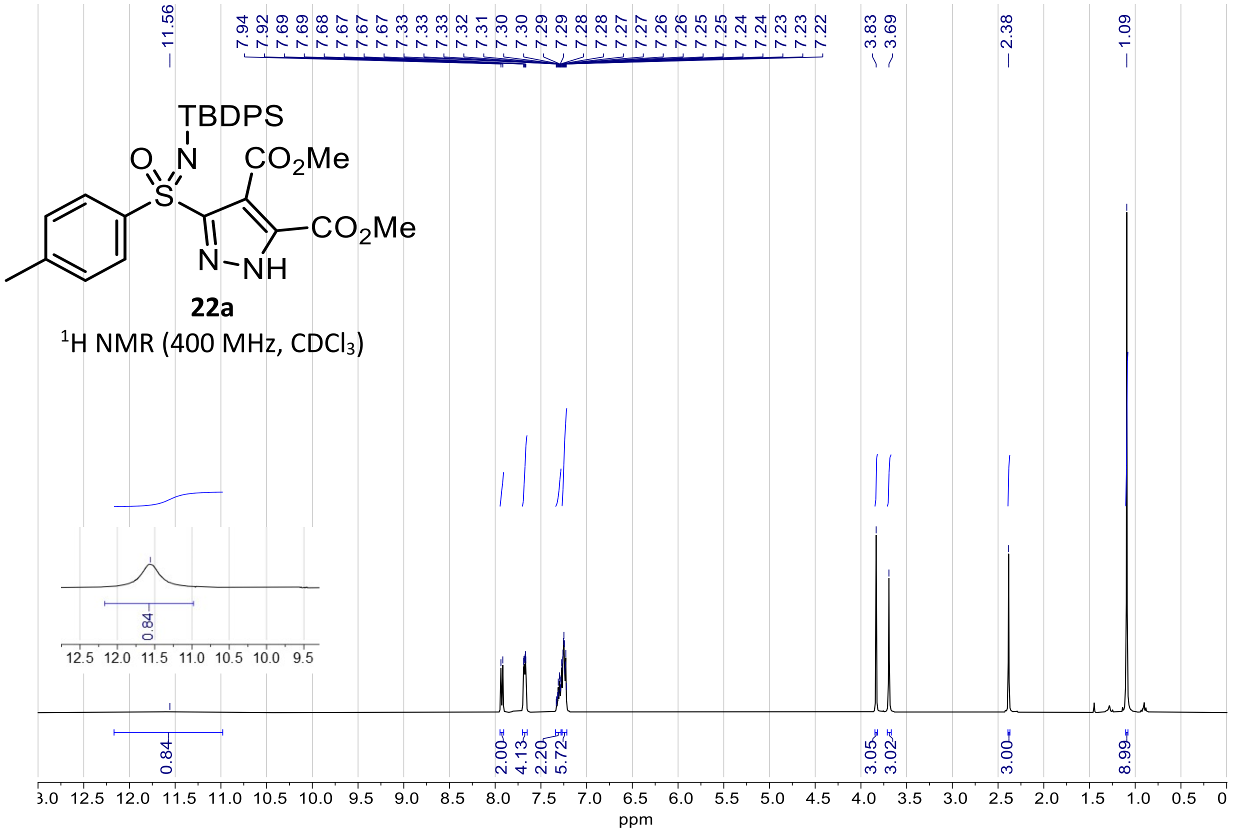
11.56
7.94 7.92 7.69 7.68 7.67 7.67 7.67 7.33 7.33 7.33 7.32 7.31 7.30 7.30 7.29 7.29 7.28 7.28 7.27 7.27 7.26 7.26 7.25 7.25 7.24 7.24 7.23 7.23 7.22
3.83 3.69
2.38
1.09
TBDPS
N
O
S
CO2Me
CO2Me
N
NH
22a
1H NMR (400 MHz, CDCl3)
0.84
12.5 12.0 11.5 11.0 10.5 10.0 9.5
2.00 4.13 2.20 5.72
3.05 3.02
3.00
8.99
ppm

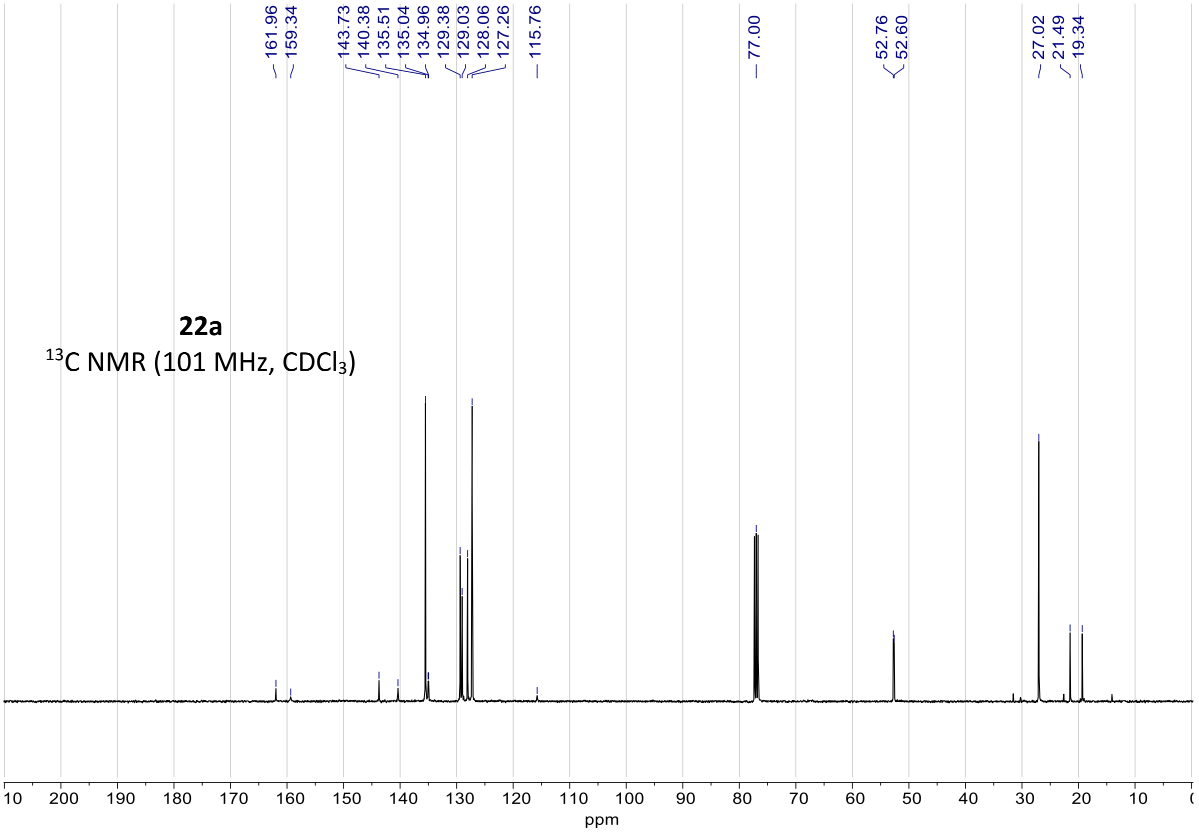
161.96 159.34
143.73 140.38 135.51 135.04 134.96 129.38 129.03 128.06 127.26
115.76
77.00
52.76 52.60
27.02 21.49 19.34
22a
13C NMR (101 MHz, CDCl3)
ppm

22a
HSQC ($CDCl_3$)

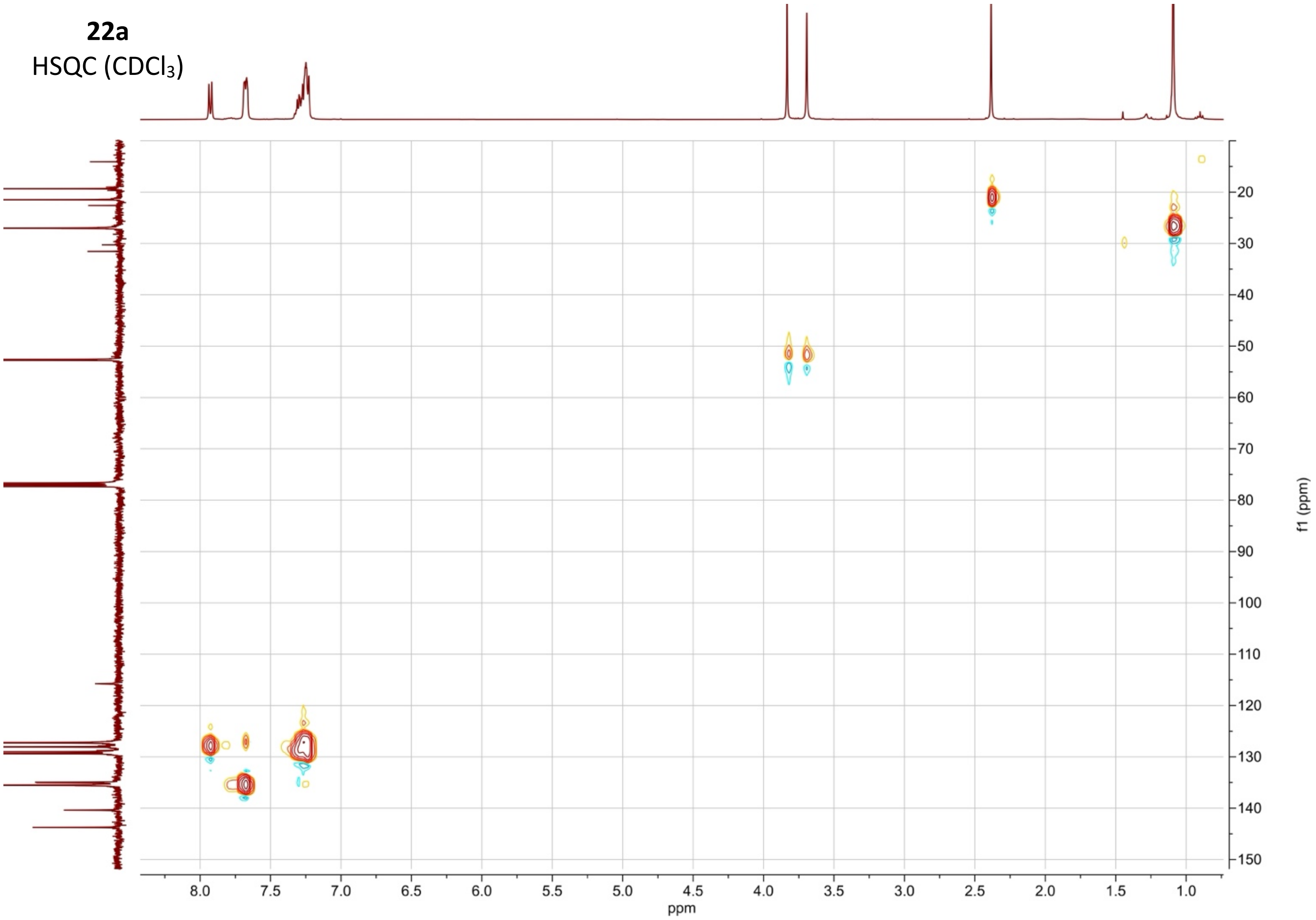

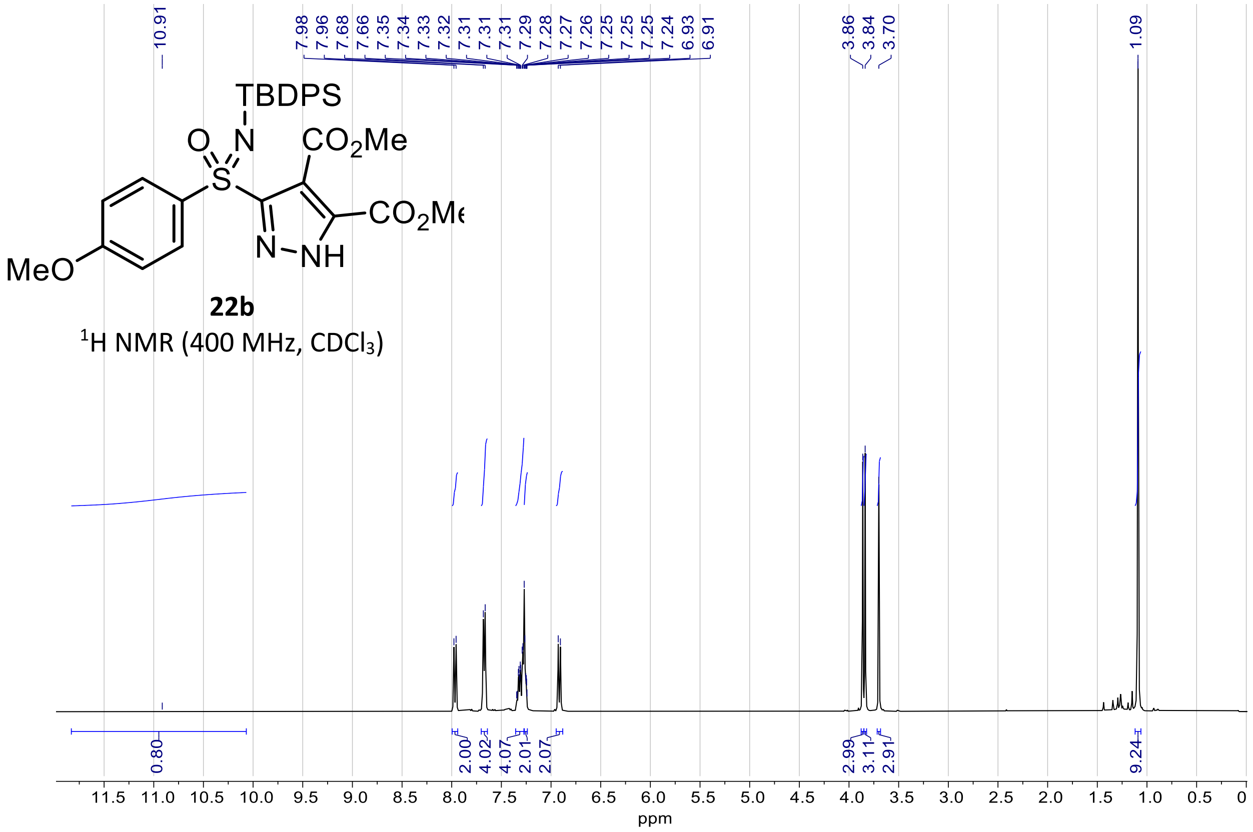

22b

^1H NMR (400 MHz, $CDCl_3$)

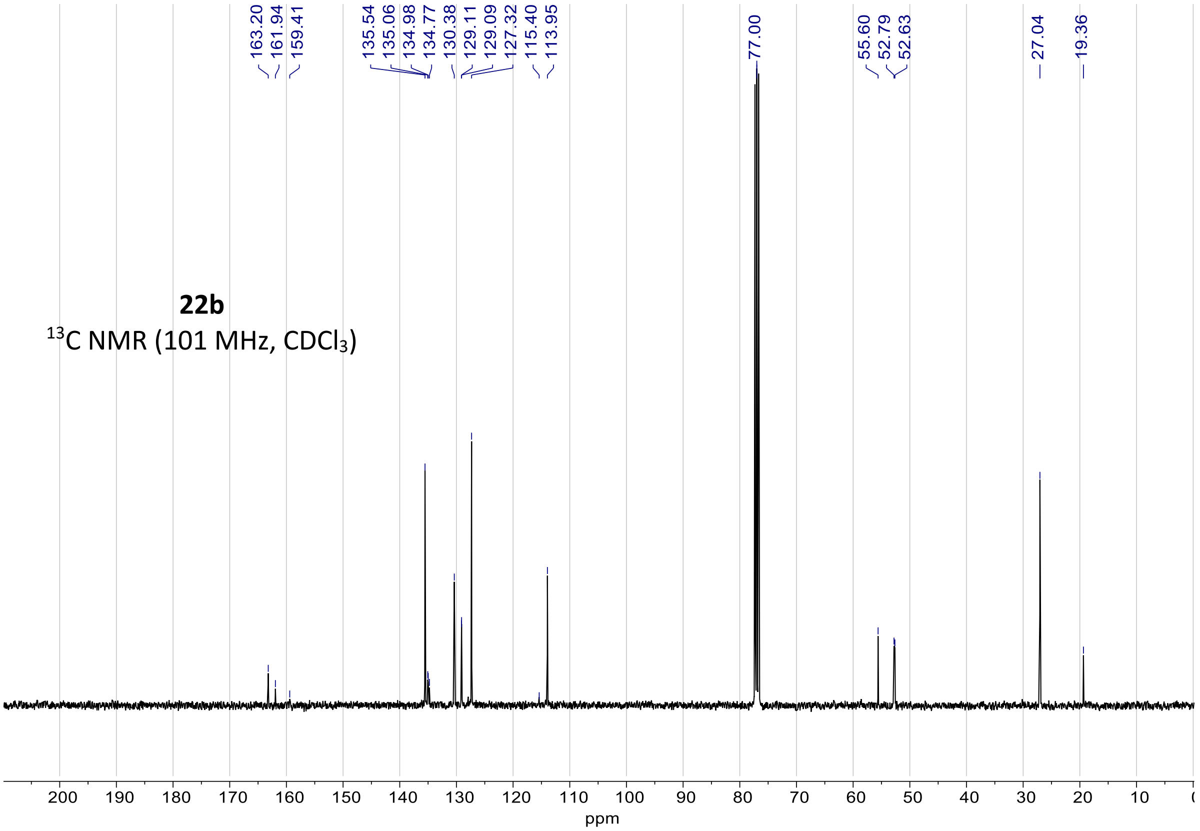

22b

^{13}C NMR (101 MHz, $CDCl_3$)

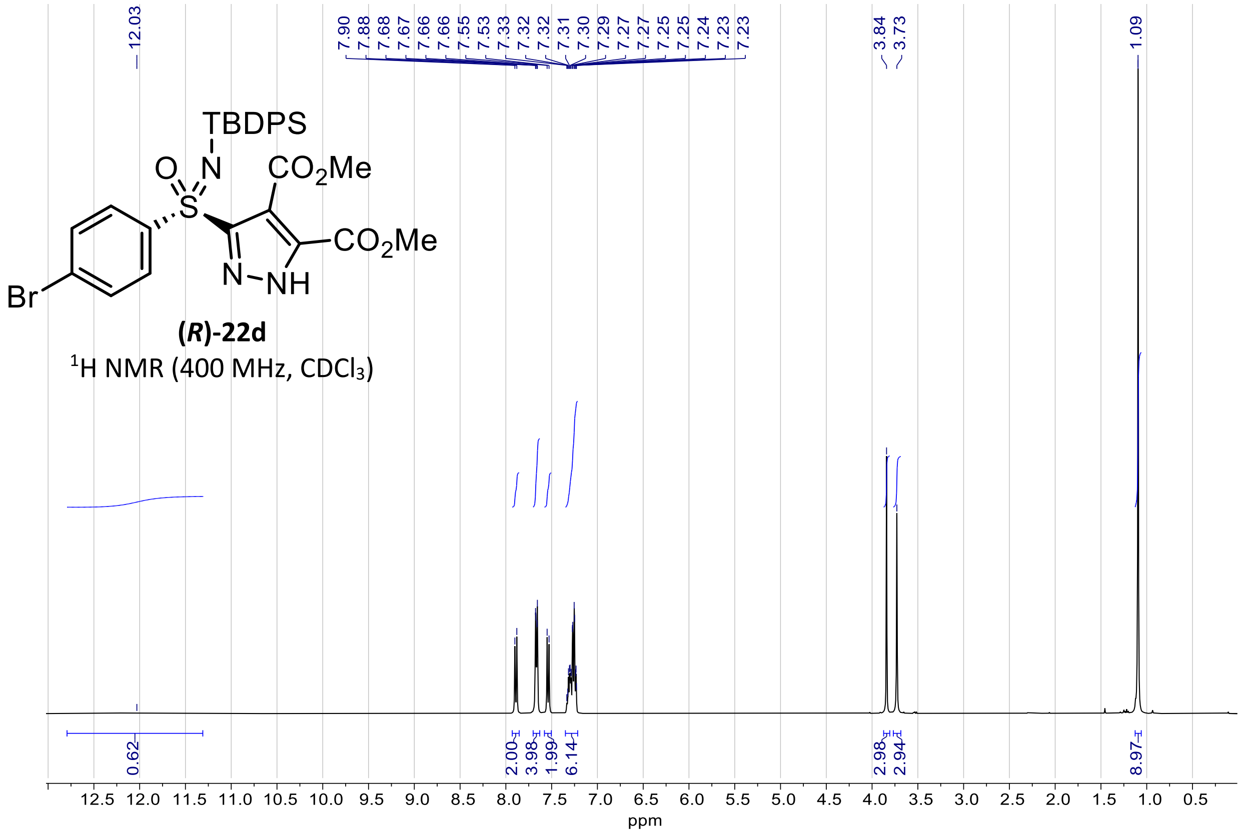

(*R*)-22d

^{1}H NMR (400 MHz, $CDCl_3$)

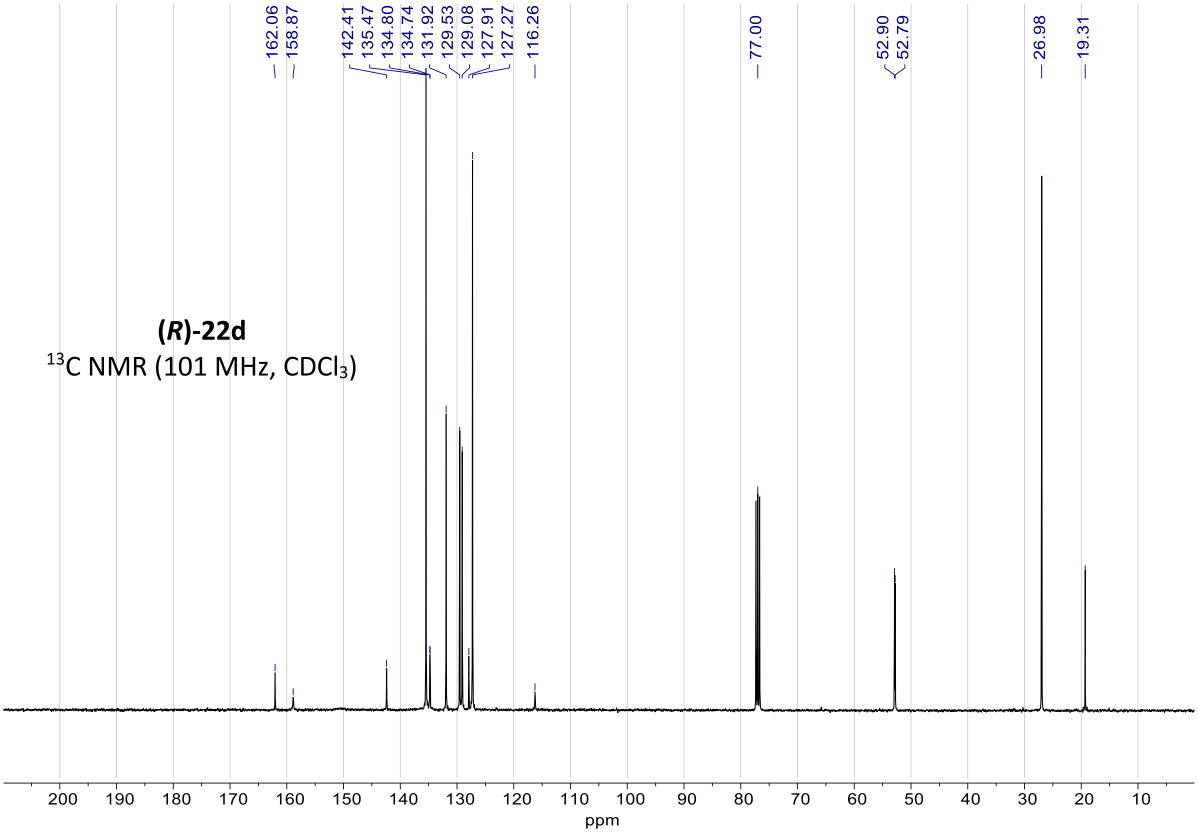

(*R*)-22d

^{13}C NMR (101 MHz, $CDCl_3$)

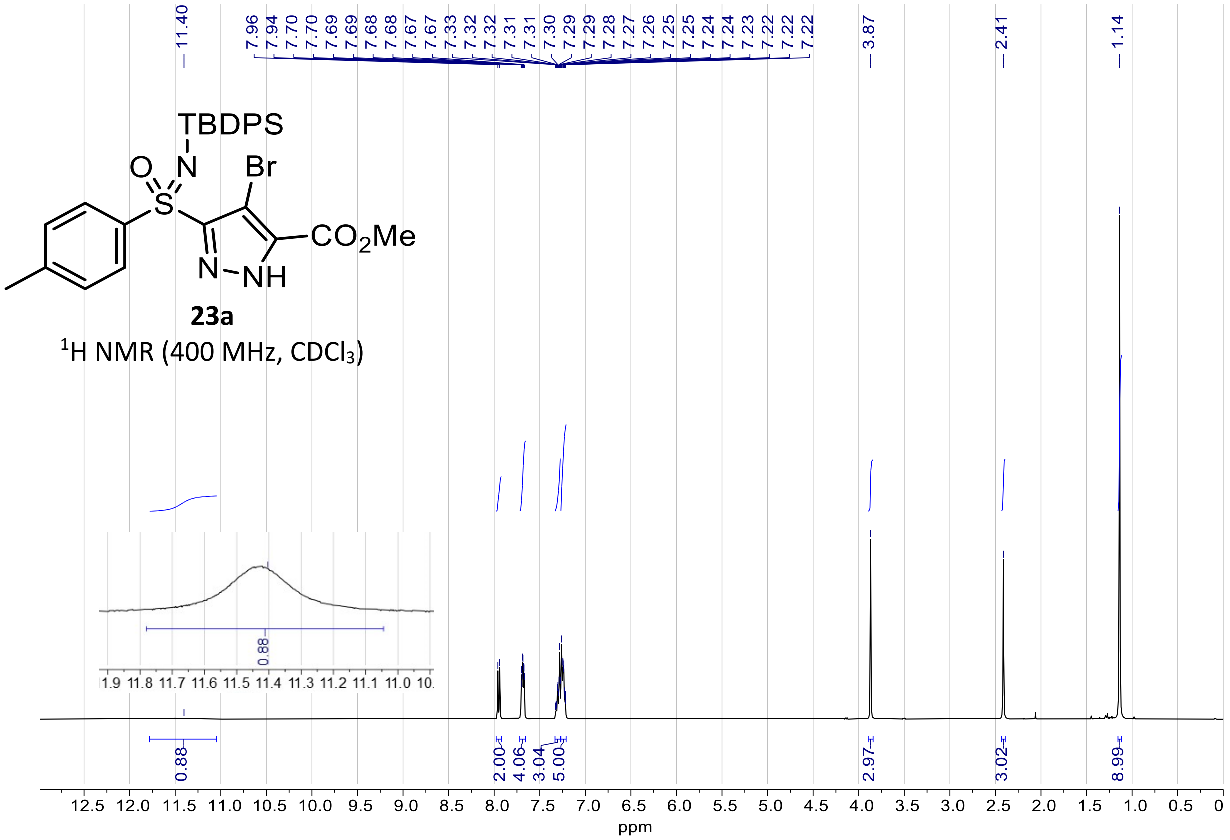
TBDPS
O
N
Br
S
CO$_2$Me
N
NH
23a
^{1}H NMR (400 MHz, CDCl$_3$)
11.40
7.96 7.94 7.70 7.70 7.69 7.69 7.68 7.68 7.67 7.67 7.33 7.32 7.32 7.31 7.31 7.30 7.29 7.29 7.28 7.27 7.26 7.25 7.25 7.24 7.24 7.23 7.22 7.22 7.22
3.87
2.41
1.14
0.88
2.00 4.06 3.04 5.00 2.97 3.02 8.99
ppm

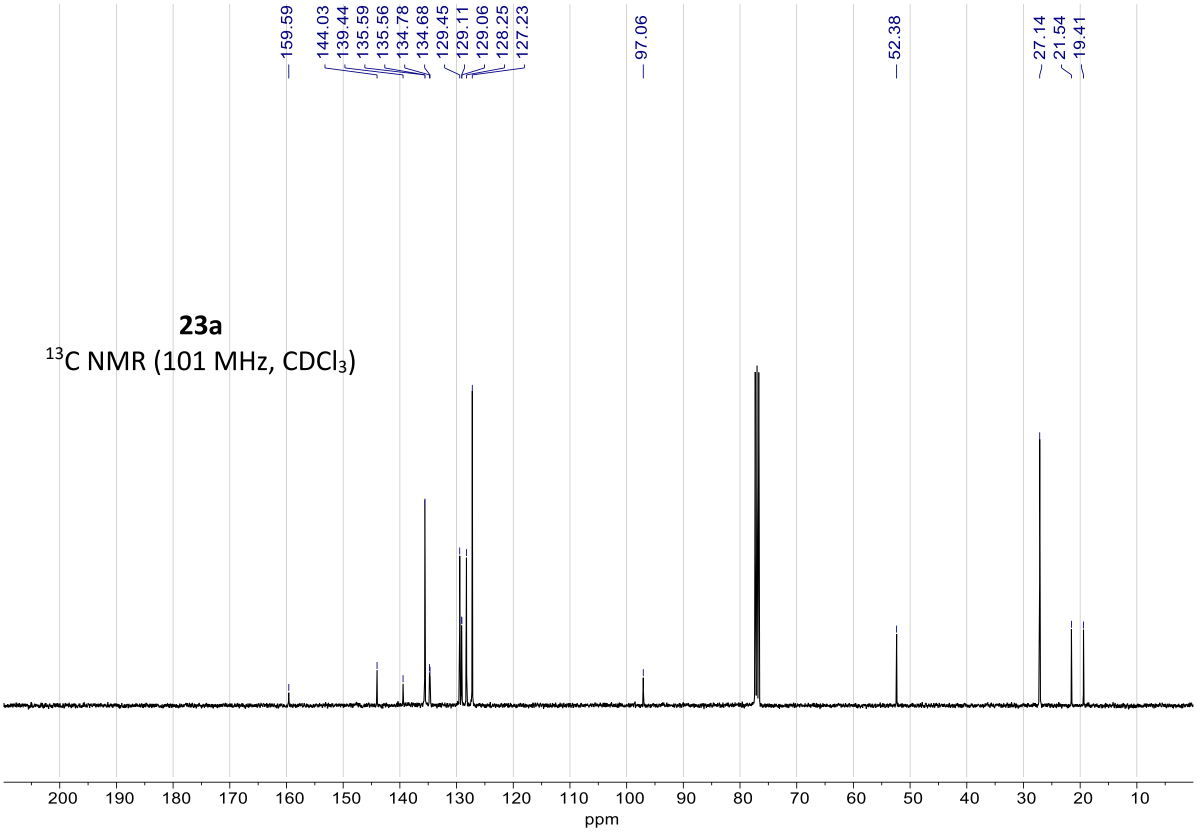
159.59
144.03 139.44 135.59 135.56 134.78 134.68 129.45 129.11 129.06 128.25 127.23
97.06
52.38
27.14 21.54 19.41
23a
^{13}C NMR (101 MHz, CDCl$_3$)
ppm

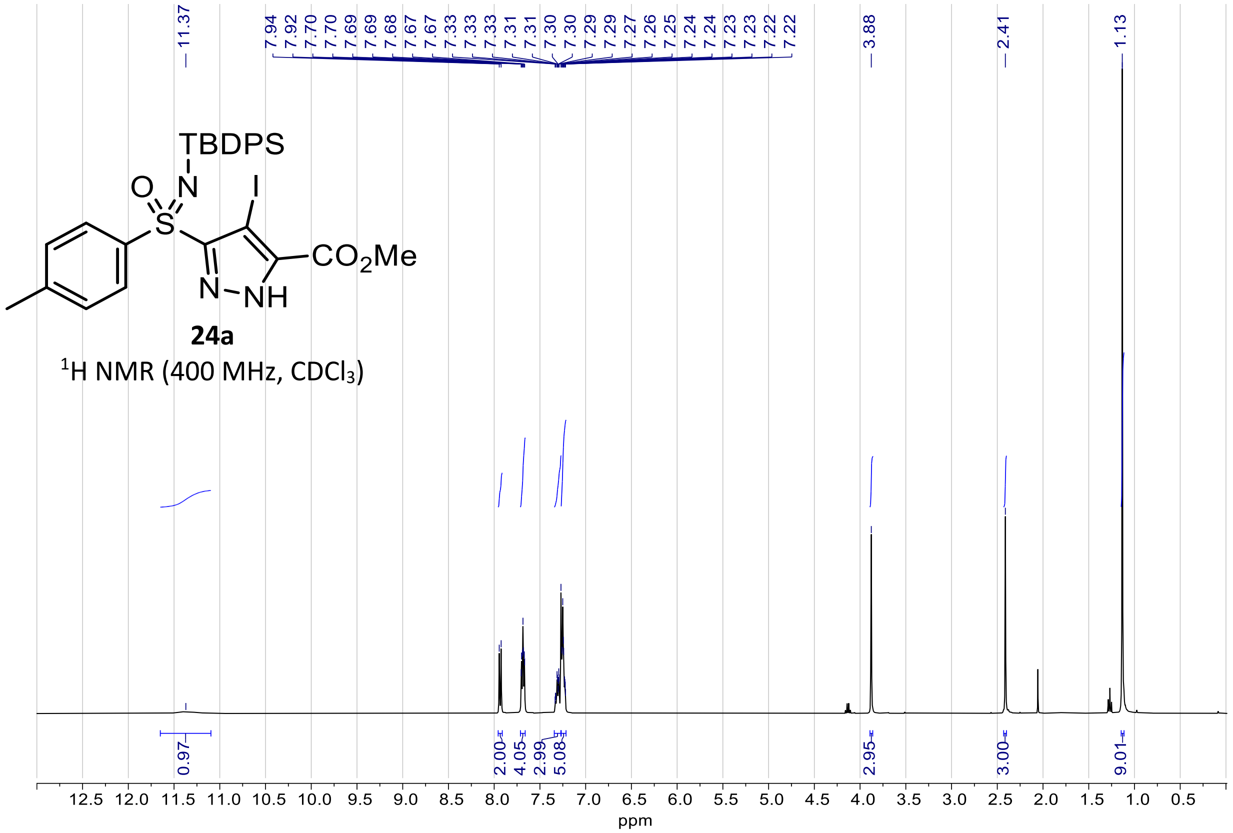
TBDPS
N
O
S
I
CO2Me
N
NH
24a
1H NMR (400 MHz, CDCl3)
11.37
7.94 7.92 7.70 7.70 7.69 7.68 7.67 7.67 7.33 7.33 7.33 7.31 7.31 7.30 7.30 7.29 7.29 7.27 7.26 7.25 7.24 7.24 7.23 7.23 7.22 7.22
3.88
2.41
1.13
0.97
2.00
4.05
2.99
5.08
2.95
3.00
9.01
12.5 12.0 11.5 11.0 10.5 10.0 9.5 9.0 8.5 8.0 7.5 7.0 6.5 6.0 5.5 5.0 4.5 4.0 3.5 3.0 2.5 2.0 1.5 1.0 0.5
ppm

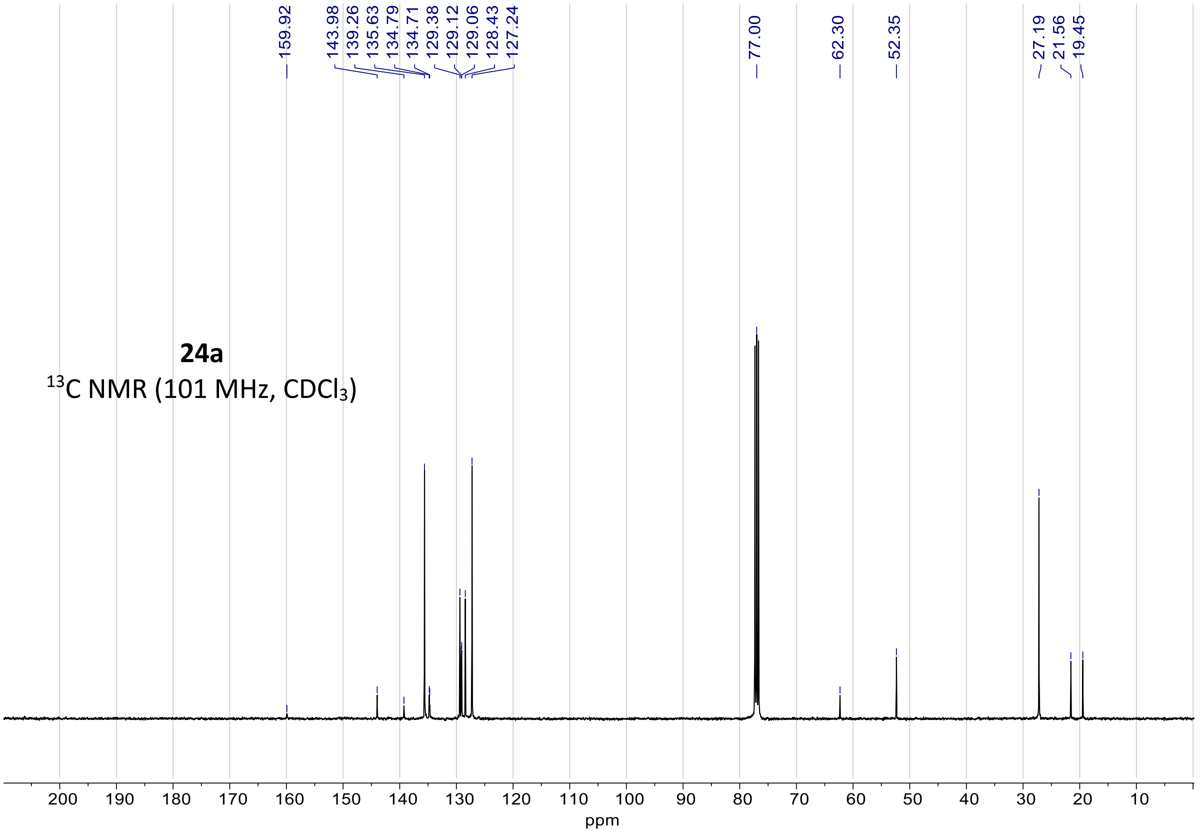
159.92
143.98 139.26 135.63 134.79 134.71 129.38 129.12 129.06 128.43 127.24
77.00
62.30
52.35
27.19 21.56 19.45
24a
13C NMR (101 MHz, CDCl3)
200 190 180 170 160 150 140 130 120 110 100 90 80 70 60 50 40 30 20 10
ppm

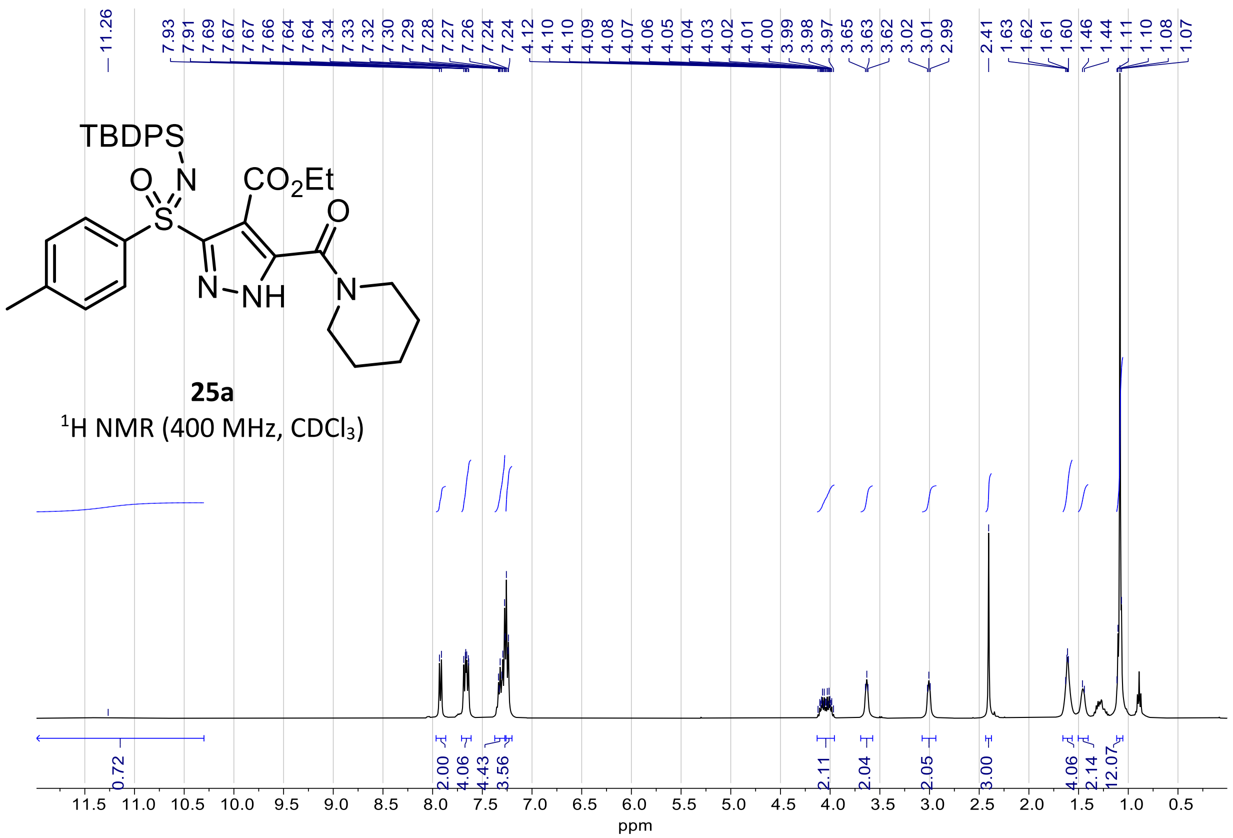
TBDPS
N
O
S
CO2Et
O
N
NH
N
25a
1H NMR (400 MHz, CDCl3)
11.26
7.93 7.91 7.69 7.67 7.67 7.66 7.64 7.64 7.34 7.33 7.32 7.30 7.29 7.28 7.27 7.26 7.24 7.24
4.12 4.10 4.10 4.09 4.08 4.07 4.06 4.05 4.04 4.03 4.02 4.01 4.00 3.99 3.98 3.97
3.65 3.63 3.62 3.02 3.01 2.99 2.41 1.63 1.62 1.61 1.60 1.46 1.44 1.11 1.10 1.08 1.07
0.72 2.00 4.06 4.43 3.56 2.11 2.04 2.05 3.00 4.06 2.14 12.07
11.5 11.0 10.5 10.0 9.5 9.0 8.5 8.0 7.5 7.0 6.5 6.0 5.5 5.0 4.5 4.0 3.5 3.0 2.5 2.0 1.5 1.0 0.5
ppm

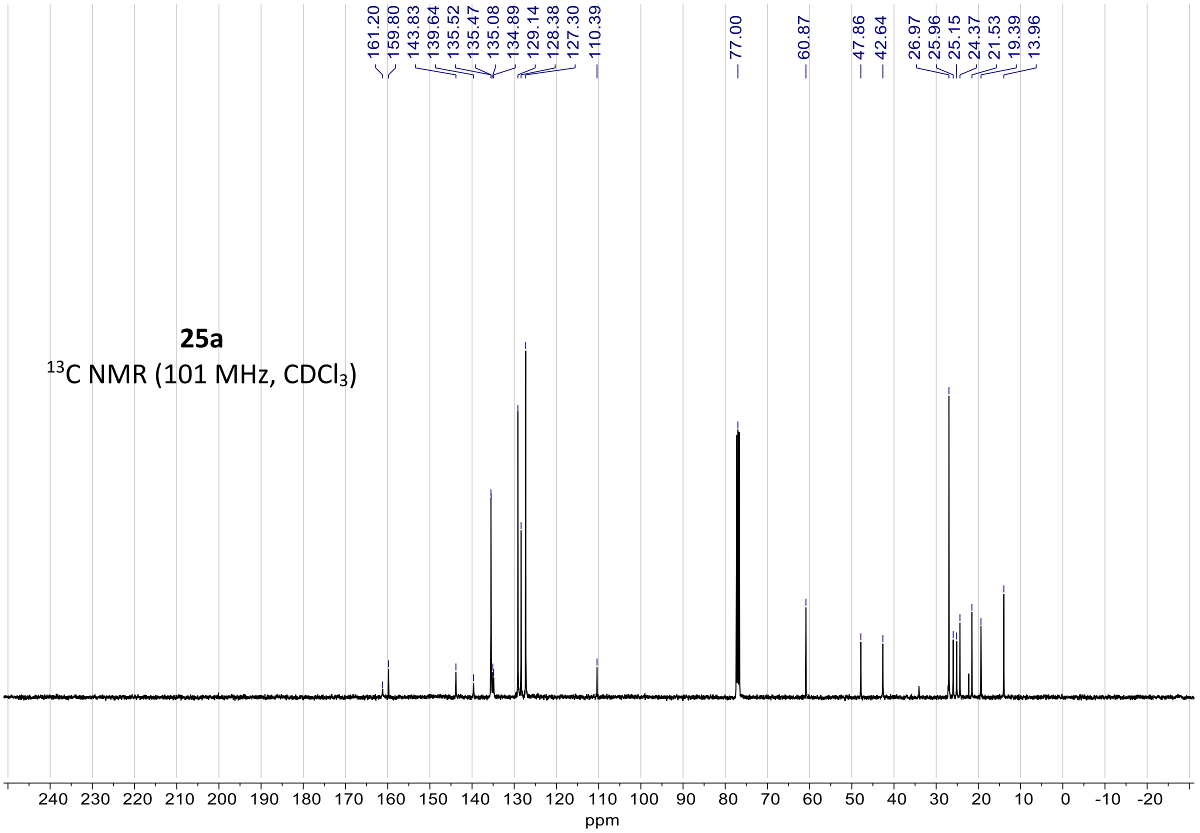
25a
13C NMR (101 MHz, CDCl3)
161.20 159.80 143.83 139.64 135.52 135.47 135.08 134.89 129.14 128.38 127.30 110.39
77.00 60.87 47.86 42.64 26.97 25.96 25.15 24.37 21.53 19.39 13.96
240 230 220 210 200 190 180 170 160 150 140 130 120 110 100 90 80 70 60 50 40 30 20 10 0 -10 -20
ppm

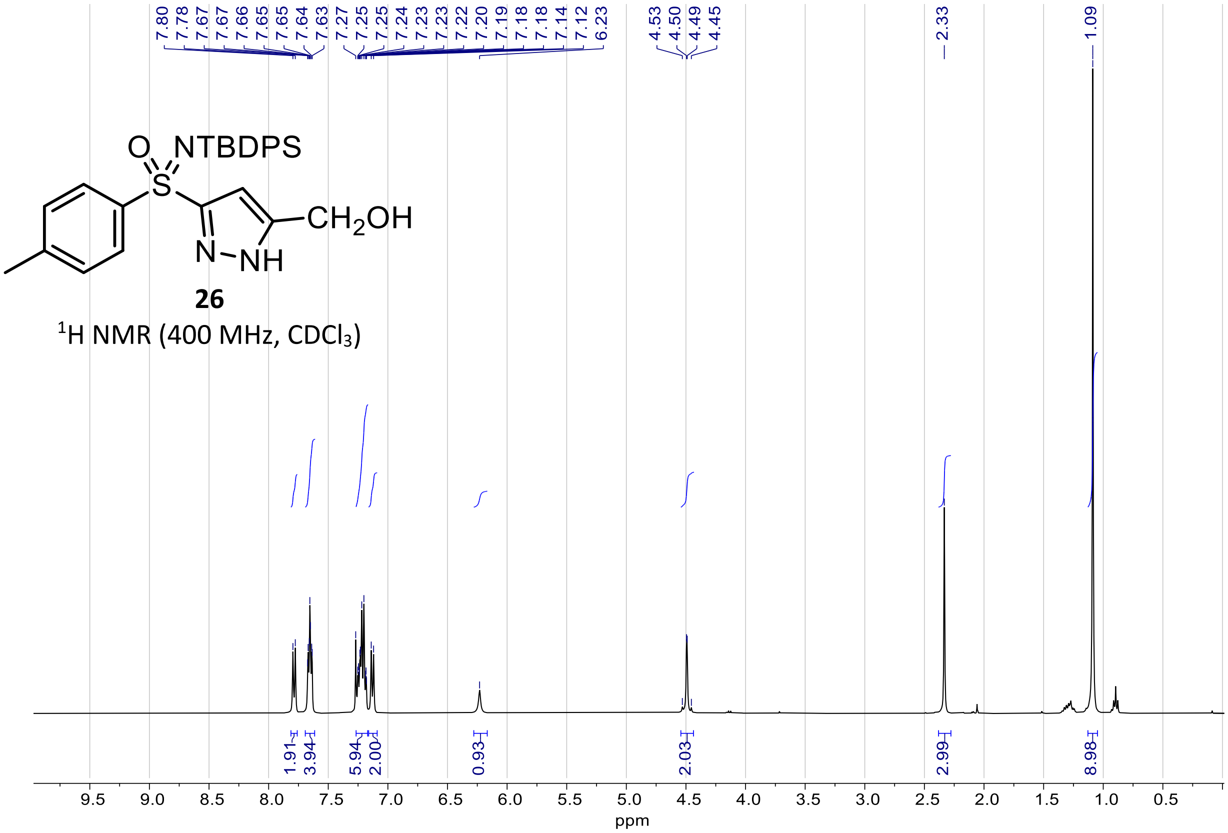

26

^{1}H NMR (400 MHz, $CDCl_3$)

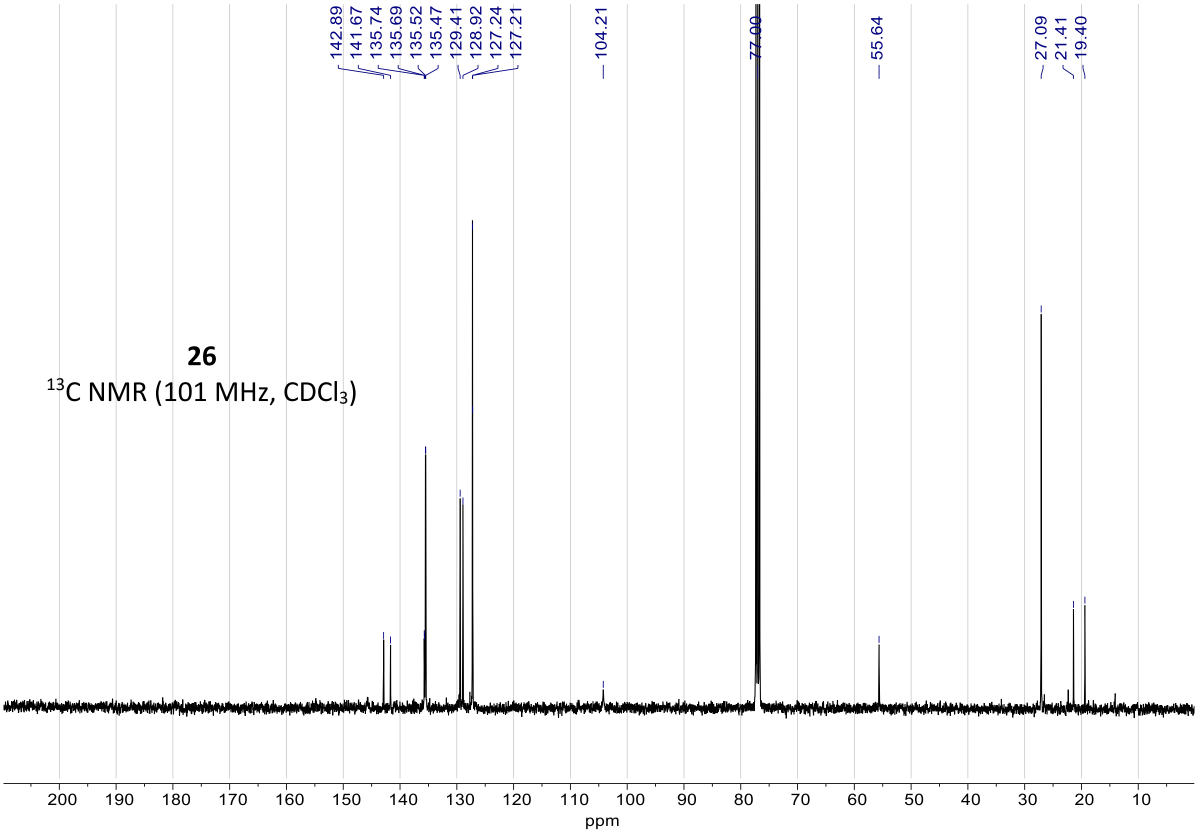

26

^{13}C NMR (101 MHz, $CDCl_3$)

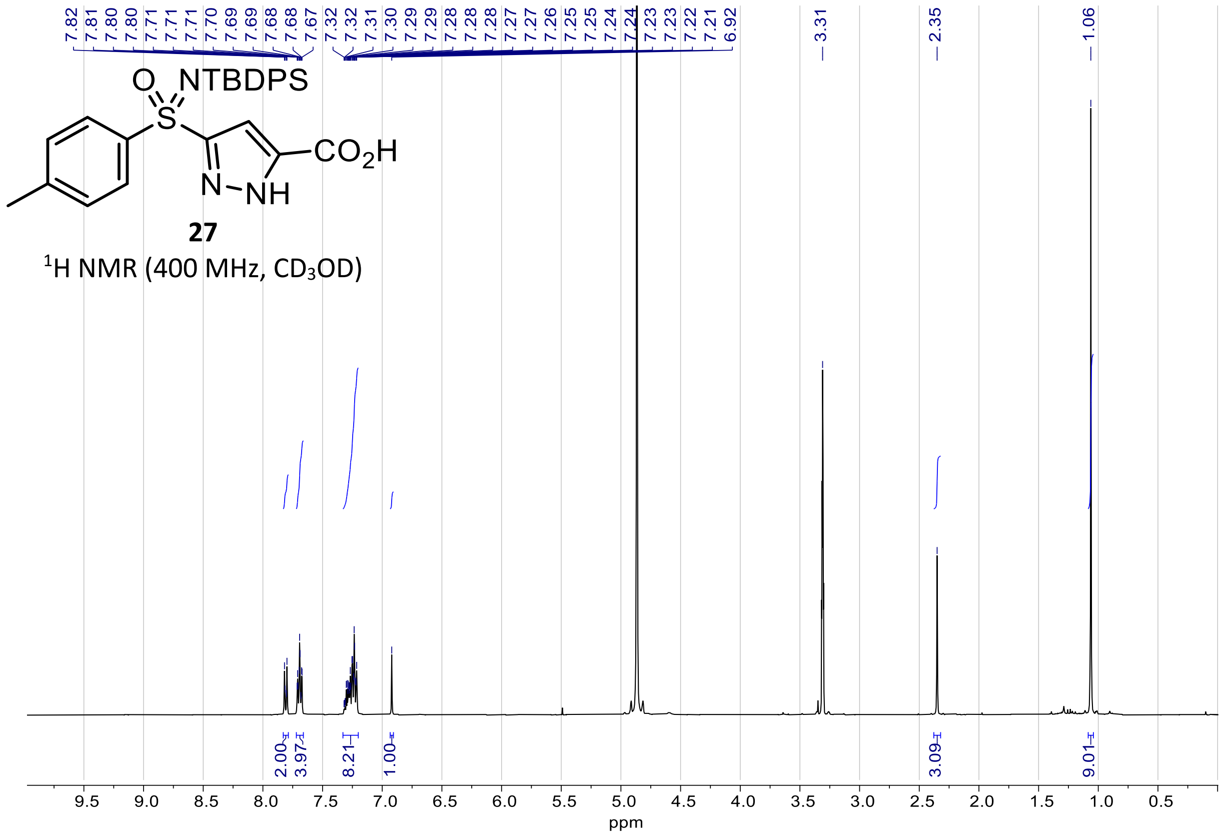
O NTBDPS
S
CO2H
N–NH
27
1H NMR (400 MHz, CD3OD)
ppm

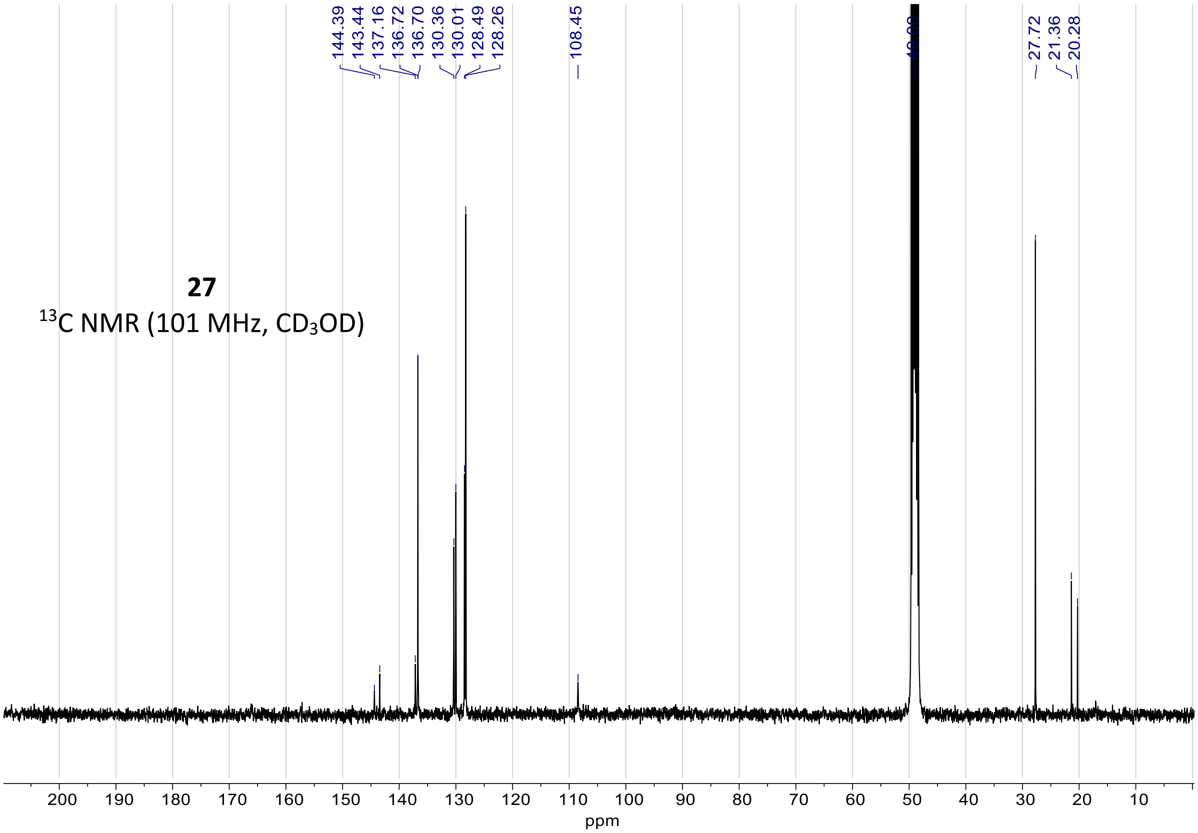
27
13C NMR (101 MHz, CD3OD)
ppm

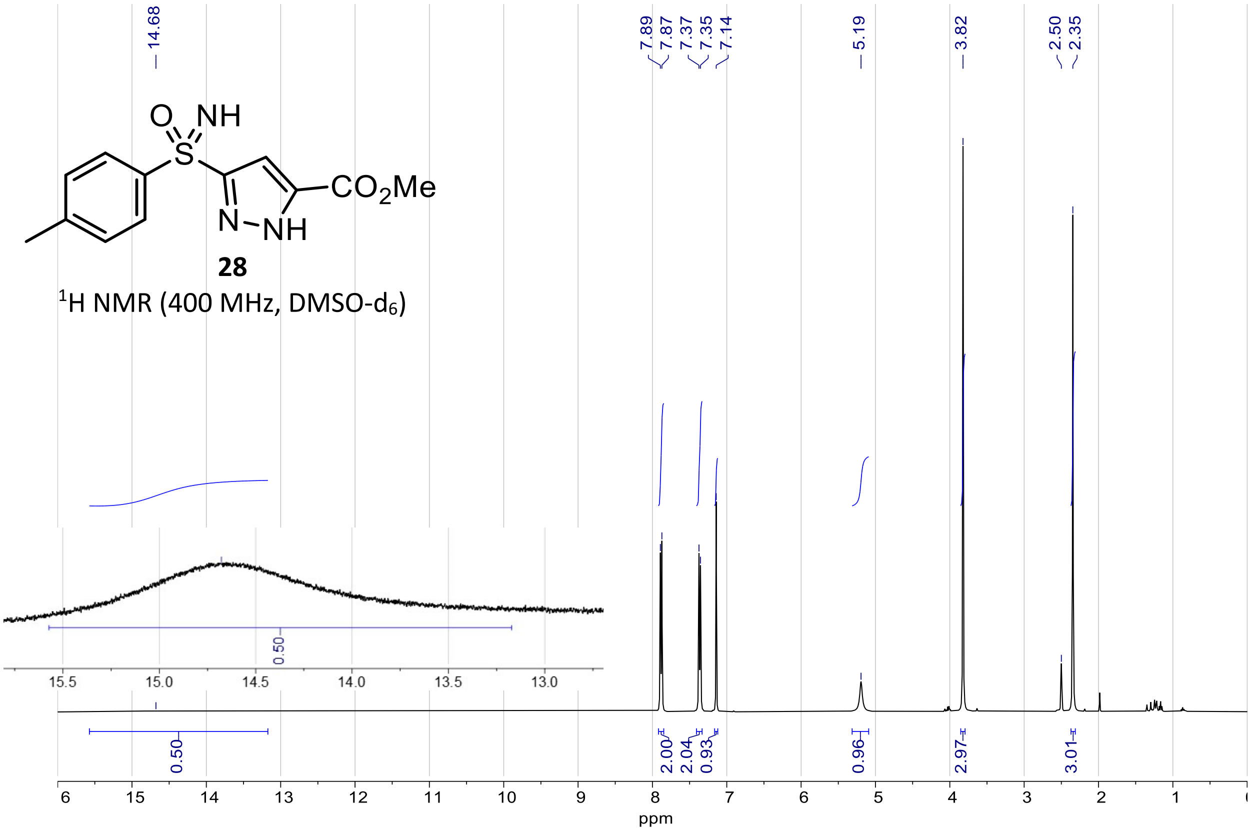

28

^{1}H NMR (400 MHz, DMSO-d_6)

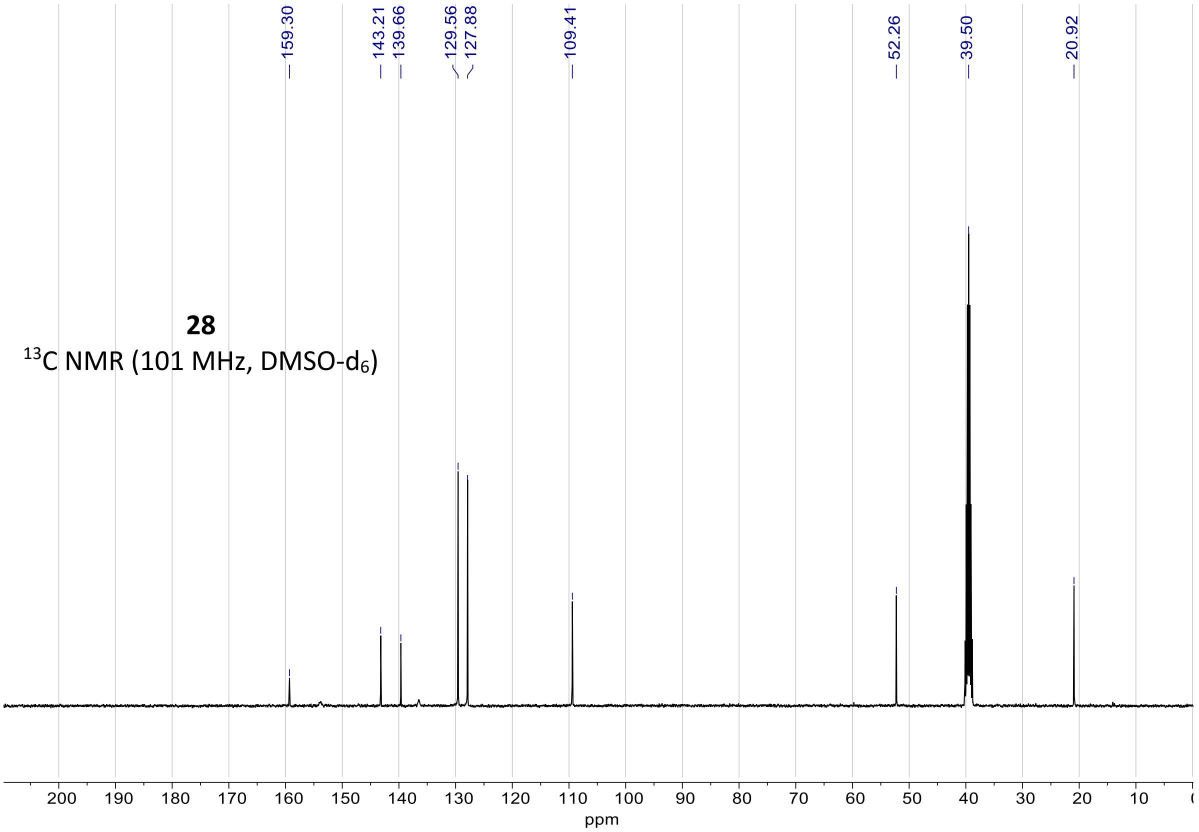

28

^{13}C NMR (101 MHz, DMSO-d_6)

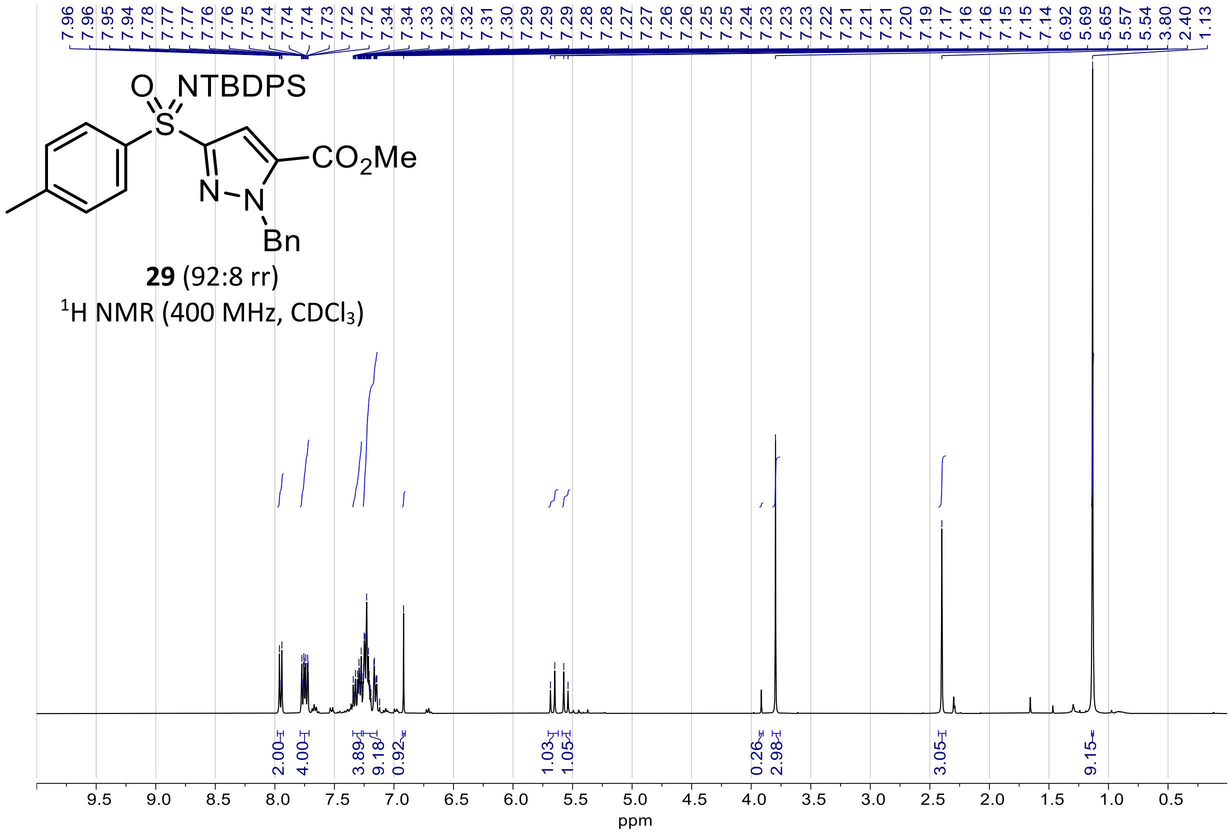
NTBDPS
CO_2Me
Bn
29 (92:8 rr)
1H NMR (400 MHz, CDCl3)
ppm

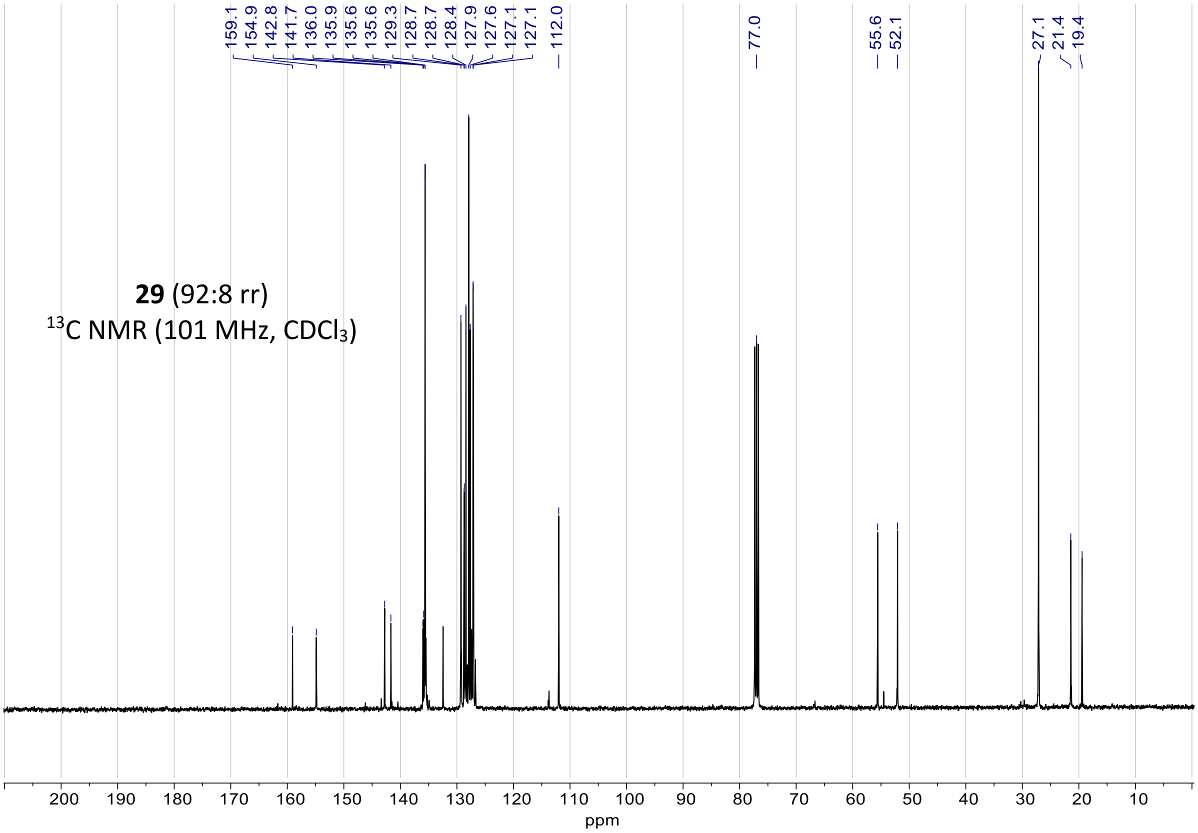
29 (92:8 rr)
13C NMR (101 MHz, CDCl3)
ppm

HPLC Traces of Enantioenriched Substrates and Products

(*S*)-(4-Bromophenyl)((*tert*-butyldiphenylsilyl)imino)(methyl)-λ^6-sulfanone ((*S*)-2d)

Conditions: Chiralpak IA column, 98:2 nhexane:iPrOH, flow rate: 1 mL min^{-1}, 35 °C, UV detection wavelength: 250 nm.

(*rac*)-(2d)

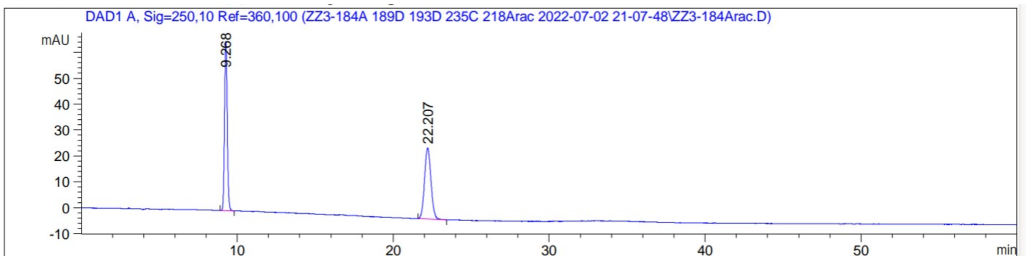

Signal 1: DAD1 A, Sig=250,10 Ref=360,100

Peak #	RetTime [min]	Type	Width [min]	Area [mAU*s]	Height [mAU]	Area %
1	9.268	BB	0.1865	786.67566	65.30374	50.1162
2	22.207	BB	0.4412	783.02728	27.41786	49.8838
Totals :				1569.70294	92.72161	

(*S*)-(2d)

$[\alpha]^{21}_D = -1$ (c 1.0, $CHCl_3$).

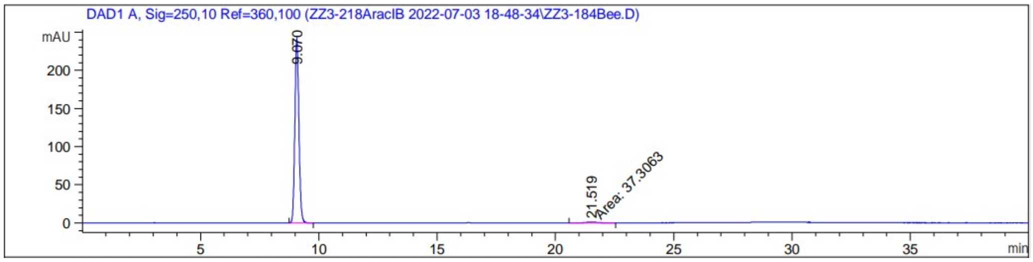

Signal 1: DAD1 A, Sig=250,10 Ref=360,100

Peak #	RetTime [min]	Type	Width [min]	Area [mAU*s]	Height [mAU]	Area %
1	9.070	BB	0.1830	2880.06543	241.79683	98.7212
2	21.519	MM	0.4848	37.30634	1.28254	1.2788
Totals :				2917.37177	243.07936	

ee = 97%

(*R*)-(4-Bromophenyl)((*tert*-butyldiphenylsilyl)imino)(2-oxopropyl)-λ^6-sulfanone ((*R*)-4d)

Conditions: Chiralpak IA column, 95:5 nhexane:iPrOH, flow rate: 1 mL min^{-1}, 35 °C, UV detection wavelength: 250 nm.

(*rac*)-(4d)

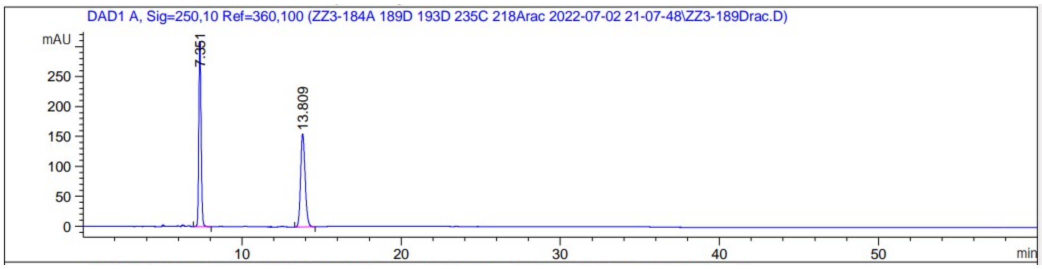

Signal 1: DAD1 A, Sig=250,10 Ref=360,100

Peak #	RetTime [min]	Type	Width [min]	Area [mAU*s]	Height [mAU]	Area %
1	7.351	BB	0.1534	3066.45850	310.13016	50.1042
2	13.809	BB	0.3035	3053.70898	155.26433	49.8958

Totals : 6120.16748 465.39449

(*R*)-(4d)

$[\alpha]^{22}_D$ = +33 (c 1.0, $CHCl_3$).

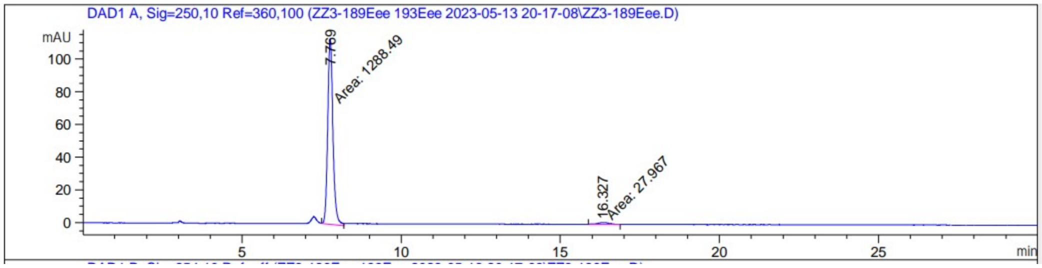

Signal 1: DAD1 A, Sig=250,10 Ref=360,100

Peak #	RetTime [min]	Type	Width [min]	Area [mAU*s]	Height [mAU]	Area %
1	7.769	MM	0.1891	1288.48828	113.58614	97.8756
2	16.327	MM	0.4240	27.96703	1.09940	2.1244

Totals : 1316.45531 114.68555

ee = 96%

(*R*)-(4-Bromophenyl)((*tert*-butyldiphenylsilyl)imino)(1-diazo-2-oxopropyl)-λ^6-sulfanone ((*R*)-6d)

Conditions: Chiralpak IA column, 98:2 nhexane:iPrOH, flow rate: 1 mL min^{-1}, 35 °C, UV detection wavelength: 250 nm.

(*rac*)-(6d)

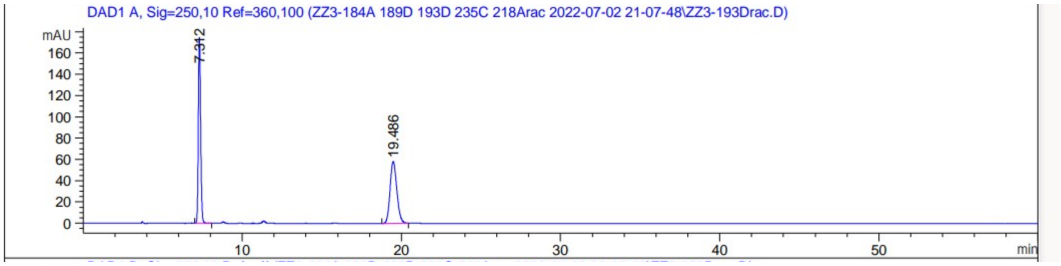

Signal 1: DAD1 A, Sig=250,10 Ref=360,100

Peak #	RetTime [min]	Type	Width [min]	Area [mAU*s]	Height [mAU]	Area %
1	7.312	BB	0.1551	1758.42041	175.18068	50.5244
2	19.486	BB	0.4555	1721.91943	58.14759	49.4756

Totals : 3480.33984 233.32827

(*R*)-(6d)

$[\alpha]^{22}_D$ = –33 (c 1.0, $CHCl_3$).

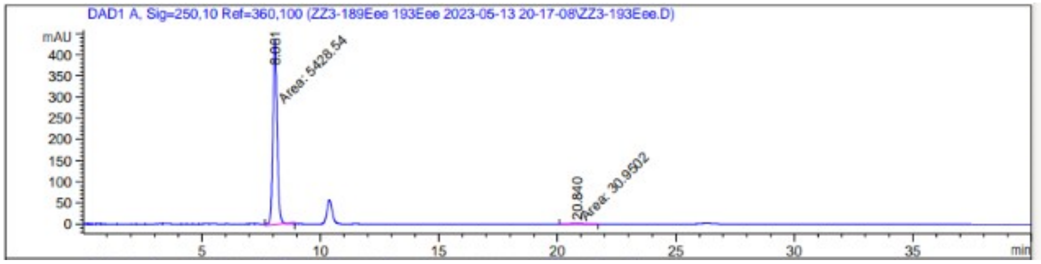

Signal 1: DAD1 A, Sig=250,10 Ref=360,100

Peak #	RetTime [min]	Type	Width [min]	Area [mAU*s]	Height [mAU]	Area %
1	8.081	MM	0.2067	5428.54492	437.79611	99.4331
2	20.840	MM	0.5874	30.95023	8.78157e-1	0.5669

Totals : 5459.49515 438.67427

ee = 99%

(*R*)-(4-Bromophenyl)((*tert*-butyldiphenylsilyl)imino)(diazomethyl)-λ^6-sulfanone ((*R*)-8d)

Conditions: Chiralpak IA column, 99:1 nhexane:iPrOH, flow rate: 1 mL min^{-1}, 35 °C, UV detection wavelength: 250 nm.

(*rac*)-(8d)

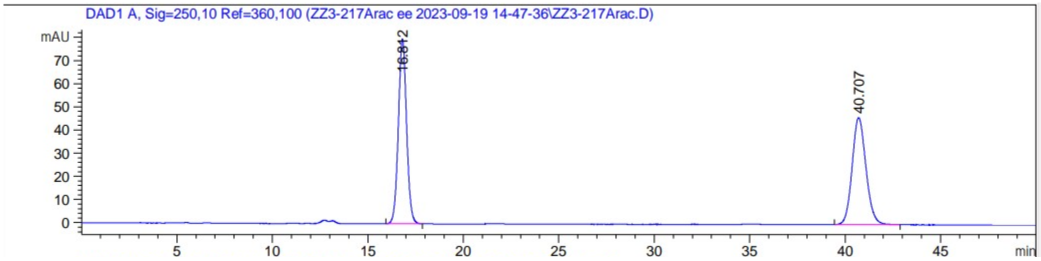

Signal 1: DAD1 A, Sig=250,10 Ref=360,100

Peak #	RetTime [min]	Type	Width [min]	Area [mAU*s]	Height [mAU]	Area %
1	16.812	BB	0.4581	2383.03516	79.87282	50.6274
2	40.707	BB	0.7706	2323.97559	46.22432	49.3726
Totals :				4707.01074	126.09713	

(*R*)-(8d)

$[\alpha]^{22}_D = -34$ (c 1.0, $CHCl_3$).

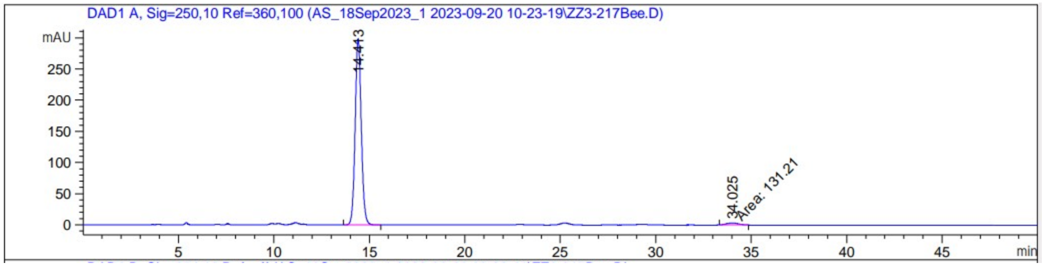

Signal 1: DAD1 A, Sig=250,10 Ref=360,100

Peak #	RetTime [min]	Type	Width [min]	Area [mAU*s]	Height [mAU]	Area %
1	14.413	BB	0.3410	6625.98047	298.47348	98.0582
2	34.025	MM	0.7030	131.20999	3.11093	1.9418
Totals :				6757.19046	301.58441	

ee = 96%

Methyl (*R*)-3-(4-bromo-*N*-(*tert*-butyldiphenylsilyl)phenylsulfonimidoyl)-1*H*-pyrazole-5-carboxylate ((*R*)-9d)

Conditions: Chiralpak IA column, 90:10 nhexane:iPrOH, flow rate: 1 mL min^{-1}, 35 °C, UV detection wavelength: 250 nm.

(*rac*)-(9d)

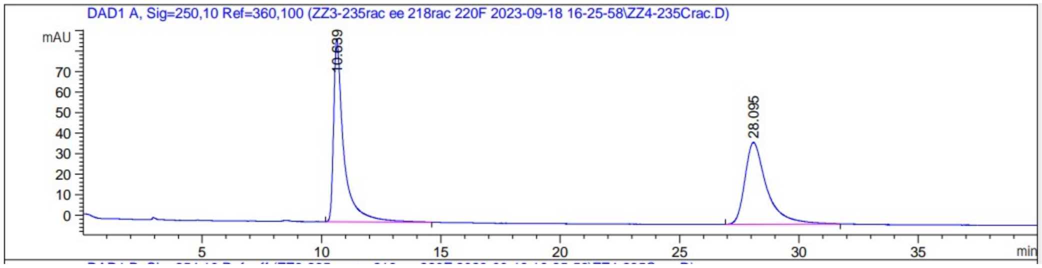

Signal 1: DAD1 A, Sig=250,10 Ref=360,100

Peak #	RetTime [min]	Type	Width [min]	Area [mAU*s]	Height [mAU]	Area %
1	10.639	BB	0.4207	2646.51196	89.06773	51.0460
2	28.095	BB	0.9253	2538.04761	39.83675	48.9540
Totals :				5184.55957	128.90448	

(*R*)-(9d)

$[\alpha]^{22}_{D}$ = □3 (c 1.0, $CHCl_3$).

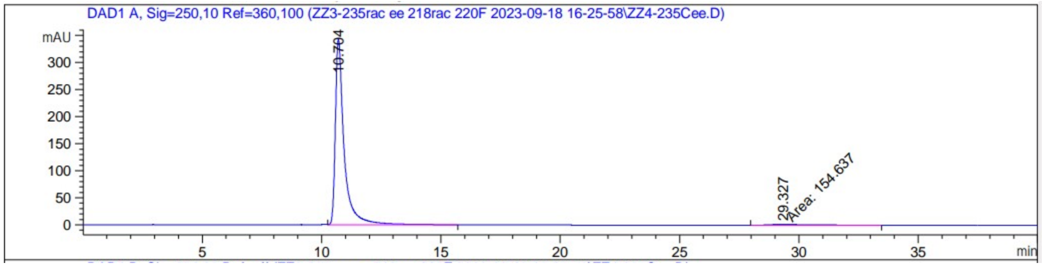

Signal 1: DAD1 A, Sig=250,10 Ref=360,100

Peak #	RetTime [min]	Type	Width [min]	Area [mAU*s]	Height [mAU]	Area %
1	10.704	VB	0.3731	8883.99316	344.25372	98.2892
2	29.327	MM	1.6875	154.63669	1.52729	1.7108
Totals :				9038.62985	345.78102	

ee = 97%

Dimethyl (*R*)-3-(4-bromo-*N*-(*tert*-butyldiphenylsilyl)phenylsulfonimidoyl)-1*H*-pyrazole-4,5-dicarboxylate (*R*)-22d

Conditions: Chiralpak IA column, 95:5 nhexane:iPrOH, flow rate: 1 mL min^{-1}, 35 °C, UV detection wavelength: 250 nm.

(*rac*)-(22d)

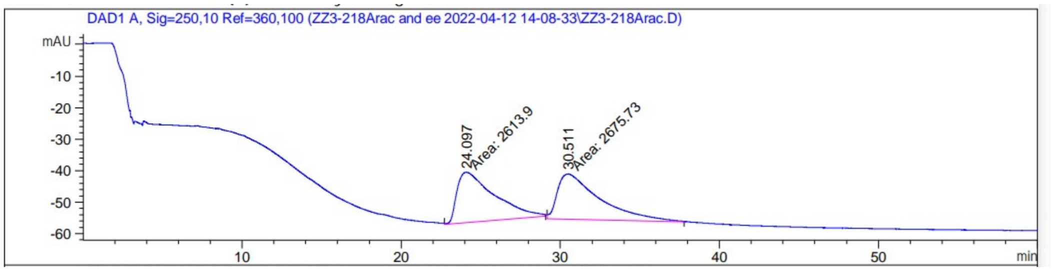

Signal 1: DAD1 A, Sig=250,10 Ref=360,100

Peak #	RetTime [min]	Type	Width [min]	Area [mAU*s]	Height [mAU]	Area %
1	24.097	MM	2.7105	2613.89966	16.07271	49.4156
2	30.511	MM	3.0919	2675.72632	14.42349	50.5844
Totals :				5289.62598	30.49619	

(*R*)-(22d)

$[\alpha]^{22}_{D}$ = +2 (c 1.0, $CHCl_3$).

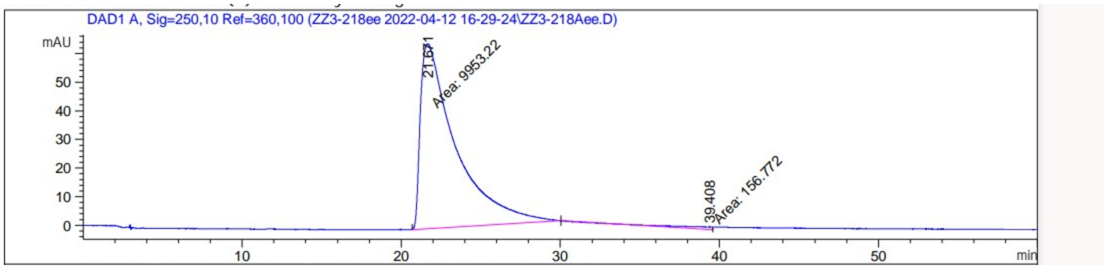

Signal 1: DAD1 A, Sig=250,10 Ref=360,100

Peak #	RetTime [min]	Type	Width [min]	Area [mAU*s]	Height [mAU]	Area %
1	21.671	MM	2.5672	9953.22461	64.61820	98.4493
2	39.408	MM	2.6235	156.77243	9.95932e-1	1.5507
Totals :				1.01100e4	65.61413	

ee = 97%

References

(1) (a) Briggs, E. L.; Ma, T.; Zhong, Z.; Tota, A.; Degennaro, L.; Luisi, R.; Bull, J. A., Synthesis of Enantioenriched NH-Sulfoximines by NH Transfer to Sulfoxides Using Ammonium Carbamate and (Diacetoxyiodo)benzene. *Org. Synth.* **2023**, *100*, 186-198. (b) Tota, A.; Spennacchio, M.; Briggs, E. L.; Ma. T.; Zhong, Z.; Degennaro, L.; Bull, J. A.; Luisi, R. Synthesis of NH-Sulfoximines from Sulfides Using Ammonium Carbamate and (Diacetoxyiodo)benzene to Transfer NH and O. *Org. Synth.* **2023**, 100, 48-60.

(2) Liu, J.; Chen, G.; Xing, J.; Liao, J. *tert*-Butanesulfinylthioether Ligands: Synthesis and Application in Palladium-Catalyzed Asymmetric Allylic Alkylation. *Tetrahedron: Asymmetry* **2011**, *22*, 575-579.

(3) Wang, T.-T.; Yang, F.-L.; Tian, S.-K., Copper-Catalyzed Sulfenylation of Boronic Acids with Sulfonyl Hydrazides. *Adv. Synth. Catal.* **2015,** *357*, 928-932.

(4) Morgan, K. F.; Hollingsworth, I. A.; Bull, J. A., Studies on the synthesis, stability and conformation of 2-sulfonyl-oxetane fragments. *Org. Biomol. Chem.* **2015,** *13*, 5265-5272.

(5) Degennaro, L.; Tota, A.; De Angelis, S.; Andresini, M.; Cardellicchio, C.; Capozzi, M. A.; Romanazzi, G.; Luisi, R., A Convenient, Mild, and Green Synthesis of NH-Sulfoximines in Flow Reactors. *Eur. J. Org. Chem.* **2017,** *2017*, 6486-6490.

(6) Kristensen, S. K.; Laursen, S. L. R.; Taarning, E.; Skrydstrup, T., Ex Situ Formation of Methanethiol: Application in the Gold(I)-Promoted Anti-Markovnikov Hydrothiolation of Olefins. *Angew. Chem. Int. Ed.* **2018,** *57*, 13887-13891.

(7) Tota, A.; Zenzola, M.; Chawner, S. J.; John-Campbell, S. St; Carlucci, C.; Romanazzi, G.; Degennaro, L.; Bull, J. A.; Luisi, R. Synthesis of NH-Sulfoximines from Sulfides by Chemoselective One-pot N- and O-Transfers. *Chem. Commun.* **2017**, *53*, 348-351.

(8) Zenzola, M.; Doran, R.; Degennaro, L.; Luisi, R.; Bull, J. A., Transfer of electrophilic NH using convenient sources of ammonia: Direct synthesis of NH sulfoximines from sulfoxides. *Angew. Chem. Int. Ed.* **2016,** *128*, 7319-7323.

(9) Frings, M.; Bolm, C.; Blum, A.; Gnamm, C. Sulfoximines from a Medicinal Chemist's Perspective: Physicochemical and in vitro Parameters Relevant for Drug Discovery. *Eur. J. Med. Chem.* **2017**, *126*, 225-245.

(10) Okaura, H., Bolm, C. Rhodium-Catalyzed Imination of Sulfoxides and Sulfides: Efficient Preparation of N-Unsubstituted Sulfoximines and Sulfilimines. *Org. Lett.* **2004**, *6*, 1305-1307.

(11) Yu, H.; Li, Z.; Bolm, C., Iron(II)-Catalyzed Direct Synthesis of NH Sulfoximines from Sulfoxides. *Angew. Chem. Int. Ed.* **2018,** *57*, 324-327.

(12) Liu, D.; Liu, Z.; Ma, C.; Jiao, K.; Sun, B.; Wei, L.; Lefranc, J.; Herber, S.; Mei, T. Nickel-Catalyzed *N*-Arylation of NH-Sulfoximines with Aryl Halides via Paired Electrolysis. *Angew. Chem. Int. Ed*. **2021**, *60*, 9444-9449.

(13) Craven, G. B.; Briggs, E. L.; Zammit, C. M.; McDermott, A.; Greed, S.; Affron, D. P.; Leinfellner, C.; Cudmore, H. R.; Tweedy, R. R.; Luisi, R.; Bull, J. A.; Armstrong, A. Synthesis and Configurational Assignment of Vinyl Sulfoximines and Sulfonimidamides. *J. Org. Chem.* **2021**, *86*, 7403-7424.

(14) Sirvent, J. A.; Bierer, D.; Webster, R.; Lücking, U. Palladium-Catalyzed Direct α-Arylation of *p*-Methoxybenzyl Protected *S,S*-Dimethylsulfoximine. *Synthesis*, **2017**, *49*, 1024-1036.

(15) Bio, M.; Nkepang, G.; You, Y., Click and photo-unclick chemistry of aminoacrylate for visible light-triggered drug release. *Chem. Commun.* **2012,** *48*, 6517-6519.

(16) López, A.; Clark, T. B.; Parra. A.; Tortosa, M. Copper-Catalyzed Enantioselective Synthesis of β-Boron β-Amino Esters. *Org. Lett.* **2017**, *19*, 6272–6275.

(17) Liu, X.; Ming, W.; Luo, X.; Friedrich, A.; Maier, J.; Radius, U.; Santos, W. L.; Marder, T. B., Regio- and Stereoselective Synthesis of 1,1-Diborylalkenes via Brønsted Base-Catalyzed Mixed Diboration of Alkynyl Esters and Amides with BpinBdan. *Eur. J. Org. Chem.* **2020**, 1941-1946.

(18) Oakdale, J. S.; Sit, R. K.; Fokin, V. V., Ruthenium-Catalyzed Cycloadditions of 1-Haloalkynes with Nitrile Oxides and Organic Azides: Synthesis of 4-Haloisoxazoles and 5-Halotriazoles. *Chem. Eur. J.* **2014,** *20*, 11101-11110.

(19) Deng, D.; Zheng, J. Process for Preparing N-H or N-Alkyl 2-Propynamide. U.S. Patent WO2014037308A1, March 13, 2014.
(20) Herck. N.; Maes, D.; Unal, K,; Guerre, M.; Winne, J. M.; Du Prez, F. E. Covalent Adaptable Networks with Tunable Exchange Rates Based on Reversible Thiol-yne Cross-Linking. *Angew. Chem. Int. Ed.* **2020**, *59*, 3609-3617.
(21) Dai, W.; Lv, Y.; Wang, L.; Shang, S.; Chen, B.; Li, G.; Gao, S., Highly efficient oxidation of alcohols catalyzed by a porphyrin-inspired manganese complex. *Chem. Commun.* **2015,** *51*, 11268-11271.
(22) Weerasiri, K. C.; Gorden, A. E. V. Oxidation of Propargylic Alcohols with a 2-Quinoxalinol Salen Copper(II) Complex and tert-Butyl Hydroperoxide. *Eur. J. Org. Chem.* **2013**, 1546-1550.
(23) Majumder, B.; Pandey, G. Synthesis of 2-Azabicyclo[*m*.*n*.0]–Alkanes and Their Application towards the Synthesis of *Strychnos* and *Stemona* Classes of Alkaloids. *Eur. J. Org. Chem.* **2020**, 3883-3888.
(24) Chen, Z.; Trudell, M. L., A Simplified Method for the Preparation of Ethynyl P-Tolyl Sulfone and Ethynyl Phenyl Sulfone. *Synth. Commun.* **1994,** *24*, 3149-3155.
(25) Cervi, A.; Vo, Y.; Chai, C. L. L.; Banwell, M. G.; Lan, P.; Willis, A. C., Gold(I)-Catalyzed Intramolecular Hydroarylation of Phenol-Derived Propiolates and Certain Related Ethers as a Route to Selectively Functionalized Coumarins and 2H-Chromenes. *J. Org. Chem.* **2021,** *86*, 178-198.
(26) Leroy, J. Preparation of 3-Bromopropiolic Esters: Methyl and *tert*-Butyl 3-Bromopropiolates. *Org. Synth.* **1997**, 74, 212.
